# Ni-Catalyzed Stereoconvergent Reductive Dimerization of Bromocyclobutenes

**DOI:** 10.1021/acs.orglett.3c03909

**Published:** 2023-12-26

**Authors:** Philipp Spieß, Sergio Armentia Matheu, Adriano Bauer, Guilhem Coussanes, Saad Shaaban, Nuno Maulide

**Affiliations:** Institute of Organic Chemistry, University of Vienna, Währinger Straße 38, 1090 Vienna, Austria

## Abstract

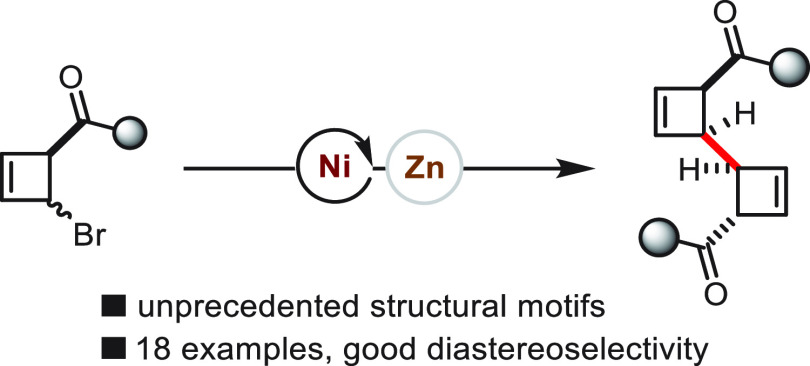

A nickel-catalyzed reductive dimerization of bromocyclobutenes to produce unusual and unprecedented cyclobutene dimers was developed. In a stereoconvergent procedure, various bromocyclobutenes were readily dimerized in good yields, with good diastereoselectivities and broad functional group tolerance. Notably, the presence of a carbonyl group in the starting material appears to dictate diastereoselectivity.

Cross-coupling reactions catalyzed by transition metals have become some of the most valuable synthetic tools over the years. Specifically, the union of a carbon electrophile [usually in the form of an organo(pseudo)halide] and a carbon nucleophile (such as an organometallic species) has proven its merit in a variety of contexts.^1−4^ In contrast, reductive electrophile–electrophile cross-couplings are less firmly established.

In line with the contemporary trend to “escape flatland”, as it commonly results from classical biaryl couplings,^5^ C–C_sp_^3^ couplings represent a particularly attractive scenario. However, such transformations also face the pervasive challenges of β-hydride elimination or hydrodehalogenation, which often hampers reaction success.^6^ Nickel has proven to be an especially useful catalytic tool in this context, as its propensity to engage in single-electron transfer (SET) processes allows various oxidation states to be accessed.^7,8^ Elegant nickel-promoted methods, including enantioconvergent coupling processes with C_sp_^3^ electrophiles, have therefore been developed.^9−17^

Cyclobutenes are important structural motifs present in a number of naturally occurring and biologically active compounds.^18,19^ However, their synthesis remains challenging and is often planned late in a synthetic pathway because of their high propensity for electrocyclic ring opening.^20,21^ Our group and others have developed valuable approaches to the synthesis of functionalized cyclobutenes^22−26^ and successfully applied them in natural product synthesis.^27−30^ In this context, 2-halo-cyclobutenes are particularly desirable building blocks.

Thus, we have previously reported a Pd-catalyzed diastereodivergent de-epimerization of 2-chloro-cyclobutenes with malonate nucleophiles which results in the formation of highly functionalized adducts **I** (Scheme 1 left) and enables the construction of two new stereocenters.^24^ In an effort to access even more complex cyclobutene scaffolds, we speculated whether a reductive dimerization of two cyclobutenes could lead to products such as **II**, carrying up to *four* contiguous stereocenters. Notably, **II** can be considered three-dimensional analogs of mono-*ortho*-substituted biaryls (Scheme 1 right and bottom). Herein, we report a stereoconvergent protocol that, for the first time, allows access to bis-cyclobutenes using nickel catalysis.

**Scheme 1:**
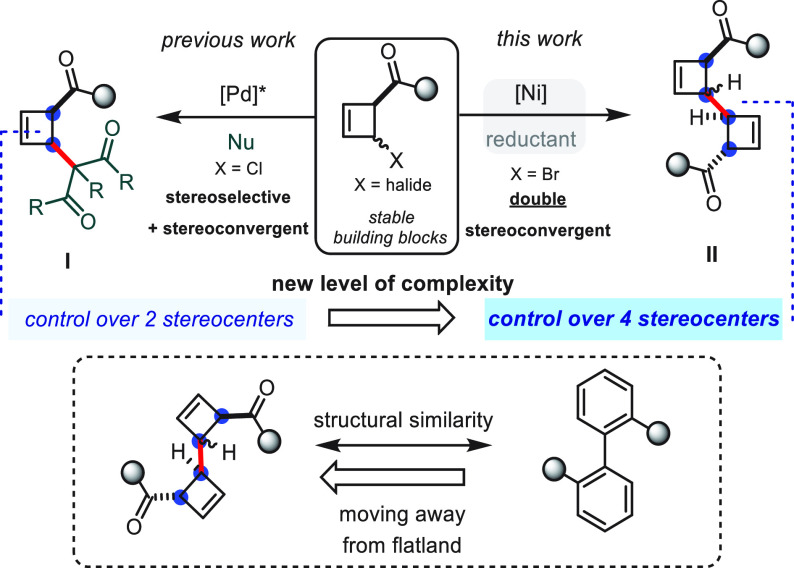
Transition-Metal-Catalyzed C-C Couplings of 2-Halo-Cyclobutenes

We began our investigations by examining the reaction of *trans*-bromocyclobutene **1a** (*trans*) under a variety of conditions (Scheme 2). After extensive experimentation, it was found that a catalytic amount of NiCl_2_·DME at room temperature affords a mixture of exclusively two diastereoisomeric cyclobutene dimers (**2aa** and **2ab, 2ac–2af** not observed) in good yield and stereoselectivity with the *cis,trans*-cyclobutene dimer (**2ab**) being obtained as the major isomer, as confirmed by X-ray analysis (see the Supporting Information for more details for the preparation of **2sa**).^31^ Subtle changes of the nickel source, as well as the reaction solvent or reductant, had a deleterious effect on either the diastereoselectivity or the conversion rate (see the Supporting Information for more details). Interestingly, it was also found that other halide analogs produced substantially worse outcomes and gave either lower yield and lower diastereoselectivity (iodocyclobutene), or resulted in a complete shutdown of reactivity (chlorocyclobutene). Employing the *cis*-configured stereoisomer led to a slightly higher yield with comparable diastereoselectivity, thus supporting the notion that this process is stereoconvergent. To further verify this outcome and affirm the scalability of the reaction, we conducted the dimerization on a 2 mmol scale using *trans*-cyclobutene **1a** and 5 mol % catalyst loading. The obtained yield was comparable with that of the small-scale reaction. Additionally, on a larger reaction scale (3.6 mmol), the same experiment was carried out using a mixture of *trans/cis*-**1a** (see the Supporting Information).

**Scheme 2:**
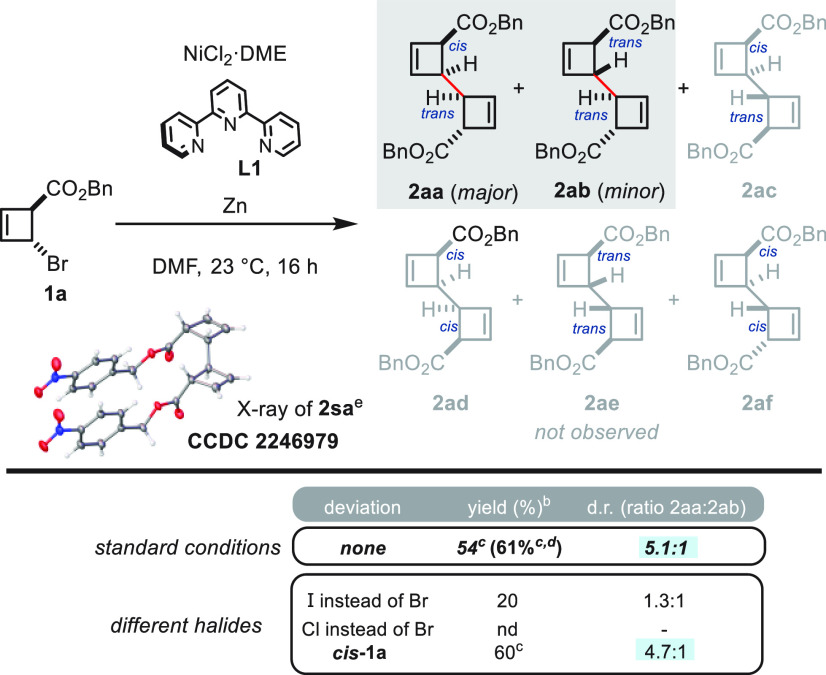
Optimization of Reaction Conditions Reactions performed on a 0.1 mmol scale with NiCl_2_·DME (18 mol %), **L1** (18 mol %), and Zn nanopowder (4 equiv). NMR yield using CH_2_Br_2_ as an internal standard. Isolated yield. Reaction was performed on 2 mmol scale with 5 mol % catalyst loading; d.r. 4.3:1. Accessed by esterification of compound **7** (from Scheme 4B); for more information, see the Supporting Information.

With the optimized conditions in hand, we focused on exploring the generality of this process. As shown in Scheme 3, various dimers of cyclobutenes bearing benzyl ester derivatives, both electron-rich (**2b**) and electron-deficient (**2c**) but also sterically hindered (**2d**), and heterocyclic structures (**2e**) were accessed in good yields and moderate to good diasteroselectivities. Alkyl cyclobutene esters reacted smoothly (**2f**–**2j**) and allowed even a bulky group (*tert*-butyl moiety in **2f**) to be in close proximity to the reaction center (Scheme 4). However, considering the results of substrates **2d** and **2f**, which show lower diastereomeric ratios with still comparable yields to the related benzyl (**2a**–**2c**) and alkyl esters (**2g**–**2i**), it clearly stands out that steric hindrance seems to have a detrimental effect on the diastereoselective outcome of the reaction and suggests a non-innocent effect of the ester functionality. Importantly, dimer **2g**, bearing TMSE (trimethylsilyl ethyl) esters, could be accessed—the presence of this group allows mild cleavage to reveal the cyclobutene carboxylic acid dimer.^24^ Notably, alkenyl (**2k**–**2l**) and propargyl (**2m**) esters, usually incompatible with state-of-the-art transition metal catalysts, were tolerated in this process. Similarly, cyclobutenes carrying phenyl esters were successfully dimerized (**2n**), which allowed for the presence of other esters (**2o**). Finally, our method could also be extended to thioesters (**2p**–**2r**), which showed excellent diastereoselectivity of up to 7.7:1 for the dimerized products.

**Scheme 3:**
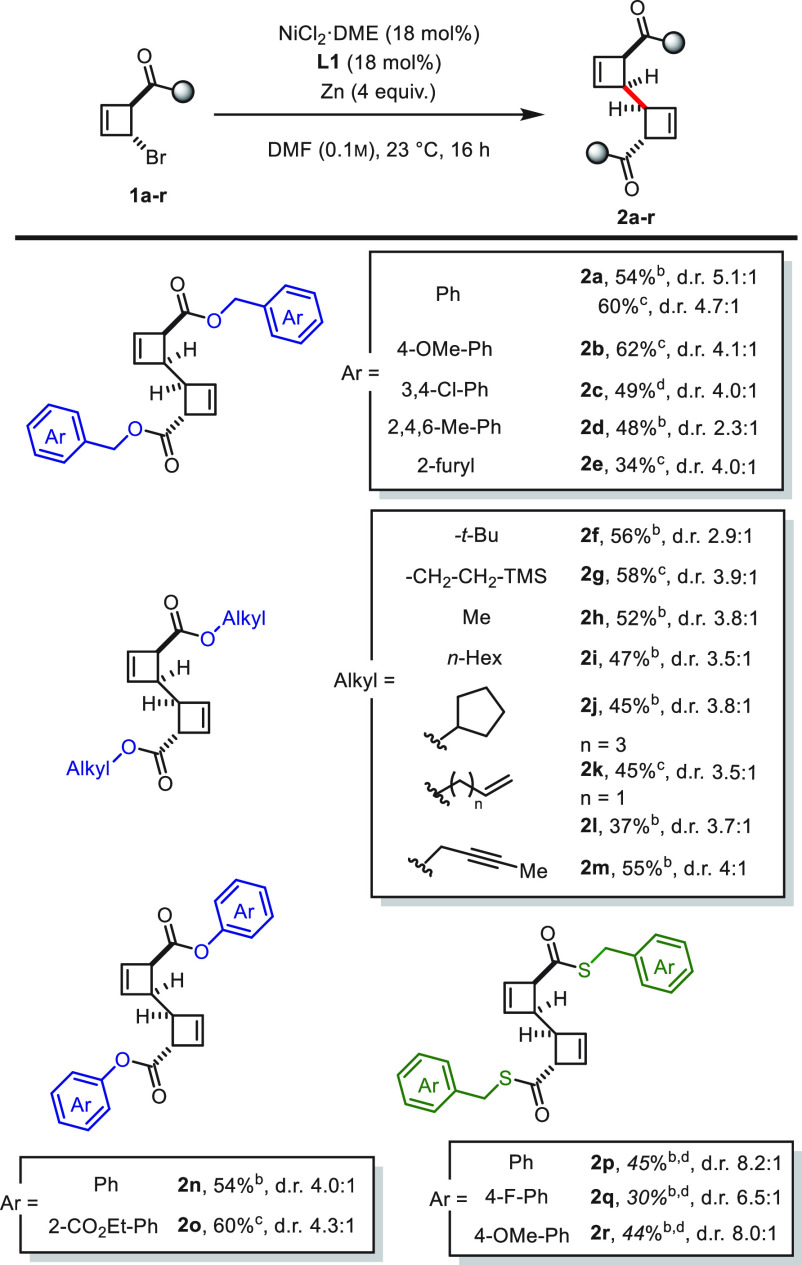
Reaction Scope for the Dimerization of 2-Bromocyclobutenes Reactions performed on a 0.1 mmol scale with NiCl_2_·DME (18 mol %), **L1** (18 mol %), and Zn nanopowder (4 equiv). From *trans*-configured starting material. From *cis*-configured starting material. Reaction time of 30 min, instead of 16 h.

**Scheme 4:**
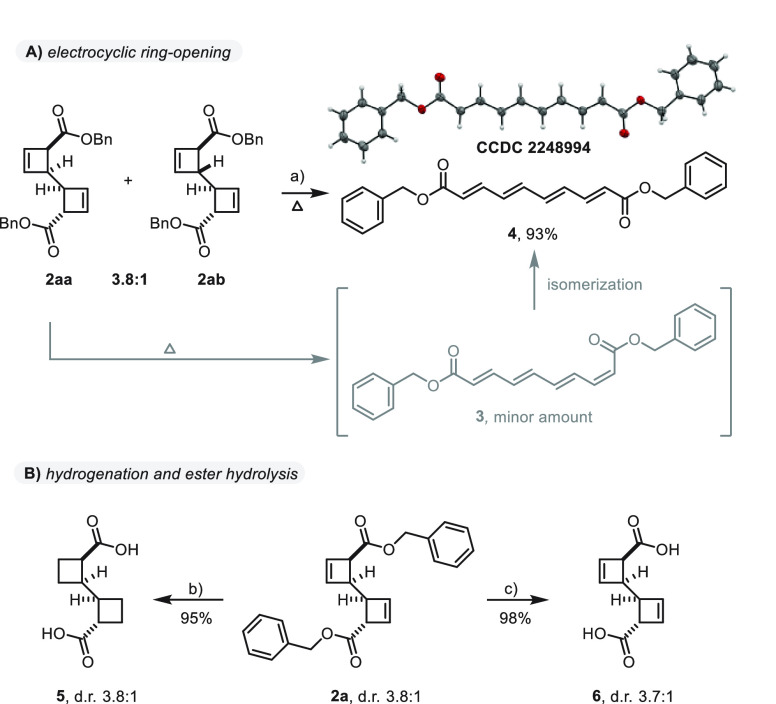
Derivatization of Cyclobutene Dimers Toluene, 70 °C, 5 d. Pd/C (10 mol %), H_2_ (1 atm), MeOH/EtOAc, 23 °C, 16 h. LiOH (2.2 equiv), THF/water, 23 °C, 15 h.

Next, we turned our attention to possible derivatization reactions. Surprisingly, heating of substrates **2aa** and **2ab** to 70 °C almost exclusively resulted in the pure *trans*-tetraene **6**, a structure confirmed by X-ray analysis (Scheme 4A). Shorter reaction times or lower reaction temperatures allowed only small amounts of ring-opened cyclobutene dimer to be formed, which demonstrated its unexpectedly high robustness to thermal conditions. Importantly, small amounts of isomer **3** were observed to be derived from diastereoisomer **2aa**, which can be taken as evidence of a rapid isomerization reaction of **3** to **4**. In addition, **2a** underwent both hydrogenation of the double bond and hydrogenolysis of the benzyl groups to yield carboxycyclobutane dimer **5**, whereas selective ester cleavage of **2a** was easily achieved, which furnished the corresponding cyclobutene carboxylic acid dimer **6** with no erosion of the diastereomeric ratio (Scheme 4B).^32^

Finally, we turned our attention to the reaction pathway and the origin of diastereoselectivity (Scheme 5). Given the previously observed difference in diastereoselectivity between thioesters and esters (see Scheme 3, with examples **2a** and **2p** or **2b** and **2r**), we wondered whether the nature of the carbonyl group might facilitate temporary coordination to the catalyst during oxidative addition.^33^ Hence, bromocyclobutene **7** carrying a *benzyl ether* instead of an ester was subjected to the optimized reaction conditions (Scheme 5A). The desired dimer **8** was obtained in moderate yield and very low d.r. (nearly 1:1) that, indeed, suggested the involvement of coordination by the carbonyl group. Furthermore, the diastereomeric ratio of the dimerization of **1a** to yield **2a** was followed over time and shown to remain unchanged, thereby ruling out the possibility that an epimerization event leading to the observed diastereomeric ratios could occur after product formation (see the Supporting Information for more details).

**Scheme 5:**
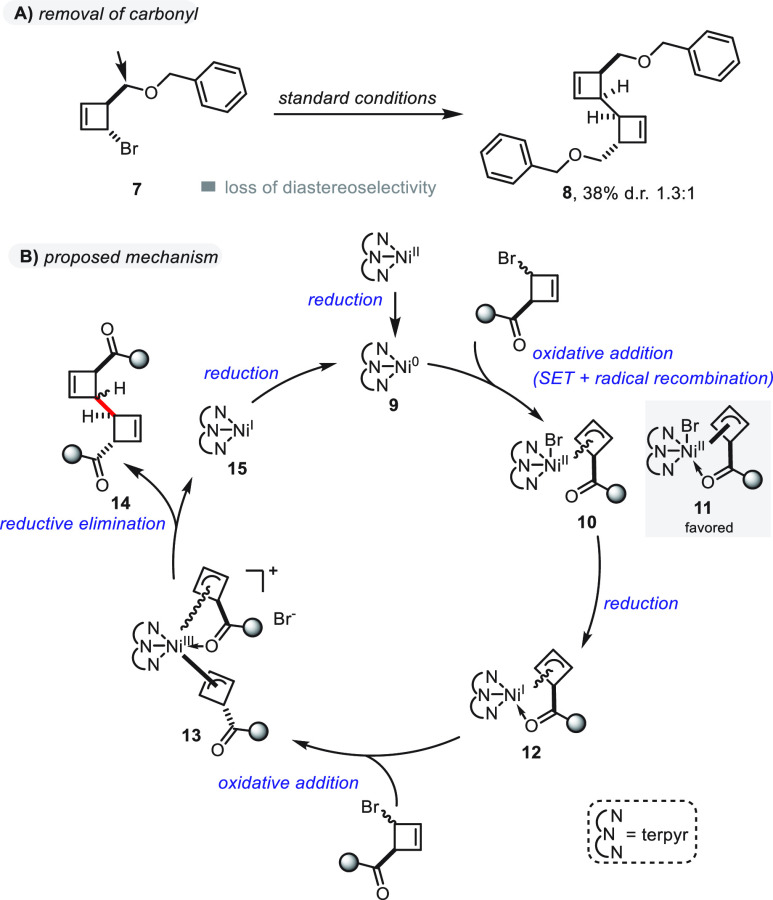
Mechanistic Experiment and Proposed Catalytic Reaction Cycle

On the basis of these results and important precedents,^34−37^ we propose a mechanism for this transformation in Scheme 5B. At the outset, Ni(II) is reduced to Ni(0) to set the stage for oxidative addition of the bromocyclobutene, which forms an allylnickel(II) complex (**10**). Along with the result of Scheme 5A,^33^ the radical recombination of nickel complex and allyl radical seems more likely to result in a *cis*-configured cyclobutene, forming complex **11** as the major species because of the coordination effect of an ester/thioester functionality.^38^ Another reduction step to form the Ni(I) complex (**12**) then takes place with a second equivalent of bromocyclobutene subsequently being introduced to form **13**. Given the expected steric congestion around the nickel center, in combination with a saturated coordination sphere, it is likely that the second cyclobutene only adds when being *trans*-configured to the nickel complex. Lastly, complex **13** is prone to reductive elimination, which gives the homocoupled product **14** and completes the catalytic cycle after another Ni(I)-to-Ni(0) reduction.

Finally, we speculated whether the bromocyclobutene could also be used in a reductive heterocoupling by carefully selecting a suitable second electrophilic partner. After screening various alkyl bromides and iodides (see the Supporting Information for a complete list of tested partners), we were, indeed, able to achieve a cross-coupling using cyclohexyl iodide (Scheme 6). As previously observed in the dimerization of cyclobutenes, this process proved to be stereoconvergent and yielded 17 as a single diasteroisomer in *trans* configuration^39^ starting from either *trans***-1a** or *cis***-1a**. Considering this stereochemical result, the ester functionality does not seem to play any role in this cross-coupling. We concluded that, because of the enhanced reactivity of the second reactant (**16**), radical recombination of an allyl radical with a Ni complex should occur only after formation of a Ni-alkyl complex, thereby preventing any carbonyl-directed oxidative addition (for a proposed mechanism of this reaction, see the Supporting Information).

**Scheme 6:**
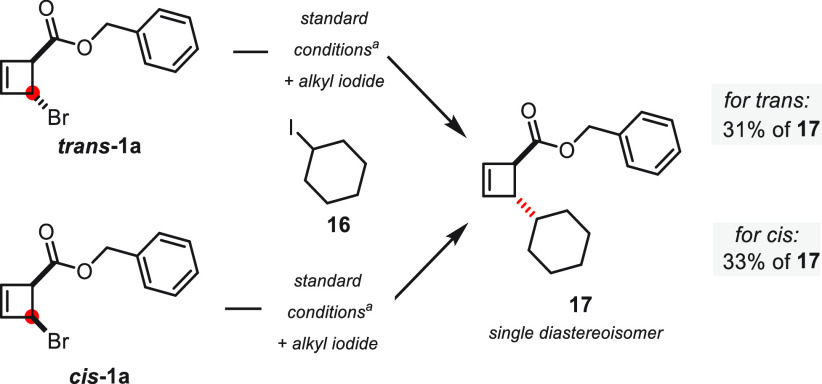
Stereoconvergent Reductive Heterocoupling of Bromocyclobutene with Alkyl Iodide Reactions performed on a 0.1 mmol scale with NiCl_2_·DME (18 mol %), **L1** (18 mol %), Zn nanopowder (4 equiv), and **16** (3.0 equiv).

In summary, we have developed the first Ni-catalyzed reductive dimerization of bromocyclobutenes in which the carbonyl function appears to be responsible for the observed diastereoselectivity. The reaction tolerates a wide range of cyclobutenes bearing ester and thioester moieties to give the desired dimers in good yields and diastereoselectivities. Initial investigations and results on a reductive heterocoupling point to further possibilities in this area.

## Data Availability

The data underlying this study are available in the published article and its Supporting Information.

## Supporting Information Available

Experimental procedures and characterization data (^1^H and ^13^C NMR) for all new compounds (PDF)
