# Supplementary material for: Ni-Catalyzed Stereoconvergent Reductive Dimerization of Bromocyclobutenes
Source: Org Lett. 2023 Dec 26;26(1):355–9. doi: 10.1021/acs.orglett.3c03909 (PMC10789092; doi:10.1021/acs.orglett.3c03909)
Supplement: Supplementary file 1 — ol3c03909_si_001.pdf [file ol3c03909_si_001.pdf]

Ni-catalyzed Stereoconvergent Reductive Dimerization of Bromo-Cyclobutenes

Philipp Spieß[#], Sergio Armentia Matheu[#], Adriano Bauer, Guilhem Coussanes, Saad Shaaban,

Nuno Maulide*

Institute of Organic Chemistry, University of Vienna, Währinger Straße 38, 1090 Vienna (Austria)
E-Mail: nuno.maulide@univie.ac.at, Homepage: http://maulide.univie.ac.at

Table of Content

1. General Information **2**

2. Optimization of the Reaction Conditions **2**

3. Experimental **7**

3.1. Synthesis of the 4-bromocyclobut-2-ene-1-carboxylic acid (S1, S2) *7*

3.2. General Procedures *10*
3.2.1. General Procedure 1 (GP1): Synthesis of Esters via in-situ formed Carbonyl Chlorides 10
3.2.2. General Procedure 2 (GP2): Synthesis of Esters using T3P 10
3.2.2. General Procedure 3 (GP3): Synthesis of thioesters via Steglich coupling 10
3.2.3. General Procedure 4 (GP4): Dimerization of 4-Bromocyclobutenes 12

3.3. Characterization of Starting Materials *13*

3.4. Characterization of Cyclobutene Dimers *30*

3.5. Unsuccessful and low-yielding Substrates *44*

3.6. Application *45*
3.6.1. Thermal Ring-Opening of Dimer 2a 45
3.6.2 Hydrogenation of Cyclobutene Dimer 2k 47
3.6.3. Hydrogenolysis + Hydrogenation of Dimer 2a 48
3.6.4. Ester Hydrolysis of Dimer 2a 49

3.7. Mechanistic Studies *50*
3.7.1. Dimerization with Ether 7 50
3.7.2. Epimerization Study of 2a 51

3.8. Determination of the Relative Stereochemistry of Cyclobutene Dimers *52*

3.9. Reductive Heterocoupling of Bromo-Cyclobutene with Alkyl Halides *55*
3.9.1. General Procedure for Heterocoupling 55
3.9.2. Proposed Mechansim 57

4. References **58**

5. NMR Spectra **59**

6. X-Ray Crystallographic Data **117**

1. General Information

Unless otherwise stated, all glassware was flame-dried before use and all reactions were performed under an atmosphere of argon. All solvents were distilled from appropriate drying agents prior to use or directly taken from commercial sealed bottles under an atmosphere of argon. All reagents were used as received from commercial suppliers unless otherwise stated. Reaction progress was monitored by thin layer chromatography (TLC) performed on aluminum plates coated with silica gel F254 with 0.2 mm thickness. Chromatograms were visualized by fluorescence quenching with UV light at 254 nm or by staining using potassium permanganate. Flash column chromatography was performed using silica gel 60 (230-400 mesh, Merck and co.). Neat infrared spectra were recorded using a Perkin-Elmer Spectrum 100 FT-IR spectrometer. Wavenumbers (ν_{max}) are reported in cm^{-1}. Mass spectra were obtained using a Finnigan MAT 8200 or (70 eV) or an Agilent 5973 (70 eV) spectrometer, using electrospray ionization (ESI). All ^{1}H NMR and ^{13}C NMR spectra were recorded using a Bruker AV-400, AV-600 spectrometer or AV-700 spectrometer at 300K. Chemical shifts are given in parts per million (ppm, δ), referenced to the solvent peak of $CDCl_3$, defined at δ = 7.26 ppm (^{1}H NMR) and δ = 77.16 (^{13}C NMR) or MeOD, defined at δ = 3.31 ppm (^{1}H NMR) and δ = 49.00 (^{13}C NMR). Coupling constants are quoted in Hz (*J*). ^{1}H NMR splitting patterns are designated as singlet (s), doublet (d), triplet (t), quartet (q) and quintet (quint) as they appeared in the spectrum. If the appearance of a signal differs from the expected splitting pattern, the observed pattern is designated as apparent (app). Splitting patterns that could not be interpreted or easily visualized are designated as multiplet (m) or broad (br). Structural assignments were made with additional information from gCOSY, gHSQC, and gHMBC experiments.

Note: In all work with cyclobutenes, the temperature was kept at a maximum of 30°C. This also applies to the evaporation of the solvent. At temperatures above 30 °C, partial ring opening can be observed for most of the compounds.

2. Optimization of the Reaction Conditions

General procedure (0.1 mmol scale):

A flame-dried Schlenk flask under argon was loaded with nickel source, ligand and reducing agent. Then the solvent (0.8 mL, 0.125 M) was added and the mixture was stirred for 5 minutes. The corresponding 4-halo-cyclobutene substrate (0.1 mmol, 1.0 eq.) was added using a Hamilton syringe. The resulting mixture was stirred for 16 h and the crude material was transferred to a separatory funnel with Et_2O. The organic phase was washed with water/brine mixture (1:1, 5x), dried over anhydrous $MgSO_4$, and the solvent was removed under reduced pressure. The crude material was analyzed adding an internal standard (CH_2Br_2).

Table 1. Optimization on the nickel source for iodo-cyclobutene dimerization.

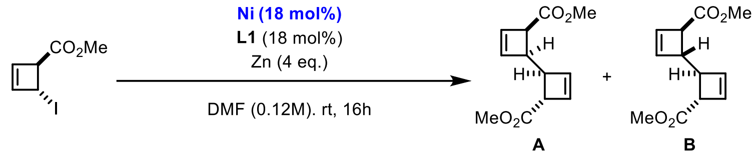

entry	nickel source	GC yield (%)
1	$NiCl_2$ DME	18%, multiple isomers
2	$NiCl_2$	15%, multiple isomers
3	$NiCl_2$ H_2O	14%, multiple isomers
4	$NiBr_2$ DME	24%, d.r. 1.2:1 (A:B)
5	$NiBr_2$	11%, multiple isomers
6	$Ni(OTf)_2$	13%, mutliple isomers

Table 2. Screening different ligands for iodo-cyclobutene dimerization.

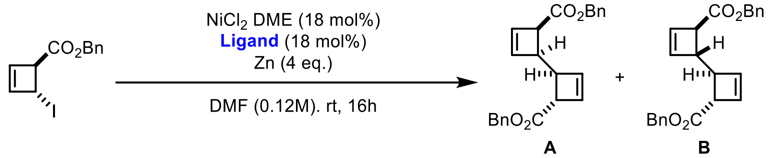

d.r. = ratio of A/B

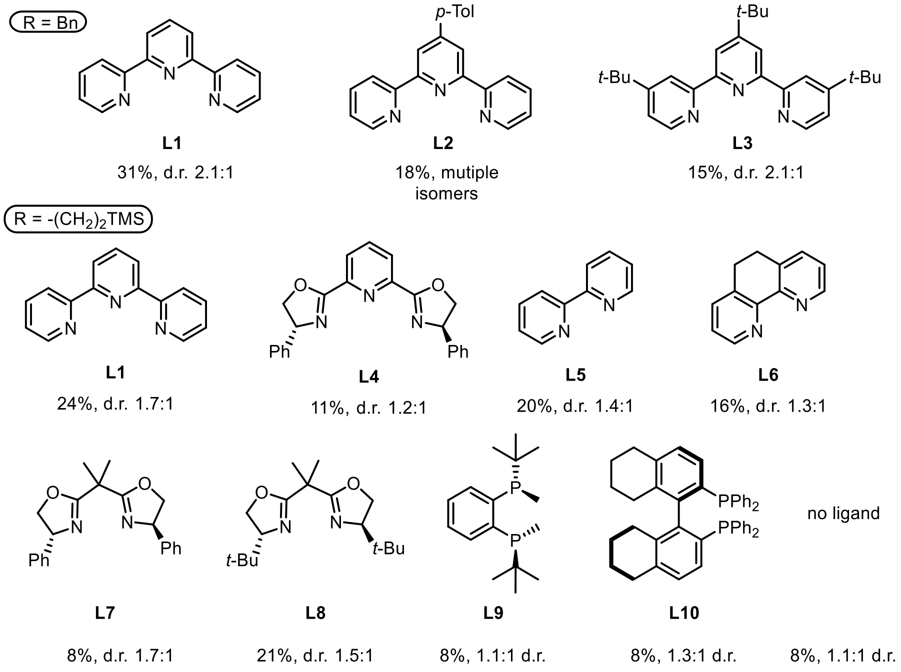

Table 3. Testing various halo-cyclobutenes.

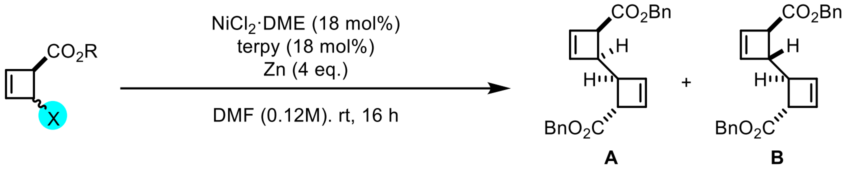

entry	X	configuration	R	NMR yield (%)	d.r. (A:B)
1	I	trans	-Bn	20	1.3:1
2	Br	trans	-Bn	52 (54)	5.1:1
3	Br	cis	-Bn	63 (60)	4.7:1
4	Cl	cis	$-CH_2CH_2TMS$	18	1.3:1
5	Cl	cis	-Bn	nd	-

Table 4. Testing nickel sources for bromo-cyclobutenes dimerization.

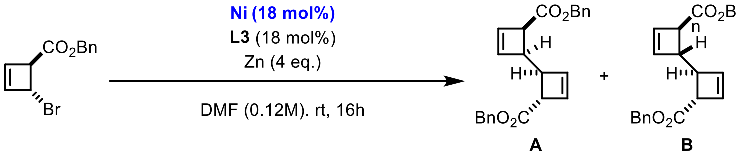

entry	nickel source	NMR yield (%)	d.r. (A:B)
1	$NiCl_2$ DME	50	4.3:1
2	$NiBr_2$ DME	32	2.4:1

Table 5. Screening ligands for bromo-cyclobutenes dimerization.

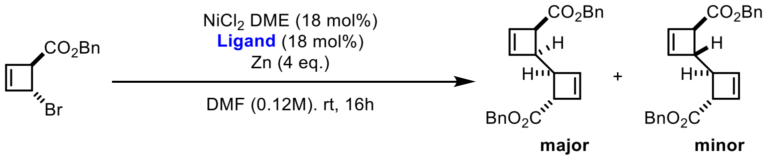

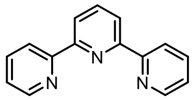

L1

50%, d.r. 4.3:1

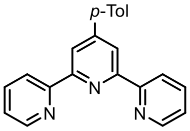

L2

6%, d.r. 5:1
70% SM

Table 6. Solvent screening.

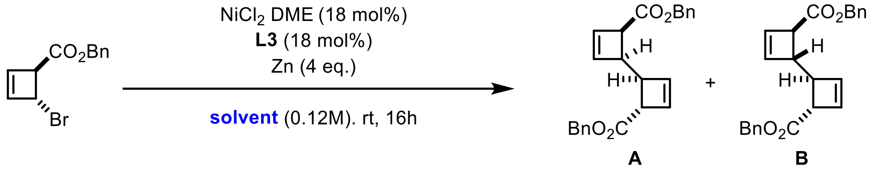

entry	solvent	NMR yield (%)	d.r. (A:B)
1	DMA	no conversion	-
2	DMF	52 (54)	5.5:1
3	MeCN	no conversion	-
4	DMSO	19	4.3:1
5	NMP	16	3.6:1
6	dioxane	no conversion	-
7	toluene	no conversion	-
8	DCM	no conversion	-
9	DME	no conversion	-
10	DMPU	50	2.1:1

Table 7. Reductants screening.

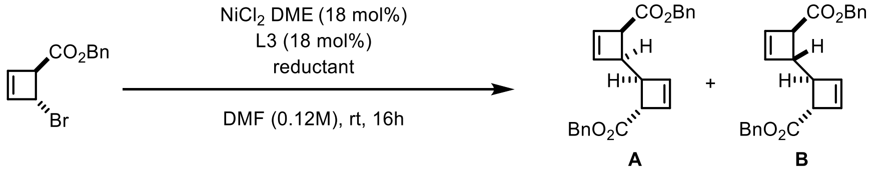

entry	reductant	NMR yield (%)	d.r. (A:B)
1	Zn* (4.0 eq.)	48	4.3:1
2	Zn* (3.0 eq.)	34	3.9:1
3	Zn* (2.0 eq.)	45	4.0:1
4	Zn* (1.4 eq.)	27	5:8:1
5	Mn (1.2 eq.)	38	2.5:1
6	Zn** (4.0 eq.)	43	3:1
7	Zn*** (4.0 eq.)	52	5.5:1

* activated zinc ** zinc powder *** zinc nanopowder

Table 8. Catalyst's loading.

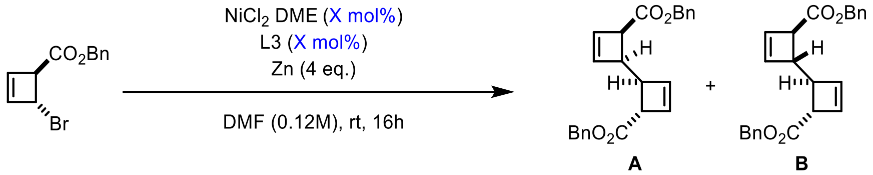

entry	Ni (mol%)	ligand (mol%)	NMR yield (%)	d.r. (A:B)
1	18	18	50	4.3:1
2	5	5	25	3.0:1

Table 9. Screening of chiral ligands for an enantioselective version.

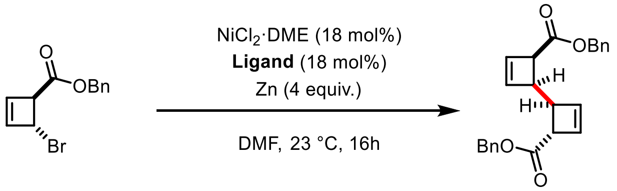

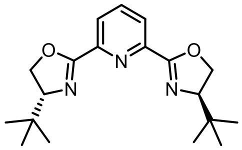

mixture of cyclobutenes

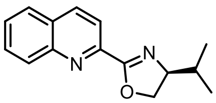

complex mixture
no cyclobutene species

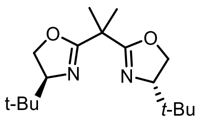

isolated:
32%, d.r. 2.6:1.2:1
➡ ee wasn't measured as 3 isomers were obtained

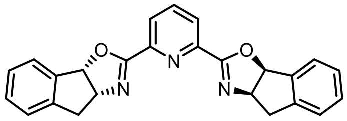

complex mixture
no product

3. Experimental

Note: In all work with cyclobutenes, the temperature was kept at a maximum of 30°C (better 25°C). This also applies to the evaporation of the solvent. At temperatures above 30 °C, partial ring opening can be observed for some of the compounds. In TLC analysis of cyclobutenes under UV light, this thermal ring-opening property could be used as an advantage to easily detect cyclobutenes after gently heating the corresponding TLC plates with a heat gun. In all cases, the resulting diene/tetraene reaction products were exceptionally UV active.

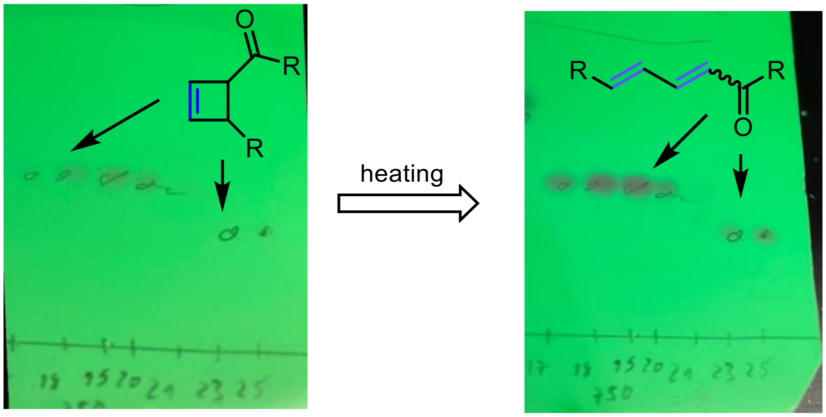

3.1. Synthesis of the 4-bromocyclobut-2-ene-1-carboxylic acid (S1, S2)

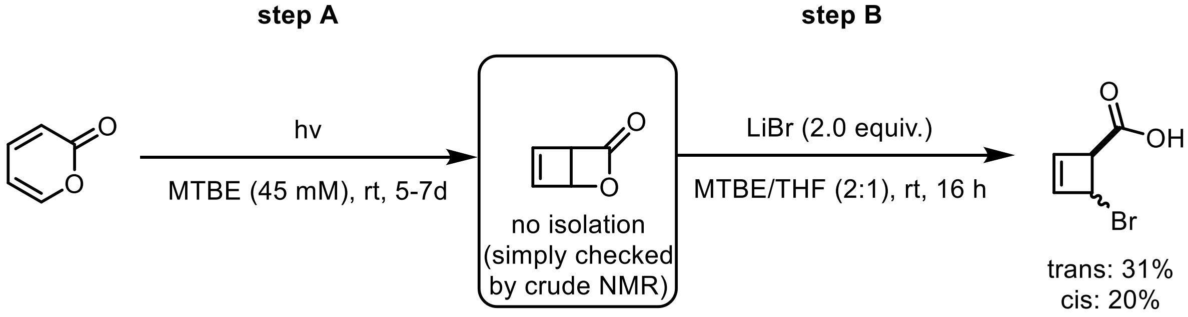

Step A:

A flame-dried 250 mL Schlenk flask was loaded with 2-pyrone (865 mg, 9.0 mmol, 1.0 equiv.) dissolved in MTBE (200 mL, 0.045 M). The solution was degassed (using an Argon balloon) and then irradiated in a *Rayonet* photoreactor for 5-7 days at room temperature until full conversion was achieved. The reaction progress was hereby followed by ^{1}H-NMR by taking a smaller aliquot (direct mixing with deuterated solvent, i.e. $CDCl_3$). The final reaction solution could be stored in a fridge (4°C) prior to use and did not show any signs of decomposition after several weeks.

^{1}H NMR (400 MHz, CDCl$_3$): δ 6.73 (app. t, *J* 3.5, 1H), 6.54 (app. t, *J* 1.9, 1H), 5.29 (dd, *J* 4.5, 1.9, 1H), 4.39 (s, 1H) ppm. NMR Data of 2-oxabicyclo[2.2.0]hex-5-en-3-one matched those reported in the literature.[1]

Step B:

An oven-dried Schlenk flask was loaded with LiBr (1.56 g, 18 mmol, 2.0 eq.) then dried with a flame gun until all the water from the hygroscopic LiBr was removed. Then, THF (100 mL) was added and the mixture stirred for 2 min (all LiBr dissolved). The solution of A was slowly added and the resulting mixture was stirred at room temperature for 16 h. The solvent was carefully evaporated (ca. 100 mbar, 25 °C, *trans*-bromo acid is volatile), and the crude material transferred to a separatory funnel using Et_2O, followed by the addition of 1M HCl. The two phases were separated and the organic phase was again washed with brine. The organic phase was dried over $MgSO_4$, filtered and the solvent removed under careful reduced pressure. The crude material was purified using column chromatography (pentane/Et_2O; pure pentane to 7:3, any use of HOAc for column chromatography leads to decomposition products) to obtain *trans*-bromo cyclobutene acid (813 mg, 4.59 mmol, 31%) as yellowish oil (little UV-active, eluting first) and *cis*- bromo cyclobutene acid (518 mg, 2.93 mmol, 20%) as a white solid (almost not UV-active, eluting second) in one of the runs.

However, the ratio and yield of this reaction may slightly differ for each reaction run, most likely depending on the remaining water content in the reaction.

***Trans*-4-bromocyclobut-2-ene-1-carboxylic acid (S1)**

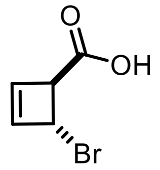

(volatile)

^{1}H NMR (400 MHz, CDCl$_3$): δ 11.05 (s, 1H), 6.38 – 6.34 (m, 1H), 6.29 – 6.24 (m, 1H), 5.10 – 5.05 (m, 1H), 3.94 – 3.88 (m, 1H) ppm.

^{13}C NMR (101 MHz, CDCl$_3$): δ 175.8, 142.1, 135.5, 57.5, 44.4 ppm.

NMR spectra matched those reported in the literature.[1]

***Cis*-4-bromocyclobut-2-ene-1-carboxylic acid (S2)**

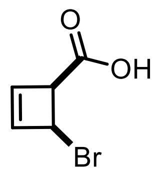

(with characteristic unpleasant odor)

^{1}H NMR (600 MHz, CDCl$_3$) δ 10.40 (bs, 1H), 6.34 (d, *J* = 2.7 Hz, 1H), 6.27 – 6.26 (m, 1H), 5.22 (dd, *J* = 4.3, 0.8 Hz, 1H), 4.16 (d, *J* = 4.1 Hz, 1H) ppm.

^{13}C NMR (151 MHz, CDCl$_3$) δ 175.9, 141.5, 135.8, 53.6, 45.31 ppm.

IR (neat) ν_{max}: 2922, 1702, 1416, 1276, 1223, 1178, 1135, 939, 893, 858.

HRMS (ESI) m/z: [M-H]$^-$ Calculated for $C_5H_4BrO_2^-$ 174.9400; Found 174.9401.

3.2. General Procedures

3.2.1. General Procedure 1 (GP1): Synthesis of Esters via in-situ formed Carbonyl Chlorides

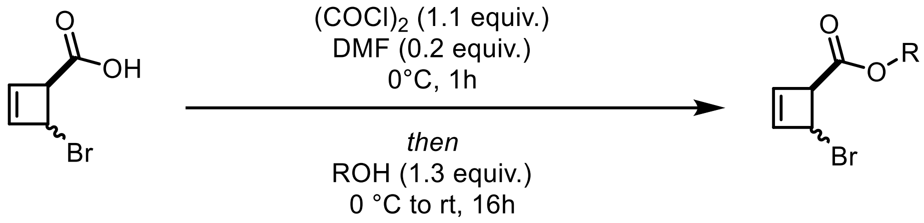

A flame-dried Schlenk flask was loaded with acid (1.0 equiv.) and subsequently DCM (0.2M) and DMF (20mol%) were added. The solution was cooled to 0°C and $(COCl)_2$ (1.1 equiv.) was added dropwise. The mixture was stirred for 1 h at 0 °C. ROH (1.3 equiv.) was added carefully and the mixture was allowed up to room temperature while stirring it for 16 h. The solvent was removed under reduced pressure and the crude material purified using column chromatography (heptane/EtOAc) to give the desired esters.

- ➔ Remark: Little to no epimerization was observed with this method.

3.2.2. General Procedure 2 (GP2): Synthesis of Esters using T3P

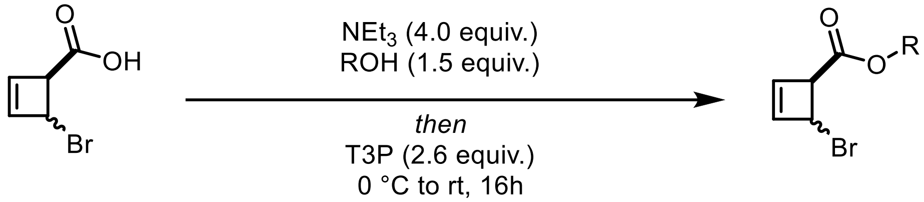

A flame-dried Schlenk flask was loaded with acid (1.0 equiv.) and dissolved in DCM (0.2M). Then, triethylamine (4.0 equiv.) and the corresponding alcohol (1.5 equiv.) were added. The mixture was cooled to 0°C and propylphosphonic anhydride (T3P, 2.6 equiv., in 50% DCM) was added carefully. The mixture was stirred for 1 h at 0 °C. ROH was added and the mixture was allowed up to room temperature while stirring it for 16 h. The solvent was removed under reduced pressure and the crude material purified using column chromatography (heptane/EtOAc) to give the desired esters.

- ➔ Remark: A strong tendency of epimerization was observed in this method

3.2.2. General Procedure 3 (GP3): Synthesis of thioesters via Steglich coupling

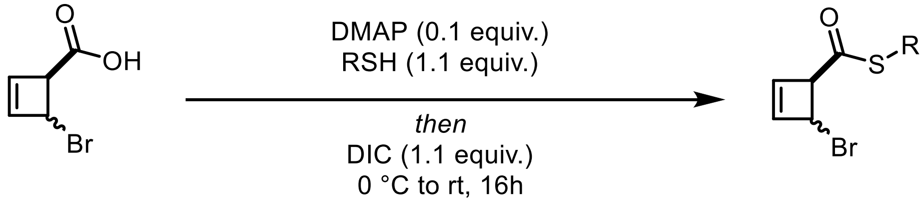

A flame-dried Schlenk flask was loaded with the carboxylic acid (1.0 equiv.) and dissolved in DCM (0.2M). Then, DMAP (0.1 equiv.) and the corresponding thiol (1.1 equiv.) were added. The mixture was cooled to 0°C and N,N'-diisopropylcarbodiimide (DIC, 1.1 equiv.) was added dropwise. The mixture was stirred for 5 min at 0 °C before removing the ice-bath and leaving it warm up at room temperature (23 °C) for 16 h. The solvent was removed under reduced pressure and the crude material purified using column chromatography (heptane/EtOAc) to give the desired esters.

3.2.3. General Procedure 4 (GP4): Dimerization of 4-Bromocyclobutenes

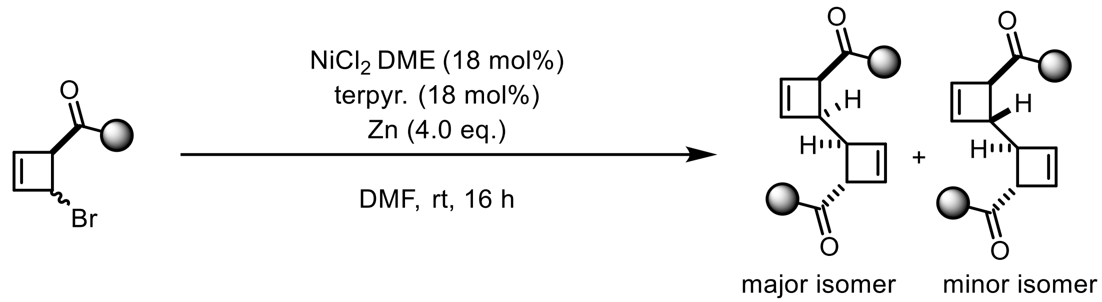

[The reaction described was performed outside the glovebox, but certain chemicals (marked with an asterisk) were stored inside the glovebox and removed before use.]*

A flame-dried Schlenk flask under Argon was loaded with nickel(II) chloride ethylene glycol dimethyl ether complex* (4.0 mg, 0.018 mmol, 0.18 equiv.), terpyridine (4.2 mg, 0.018 mmol, 0.18 equiv.), and zinc* (26.2 mg, 0.4 mmol, 4.0 equiv.). DMF (0.8 mL, 0.125M) was added and the mixture was stirred for 5 min before lastly the corresponding cyclobutene substrate (0.1 mmol, 1.0 equiv.) was added. The reaction was stirred for 16 h. The crude material was transferred to a separatory funnel using Et_2O and the organic phase was washed with water/brine mixture (1:1, 5x). Subsequently, the organic phase was dried over anhydrous $MgSO_4$, and the solvent removed under reduced pressure. The crude material was purified by column chromatography (SiO_2, heptanes/EtOAc).

<u>Used chemicals and their suppliers:</u>

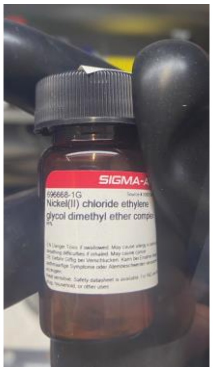

$NiCl_2$·DME
(supplier: Sigma)
stored inside GB

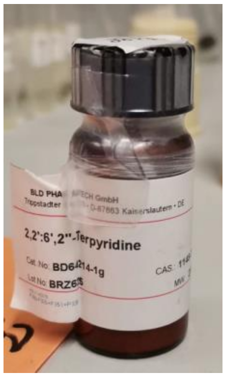

terpyridine
(supplier: BLD)

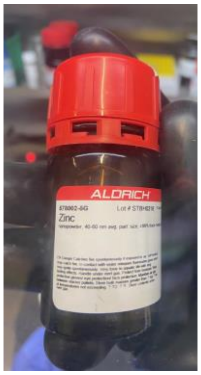

Zn nanopowder
(suppliere: Sigma)
stored inside GB

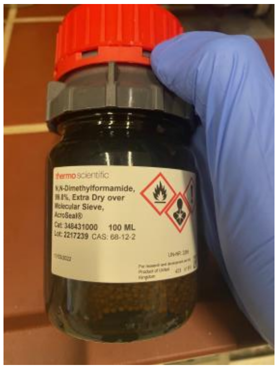

DMF (dry)
(suppliere: termo scientific)

3.3. Characterization of Starting Materials

Benzyl-4-bromocyclobut-2-ene-1-carboxylate (1a)

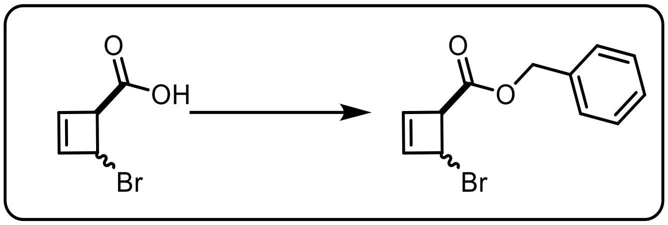

Following GP1 (8.6 mmol scale, cis/trans mixture (2:1), the desired benzyl esters were obtained as yellow liquids (for trans: 800 mg, 2.99 mmol, 35%; for cis: 962 mg, 3.6 mmol, 42%).

***Trans*:**

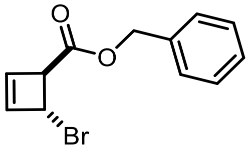

^{1}H NMR (400 MHz, $CDCl_3$): δ 7.39 – 7.34 (m, 5H), 6.33 (s, 1H), 6.26 (s, 1H), 5.17 (s, 2H), 5.09 (s, 1H), 3.91 (s, 1H) ppm.

^{13}C NMR (101 MHz, $CDCl_3$): δ 170.2, 141.8, 135.9, 135.6, 128.8, 128.6, 128.4, 67.0, 58.0, 44.9 ppm.

NMR spectra matched those reported in the literature.[1]

***Cis*:**

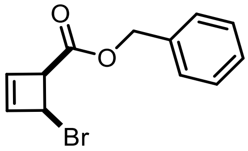

^{1}H NMR (400 MHz, $CDCl_3$): δ 7.42 – 7.36 (m, 5H), 6.32 (d, *J* = 2.7 Hz, 1H), 6.28 – 6.25 (m, 1H), 5.22 – 5.19 (m, 3H), 4.15 (d, *J* = 4.2 Hz, 1H) ppm.

^{13}C NMR (101 MHz, $CDCl_3$): δ 170.2, 141.8, 135.9, 135.6, 128.8, 128.6, 128.4, 67.0, 58.0, 44.9 ppm.

IR (neat) ν_{max}: 1734, 1709, 1620, 1563, 1498, 1455, 1413, 1263, 1241, 1184, 1159, 1080, 1023, 998, 950, 831.

HRMS (ESI) m/z: $[M+Na]^+$ Calculated for $C_{12}H_{11}O_2BrNa^+$288.9840; Found 288.9835.

***Cis*-4-methoxybenzyl-4-bromocyclobut-2-ene-1-carboxylate (1b)**

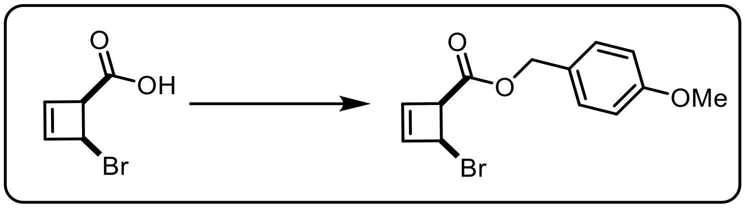

Following GP1 (1.0 mmol scale, pure trans), the desired benzyl ester was obtained as yellow liquid (114 mg, 0.38 mmol, 38%) and single diastereoisomer.

^{1}H NMR (400 MHz, $CDCl_3$): δ 7.37 – 7.33 (m, 2H), 6.91 – 6.87 (m, 2H), 6.30 (d, *J* = 2.5 Hz, 1H), 6.27 – 6.24 (m, 1H), 5.20 – 5.17 (m, 1H), 5.14 (s, 2H), 4.11 (dd, *J* = 4.2, 0.5 Hz, 1H), 3.81 (s, 3H) ppm.

^{13}C NMR (101 MHz, $CDCl_3$): δ 170.1, 159.9, 141.1, 136.3, 130.7, 127.8, 114.0, 67.0, 55.4, 53.8, 46.2 ppm.

IR (neat) ν_{max}: 1729, 1513, 1245, 1171, 1158, 1033, 822, 754.

HRMS (ESI) m/z: $[M+Na]^+$ Calculated for $C_{13}H_{13}O_3BrNa^+$ 318.9946; Found 318.9942.

***Trans*-3,4-dichlorobenzyl-4-bromocyclobut-2-ene-1-carboxylate (1c)**

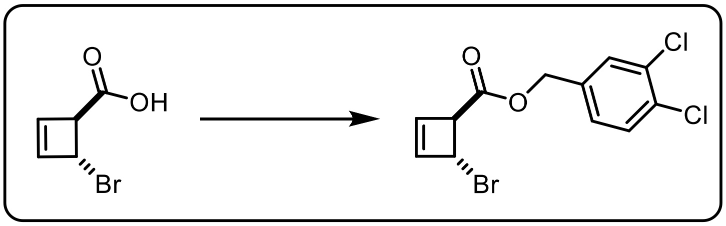

Following GP2 (2.9 mmol, *trans* diastereomer), the titled compound was obtained as a light-yellow oil (299 mg, 0.89 mmol, 30%) and single diastereosisomer.

^{1}H NMR (600 MHz, $CDCl_3$): δ 7.46 – 7.43 (m, 2H), 7.19 (dd, *J* = 8.2, 1.9 Hz, 1H), 6.35 – 6.33 (m, 1H), 6.26 – 6.24 (m, 1H), 5.10 (s, 2H), 5.07 (d, *J* = 0.6 Hz, 1H), 3.91 (d, *J* = 1.1 Hz, 1H) ppm.

^{13}C NMR (151 MHz, $CDCl_3$): δ 169.9, 142.0, 135.7, 135.6, 133.0, 132.8, 130.8, 130.3, 127.6, 65.4, 57.7, 44.7 ppm.

IR (neat) ν_{max}: 1732, 1627, 1563, 1474, 1400, 1364, 1326, 1277, 1248, 1211, 1179, 1159, 1124, 1031, 987, 946.

HRMS (ESI) m/z: $[M+Na]^+$ Calculated for $C_{12}H_9BrCl_2NaO_2^+$ 356.9055; Found 356.9055.

***Trans*-2,4,6-trimethylbenzyl-4-bromocyclobut-2-ene-1-carboxylate (1d)**

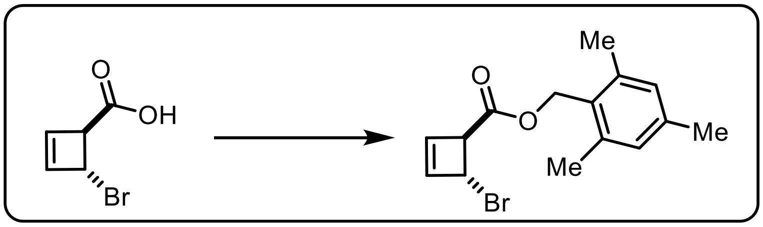

Following GP1 (1.0 mmol scale, pure trans), the desired benzyl ester was obtained as yellow liquid (66 mg, 0.21 mmol, 21%) and single diastereoisomer.

^{1}H NMR (400 MHz, $CDCl_3$): δ 6.89 (s, 2H), 6.33 – 6.29 (m, 1H), 6.25 – 6.19 (m, 1H), 5.22 (s, 2H), 5.06 (s, 1H), 3.86 (s, 1H), 2.35 (s, 6H), 2.28 (s, 3H) ppm.

^{13}C NMR (151 MHz, $CDCl_3$): δ 170.5, 141.7, 138.9, 138.4, 136.0, 129.3, 128.7, 62.0, 58.0, 44.9, 21.2, 19.7 ppm.

IR (neat) ν_{max}: 1728, 1178, 1159, 1137, 850, 750.

HRMS (ESI) m/z: $[M+Na]^+$ Calculated for $C_{15}H_{17}O_2BrNa^+$ 331.0310; Found 331.0301.

***Trans*-furan-2-ylmethyl-4-bromocyclobut-2-ene-1-carboxylate (1e)**

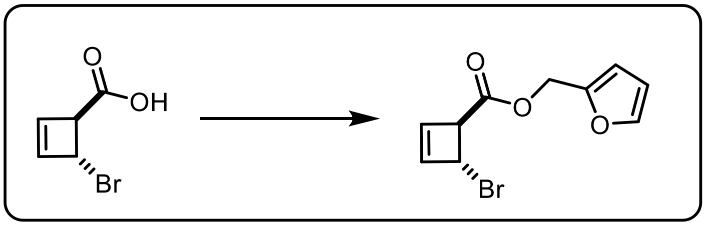

Following GP1 (1.0 mmol scale, pure trans acid as starting material), the desired ester was obtained as yellow liquid (80 mg, 0.31 mmol, 31%).

^{1}H NMR (400 MHz, $CDCl_3$): δ 7.45 – 7.41 (m, 1H), 6.43 (app d, *J* = 3.1 Hz, 1H), 6.39 – 6.36 (m, 1H), 6.33 – 6.30 (m, 1H), 6.26 – 6.22 (m, 1H), 5.11 (s, 2H), 5.07 (s, 1H), 3.90 – 3.86 (m, 1H) ppm.

^{13}C NMR (101 MHz, $CDCl_3$): δ 170.0, 149.1, 143.6, 141.8, 135.9, 111.2, 110.8, 58.8, 57.8, 44.8 ppm.

IR (neat) ν_{max}: 1734, 1629, 1502, 1370, 1326, 1277, 1249, 1181, 1162, 1122, 1080, 1016, 991, 945, 918.

HRMS (ESI) m/z: $[M+Na]^+$ Calculated for $C_{10}H_9BrNaO_3^+$ 278.9627; Found 278.9624.

Tert-butyl-4-bromocyclobut-2-ene-1-carboxylate (1f)

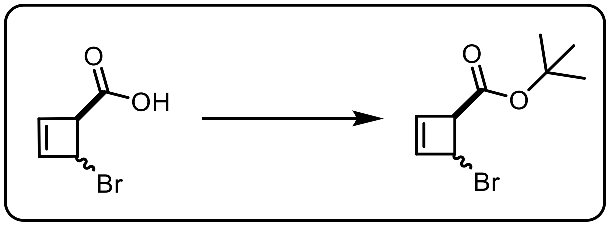

Following GP2, starting from the corresponding bromoacid (1.25 mmol, cis/trans mixture (1:9)), the desired phenyl esters were obtained as clear oils (for trans: 106 mg, 0.41 mmol, 33%; for cis: 65 mg, 0.25 mmol, 20%). It was not possible to fully characterize the *cis* diastereomer after isolation due to rapid decomposition.

Trans:

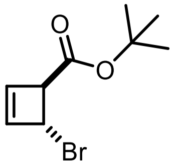

^{1}H NMR (400 MHz, CDCl$_3$) δ 6.32 – 6.30 (m, 1H), 6.25 – 6.22 (m, 1H), 5.03 – 5.02 (m, 1H), 3.78 – 3.77 (m, 1H), 1.47 (s, 9H) ppm.

^{13}C NMR (101 MHz, CDCl$_3$): δ 169.7, 141.4, 136.5, 81.8, 59.2, 45.3, 28.2 ppm.

IR (neat) ν_{max}: 2957, 1730, 1461, 1369, 1337, 1277, 1260, 1155, 1084, 1015.

HRMS (ESI) m/z: $[M+Na]^+$ Calculated for $C_9H_{13}BrNaO_2^+$ 254.9991; Found 254.9987.

2-(trimethylsilyl)ethyl-4-bromocyclobut-2-ene-1-carboxylate (1g)

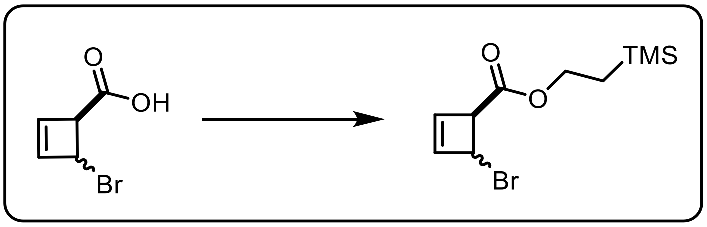

Following GP2, starting from the corresponding bromoacid (1.21 mmol, cis/trans mixture (1:9)), the desired phenyl esters were obtained as clear oils (for trans: 106 mg, 0.38 mmol, 31%; for cis: 57 mg, 0-21 mmol, 17%).

Trans:

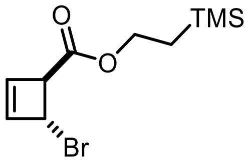

^{1}H NMR (600 MHz, CDCl$_3$): δ 6.33 – 6.31 (m, 1H), 6.26 – 6.24 (m, 1H), 5.06 (s, 1H), 4.23 – 4.20 (m, 2H), 3.84 (d, *J* = 1.1 Hz, 1H), 1.03 – 0.99 (m, 2H), 0.05 (s, 9H) ppm.

^{13}C NMR (151 MHz, CDCl$_3$): δ 170.5, 141.6, 136.1, 63.7, 58.2, 45.0, 17.6, -1.3 ppm.

IR (neat) ν_{max}: 2953, 2898, 1730, 1631, 1412, 1380, 1326, 1310, 1278, 1217, 1177, 1123, 1063, 989, 938.

HRMS (ESI) m/z: $[M+Na]^+$ Calculated for $C_{10}H_{17}BrNaO_2Si^+$ 299.0073; Found 299.0073.

***Cis*:**

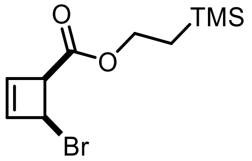

^{1}H NMR (600 MHz, CDCl$_3$) δ 6.30 (d, *J* = 2.6 Hz, 1H), 6.25 – 6.24 (m, 1H), 5.18 (d, *J* = 4.2 Hz, 1H), 4.26 (td, *J* = 9.0, 1.9 Hz, 2H), 4.06 (d, *J* = 4.2 Hz, 1H), 1.05 (ddd, *J* = 10.5, 6.9, 2.5 Hz, 2H), 0.04 (s, 9H) ppm.

^{13}C NMR (151 MHz, CDCl$_3$) δ 170.1, 140.7, 136.2, 63.4, 53.7, 46.1, 17.4, -1.5 ppm.

IR (neat) ν_{max}: 2954, 1731, 1712, 1630, 1379, 1327, 1309, 1278, 1249, 1217, 1177, 1161, 1124, 1064.

HRMS (ESI) m/z: $[M+Na]^+$ Calculated for $C_{10}H_{17}BrNaO_2Si^+$ 299.0073; Found 299.0071.

Methyl-4-bromocyclobut-2-ene-1-carboxylate (1h)

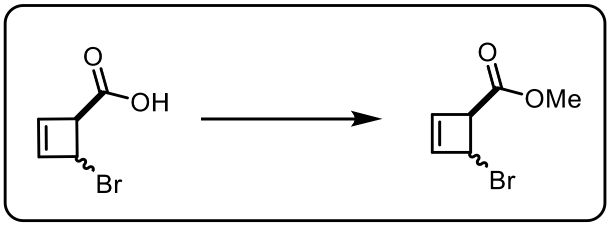

Following GP1, starting from the corresponding bromoacid (1.21 mmol, cis/trans mixture (1:1)), the desired methyl esters were obtained as off-yellow oils (for trans: 193 mg, 1.01 mmol, 34%; the cis isomer co-eluted with an oxalate-derived impurity, and it was not possible to isolate pure).

Trans:

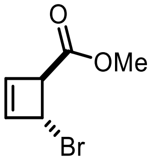

^{1}H NMR (400 MHz, CDCl3) δ 6.33 – 6.31 (m, 1H), 6.26 – 6.23 (m, 1H), 5.06 (s, 1H), 3.87 – 3.86 (m, 1H), 3.73 (s, 3H).

NMR spectra matched those reported in the literature.[1]

Hexyl-4-bromocyclobut-2-ene-1-carboxylate (1i)

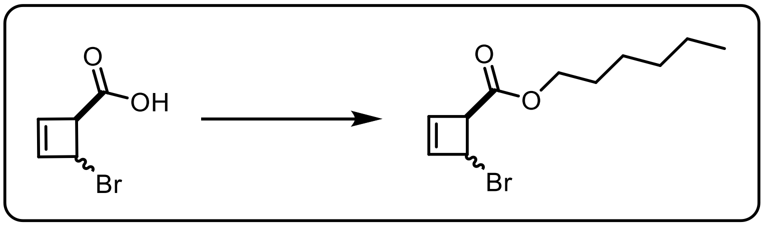

Following GP2 (1.25 mmol, cis/trans mixture (1:9)), the desired phenyl esters were obtained as clear oils (for trans: 106 mg, 0.41 mmol, 33%; for cis: 65 mg, 0.25 mmol, 20%).

Trans:

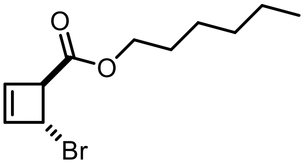

^{1}H NMR (600 MHz, $CDCl_3$) δ 6.33 – 6.31 (m, 1H), 6.25 – 6.24 (m, 1H), 5.07 – 5.06 (m, 1H), 4.11 (t, *J* = 6.8 Hz, 2H), 3.86 – 3.85 (m, 1H), 1.66 – 1.62 (m, 2H), 1.36 – 1.29 (m, 6H), 0.89 (t, *J* = 6.9 Hz, 3H) ppm.

^{13}C NMR (151 MHz, $CDCl_3$) δ 170.3, 141.5, 135.9, 65.3, 57.9, 44.9, 31.4, 28.5, 25.5, 22.5, 14.0 ppm.

IR (neat) ν_{max}: 2959, 2930, 2858, 1730, 1630, 1468, 1330, 1264, 1180, 1165, 1125, 1020.

HRMS (ESI) m/z: $[M+H]^+$ Calculated for $C_{11}H_{18}BrO_2^+$ 261.0485; Found 261.0483.

***Cis*:**

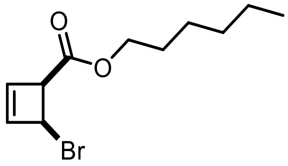

^{1}H NMR (400 MHz, $CDCl_3$): δ 6.31 (d, *J* = 2.6 Hz, 1H), 6.27 – 6.25 (m, 1H), 5.19 (d, *J* = 4.1 Hz, 1H), 4.17 (td, *J* = 6.7, 1.0 Hz, 2H), 4.09 (d, *J* = 4.2 Hz, 1H), 1.73 – 1.63 (m, 2H), 1.43 – 1.25 (m, 6H), 0.89 (t, *J* = 6.9 Hz, 3H) ppm.

^{13}C NMR (101 MHz, $CDCl_3$): δ 170.1, 140.8, 136.2, 65.3, 53.7, 46.1, 31.4, 28.6, 25.6, 22.5, 14.0 ppm.

IR (neat) ν_{max}: 2957, 2930, 2857, 1731, 1467, 1333, 1286, 1266, 1204, 1187, 1167, 1137, 1113, 1021.

HRMS (ESI) m/z: $[M+H]^+$ Calculated for $C_{11}H_{18}BrO_2^+$ 261.0485; Found 261.261.0480.

Cyclopentyl-4-bromocyclobut-2-ene-1-carboxylate (1j)

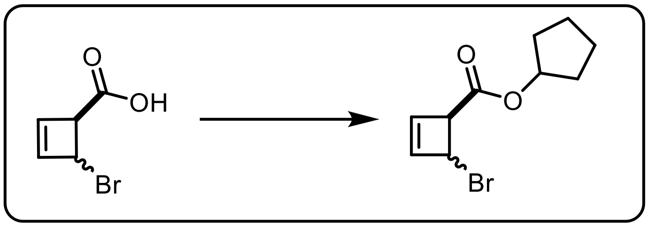

Following GP2 (0.49 mmol, cis/trans mixture (3:1)), the desired cyclopentyl esters were obtained as clear oils (for trans: 36 mg, 0.15 mmol, 30%; for cis: 44 mg, 0.18 mmol, 37%).

Trans:

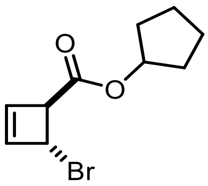

^{1}H NMR (600 MHz, $CDCl_3$): δ 6.32 – 6.31 (m, 1H), 6.24 – 6.23 (m, 1H), 5.19 (dt, *J* = 9.1, 3.2 Hz, 1H), 5.05 (s, 1H), 3.82 (s, 1H), 1.90 – 1.84 (m, 2H), 1.75 – 1.67 (m, 4H), 1.62 – 1.58 (m, 2H) ppm.

^{13}C NMR (151 MHz, $CDCl_3$): δ 170.1, 141.6, 136.2, 78.1, 58.3, 45.1, 32.8, 32.8, 23.9 ppm.

IR (neat) ν_{max}: 2960, 1730, 1330, 1278, 1262, 1197, 1163, 1035, 990, 950, 860.

HRMS (ESI) m/z: $[M+Na]^+$ Calculated for $C_{10}H_{13}BrNaO_2^+$ 266.9991; Found 266.9992.

***Cis*:**

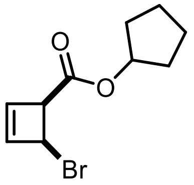

^{1}H NMR (600 MHz, CDCl$_3$) δ 6.29 (d, *J* = 2.6 Hz, 1H), 6.24 – 6.23 (m, 1H), 5.28 – 5.25 (m, 1H), 5.19 – 5.17 (m, 1H), 4.03 (dd, *J* = 4.2, 0.6 Hz, 1H), 1.88 – 1.84 (m, 2H), 1.80 – 1.72 (m, 4H), 1.62 – 1.57 (m, 2H) ppm.

^{13}C NMR (151 MHz, CDCl$_3$) δ 167.0, 140.8, 136.4, 78.3, 53.7, 46.4, 32.8, 23.8, 23.8 ppm.

IR (neat) ν_{max}: 2967, 2873, 1724, 1436, 1366, 1334, 1287, 1266, 1204, 1193, 1160, 1138, 1116, 1036, 995, 961.

HRMS (ESI) m/z: $[M+Na]^+$ Calculated for $C_{10}H_{13}BrNaO_2^+$ 266.9991; Found 266.9989.

Pent-4-en-1-yl-4-bromocyclobut-2-ene-1-carboxylate (1k)

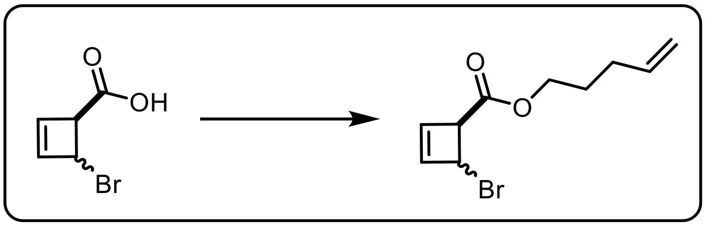

Following GP1 (1.94 mmol scale, cis/trans mixture 1.4:1), the desired alkyl esters were obtained as yellow liquids (for trans: 140 mg, 0.57 mmol, 29%; for cis: 133 mg, 0.54 mmol, 28%).

***Trans*:**

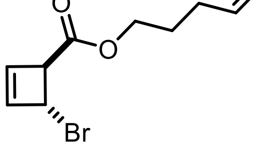

^{1}H NMR (400 MHz, CDCl$_3$): δ 6.35 – 6.31 (m, 1H), 6.26 – 6.23 (m, *J* = 2.5, 1.2 Hz, 1H), 5.84 – 5.76 (m, 1H), 5.06 (s, 1H), 5.04 – 4.97 (m, 2H), 4.15 – 4.12 (m, 2H), 3.86 (d, *J* = 1.2 Hz, 1H), 2.15 – 2.10 (m, 2H), 1.78 – 1.73 (m, 2H) ppm.

^{13}C NMR (101 MHz, CDCl$_3$): δ 170.4, 141.7, 137.4, 136.0, 115.6, 64.7, 58.0, 45.0, 30.1, 27.8 ppm.

IR (neat) ν_{max}: 1732, 1712, 1327, 1277, 1177, 1164, 1124, 990, 913, 753.

HRMS (ESI) m/z: $[M+Na]^+$ Calculated for $C_{10}H_{13}BrNaO_2^+$ 266.9991; Found 266.9991.

Cis:

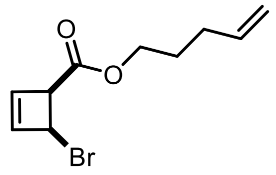

Na^+

^{1}H NMR (400 MHz, CDCl$_3$): δ 6.31 (d, *J* = 2.6 Hz, 1H), 6.27 – 6.24 (m, 1H), 5.81 (ddt, *J* = 16.9, 10.2, 6.6 Hz, 1H), 5.20 (d, *J* = 4.2 Hz, 1H), 5.08 – 5.02 (m, 1H), 5.01 – 4.97 (m, 1H), 4.19 (td, *J* = 6.6, 1.5 Hz, 2H), 4.09 (d, *J* = 4.2 Hz, 1H), 2.20 – 2.13 (m, 2H), 1.83 – 1.75 (m, 2H) ppm.

^{13}C NMR (101 MHz, CDCl$_3$): δ 170.2, 141.0, 137.6, 136.3, 115.5, 64.7, 53.9, 46.2, 30.2, 28.0 ppm.

IR (neat) ν_{max}: 1730, 1332, 1286, 1264, 1204, 1187, 1166, 913, 758.

HRMS (ESI) m/z: $[M+Na]^+$ Calculated for $C_{10}H_{13}BrNaO_2^+$ 266.9991; Found 266.9991.

Allyl-4-bromocyclobut-2-ene-1-carboxylate (1l)

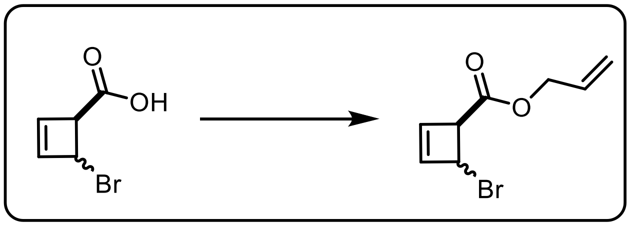

Following GP1 (1.0 mmol scale, trans/cis mixture 1.3:1) the desired allylic esters were obtained as yellow liquids (for trans: 79.1 mg, 0.36 mmol, 36%; for cis: 34.2 mg, 0.16 mmol, 16%).

Trans:

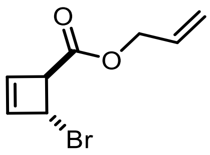

^{1}H NMR (400 MHz, CDCl$_3$): δ 6.35 – 6.32 (m, *J* = 2.1, 1.3 Hz, 1H), 6.27 – 6.25 (m, *J* = 2.4, 1.1 Hz, 1H), 5.97 – 5.87 (m, 1H), 5.37 – 5.30 (m, *J* = 17.2, 2.8, 1.4 Hz, 1H), 5.28 – 5.24 (m, 1H), 5.09 – 5.07 (m, *J* = 0.7 Hz, 1H), 4.63 (dt, *J* = 5.8, 1.3 Hz, 2H), 3.90 – 3.88 (m, 1H) ppm.

^{13}C NMR (101 MHz, CDCl$_3$): δ 170.0, 141.8, 135.9, 131.8, 118.9, 65.9, 57.9, 44.9 ppm.

IR (neat) ν_{max}: 1735, 1180, 904, 760, 731, 650.

HRMS (ESI) m/z: $[M+Na]^+$ Calculated for $C_8H_9BrNaO_2^+$ 238.9684; Found 238.9676.

Cis:

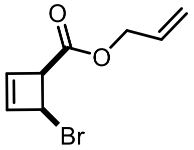

^{1}H NMR (400 MHz, CDCl$_3$): δ 6.32 (d, *J* = 2.6 Hz, 1H), 6.28 – 6.26 (m, 1H), 6.02 – 5.92 (m, 1H), 5.40 – 5.34 (m, 1H), 5.29 – 5.25 (m, 1H), 5.21 – 5.19 (m, 1H), 4.68 (dt, *J* = 5.9, 1.3 Hz, 2H), 4.13 (d, *J* = 4.2 Hz, 1H) ppm.

^{13}C NMR (101 MHz, CDCl$_3$): δ 169.9, 141.1, 136.2, 132.2, 118.9, 66.0, 53.8, 46.1 ppm.

IR (neat) ν_{max}: 1732, 1203, 1164, 990, 933, 759.

HRMS (ESI) m/z: $[M+Na]^+$ Calculated for $C_8H_9BrNaO_2^+$ 238.9684; Found 238.9676.

But-2-yn-1-yl-4-bromocyclobut-2-ene-1-carboxylate (1m)

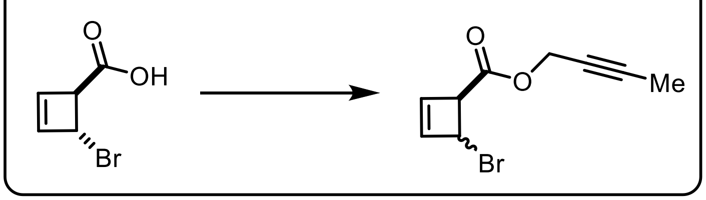

Following GP2 (0.6 mmol scale, pure cis bromo acid as starting material), the desired benzyl esters were obtained as yellow liquids (for trans: 26.5 mg, 0.12 mmol, 19%; for cis: 31.6 mg, 0.14 mmol, 23%).

Trans:

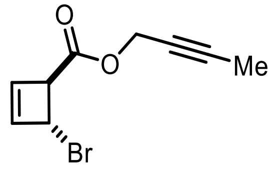

^{1}H NMR (400 MHz, CDCl$_3$): δ 6.34 – 6.31 (m, 1H), 6.26 (dt, *J* = 2.5, 1.2 Hz, 1H), 5.09 (d, *J* = 0.7 Hz, 1H), 4.69 (q, *J* = 2.4 Hz, 2H), 3.90 (dd, *J* = 2.4, 1.2 Hz, 1H), 1.86 (t, *J* = 2.4 Hz, 3H) ppm.

^{13}C NMR (101 MHz, CDCl$_3$): δ 169.8, 141.8, 135.8, 83.9, 72.8, 57.7, 53.6, 44.7, 3.8 ppm.

IR (neat) ν_{max}: 1738, 1325, 1277, 1250, 1181, 1160, 990, 757.

HRMS (ESI) m/z: [M+Na]$^+$ Calculated for $C_9H_9O_2BrNa^+$ 250.9684; Found 250.9678.

Cis:

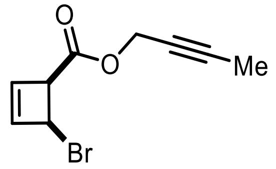

^{1}H NMR (400 MHz, CDCl$_3$): δ 6.34 (d, *J* = 2.7 Hz, 1H), 6.30 – 6.28 (m, 1H), 5.23 (d, *J* = 4.2 Hz, 1H), 4.90 – 4.64 (m, 2H), 4.17 (d, *J* = 4.2 Hz, 1H), 1.89 (t, *J* = 2.4 Hz, 3H) ppm.

^{13}C NMR (101 MHz, CDCl$_3$): δ 169.6, 141.2, 136.0, 83.8, 73.0, 53.6, 46.0, 3.9 ppm.

IR (neat) ν_{max}: 1734, 1183, 1157, 1135, 1111, 988, 757.

HRMS (ESI) m/z: [M+Na]$^+$ Calculated for $C_9H_9O_2BrNa^+$ 250.9684; Found 250.9677.

***Trans*-phenyl-4-bromocyclobut-2-ene-1-carboxylate (1n)**

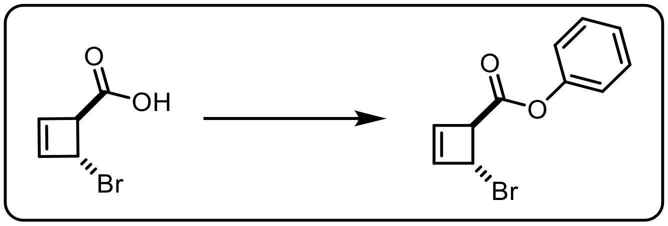

Following GP2 (0.48 mmol, *trans* diastereomer), the titled compound was obtained as a light-yellow oil (77 mg, 304 µmol, 63%).

^{1}H NMR (600 MHz, CDCl$_3$): δ 7.41 – 7.38 (m, 2H), 7.27 – 7.24 (m, 1H), 7.12 – 7.10 (m, 2H), 6.42 – 6.40 (m, 1H), 6.38 – 6.37 (m, 1H), 5.21 (d, *J* = 0.6 Hz, 1H), 4.11 (d, *J* = 1.2 Hz, 1H) ppm.

^{13}C NMR (151 MHz, CDCl$_3$): δ 168.7, 150.5, 142.1, 135.7, 129.7, 126.3, 121.5, 57.9, 44.7 ppm.

IR (neat) ν_{max}: 3068, 1753, 1627, 1592, 1493, 1411, 1333, 1309, 1276, 1235, 1191, 1161, 1110, 1069, 988, 933.

HRMS (ESI) m/z: $[M+Na]^+$ Calculated for $C_{11}H_9BrNaO_2^+$ 274.9678; Found 274.9678.

Ethyl 2-((-4-bromocyclobut-2-ene-1-carbonyl)oxy)benzoate (1o)

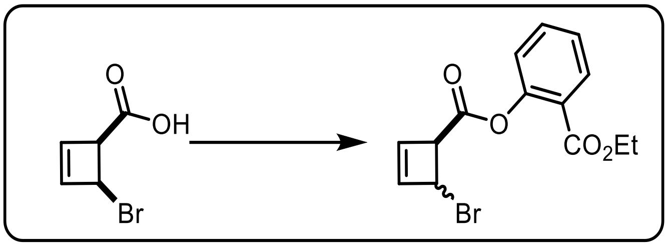

Following GP2 (0.6 mmol scale, pure cis bromo acid as starting material), the desired phenyl esters were obtained as yellow liquids (for trans: 37.1 mg, 0.22 mmol, 37%; for cis: 10.5 mg, 0.06 mmol, 11%).

Trans:

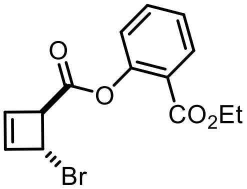

^{1}H NMR (400 MHz, CDCl$_3$): δ 8.03 (dd, *J* = 7.8, 1.7 Hz, 1H), 7.57 (td, *J* = 7.9, 1.7 Hz, 1H), 7.34 (dd, *J* = 7.6, 1.0 Hz, 1H), 7.11 (dd, *J* = 8.1, 1.0 Hz, 1H), 6.46 – 6.39 (m, 2H), 5.35 (s, 1H), 4.33 (q, *J* = 7.1 Hz, 2H), 4.15 (d, *J* = 1.2 Hz, 1H), 1.37 (t, *J* = 7.1 Hz, 3H) ppm.

^{13}C NMR (101 MHz, CDCl$_3$): δ 168.8, 164.3, 150.4, 141.8, 135.8, 133.9, 131.9, 126.4, 123.7, 123.5, 61.3, 57.8, 44.9, 14.4 ppm.

IR (neat) ν_{max}: 1758, 1718, 1294, 1273, 1258, 1200, 1154, 1128, 1080.

HRMS (ESI) m/z: $[M+Na]^+$ Calculated for $C_{14}H_{23}O_4BrNa^+$ 346.9895; Found 346.9889.

***Cis*:**

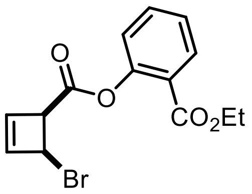

^{1}H NMR (400 MHz, CDCl$_3$): δ 8.03 (dd, *J* = 8.0, 1.5 Hz, 1H), 7.56 (td, *J* = 7.8, 1.6 Hz, 1H), 7.33 (dd, *J* = 11.9, 4.4 Hz, 2H), 6.39 (d, *J* = 0.5 Hz, 2H), 5.34 (d, *J* = 4.2 Hz, 1H), 4.43 (d, *J* = 4.2 Hz, 1H), 4.33 (q, *J* = 7.1 Hz, 2H), 1.37 (t, *J* = 7.1 Hz, 3H) ppm.

^{13}C NMR (101 MHz, CDCl$_3$): δ 168.5, 164.6, 150.7, 141.3, 136.3, 134.0, 131.8, 126.3, 124.4, 123.5, 61.2, 53.6, 46.2, 14.4 ppm.

IR (neat) ν_{max}: 1764, 1720, 1294, 1252, 1146, 1131, 1081.

HRMS (ESI) m/z: $[M+Na]^+$ Calculated for $C_{14}H_{23}O_4BrNa^+$ 346.9895; Found 346.9891.

***S*-Benzyl 4-bromocyclobut-2-ene-1-carbothioate (1p)**

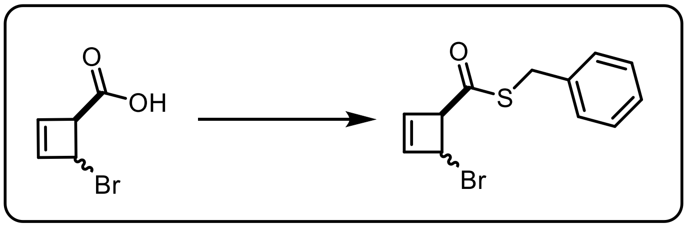

Following GP2, starting from the corresponding bromoacid (1.25 mmol, cis/trans mixture (1:9)), the desired phenyl esters were obtained as clear oils (for trans: 129 mg, 0.46 mmol, 36%; for cis: 20 mg, 0.071 mmol, 6%).

Trans:

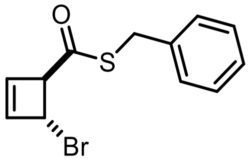

^{1}H NMR (400 MHz, CDCl$_3$) δ 7.35 – 7.27 (m, 5H), 6.41 – 6.39 (m, 1H), 6.31 – 6.29 (m, 1H), 5.06 (s, 1H), 4.18 (s, 2H), 4.05 (d, *J* = 1.0 Hz, 1H) ppm.

^{13}C NMR (151 MHz, CDCl$_3$) δ 195.5, 142.4, 136.9, 136.0, 128.9, 128.7, 127.5, 64.9, 45.1, 33.2 ppm.

IR (neat) ν_{max}: 1679, 1603, 1495, 1453, 1410, 1306, 1272, 1241, 1181, 1132, 1091, 1030, 995, 913, 832, 815.

HRMS (ESI) m/z: $[M+Na]^+$ Calculated for $C_{12}H_{11}BrNaOS^+$ 304.9606; Found 304.9606.

***Cis*:**

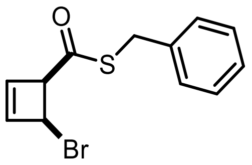

^{1}H NMR (600 MHz, CDCl$_3$) δ 7.34 – 7.28 (m, 4H), 7.26 – 7.22 (m, 1H), 6.41 – 6.40 (m, 1H), 6.29 – 6.27 (m, 1H), 5.19 (d, *J* = 4.1 Hz, 1H), 4.22 (s, 2H), 4.20 (d, *J* = 4.3 Hz, 1H) ppm.

^{13}C NMR (151 MHz, CDCl$_3$) δ 196.1, 142.4, 137.06, 135.5, 129.0, 128.6, 127.3, 59.6, 46.0, 33.4 ppm.

IR (neat) ν_{max}: 1664, 1603, 1494, 1453, 1409, 1271, 1241, 1184, 1127, 1092, 1071, 1028, 980, 931.

HRMS (ESI) m/z: $[M+Na]^+$ Calculated for $C_{12}H_{11}BrNaOS^+$ 304.9606; Found 304.9603.

***S*-(4-methoxybenzyl)-4-bromocyclobut-2-ene-1-carbothioate (1q)**

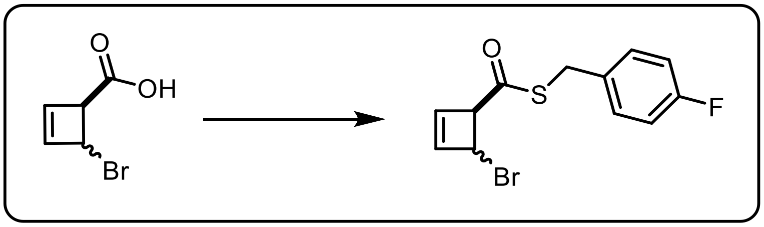

Following GP3, starting from the corresponding bromoacid (1.32 mmol, cis/trans mixture (3:1)), procedure afforded a mixture of the *trans*- and *cis*-diastereomers respectively. It was only possible to isolate the *trans*-diastereomer (217 mg, 0.72 mmol, 55%) as the other one co-eluted with unidentified impurities.

Trans:

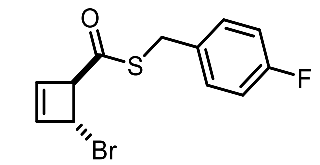

^{1}H NMR (600 MHz, CDCl$_3$) δ 7.27 – 7.24 (m, 2H), 6.99 (t, *J* = 8.6 Hz, 2H), 6.37 (s, 1H), 6.27 (d, *J* = 1.2 Hz, 1H), 5.03 (s, 1H), 4.12 (s, 2H), 4.02 (s, 1H) ppm.

^{13}C NMR (151 MHz, CDCl$_3$) δ 195.39, 162.09 (d, *J* = 246.3 Hz), 142.10, 135.91, 132.78 (d, *J* = 3.2 Hz), 130.49 (d, *J* = 7.8 Hz), 115.58 (d, *J* = 21.7 Hz), 64.88, 45.04, 32.49 ppm.

^{19}F NMR (565 MHz, CDCl$_3$) δ -114.69 ppm.

IR (neat) ν_{max}: 1681, 1661, 1603, 1508, 1417, 1273, 1221, 1182, 1157, 1133, 1091, 1032, 996.

HRMS (ESI) m/z: $[M+Na]^+$ Calculated for $C_{12}H_{10}BrFNaOS^+$ 322.9512; Found 322.9507.

***S*-(4-Methoxybenzyl)-4-bromocyclobut-2-ene-1-carbothioate (1r)**

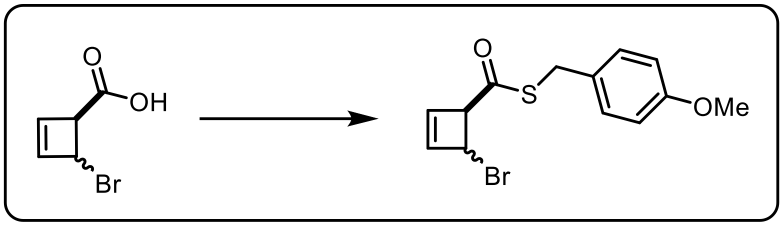

Following GP3, starting from the corresponding bromoacid (1.54 mmol, cis/trans mixture (3:1)), the procedure afforded a mixture of the *trans*- and *cis*-diastereomers respectively. It was only possible to isolate the *trans*-diastereomer (210 mg, 0.67 mmol, 44%), as the other one co-eluted with unidentified impurities.

Trans:

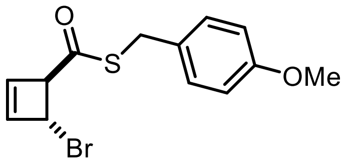

^{1}H NMR (400 MHz, CDCl$_3$): δ 7.23 (d, *J* = 8.6 Hz, 2H), 6.86 (d, *J* = 8.6 Hz, 2H), 6.40 – 6.38 (m, 1H), 6.30 – 6.28 (m, 1H), 5.06 (s, 1H), 4.14 (s, 2H), 4.04 (s, 1H), 3.81 (s, 3H) ppm.

^{13}C NMR (151 MHz, CDCl$_3$): δ 195.9, 159.1, 142.2, 136.2, 130.2, 129.0, 114.3, 65.1, 55.4, 45.3, 33.0 ppm.

IR (neat) ν_{max}: 2834, 1680, 1662, 1607, 1583, 1511, 1463, 1440, 1301, 1250, 1238, 1175, 1130, 1091, 1030, 995, 933.

HRMS (ESI) m/z: $[M+Na]^+$ Calculated for $C_{13}H_{13}BrNaO_2S^+$ 334.9712; Found 334.9712.

***Trans*- (((-4-bromocyclobut-2-en-1-yl)methoxy)methyl)benzene (7)**

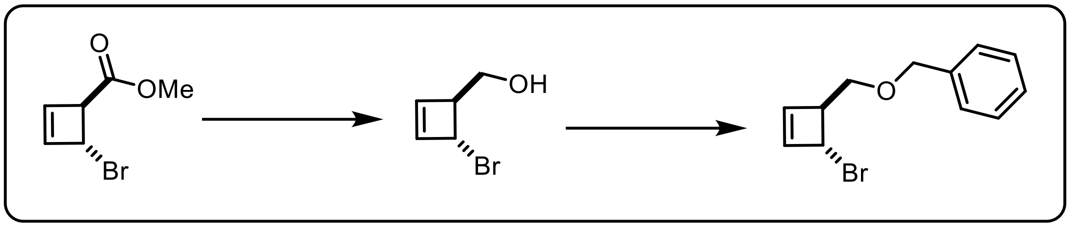

A flame-dried finger Schlenk equipped with a stirring bar and filled with argon was cooled down to -78°C and charged with DIBAL-H (1 M in toluene, 1.31 mL, 1.31 mmol, 2.5 equiv.). Methyl ester **1g** (100 mg, 0.52 mmol, 1.0 equiv.) was parallelly dissolved in dry toluene (2 mL, 0.26M) and added drop-wise to the cold DIBAL-H solution, under stirring. The resulting reaction mixture was left stirring at -78°C for 1 h. At this point the reaction was quenched with saturated aqueous solution of Rochelle salt, and was left stirring at 23°C for 5 min. The crude was then extracted with DCM. The combined organic phases were washed with brine, dried on $MgSO_4$, filtered and concentrated under reduced pressure. The concentrated crude was purified through silica gel column chromatography (0:1 to 1:1 Et_2O/pentane), affording the desired alcohol (36 mg, 0.22 mmol, 42%) as a yellow oil which was directly used in the next step without further purification due to its potential volatility.

A flame-dried finger Schlenk equipped with a stirring bar and filled with argon was charged with a solution of *trans*-cyclobutene alcohol assumed to be pure (36 mg, 0.22 mmol, 1.0 equiv.). The solution was cooled to 0°C before the addition of NaH (60% oil suspension, 11 mg, 0.26 mmol, 1.2 equiv.). The resulting mixture was left stirring at the same temperature for 1 h. At this point, benzyl bromide (52 µL, 0.44 mmol) was added, and the reaction crude was left to warm up to 23°C and kept under stirring for 18 h. At this point, the reaction was quenched with saturated NH_4Cl solution, and extracted with DCM. The combined organic phases were dried on $MgSO_4$, filtered and concentrated under reduced pressure. The resulting concentrated crude was purified via silica gel column chromatography (0:1 to 1:9 Et_2O:Pentane), affording the desired *trans*-bromobenzyl ether (15 mg, 0.059 mmol, 27%) as a light-yellow oil.

^{1}H NMR (400 MHz, $CDCl_3$) δ 7.38 – 7.29 (m, 5H), 6.26 – 6.24 (m, 1H,), 6.21 – 6.19 (m, 1H), 4.77 (s, 1H), 4.54 (s, 2H), 3.61 (dd, *J* = 10.2, 5.1 Hz, 1H), 3.53 (dd, *J* = 10.2, 5.8 Hz, 1H), 3.39 – 3.35 (m, 1H).

^{13}C NMR (151 MHz, $CDCl_3$) δ 138.9, 138.5, 138.1, 128.4, 127.7, 127.7, 127.6, 73.1, 69.3, 56.7, 46.9 ppm.

IR (neat) ν_{max}: 1454, 1361, 1183, 1101, 1075, 980, 737, 697.

HRMS (ESI) m/z: $[M+Na]^+$ Calculated for $C_{12}H_{13}BrNaO^+$ 275.0042; Found 275.0038.

3.4. Characterization of Cyclobutene Dimers

Note: In the following, all cyclobutene dimers were characterized as a mixture of two diastereoisomers. The two diastereoisomers could not be separated by column chromatography. Signals from the minor isomer are marked with an asterisk (*).

Dibenzyl-[1,1'-bi(cyclobutane)]-3,3'-diene-2,2'-dicarboxylate (2a)

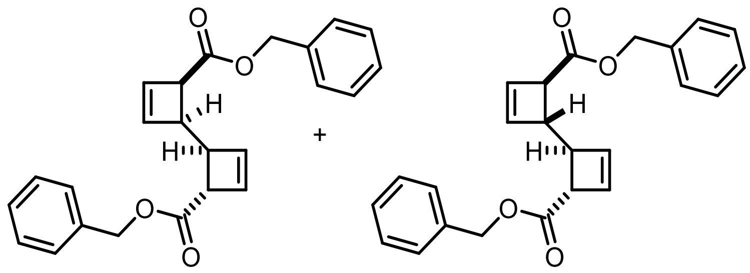

5.1:1

Following GP4 using the ***trans*-1a** (0.1 mmol), the titled compound was obtained in a diastereomeric mixture with a ratio of 5.1:1 as a colorless oil (10.1 mg, 27 µmol, 54%). The same reaction starting from the cis isomer (***cis*-1a**) (0.1 mmol), provided the desired compound in a similar ratio of 4.7:1 and similar yield (11.2 mg, 30 µmol, 60%).

^{1}H NMR (700 MHz, $CDCl_3$): δ 7.36 – 7.31 (m, 10H), 6.27 (d, *J* = 2.5 Hz, 1.6H), 6.25 (d, *J* = 2.5 Hz, 0.4H*), 6.16 (dd, *J* = 2.8, 0.8 Hz, 0.4H*), 6.15 (dd, *J* = 2.8, 0.9 Hz, 1.6H), 5.15 – 5.11 (m, 4H), 3.41 (app s, 0.4H*), 3.36 (app s, 1.6H), 3.25 (app s, 0.4H*), 3.18 (app s, 1.6H) ppm.

^{13}C NMR (176 MHz, $CDCl_3$): δ 172.6*, 172.5, 141.1, 140.3*, 136.1, 136.1*, 135.0*, 134.6, 128.7, 128.3, 128.2*, 128.1, 66.5*, 66.4, 50.0, 49.3*, 49.0, 48.3* ppm.

IR (neat) $ν_{max}$: 1728, 1156, 989, 754, 731, 695.

HRMS (ESI) m/z: $[M+H]^+$ Calculated for $C_{24}H_{23}O_4^+$ 375.1591; Found 375.1586.

Large scale:

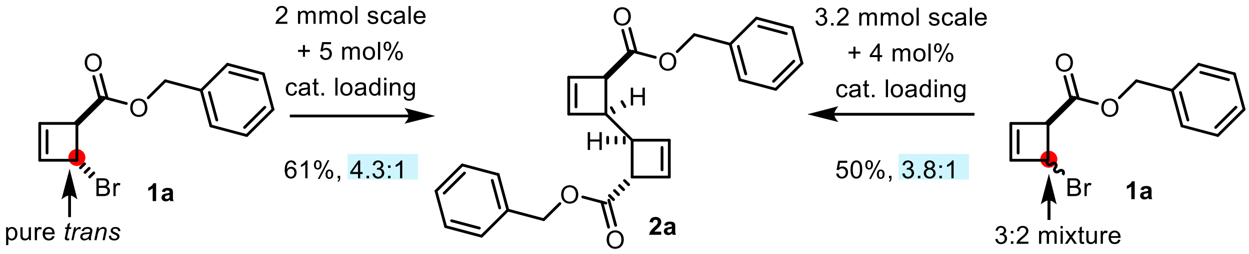

GP4 was followed using ***trans*-1a** (534 mg, 2.0 mmol, 1.0 equiv.) with a reduced catalyst loading, nickel(II) chloride ethylene glycol dimethyl ether complex (22.0 mg, 0.1 mmol, 0.05 equiv.) and terpyridine (23.3 mg, 0.1 mmol, 0.05 equiv.), zinc (523 mg, 8 mmol, 4.0 equiv.) and DMF (16 mL, 0.125M). The desired product **2a** was obtained as a colorless oil (230 mg, 1.22 mmol, 61%) with a diastereomeric ratio of 4:3:1.

The same reaction was also repeated starting from a mixture of ***trans*-1a** and ***cis*-1a** (ratio 3:2, 952 mg, 3.2 mmol, 1.0 equiv.) using nickel(II) chloride ethylene glycol dimethyl ether complex (28.2 mg, 0.128 mmol, 0.04 equiv.), terpyridine (29.9 mg, 0.128 mmol, 0.04 equiv.), zinc (839 mg, 12.8 mmol, 4.0 equiv.) and DMF (40 mL, 0.125M). After column chromatography, **2a** was isolated as a colorless oil (300 mg, 0.8 mmol, 50%) with a diastereomeric ratio of 3.8:1.

Bis(4-methoxybenzyl)-[1,1'-bi(cyclobutane)]-3,3'-diene-2,2'-dicarboxylate (2b)

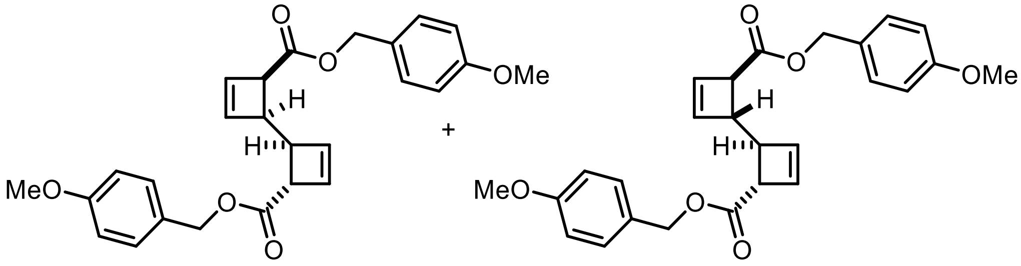

d.r. 4.1:1

Following GP4 using the cis isomer starting material (**1b**, 0.1 mmol), the titled compound was obtained in a diastereomeric mixture with a ratio of 4.1:1 as a colorless oil (13.4 mg, 31 µmol, 62%).

^{1}H NMR (700 MHz, $CDCl_3$): δ 7.27 – 7.25 (m, 4H), 6.88 – 6.85 (m, 4H), 6.25 (d, *J* = 2.7 Hz, 1.6H), 6.22 (d, *J* = 2.6 Hz, 0.4H*), 6.13 (dd, *J* = 2.8, 0.7 Hz, 0.4H*), 6.12 (dd, *J* = 2.8, 0.8 Hz, 1.6H), 5.06 (d, *J* = 3.9 Hz, 4H), 3.80 (s, 6H), 3.36 (app s, 0.4H*), 3.31 (app s, 1.6H), 3.21 (app s, 0.4H*), 3.15 (app s, 1.6H) ppm.

^{13}C NMR (176 MHz, $CDCl_3$): δ 172.7*, 172.6, 159.7, 141.1, 140.2*, 135.0*, 134.6, 130.0, 130.0*, 128.3, 114.1, 114.1*, 66.3, 66.3*, 55.4, 49.9, 49.3*, 49.0, 48.3* ppm.

IR (neat) ν_{max}: 1728, 1515, 1248, 1173, 1160, 1034, 822.

HRMS (ESI+): exact mass calculated for [M+Na]+ ($C_{26}H_{26}O_6Na^+$) requires m/z 457.1627, found m/z 457.1626.

HRMS (ESI) m/z: [M+Na]+ Calculated for $C_{26}H_{26}O_6Na^+$ 457.1627; Found 457.1626.

Bis(3,4-dichlorobenzyl)-[1,1'-bi(cyclobutane)]-3,3'-diene-2,2'-dicarboxylate (2c)

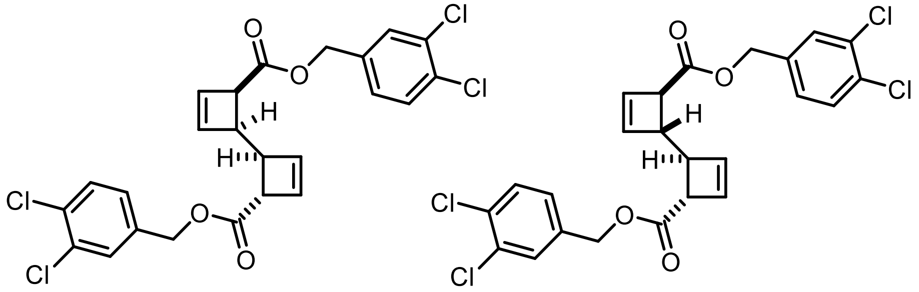

d.r. 4.0:1

Following GP4 using a 5:1 mixture of the cis:trans starting material (**1c**, 0.21 mmol), the titled compound was obtained in a diastereomeric mixture with a ratio of 4:1 as a colorless oil (26 mg, 0.051 mmol, 49%).

^{1}H NMR (600 MHz, CDCl$_3$): δ 7.44 – 7.40 (m, 4H), 7.17 – 7.15 (m, 2H), 6.29 (d, *J* = 2.6 Hz, 1.6H), 6.26 (d, *J* = 2.6 Hz, 0.4H*), 6.17 – 6.15 (m, 2H), 5.08 – 5.06 (m, 4H), 3.40 (s, 0.4H*), 3.36 (s, 1.6H), 3.24 (s, 0.4H*), 3.16 (s, 1.6H) ppm.

^{13}C NMR (151 MHz, CDCl$_3$): δ 172.3*, 172.1, 141.2, 140.3*, 136.3, 134.8*, 134.5, 134.1*, 132.8, 132.5*, 132.5, 132.0*, 130.7*, 130.7, 130.1*, 130.0*, 127.4*, 127.3, 64.9*, 64.9, 50.1, 49.3*, 48.9, 48.1* ppm.

IR (neat) ν_{max}: 2925, 1730, 1474, 1401, 1364, 1263, 1214, 1160, 1131, 1031, 874, 816.

HRMS (ESI) m/z: [M+Na]+ Calculated for $C_{24}H_{18}Cl_4NaO_4^+$ 532.9851; Found 532.9855.

Bis(2,4,6-trimethylbenzyl)-[1,1'-bi(cyclobutane)]-3,3'-diene-2,2'-dicarboxylate (2d)

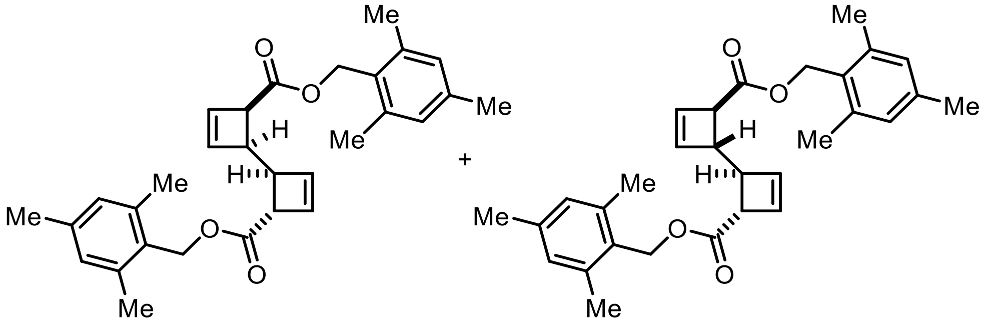

d.r. 2.3:1

Following GP4 using the trans isomer starting material (**1d**, 0.1 mmol), the titled compound was obtained in a diastereomeric mixture with a ratio of 2.3:1 as a colorless oil (10.9 mg, 24 µmol, 48%).

^{1}H NMR (600 MHz, $CDCl_3$): δ 6.87 – 6.85 (m, 4H), 6.22 (d, *J* = 2.6 Hz, 1.4H), 6.20 (d, *J* = 2.6 Hz, 0.6H*), 6.11 – 6.08 (m, 2H), 5.16 (s, 4H), 3.31 (app s, 0.6H*), 3.27 (app s, 1.4H), 3.19 (app s, 0.6H*), 3.16 (app s, 1.4H), 2.32 – 2.31 (m, 12H), 2.28 (s, 6H) ppm.

^{13}C NMR (151 MHz, $CDCl_3$): δ 173.0*, 172.8, 141.0, 140.2*, 138.5, 138.3, 138.3, 135.1*, 134.8, 129.2, 61.5, 61.5, 49.7, 49.4*, 48.8, 48.3*, 21.2, 19.7 ppm.

IR (neat) ν_{max}: 1727, 1252, 1159, 1013, 851, 735.

HRMS (ESI) m/z: $[M+H]^+$ Calculated for $C_{30}H_{35}O_4^+$ 459.2530; Found 459.2523.

Bis(furan-2-ylmethyl)-[1,1'-bi(cyclobutane)]-3,3'-diene-2,2'-dicarboxylate (2e)

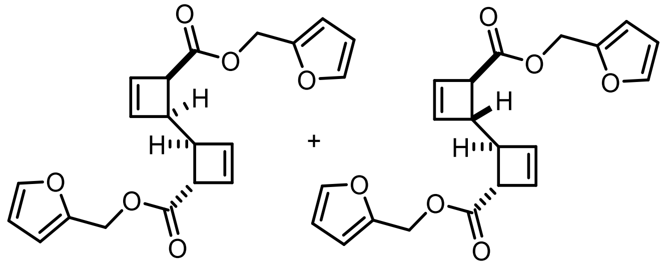

4:1

Following GP4 using the cis isomer starting material (**1e**, 0.1 mmol), the titled compound was obtained in a diastereomeric mixture with a ratio of 4:1 as a colorless oil (6 mg, 17 µmol, 34%).

^{1}H NMR (600 MHz, $CDCl_3$): δ 7.43 – 7.40 (m, J = 8.2 Hz, 2H), 6.42 – 6.40 (m, J = 2.9 Hz, 0.3H*), 6.40 – 6.38 (m, J = 2.8 Hz, 1.7H), 6.37 – 6.34 (m, 2H), 6.25 (d, J = 2.5 Hz, 1.6H), 6.23 – 6.22 (m, 0.4H*), 6.13 – 6.11 (m, 2H), 5.08 – 5.06 (m, 4H), 3.35 (s, 0.4H*), 3.31 (s, 1.6H), 3.21 (s, 0.4H*), 3.17 (s, 1.6H) ppm.

^{13}C NMR (151 MHz, $CDCl_3$) δ 172.4*, 172.3, 149.7, 149.5*, 143.5*, 143.3, 141.1, 140.3*, 134.9*, 134.6, 110.9*, 110.7*, 110.7, 110.6, 58.4, 49.7, 49.2*, 48.8, 48.2*ppm.

IR (neat) ν_{max}: 2923, 2853, 1729, 1502, 1369, 1338, 1260, 1228, 1152, 1119, 1079, 1014, 919.

HRMS (ESI) m/z: $[M+H]^+$ Calculated for $C_{20}H_{18}NaO_6^+$ 377.0996; Found 377.0997.

Di-*tert*-butyl-[1,1'-bi(cyclobutane)]-3,3'-diene-2,2'-dicarboxylate (2f)

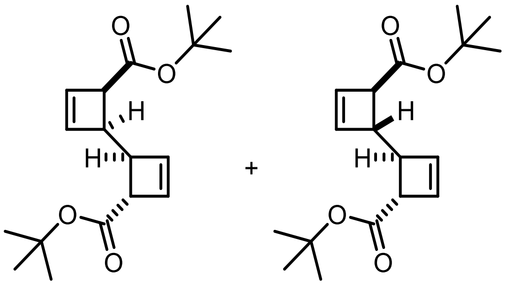

2.9:1

Following GP4 using the trans isomer starting material (**1f**, 0.1 mmol), the titled compound was obtained in a diastereomeric mixture with a ratio of 2.9:1 as light yellow oil (8.6 mg, 28 µmol, 56%).

^{1}H NMR (700 MHz, $CDCl_3$): δ 6.26 (d, J = 2.7 Hz, 1.5H), 6.22 (d, J = 2.5 Hz, 0.5H*), 6.13 – 6.11 (m, 2H), 3.28 (s, 0.5H*), 3.24 (s, 1.5H), 3.13 (s, 0.5H*), 3.04 (s, 1.5H), 1.45 (d, J = 1.2 Hz, 18H) ppm.

^{13}C NMR (176 MHz, $CDCl_3$) δ 172.3*, 172.1, 140.7, 139.8*, 135.0*, 134.7, 80.3, 80.3, 50.3, 49.4*, 49.3*, 28.1, 28.0* ppm.

IR (neat) ν_{max}: 2978, 2930, 1723, 1626, 1257, 1210, 1148, 1119, 955, 907, 847, 793.

HRMS (ESI) m/z: $[M+Na]^+$ Calculated for $C_{18}H_{26}NaO_4^+$ 329.1723; Found 329.1727.

Bis(2-(trimethylsilyl)ethyl)-[1,1'-bi(cyclobutane)]-3,3'-diene-2,2'-dicarboxylate (2g)

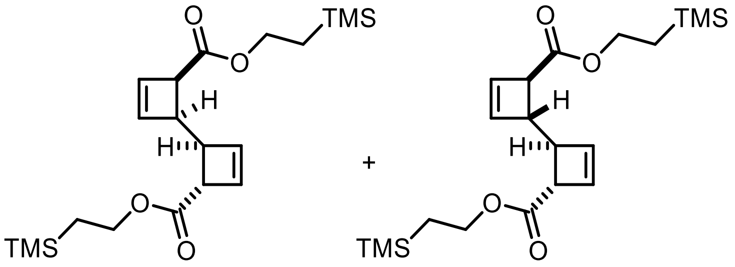

3.9:1

Following GP4 using the cis isomer starting material (**1g**, 0.10 mmol), the titled compound was obtained in a diastereomeric mixture with a ratio of 3.9:1 as a colorless oil (12 mg, 29 µmol, 58%).

^{1}H NMR (700 MHz, CDCl$_3$): δ 6.28 (d, *J* = 2.7 Hz, 1.6H), 6.24 (d, *J* = 2.6 Hz, 0.4H*), 6.15 – 6.13 (m, 2H), 4.20 – 4.16 (m, 4H), 3.33 (s, 0.4H*), 3.29 (s, 1.6H), 3.21 (s, 0.4H*), 3.16 (s, 1.6H), 1.01 – 0.97 (m, 4H), 0.04 (d, *J* = 2.6 Hz, 18H) ppm.

^{13}C NMR (176 MHz, CDCl$_3$) δ 173.1*, 172.9, 141.0, 140.2*, 135.1, 134.8*, 63.0, 49.82, 49.3*, 49.2, 48.6*, 17.5, 17.5*, -1.3 ppm.

IR (neat) ν_{max}: 2953, 2897, 1727, 1627, 1379, 1338, 1303, 1248, 1219, 1160, 1121 1062, 1042, 936.

HRMS (ESI) m/z: $[M+Na]^+$ Calculated for $C_{20}H_{34}NaO_4Si_2^+$ 417.1888; Found 417.1886.

Dimethyl-[1,1'-bi(cyclobutane)]-3,3'-diene-2,2'-dicarboxylate (2h)

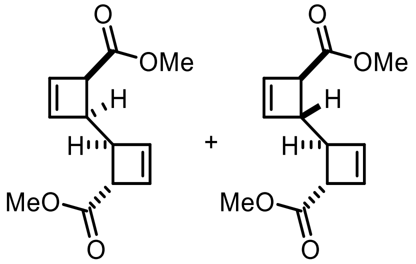

3.8:1

Following GP4 using the trans isomer starting material (**1h**, 0.21 mmol), the titled compound was obtained in a diastereomeric mixture with a ratio of 3.8:1 as a colorless oil (12 mg, 0.054 mmol, 52%).

^{1}H NMR (700 MHz, CDCl$_3$) δ 6.29 (d, *J* = 2.7 Hz, 1.6H), 6.25 (d, *J* = 2.7 Hz, 0.4H*), 6.15 – 6.14 (m, 2H), 3.70 (s, 6H), 3.35 (s, 0.4H*), 3.33 (s, 1.6H), 3.23 (s, 0.4H*), 3.18 (s, 1.6H).

^{13}C NMR (176 MHz, CDCl$_3$): δ 173.1*, 173.0, 140.9, 140.1*, 134.9*, 134.5, 51.9, 49.7, 49.1, 48.7, 48.0 ppm.

IR (neat) ν$_{max}$: 2953, 1730, 1435, 1340, 1310, 1262, 1198, 1167, 1029, 1000.

HRMS (ESI) m/z: [M+Na]$^+$ Calculated for $C_{12}H_{14}NaO_4^+$ 245.0784; Found 245.0781.

Dihexyl-[1,1'-bi(cyclobutane)]-3,3'-diene-2,2'-dicarboxylate (2i)

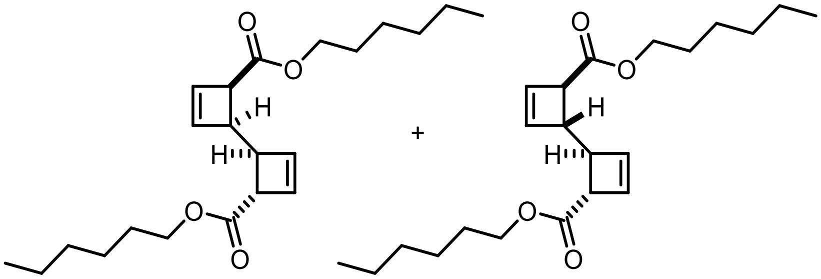

3.5:1

Following GP4 using the trans isomer starting material (**1i**, 0.1 mmol), the titled compound was obtained in a diastereomeric mixture with a ratio of 3.5:1 as a colorless oil (8.5 mg, 23 µmol, 47%).

^{1}H NMR (600 MHz, CDCl$_3$): δ 6.28 (d, *J* = 2.6 Hz, 1.6H), 6.25 (d, *J* = 2.6 Hz, 0.4H*), 6.17 – 6.13 (m, 2H), 4.12 – 4.04 (m, 4H), 3.35 (s, 0.4H*), 3.32 (s, 1.6H), 3.21 (s, 0.4H*), 3.15 (s, 1.6H), 1.66 – 1.60 (m, 4H), 1.37 – 1.26 (m, 12H), 0.89 (t, *J* = 6.9 Hz, 6H) ppm.

^{13}C NMR (151 MHz, CDCl$_3$): δ 172.8, 172.7*, 140.9*, 140.1, 134.9, 134.6, 64.7, 49.9, 49.3*, 49.0, 48.3*, 31.4, 28.6, 25.5, 22.5, 14.0 ppm.

IR (neat) ν$_{max}$: 2958, 2923, 2856, 1729, 1468, 1340, 1306, 1275, 1258, 1194, 1163, 1060, 797.

HRMS (ESI) m/z: [M+Na]$^+$ Calculated for $C_{22}H_{34}NaO_4^+$ 385.2349; Found 385.2350.

Dicyclopentyl (1R,1'R,2R,2'S)-[1,1'-bi(cyclobutane)]-3,3'-diene-2,2'-dicarboxylate (2j)

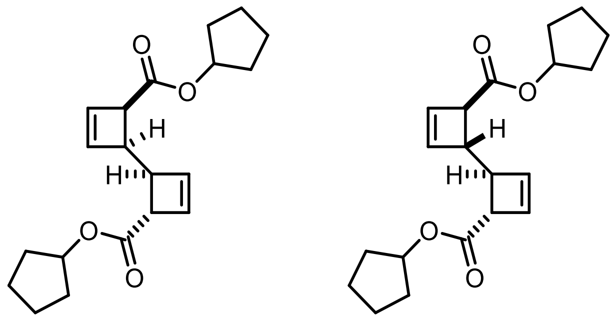

3.8:1

Following GP4 using the trans isomer starting material (**1j**, 0.081 mmol), the titled compound was obtained in a diastereomeric mixture with a ratio of 3.8:1 as a colorless oil (6.0 mg, 18 µmol, 45%).

^{1}H NMR (700 MHz, CDCl$_3$): δ 6.26 (d, *J* = 2.6 Hz, 1.6H), 6.23 (d, *J* = 2.6 Hz, 0.4H*), 6.14 (dd, *J* = 2.8, 0.8 Hz, 0.4H*), 6.13 (dd, *J* = 2.8, 0.8 Hz, 1.6H), 5.18 (dt, *J* = 9.1, 3.1 Hz, 2H), 3.31 (s, 0.4H*), 3.26 (s, 1.6H), 3.16 (s, 0.4H*), 3.07 (s, 1.6H), 1.88 – 1.82 (m, 4H), 1.74 – 1.65 (m, 8H), 1.61 – 1.58 (m, 4H) ppm.

^{13}C NMR (176 MHz, CDCl$_3$): δ 172.7*, 172.6, 140.9, 140.0*, 135.1*, 134.7, 77.3*, 77.2, 50.4, 49.6, 49.4*, 48.5*, 32.9, 32.8, 32.8*, 23.9, 23.9*, 23.8* ppm.

IR (neat) ν_{max}: 2961, 1726, 1379, 1323, 1266, 1160, 1035, 957.

HRMS (ESI) m/z: [M+Na]$^+$ Calculated for $C_{20}H_{26}NaO_4^+$ 353.1723; Found 353.1714.

Di(pent-4-en-1-yl)-[1,1'-bi(cyclobutane)]-3,3'-diene-2,2'-dicarboxylate (2k)

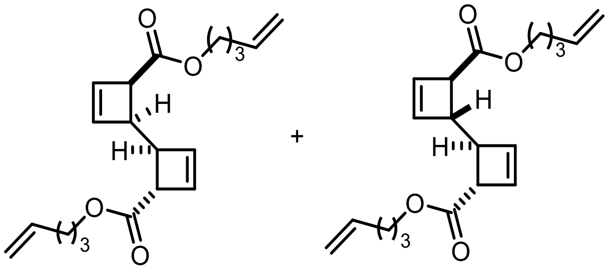

d.r. 3.5:1

Following GP4 using the cis isomer starting material (**1k**, 0.1 mmol), the titled compound was obtained in a diastereomeric mixture with a ratio of 3.5:1 as a colorless oil (7.4 mg, 22 µmol, 45%).

^{1}H NMR (400 MHz, CDCl$_3$): δ 6.29 (d, *J* = 2.5 Hz, 1.6H), 6.25 (d, *J* = 2.4 Hz, 0.4H*), 6.17 – 6.13 (m, 2H), 5.85 – 5.75 (m, 2H), 5.06 – 4.97 (m, 4H), 4.12 – 4.08 (m, 4H), 3.35 (app s, 0.4H*), 3.32 (app s, 1.6H), 3.22 (app s, 0.4H*), 3.15 (app s, 1.6H), 2.14 – 2.09 (m, 4H), 1.77 – 1.71 (m, 4H) ppm.

^{13}C NMR (101 MHz, $CDCl_3$): δ 172.9*, 172.7, 141.0, 140.2*, 137.6, 135.0*, 134.7, 115.5, 64.1, 50.1, 49.4*, 49.2, 48.4*, 30.2, 28.0 ppm.

IR (neat) ν_{max}: 1729, 1260, 1165, 1020, 993, 913, 763.

HRMS (ESI) m/z: $[M+Na]^+$ Calculated for $C_{20}H_{26}NaO_4^+$ 353.1723; Found 353.1723.

Diallyl-[1,1'-bi(cyclobutane)]-3,3'-diene-2,2'-dicarboxylate (2l)

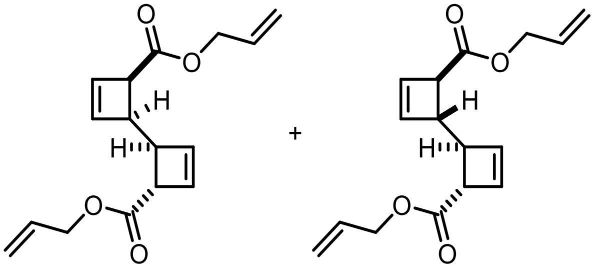

d.r. 3.7:1

Following GP4 using the trans isomer starting material (**1l**, 0.29 mmol), the titled compound was obtained in a diastereomeric mixture with a ratio of 3.7:1 as a colorless oil (14.8 mg, 54 µmol, 37%).

^{1}H NMR (500 MHz, CDCl$_3$): δ 6.30 (d, *J* = 2.7 Hz, 1.5H), 6.26 (d, *J* = 2.7 Hz, 0.5H*), 6.17 – 6.15 (m, 2H), 5.96 – 5.88 (m, 2H), 5.32 (dd, *J* = 17.2, 1.4 Hz, 2H), 5.23 (dd, *J* = 10.4, 1.2 Hz, 2H), 4.60 (d, *J* = 5.6 Hz, 4H), 3.39 (app s, 0.5H*), 3.36 (app s, 1.5H), 3.25 (app s, 0.5H*), 3.19 (app s, 1.5H) ppm.

^{13}C NMR (126 MHz, CDCl$_3$): δ 172.5*, 172.4, 141.1, 140.3*, 135.0*, 134.7, 132.3, 118.3*, 118.2, 65.3, 50.0, 49.4*, 49.0, 48.3* ppm.

IR (neat) ν$_{max}$: 1730, 1274, 1160, 991, 933.

HRMS (ESI) m/z: [M+H]$^+$ Calculated for $C_{16}H_{19}O_4^+$ 275.1278; Found 275.1278.

Di(but-2-yn-1-yl)-[1,1'-bi(cyclobutane)]-3,3'-diene-2,2'-dicarboxylate (2m)

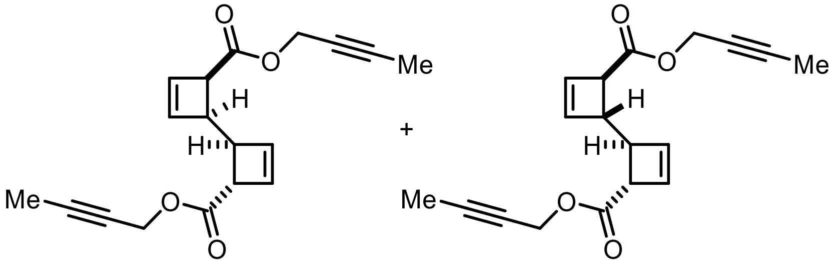

d.r. 4:1

Following GP4 using the trans isomer starting material (**1m**, 84 µmol), the titled compound was obtained in a diastereomeric mixture with a ratio of 4.0:1 as a colorless oil (6.6 mg, 22 µmol, 55%).

^{1}H NMR (600 MHz, CDCl$_3$): δ 6.29 (d, *J* = 2.6 Hz, 1.6H), 6.26 (d, *J* = 2.6 Hz, 0.4H*), 6.18 – 6.13 (m, 2H), 4.66 (d, *J* = 2.2 Hz, 2H), 3.38 (app s, 0.4H*), 3.35 (app s, 1.6H), 3.26 (app s, 0.4H*), 3.23 (app s, 1.6H), 1.86 (t, *J* = 2.3 Hz, 3H) ppm.

^{13}C NMR (101 MHz, CDCl$_3$): δ 172.2*, 172.1, 141.2, 140.4*, 134.9*, 134.6, 83.4, 73.3, 53.1, 49.5, 49.1*, 48.7, 48.1*, 3.8 ppm.

IR (neat) ν_{max}: 1735, 1236, 1159, 991.

HRMS (ESI) m/z: $[M+Na]^+$ Calculated for $C_{18}H_{18}O_4Na^+$ 321.1103; Found 321.1095.

Diphenyl-[1,1'-bi(cyclobutane)]-3,3'-diene-2,2'-dicarboxylate (2n)

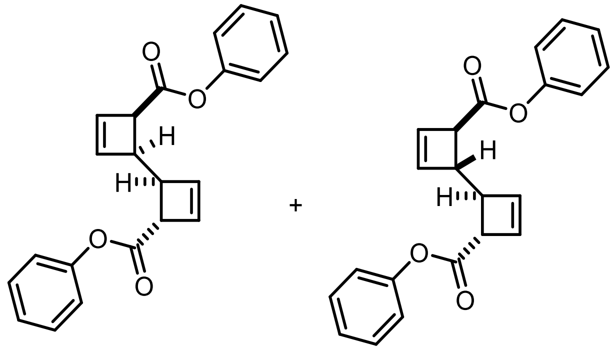

4.0:1

Following GP4 using the trans isomer starting material (**1n**, 0.2 mmol), the titled compound was obtained in a diastereomeric mixture with a ratio of 4.0:1 as a colorless oil (9.4 mg, 27 µmol, 54%).

^{1}H NMR (600 MHz, CDCl$_3$): δ 7.38 (t, *J* = 7.8 Hz, 4H), 7.23 (t, *J* = 7.4 Hz, 2H), 7.11 (d, *J* = 7.8 Hz, 4H), 6.41 (d, *J* = 2.6 Hz, 1.6H), 6.38 (d, *J* = 2.5 Hz, 0.4H*), 6.30 (dd, *J* = 7.1, 2.8 Hz, 2H), 3.68 (s, 0.4H*), 3.64 (s, 1.6H), 3.49 (s, 0.4H*), 3.42 (s, 1.6H) ppm.

^{13}C NMR (151 MHz, CDCl$_3$): δ 171.1*, 171.0, 150.9, 141.4, 140.5*, 134.9*, 134.5, 129.6, 126.0, 121.6*, 121.6, 50.2, 49.6*, 49.1, 48.4* ppm.

IR (neat) ν_{max}: 2924, 1745, 1593, 1492, 1456, 1340, 1303, 1257, 1194, 1162, 1125, 1071, 1026, 934.

HRMS (ESI) m/z: $[M+Na]^+$ Calculated for $C_{22}H_{18}NaO_4^+$ 369.1097; Found 369.1102.

Bis(2-(ethoxycarbonyl)phenyl)-[1,1'-bi(cyclobutane)]-3,3'-diene-2,2'-dicarboxylate (2o)

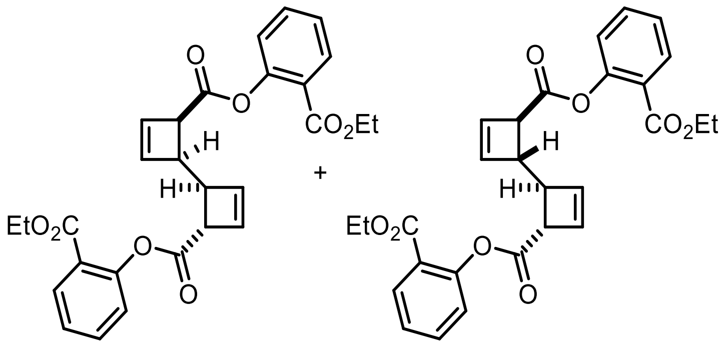

d.r. 4.3:1

Following GP4 using the cis isomer starting material (**1o**, 0.21 mmol), the titled compound was obtained in a diastereomeric mixture with a ratio of 4.3:1 as a colorless oil (30.7 mg, 63 µmol, 60%).

^{1}H NMR (700 MHz, CDCl$_3$): δ 8.00 (dd, *J* = 7.8, 1.4 Hz, 2H), 7.54 (dd, *J* = 11.0, 4.4 Hz, 2H), 7.30 (t, *J* = 7.6 Hz, 2H), 7.10 (d, *J* = 8.0 Hz, 1.6H), 6.44 (d, *J* = 2.5 Hz, 0.4H*), 6.40 (d, *J* = 2.4 Hz, 1.6H), 6.34 (d, *J* = 2.7 Hz, 0.4H*), 6.33 (d, *J* = 2.5 Hz, 1.6H), 4.29 (q, *J* = 7.1 Hz, 4H), 3.77 (app s, 0.4H*), 3.75 (app s, 1.6H), 3.63 (app s, 0.4H*), 3.58 (app s, 1.6H), 1.33 (t, *J* = 7.1 Hz, 6H) ppm.

^{13}C NMR (151 MHz, CDCl$_3$): δ 171.2*, 171.1, 164.5, 150.7, 141.5, 140.6*, 134.9*, 134.5, 133.7, 131.8*, 131.7, 126.1, 124.0, 123.9*, 123.8, 61.2, 49.8, 49.2*, 48.8, 48.3*, 14.4 ppm.

IR (neat) ν_{max}: 1756, 1721, 1294, 1254, 1201, 1137, 1119, 1080, 742.

HRMS (ESI) m/z: $[M+Na]^+$ Calculated for $C_{28}H_{26}O_8Na^+$ 513.1520; Found 513.1523.

S,S-Dibenzyl-[1,1'-bi(cyclobutane)]-3,3'-diene-2,2'-bis(carbothioate) (2p)

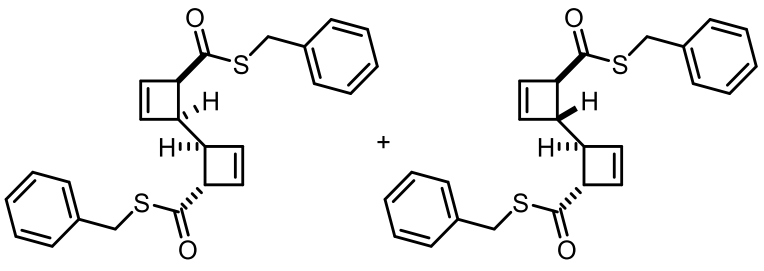

8.2:1

Following GP4, with a reaction time of 30 min instead, using the trans isomer starting material (**1p**, 0.1 mmol), the titled compound was obtained in a diastereomeric mixture with a ratio of 8.2:1 as light yellow oil (9.4 mg, 23 µmol, 45%).

^{1}H NMR (400 MHz, CDCl$_3$) δ 7.30 – 7.27 (m, 8H), 7.25 – 7.22 (m, 2H*), 6.37 – 6.35 (m, 1.8H), 6.34 – 6.32 (m, 0.2H*), 6.19 – 6.17 (m, 2H), 4.14 – 4.11 (m, 4H), 3.56 – 3.53 (m, 2H), 3.21 (s, 0.2H*), 3.15 (s, 1.8H) ppm.

^{13}C NMR (151 MHz, CDCl$_3$) δ 198.1*, 198.0, 141.9, 141.1*, 137.5*, 137.4, 134.8*, 134.6, 128.9, 128.6, 127.3*, 56.7, 56.4*, 51.1, 50.4*, 33.21 ppm.

IR (neat) ν_{max}: 2924, 2853, 1789, 1669, 1601, 1453, 1410, 1071, 1027, 916, 816, 769.

HRMS (ESI) m/z: [M+Na]$^+$ Calculated for $C_{24}H_{22}NaO_2S_2^+$ 429.0953; Found 429.0953.

S,S-bis(4-fluorobenzyl)-[1,1'-bi(cyclobutane)]-3,3'-diene-2,2'-bis(carbothioate) (2q)

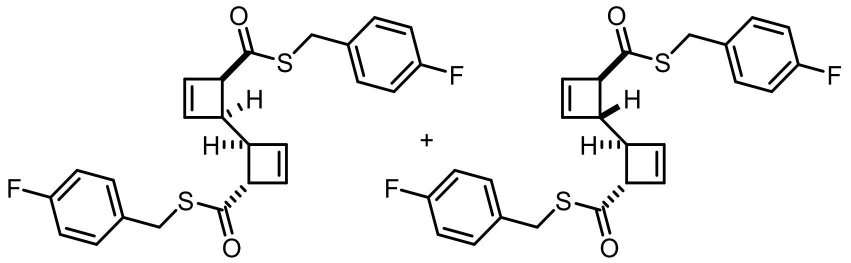

6.5:1

Following GP4, with a reaction time of 30 min instead, using the trans isomer starting material (**1q**, 0.10 mmol), the titled compound was obtained in a diastereomeric mixture with a ratio of 6.5:1 as light yellow oil (7 mg, 16 µmol, 30%).

^{1}H NMR (700 MHz, CDCl$_3$): δ 7.26 – 7.24 (m, 4H), 6.98 – 6.96 (m, 4H), 6.35 (d, *J* = 2.7 Hz, 1.7H), 6.33 (d, *J* = 2.7 Hz, 0.3H*), 6.18 – 6.17 (m, 2H), 4.09 (s, 4H), 3.54 (s, 0.3H*), 3.52 (s, 1.7H), 3.19 (s, 0.3H*), 3.13 (s, 1.7H) ppm.

^{13}C NMR (176 MHz, CDCl$_3$): δ 198.2*, 198.0, 162.16 (d, *J* = 246.0 Hz), 142.1, 141.3*, 134.9*, 134.7, 133.48 (d, *J* = 3.3 Hz), 130.62 (d, *J* = 8.1 Hz), 115.65 (d, *J* = 21.5 Hz), 56.9, 56.5*, 51.2, 50.5*, 32.6, 32.6* ppm.

^{19}F NMR (377 MHz, CDCl$_3$) δ -115.08 ppm.

IR (neat) ν_{max}: 2924, 1676, 1603, 1510, 1223, 1158, 1093, 1033, 836.

HRMS (ESI) m/z: [M+Na]$^+$ Calculated for $C_{24}H_{20}F_2NaO_2S_2^+$ 465.0765; Found 465.0765.

S,S-bis(4-methoxybenzyl)-[1,1'-bi(cyclobutane)]-3,3'-diene-2,2'-bis(carbothioate) (2r)

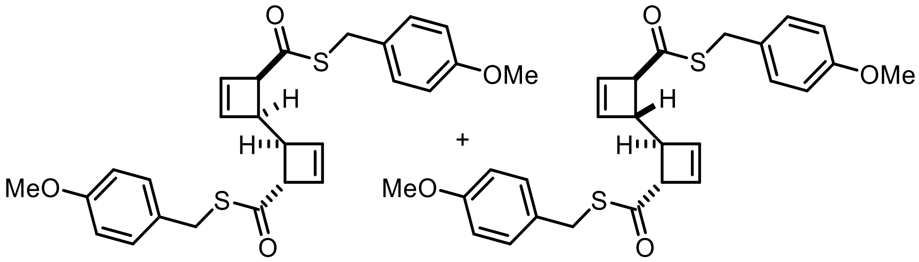

8.0:1

Following GP4, with a reaction time of 30 min instead, using the trans isomer starting material (**1r**, 0.098 mmol), the titled compound was obtained in a diastereomeric mixture with a ratio of 8.0:1 as light yellow oil (10 mg, 21 µmol, 44%).

^{1}H NMR (600 MHz, $CDCl_3$): δ 7.21 (d, *J* = 8.6 Hz, 4H), 6.82 (d, *J* = 8.6 Hz, 4H), 6.35 (d, *J* = 2.7 Hz, 1.8H), 6.32 (d, *J* = 2.6 Hz, 0.2H*), 6.17 (d, *J* = 2.6 Hz, 2H), 4.08 (s, 4H), 3.78 (s, 6H), 3.54 (s, 0.2H*), 3.52 (s, 1.8H), 3.20 (s, 0.2H*), 3.14 (s, 1.8H) ppm.

^{13}C NMR (151 MHz, $CDCl_3$): δ 198.3, 159.0, 142.0, 141.2*, 135.0*, 134.8, 130.2, 129.6, 114.2, 56.9, 56.6*, 55.4, 51.2, 50.6*, 32.9 ppm.

IR (neat) ν_{max}: 2930, 1668, 1607, 1511, 1464, 1301, 1251, 1240, 1176, 1131, 1094, 1031, 1001, 837.

HRMS (ESI) m/z: $[M+Na]^+$ Calculated for $C_{26}H_{26}NaO_4S_2^+$ 489.1165; Found 489.1165.

3.5. Unsuccessful and low-yielding Substrates

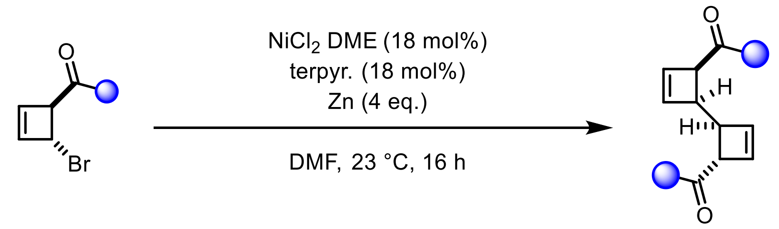

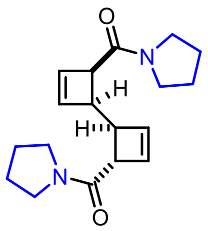

from *trans* SM
0 %
(decomposition)

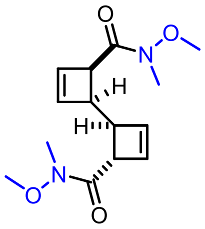

from *trans* SM
complex mixture

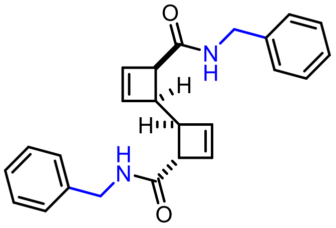

from *trans* SM
complex mixture

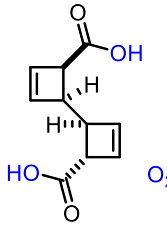

from *trans*
(8%), 2.1:1 dr

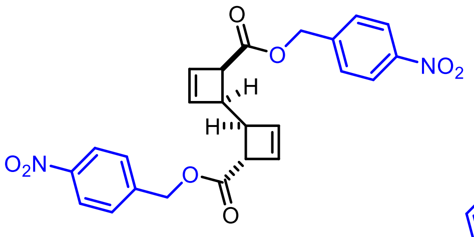

from trans
only SM

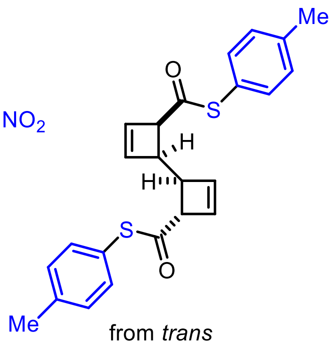

from *trans*
complex mixture

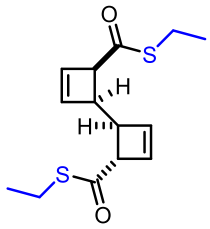

from *cis*
no conversion

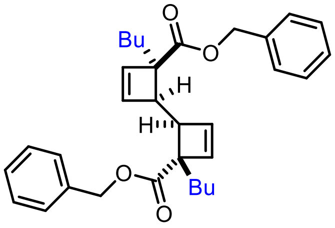

from *cis*
decomposition

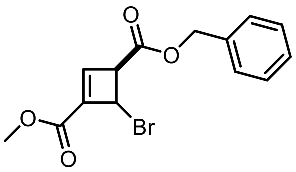

failed preparation
from coumalate

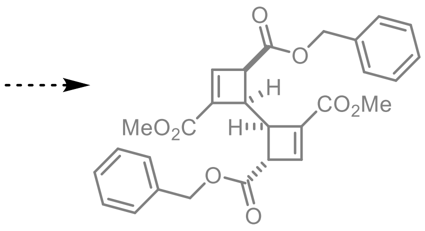

3.6. Application

3.6.1. Thermal Ring-Opening of Dimer 2a

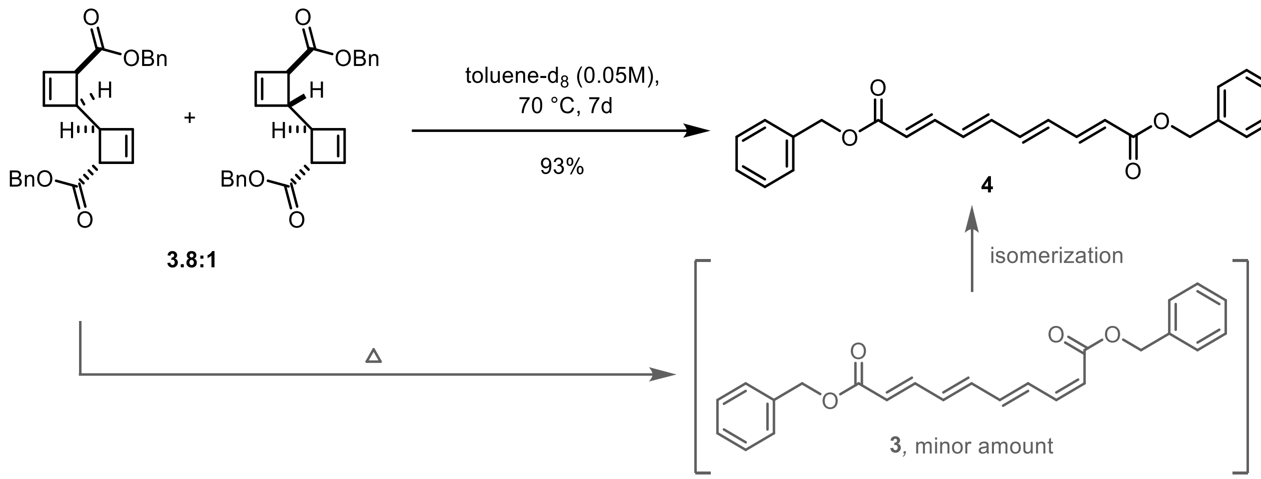

Cyclobutene dimer 2a (23.6 mg, 0.063 mmol, 1.0 equiv.) in toluene-d_8 (0.05 M) was added to a reaction vessel and heated to 70 °C (no argon atmosphere required). The progress of the reaction was monitored by taking small amounts for NMR analysis. After 5 days, full conversion was observed and heating was stopped. The solvent was evaporated and the crude material was subjected to column chromatography (heptane/EtOAc) to afford all *trans*-tetraene **4** as a white solid (22 mg, 0.059 mmol, 93%). In addition to compound **4**, small traces of a tetraene (**3**) representing the *Z,E,E,E* isomer (expected conrotatory ring opening product of the cis-trans-cyclobutene dimer) were also isolated.

Note: All our attempts to obtain larger amounts of **3**, i.e., lower reaction temperature or shorter reaction time, were unsuccessful, leading us to believe that the isomerization of **3** to **4** is a relatively fast reaction process.

Dibenzyl (2E,4E,6E,8E)-deca-2,4,6,8-tetraenedioate (4)

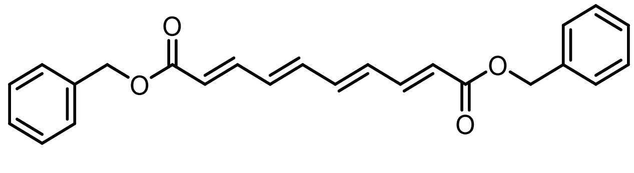

^{1}H NMR (400 MHz, CDCl$_3$): δ 7.43 – 7.31 (m, 12H), 6.65 – 6.57 (m, 2H), 6.47 (td, J = 11.2, 3.3 Hz, 2H), 6.01 (d, J = 15.3 Hz, 2H), 5.20 (s, 4H) ppm.

^{13}C NMR (101 MHz, CDCl$_3$): δ 166.7, 144.1, 139.3, 136.1, 133.8, 128.7, 128.4, 122.7, 66.5 ppm.

IR (neat) ν_{max}: 1703, 1621, 1315, 1296, 1227, 1159, 1121, 1015, 1004, 824, 7333, 723, 691.

HRMS (ESI⁺): exact mass calculated for $[M+H]^+$ ($C_{24}H_{23}O_4^+$) requires m/z 375.1591, found m/z 375.1586.

Dibenzyl (2Z,4E,6E,8E)-deca-2,4,6,8-tetraenedioate (3)

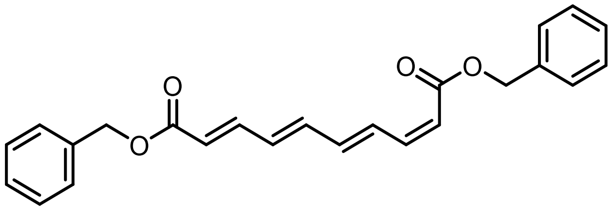

After column chromatography, only very small amounts of this tetraene could be isolated, so we could only characterize the compound by ^{1}H NMR and verify its mass by HRMS.

^{1}H NMR (400 MHz, CDCl$_3$): δ 7.75 – 7.66 (m, 1H), 7.43 – 7.30 (m, *J* = 8.3, 5.2 Hz, 11H), 6.72 – 6.61 (m, *J* = 2H), 6.56 – 6.43 (m, 2H), 6.00 (d, *J* = 15.2 Hz, 1H), 5.79 (d, *J* = 11.2 Hz, 1H), 5.21 (s, 2H), 5.19 (s, 2H) ppm.

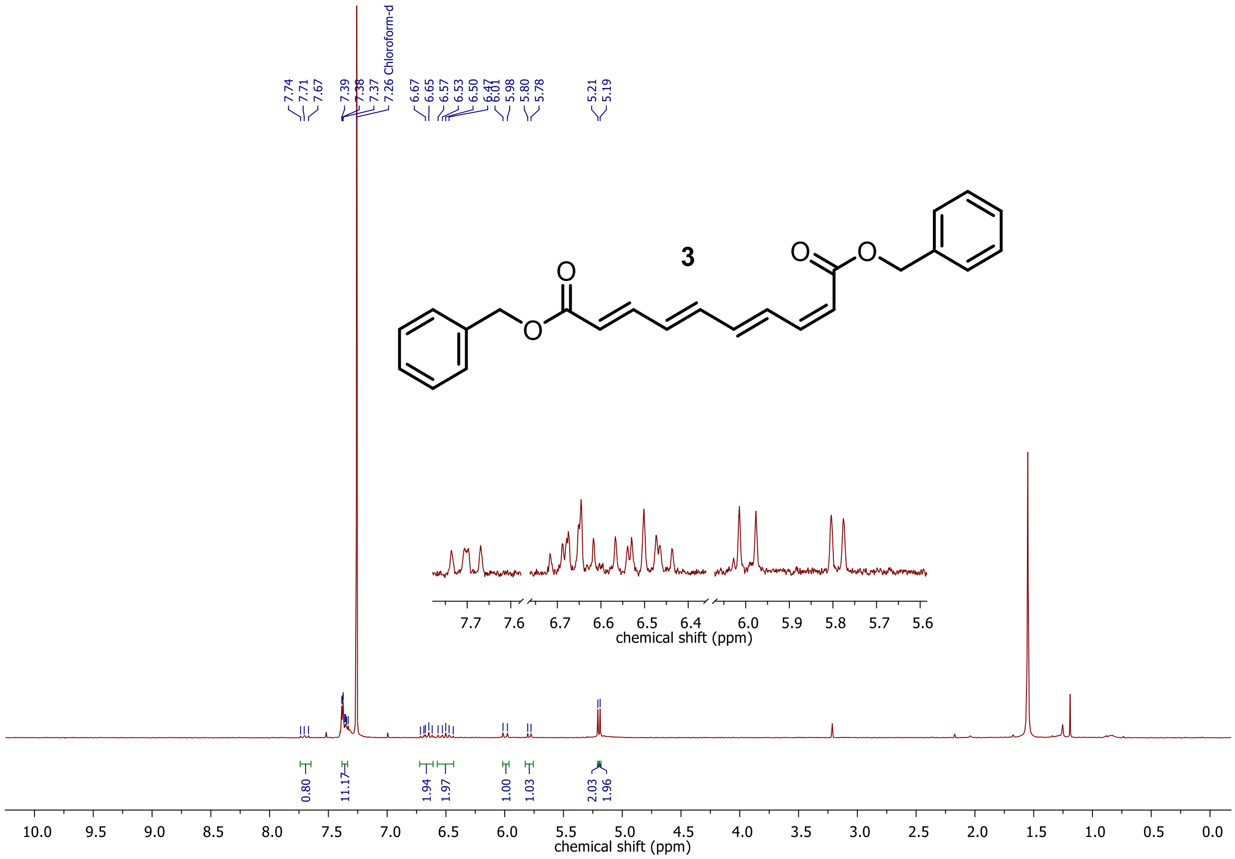

3.6.2 Hydrogenation of Cyclobutene Dimer 2k

Bis(2-(ethoxycarbonyl)phenyl)-[1,1'-bi(cyclobutane)]-2,2'-dicarboxylate (S3)

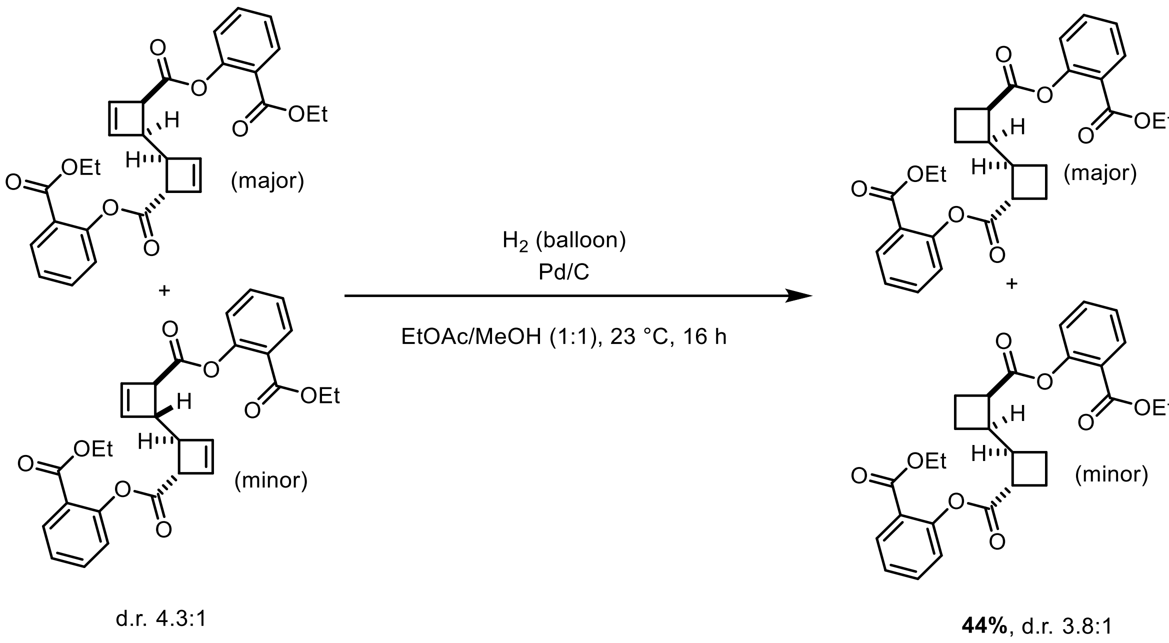

A flame-dried Schlenk flask under Argon atmosphere was loaded with Pd/C (46.4 mg, 44 µmol, 1.0 equiv., 10% on carbon) and cyclobutene dimer **2k** (21.4 mg, 44 µmol, 1.0 equiv.). Dry MeOH (2 mL) and EtOAc (2 mL) were then added (11.0 mM solution).The reaction mixture was degassed with an H_2 balloon for 30 min and then kept under an H_2 atmosphere. The reaction was stirred for 16 h before passing through a Celite pad with MeOH and EtOAc. The filtrate was concentrated and subjected to column chromatography (gradient: heptane to heptane/EtOAc (7:3)). The titled compound was obtained as a colorless oil (10 mg, 20 µmol, 44%) in a diastereomeric ratio of 3.8:1.

^{1}H NMR (700 MHz, CDCl$_3$): δ 7.93 – 7.89 (m, 2H), 7.45 – 7.42 (m, 0.4H*), 7.41 (td, *J* = 7.8, 1.7 Hz, 1.6H), 7.23 – 7.19 (m, 2H), 6.99 (dd, *J* = 8.1, 0.9 Hz, 0.4H*), 6.94 (dd, *J* = 8.1, 0.9 Hz, 1.6H), 4.22 (q, *J* = 7.1 Hz, 4H), 3.21 – 3.16 (m, 2H), 2.96 – 2.90 (m, 2H), 2.34 – 2.28 (m, 2H), 2.18 – 2.12 (m, 2H), 2.03 – 1.95 (m, 2H), 1.81 – 1.70 (m, 2H), 1.26 (t, *J* = 7.2 Hz, 6H) ppm.

^{13}C NMR (176 MHz, CDCl$_3$): δ 173.3, 164.6, 150.7, 133.7*, 133.6, 131.7*, 131.6, 126.0*, 125.9, 124.1, 124.0*, 123.9, 123.8*, 61.2*, 61.2, 43.4*, 43.0, 41.7, 41.6*, 21.9*, 21.7, 21.3, 21.1*, 14.4 ppm.

IR (neat) ν_{max}: 1754,1720, 1294, 1253, 1199, 1133, 1120, 1077, 736.

HRMS (ESI) m/z: [M+Na]$^+$ Calculated for $C_{28}H_{30}O_8Na^+$ 517.1833; Found 517.1838.

3.6.3. Hydrogenolysis + Hydrogenation of Dimer 2a

[1,1'-Bi(cyclobutane)]-2,2'-dicarboxylic acid (5)

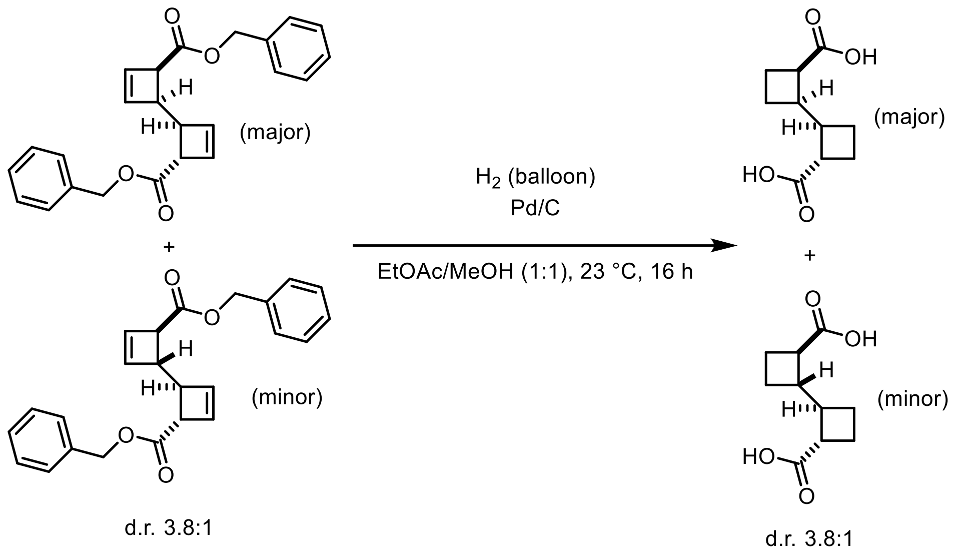

A flame-dried Schlenk flask under Argon atmosphere was loaded with Pd/C (106 mg, 0.1 mmol, 1.0 equiv., 10% on carbon) and cyclobutene dimer **2a** (37.4 mg, 0.1 mmol, 1.0 equiv.). Then, dry MeOH (4 mL) and EtOAc (4 mL) were added (12.5 mM solution). The reaction mixture was degassed with a H_2 balloon for 30 min and then kept under a H_2 atmosphere. The reaction was stirred for 16 h, before it was passed though a Celite pad using MeOH and EtOAc. The filtrate was concentrated and subjected to column chromatography (gradient: pure EtOAc then heptane/EtOAc (8:2)+ 3% HOAc). Finally, the titled compound was obtained a white solid (23 mg, 0.095 mmol, 95%) in a diastereomeric ratio of 3.8:1.

^{1}H NMR (600 MHz, MeOD): δ 2.85 (q, *J* = 8.5 Hz, 2H), 2.65 – 2.56 (m, 2H), 2.10 – 2.02 (m, 4H), 1.96 – 1.86 (m, 2H), 1.73 – 1.61 (m, 2H) ppm.

^{13}C NMR (151 MHz, MeOD): δ 178.8, 178.7*, 44.7*, 44.5, 42.7, 42.6*, 22.4*, 22.3 (2C), 22.1* ppm.

IR (neat) ν_{max}: 1692, 1416, 1288, 1258, 1216, 1192, 934, 739.

HRMS (ESI) m/z: $[M+Na]^+$ Calculated for $C_{10}H_{14}O_4Na^+$ 221.0790; Found 221.0782.

3.6.4. Ester Hydrolysis of Dimer 2a

[1,1'-bi(cyclobutane)]-3,3'-diene-2,2'-dicarboxylic acid (6)

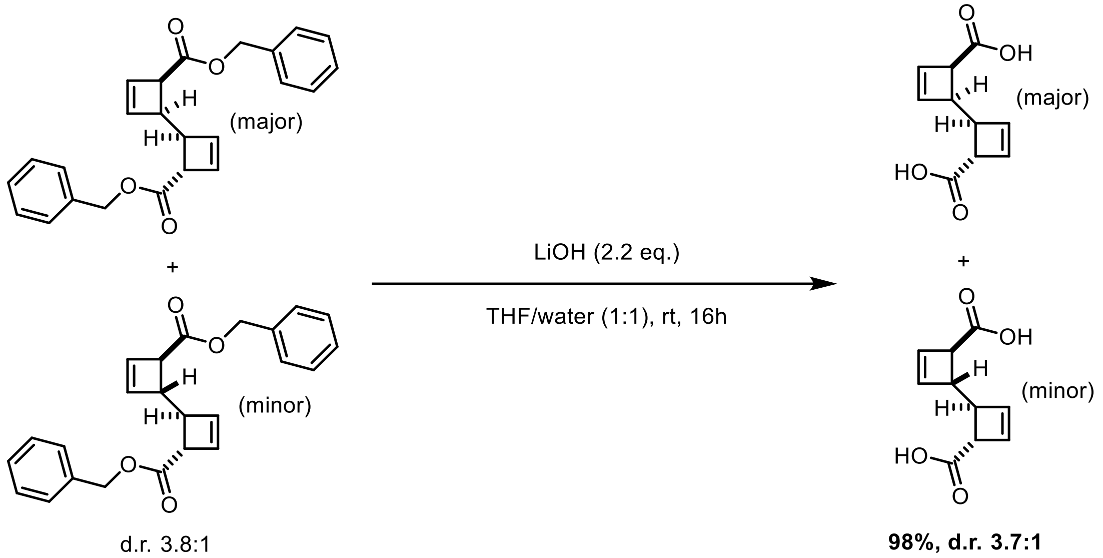

A vial was loaded with a solution of dimer **2a** (37.4 mg, 0.1 mmol, 1.0 equiv.) in THF (1 mL), subsequently water (1 mL) was added. Then, LiOH (9.2 mg, 0.22 mmol, 2.2 equiv.) was added in one portion. The resulting mixture was stirred for 15 h. The, the reaction was quenched using a saturated aqueous solution of NH_4Cl (small amounts). The aqueous phase was washed twice with EtOAc via extraction. The aqueous phase was concentrated and the crude material subjected to column chromatography (gradient: pure EtOAc then heptane/EtOAc (8:2)+ 3% HOAc). The desired acid was obtained as white solid (19.0 mg, 0.098 mmol, 98%) in a diastereomeric ratio of 3.7:1.

^{1}H NMR (600 MHz, Acetone): δ 6.34 (d, *J* = 2.4 Hz, 1.6H), 6.28 (d, *J* = 2.3 Hz, 0.4H*), 6.17 (d, *J* = 2.3 Hz, 0.4H*), 6.15 (d, *J* = 2.3 Hz, 1.6H), 3.37 – 3.34 (m, 2H), 3.23 (s, 0.4H*), 3.15 (s, 1.6H) ppm.

^{13}C NMR (151 MHz, Acetone): δ 173.7*, 173.6, 141.5, 140.7*, 136.0*, 135.7, 50.8, 50.1*, 49.8, 49.0* ppm.

IR (neat) ν_{max}: 1695, 1276, 1238, 1212, 1184, 972, 755, 723.

HRMS (ESI) m/z: $[M+Na]^+$ Calculated for $C_{10}H_{10}O_4Na^+$ 217.0477; Found 217.0468.

3.7. Mechanistic Studies

3.7.1. Dimerization with Ether 7

4,4'-bis((benzyloxy)methyl)-[1,1'-bi(cyclobutane)]-2,2'-diene (8)

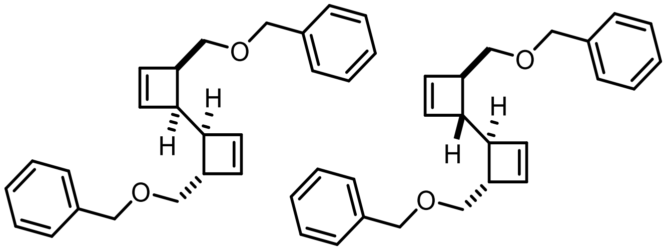

1.3:1

Following GP4 using ether **7** (0.095 mmol), the titled compound was obtained in a diastereomeric mixture with a ratio of 1.3:1 as yellowish oil (6.1 mg, 18 µmol, 38%).

^{1}H NMR (600 MHz, $CDCl_3$) δ 7.34 – 7.32 (m, 8H), 7.28 – 7.25 (m, 2H), 6.18 – 6.17 (m, 1H), 6.16 – 6.14 (m, 3H), 4.55 – 4.50 (m, 4H), 3.57 – 3.53 (m, 4H), 2.79 – 2.74 (m, 2H), 2.65 (s, 1H*), 2.62 (s, 1H) ppm.

^{13}C NMR (151 MHz, $CDCl_3$) δ 139.4, 139.1*, 138.7, 137.4*, 137.4, 128.3, 127.5, 127.5*, 127.4*, 73.0, 73.0, 72.9, 72.9, 49.7, 49.4*, 47.2, 46.7* ppm.

IR (neat) ν_{max}: 2924, 2850, 1453, 1361, 1277, 1260, 1204, 1113, 1097, 1071, 1028, 934, 904, 731.

HRMS (ESI) m/z: $[M+Na]^+$ Calculated for $C_{24}H_{26}NaO_2^+$ 369.1825; Found 369.1829.

3.7.2. Epimerization Study of 2a

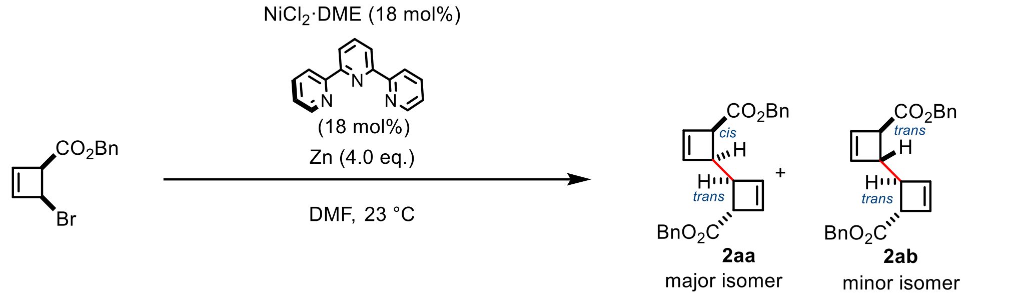

aliquots taken after different times

after 5 min	after 40 min	after 16h
**d.r. 4.1:1**	**d.r. 4.0:1**	**d.r. 3.9:1**

3.8. Determination of the Relative Stereochemistry of Cyclobutene Dimers

Listing of possible diastereoisomers in the radical coupling of cyclobutenes:

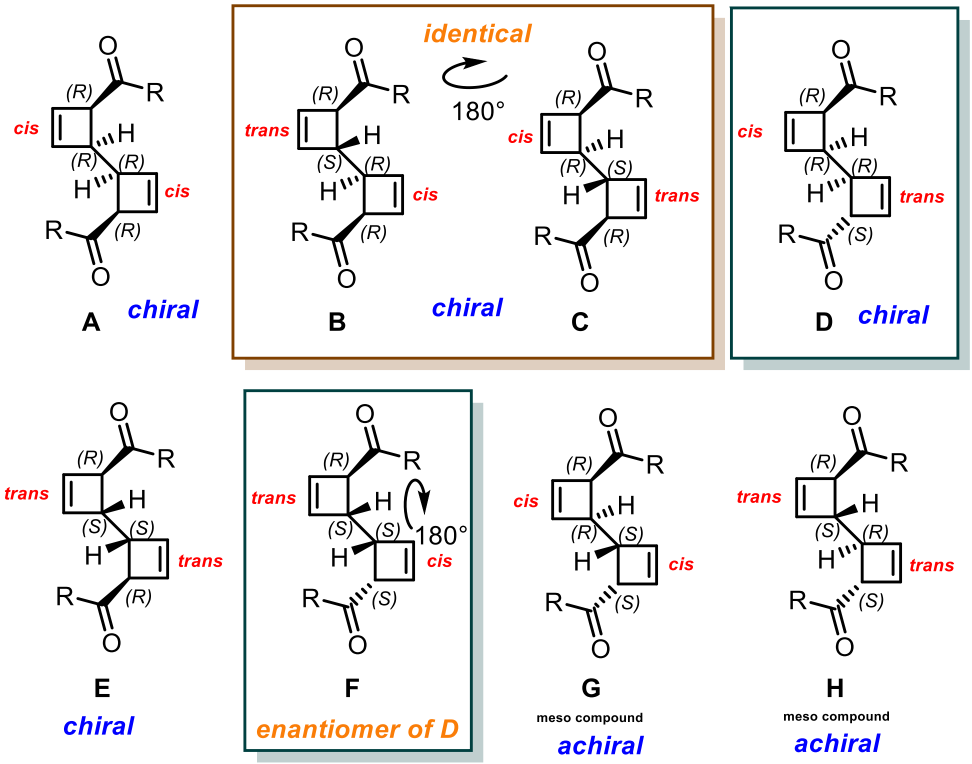

Theoretically, 8 diasteroisomers can be formed in this reaction. However, due to symmetry, B and C as well as D and F are identical, so that only 6 isomers are formed. 2 of them are cis-trans, 2 are trans-trans, 2 are cis-cis. Of the latter two, there is one diastereomer each, which is a meso compound (see G and H).

<u>**Evidence for Major Isomer (X-Ray):**</u>

To obtain an X-ray structure of a cyclobutene dimer for structural confirmation, compound **2sa** was targeted (most other dimers are oils). However, under our nickel-catalyzed conditions, no reaction occurred and only starting material was observed (see section: Unsuccessful and Low-Yield Substrates). This shutdown of catalysis has been observed before in other nickel-catalyzed procedures. [2,3] Nevertheless, to gain access to compound **S3**, we decided to esterify compound **7** in order to afford di-ester **2sa**.

Bis(4-nitrobenzyl)-[1,1'-bi(cyclobutane)]-3,3'-diene-2,2'-dicarboxylate (2sa)

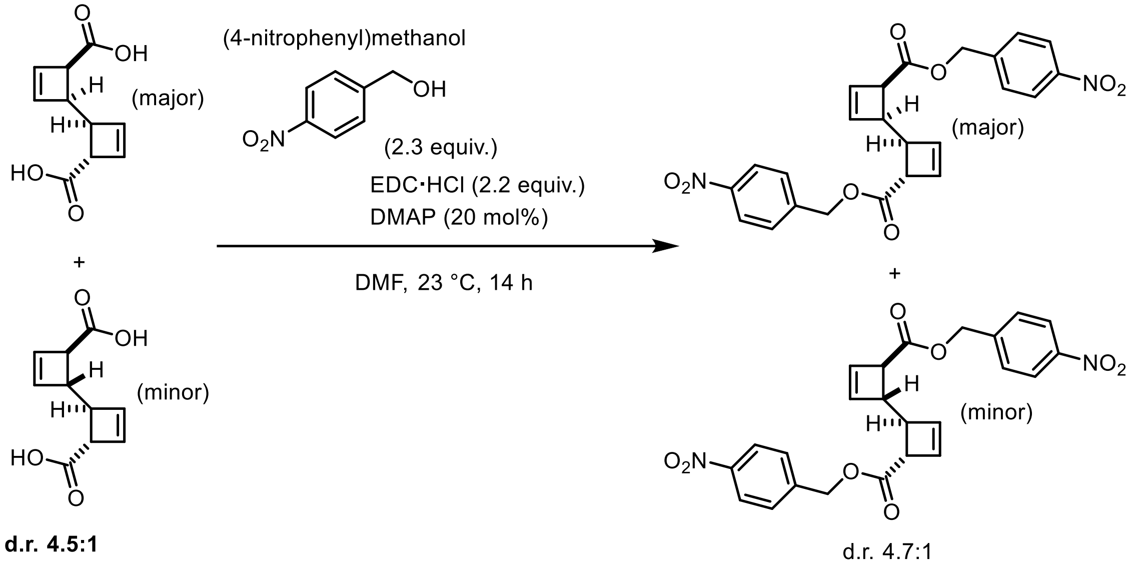

A flame-dried Schlenk flask was loaded with the diacid (34 mg, 0.18 mmol, 3.6:1 major:minor, 1.0 equiv.) and dissolved in DMF (1.8 mL, 0.1M). Then, DMAP (4.4 mg, 0.035 mmo, 0.2 equiv.) and 4-nitrophenyl methanol (62.3 mg, 0.40 mmol, 2.2 equiv.) were added. The mixture was cooled to 0°C before the addition of EDC·HCl (74 mg, 0.39 mmol, 2.1 equiv.). The resulting crude was then left to warm up to room temperature, and stirred for antoher 14 h. After that, the mixture was diluted in DCM extracted with sat. $NaHCO_3$ solution, sat. NH_4Cl solution, and brine. The organic phases were dried on $MgSO_4$, filtered and concentrated under reduced pressure. The concentrated crude was purified via column chromatography (pentane:Et_2O), affording the desired di-ester as a co-eluting 1:1.6 mixture with the benzylic alcohol (7.9 mg, 16 µmol, 9%) as an off-white solid.

^{1}H NMR (600 MHz, $CDCl_3$) δ 8.21 – 8.20 (m, 4H), 7.49 (d, *J* = 8.4 Hz, 4H), 6.31 (s, 1.7H), 6.29 – 6.26 (m, 0.3H*), 6.19 – 6.15 (m, 2H), 5.22 (s, 4H), 3.44 (s, 0.3H*), 3.39 (s, 1.7H), 3.27 (s, 0.3H*), 3.20 (s, 1.7H) ppm.

^{13}C NMR (151 MHz, $CDCl_3$) δ 172.0, 147.8, 143.3, 141.3, 140.4*, 134.8*, 134.4, 128.4, 128.3, 127.1, 124.0, 65.0, 50.0, 49.3*, 48.9, 48.1* ppm.

IR (neat) ν_{max}: 3515, 1729, 1511, 1306, 1170, 1042, 1013, 979, 926.

HRMS (ESI) m/z: $[M+Na]^+$ Calculated for $C_{24}H_{20}N_2NaO_8^+$ 487.1112; Found 487.1115.

Hint for minor isomer - HPLC measurement:

Having gained a clearer insight into the structure of the major isomer, further insight was needed to elucidate the structure of the minor isomer observed in our protocol. Therefore, we decided to measure the cyclobutene dimer **2a** (chosen for its high UV activity) using a chiral HPLC. As expected, the major isomer **2a** split into its two enantiomers, but the minor isomer did not split into two peaks. From this observation, we concluded that the minor isomer must be achiral, which fits either the *cis-cis* or *trans-*

trans meso cyclobutene dimer. However, due to observed ring opening products of **4a** upon thermal conditions (see 3.6.1) and our proposed mechanism (see manuscript), the *cis-cis* isomer seems highly unlikely.

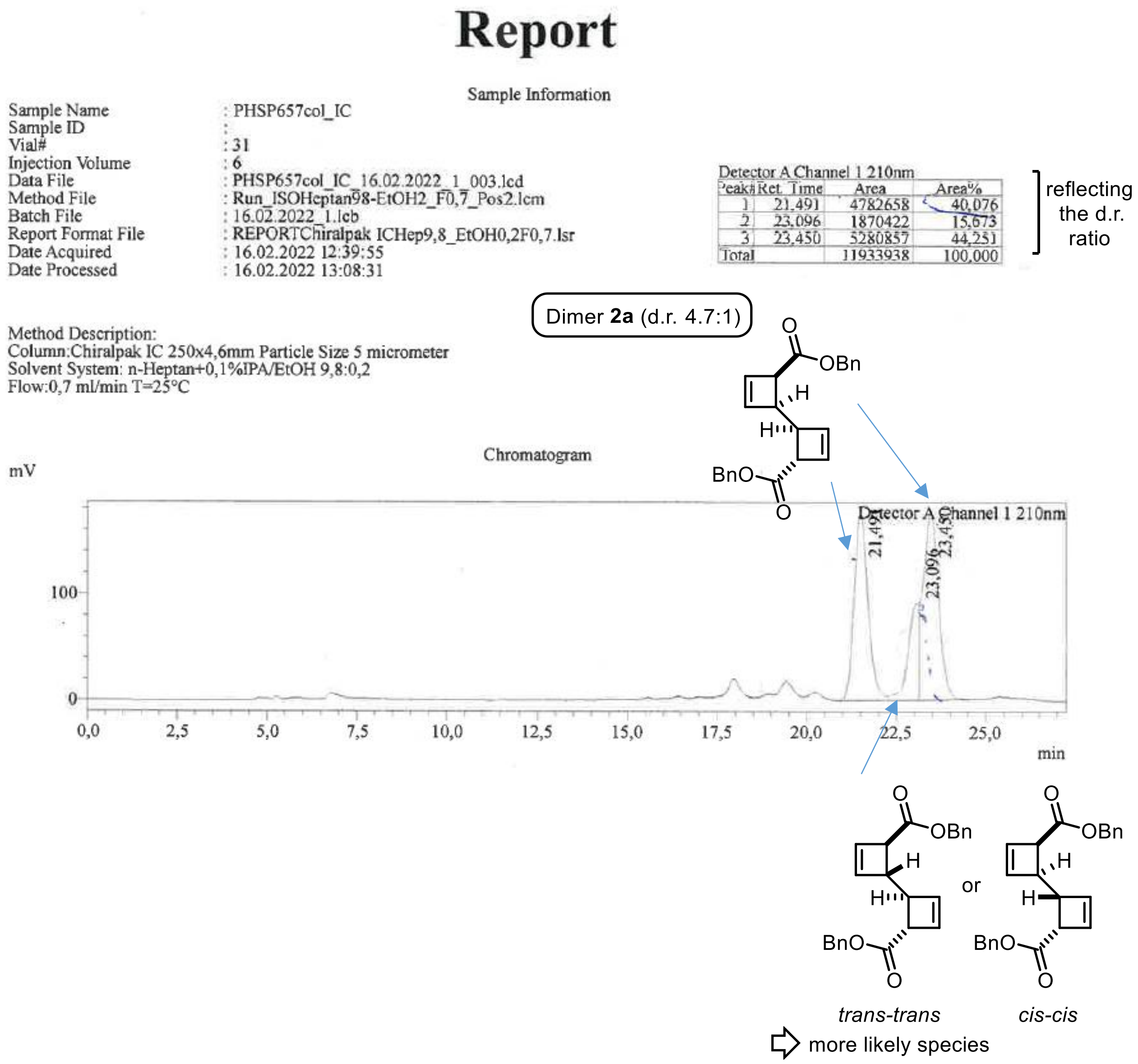

Report

Sample Information

Sample Name : PHSP657col_IC
Sample ID :
Vial# : 31
Injection Volume : 6
Data File : PHSP657col_IC_16.02.2022_1_003.lcd
Method File : Run_ISOHeptan98-EtOH2_F0,7_Pos2.lcm
Batch File : 16.02.2022_1.lcb
Report Format File : REPORTChiralpak ICHep9,8_EtOH0,2F0,7.lsr
Date Acquired : 16.02.2022 12:39:55
Date Processed : 16.02.2022 13:08:31

Detector A Channel 1 210nm

Peak#	Ret. Time	Area	Area%
1	21,491	4782658	40,076
2	23,096	1870422	15,673
3	23,450	5280857	44,251
Total		11933938	100,000

Method Description:
Column:Chiralpak IC 250x4,6mm Particle Size 5 micrometer
Solvent System: n-Heptan+0,1%IPA/EtOH 9,8:0,2
Flow:0,7 ml/min T=25°C

Chromatogram

3.9. Reductive Heterocoupling of Bromo-Cyclobutene with Alkyl Halides

3.9.1. General Procedure for Heterocoupling

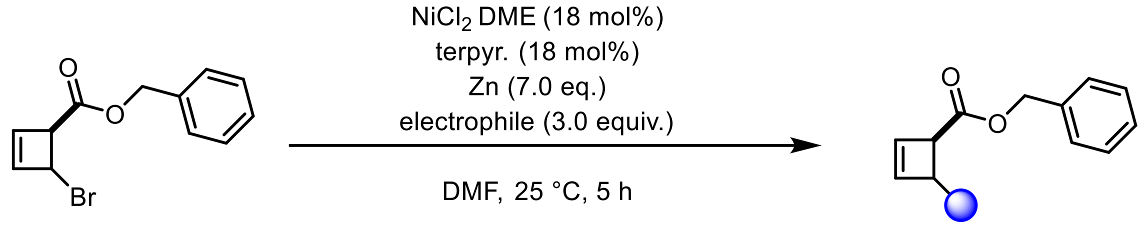

[The reaction described was performed outside the glovebox, but certain chemicals (marked with an asterisk) were stored inside the glovebox and removed before use.]*

A flame-dried Schlenk flask under Argon was loaded with nickel(II) chloride ethylene glycol dimethyl ether complex* (4.0 mg, 0.018 mmol, 0.18 equiv.), terpyridine (4.2 mg, 0.018 mmol, 0.18 equiv.), and zinc* (45.8 mg, 0.7 mmol, 7.0 equiv.). DMF (0.8 mL, 0.125M) was added and the mixture was stirred for 5 min before the alkyl halide (3.0 equiv.) was added, followed immediately by the cyclobutene ester (0.1mmol, 1.0 equiv.). The reaction was stirred for 5 h. Then, the crude material was transferred to a separatory funnel using Et_2O and the organic phase was washed with water/brine mixture (1:1, 5x). Subsequently, the organic phase was dried over anhydrous $MgSO_4$, and the solvent removed under reduced pressure. The crude material was purified by column chromatography (SiO_2, heptanes/EtOAc).

Results for different alkyl halides:

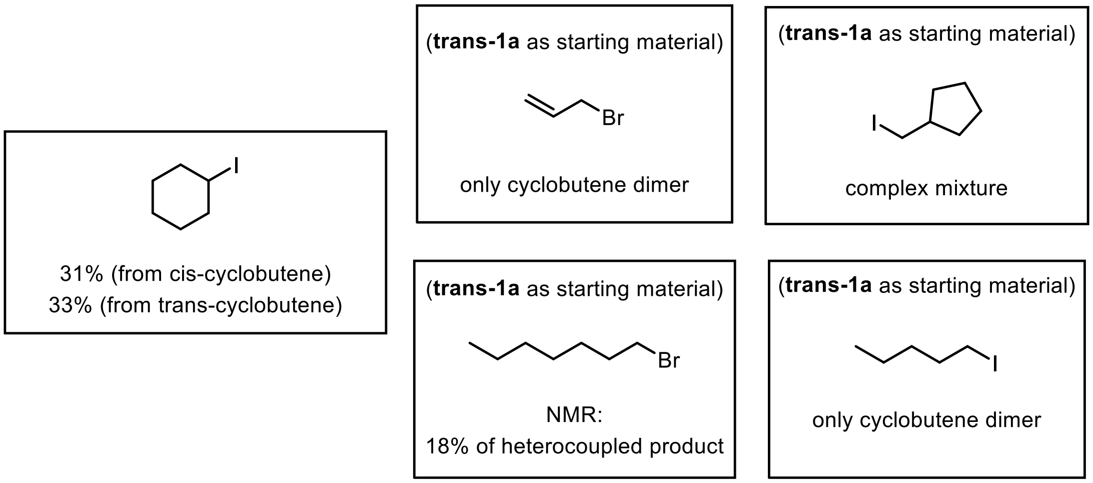

Trans-benzyl-4-cyclohexylcyclobut-2-ene-1-carboxylate (17)

Following the general procedure using *cis*-**1a** as starting material (0.1 mmol), the titled compound was obtained as a colorless oil (8.2 mg, 31 µmol, 31%). Using *trans*-**1a** the same product was obtained in a comparable yield (8.9 mg, 33 µmol, 33%).

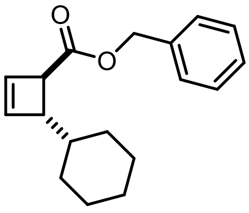

^{1}H NMR (400 MHz, $CDCl_3$): δ 7.37 – 7.31 (m, 5H), 6.36 – 6.32 (m, 1H), 6.11 (d, *J* = 2.8 Hz, 1H), 5.13 (d, *J* = 1.8 Hz, 2H), 3.33 (s, 1H), 2.73 (d, *J* = 8.9 Hz, 1H), 1.86 – 1.78 (m, 1H), 1.77 – 1.63 (m, 5H), 1.37 – 1.17 (m, 5H), 1.02 – 0.93 (m, 2H) ppm.

^{13}C NMR (101 MHz, $CDCl_3$): δ 173.4, 142.1, 136.4, 133.7, 128.7, 128.2, 128.1, 66.3, 54.5, 49.9, 41.8, 31.0, 30.5, 26.5, 26.1 (2C) ppm.

IR (neat) ν_{max}: 2912, 2850, 1730, 1448, 1246, 1212, 1153, 1016, 1001, 732, 695

HRMS (ESI) m/z: $[M+Na]^+$ Calculated for $C_{18}H_{18}O_2Na^+$ 293.1512; Found 293.1511.

Benzyl (2E,4E)-5-cyclohexylpenta-2,4-dienoate (S4)

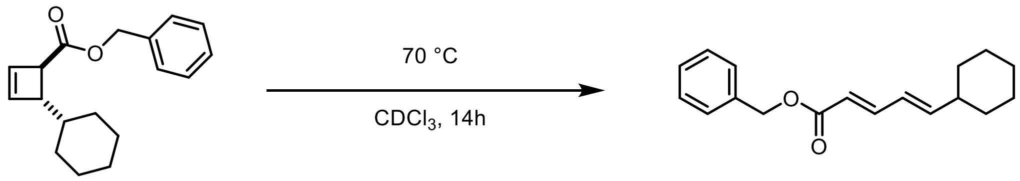

Compound **17** (3.8 mg, 14 µmol, 1.0 equiv.) in chloroform (1 ml, 14 mM) was heated to 70 °C for 14h. After evaporation of the solvent, the crude material was passed through a short silica plug to provide the titled compound as yellowish oil (3.2 mg, 12 µmol, 85%).

^{1}H NMR (400 MHz, $CDCl_3$): δ 7.37 – 7.32 (m, , 6H), 6.14 (dd, *J* = 15.3, 9.8 Hz, 1H), 6.07 (dd, *J* = 15.2, 6.1 Hz, 1H), 5.84 (d, *J* = 15.4 Hz, 1H), 5.19 (s, 2H), 2.13 – 2.04 (m, 1H), 1.77 – 1.72 (m, 4H), 1.65 – 1.62 (m, 1H), 1.33 – 1.25 (m, 5H) ppm.

^{13}C NMR (101 MHz, $CDCl_3$): δ 167.2, 150.7, 146.2, 136.4, 128.7, 128.3, 128.3, 126.0, 119.0, 66.2, 41.3, 32.4, 26.1, 25.9 ppm.

All NMR was in good accordance with the literature.[4]

3.9.2. Proposed Mechansim

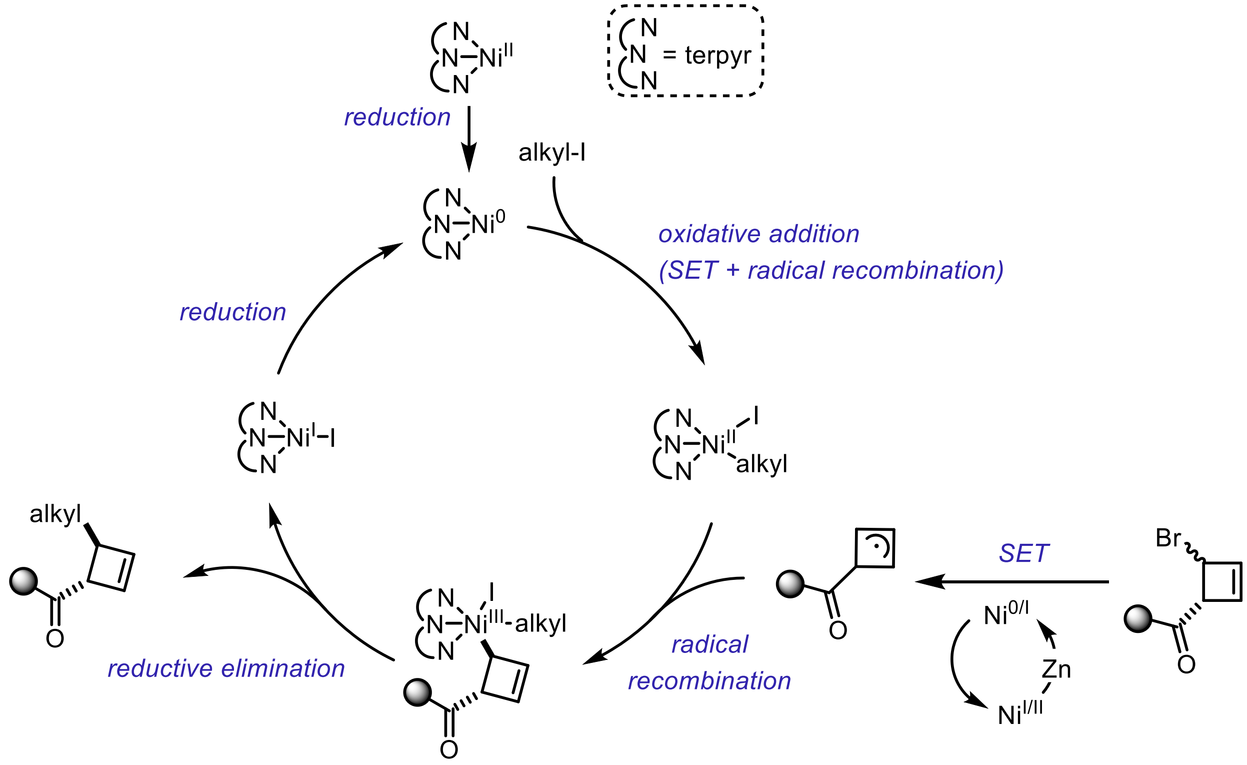

4. References

(1) Souris, C.; Frébault, F.; Patel, A.; Audisio, D.; Houk, K. N.; Maulide, N. Stereoselective Synthesis of Dienyl-Carboxylate Building Blocks: Formal Synthesis of Inthomycin C. *Org. Lett.* **2013**, *15* (13), 3242–3245.

(2) Berman, R. S.; Kochi, J. K. Kinetics and Mechanism of Oxygen Atom Transfer from Nitro Compounds Mediated by Nickel(0) Complexes. *Inorg. Chem.* **1980**, *19* (1), 248–254.

(3) Apolinar, O.; Tran, V. T.; Kim, N.; Schmidt, M. A.; Derosa, J.; Engle, K. M. Sulfonamide Directivity Enables Ni-Catalyzed 1,2-Diarylation of Diverse Alkenyl Amines. *ACS Catal.* **2020**, *10* (23), 14234–14239.

(4) Luo, Y.; Roy, I. D.; Madec, A. G. E.; Lam, H. W. Enantioselective Synthesis of Allylboronates and Allylic Alcohols by Copper-Catalyzed 1,6-Boration. *Angew. Chem.* **2014**, *53* (16), 4186–4190.

5. NMR Spectra

^{1}H NMR (400 MHz, $CDCl_3$)

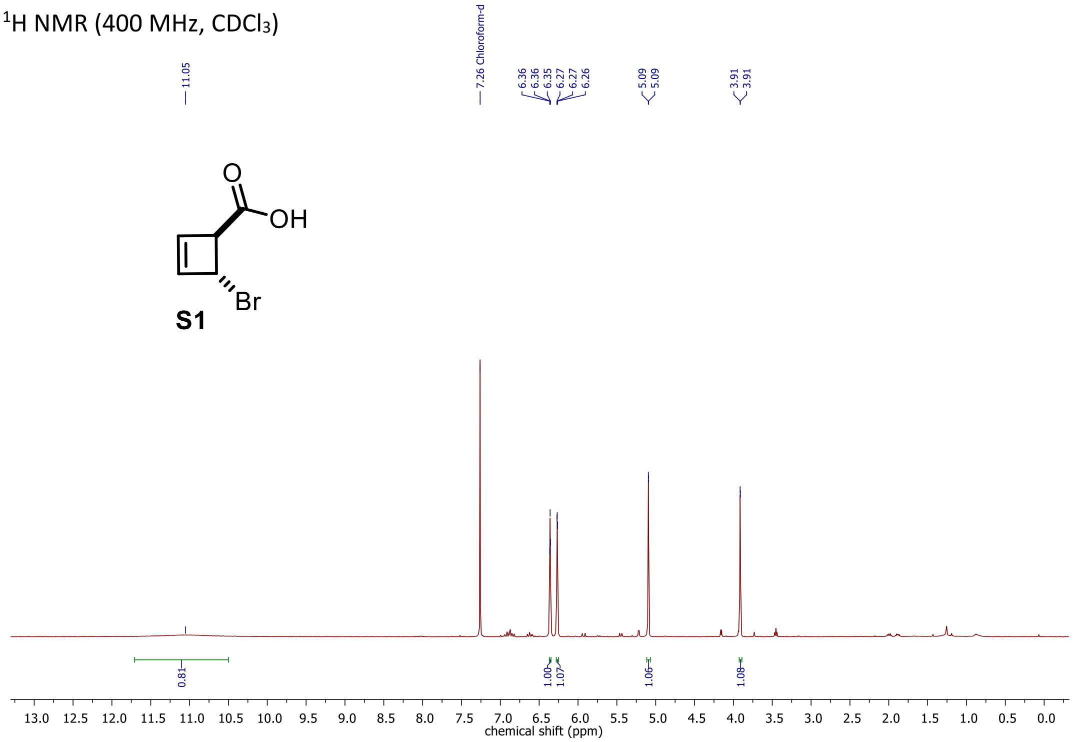

^{13}C NMR (101 MHz, $CDCl_3$)

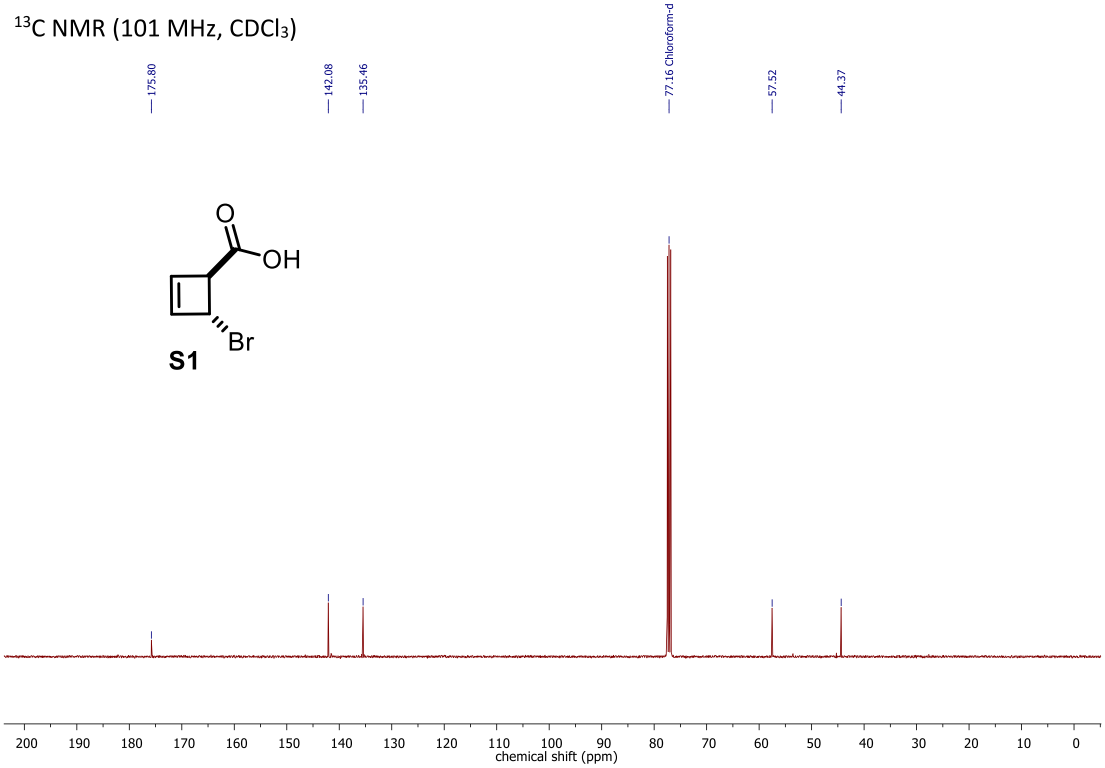

^1H NMR (600 MHz, $CDCl_3$)

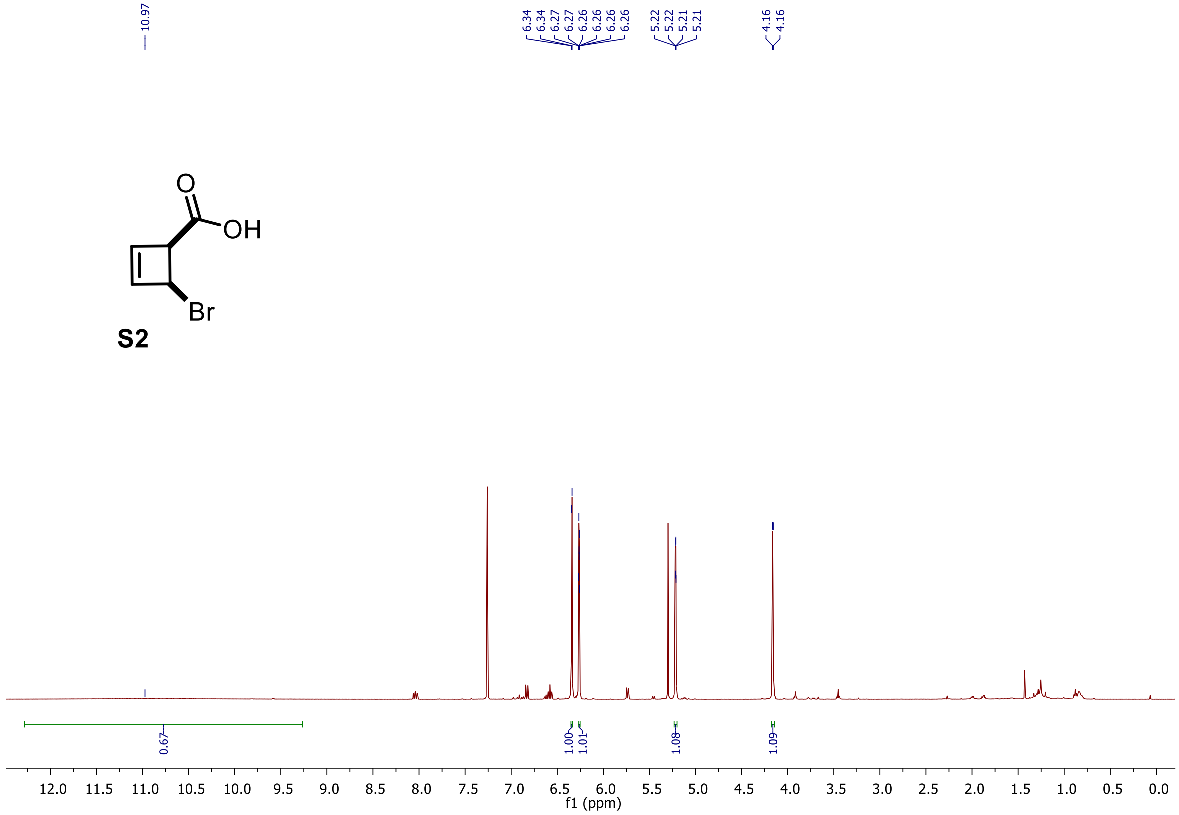

^{13}C NMR (151 MHz, $CDCl_3$)

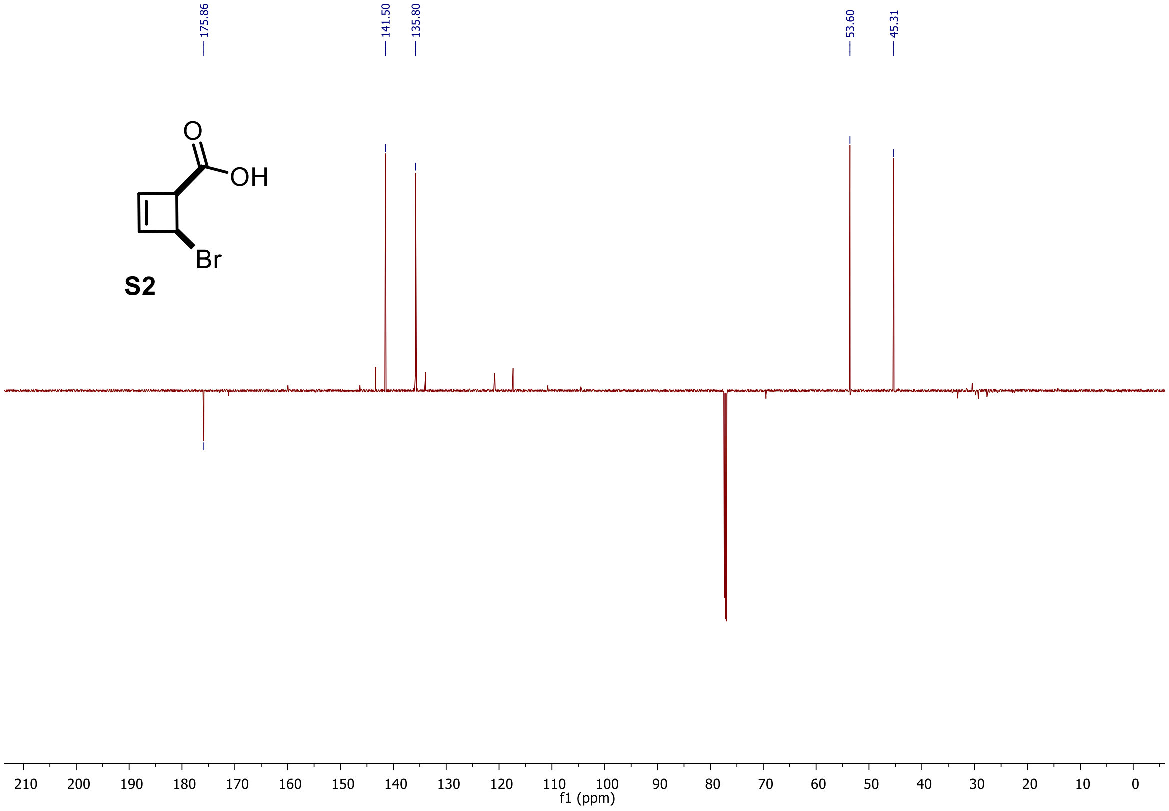

^{1}H NMR (400 MHz, $CDCl_3$)

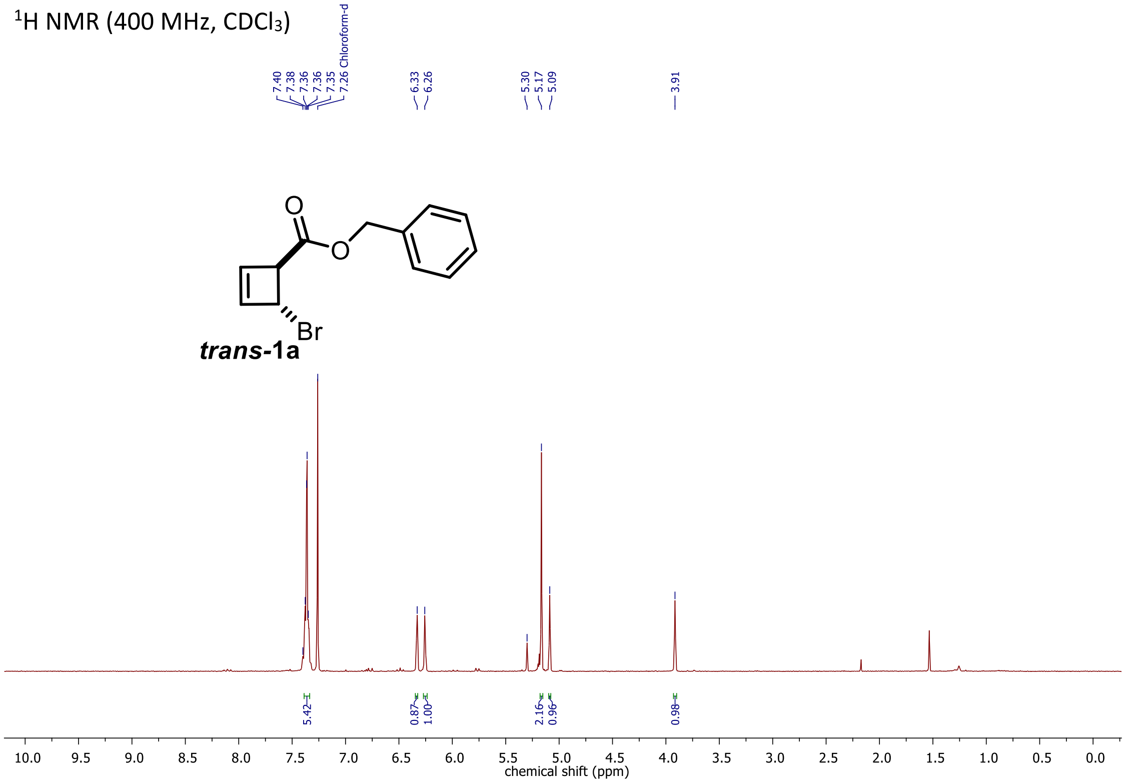

^{13}C NMR (101 MHz, $CDCl_3$)

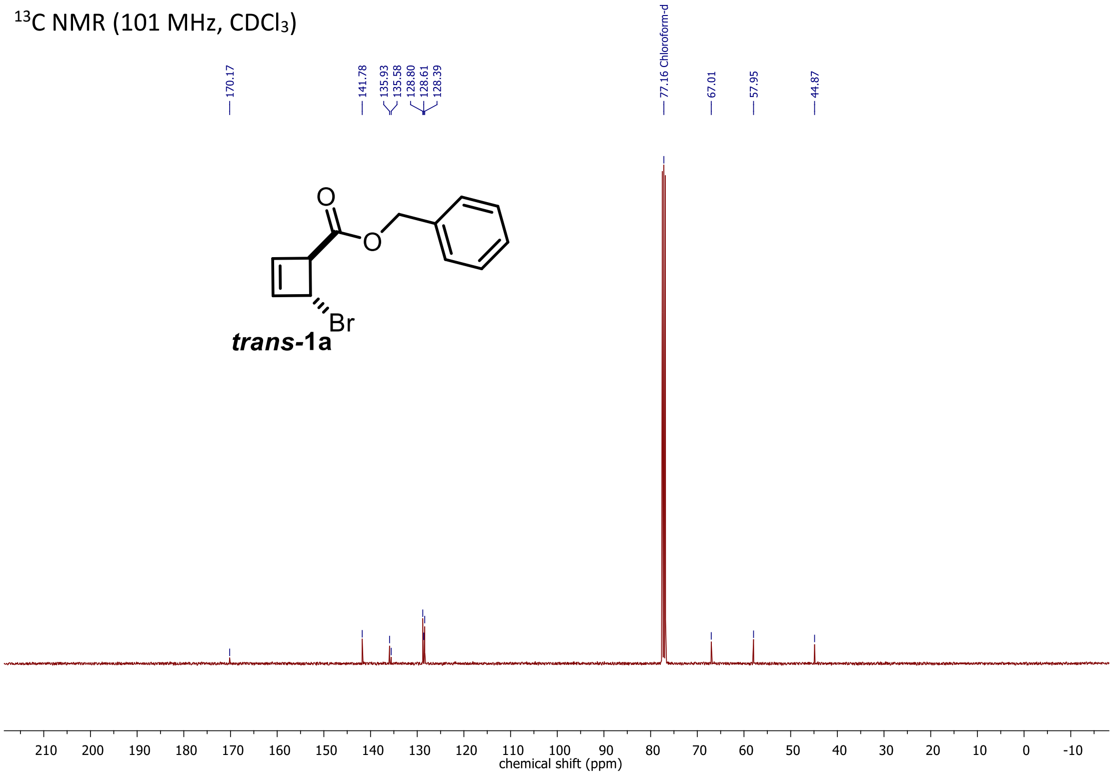

^{1}H NMR (400 MHz, $CDCl_3$)

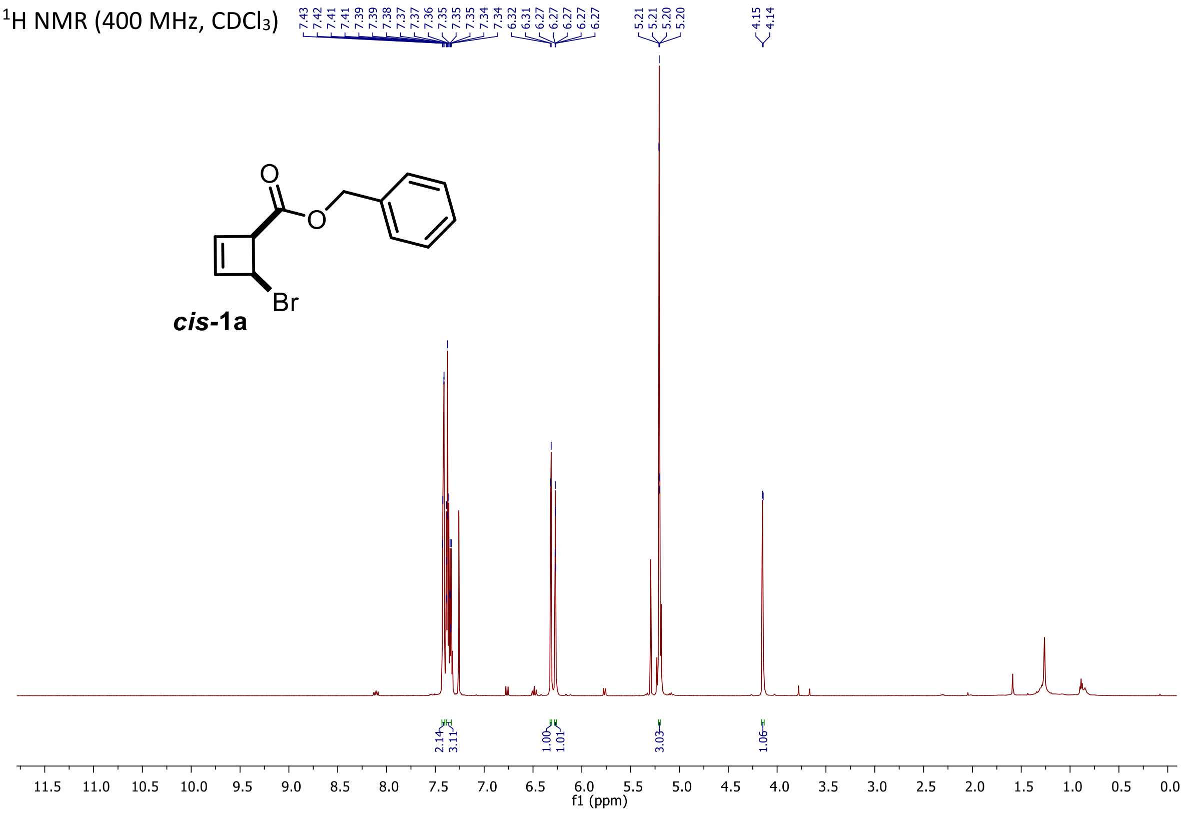

^{13}C NMR (101 MHz, $CDCl_3$)

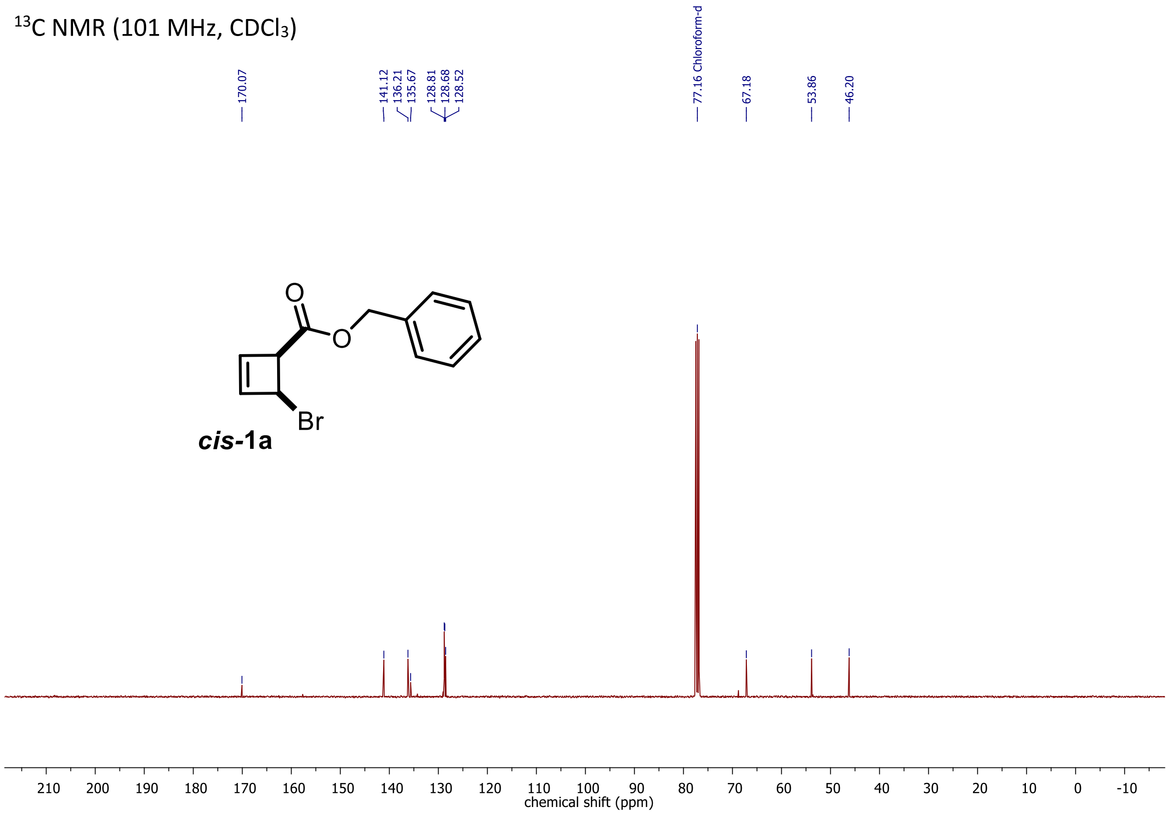

^{1}H NMR (400 MHz, $CDCl_3$)

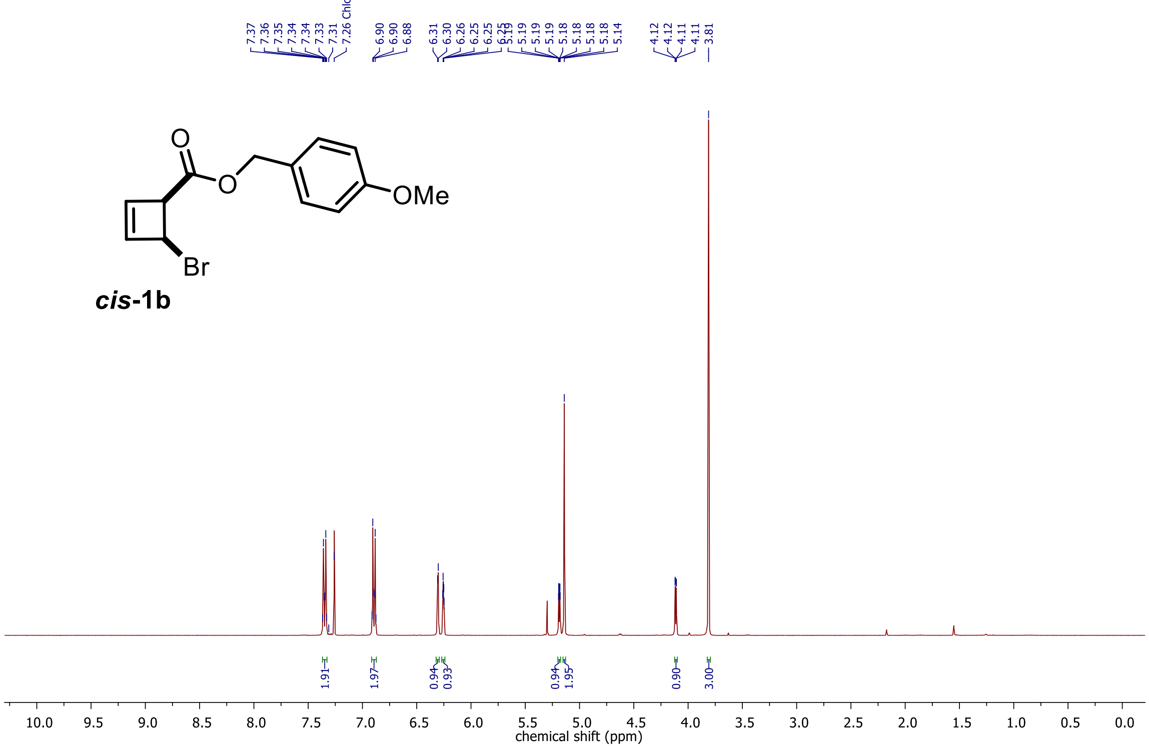

^{13}C NMR (101 MHz, $CDCl_3$)

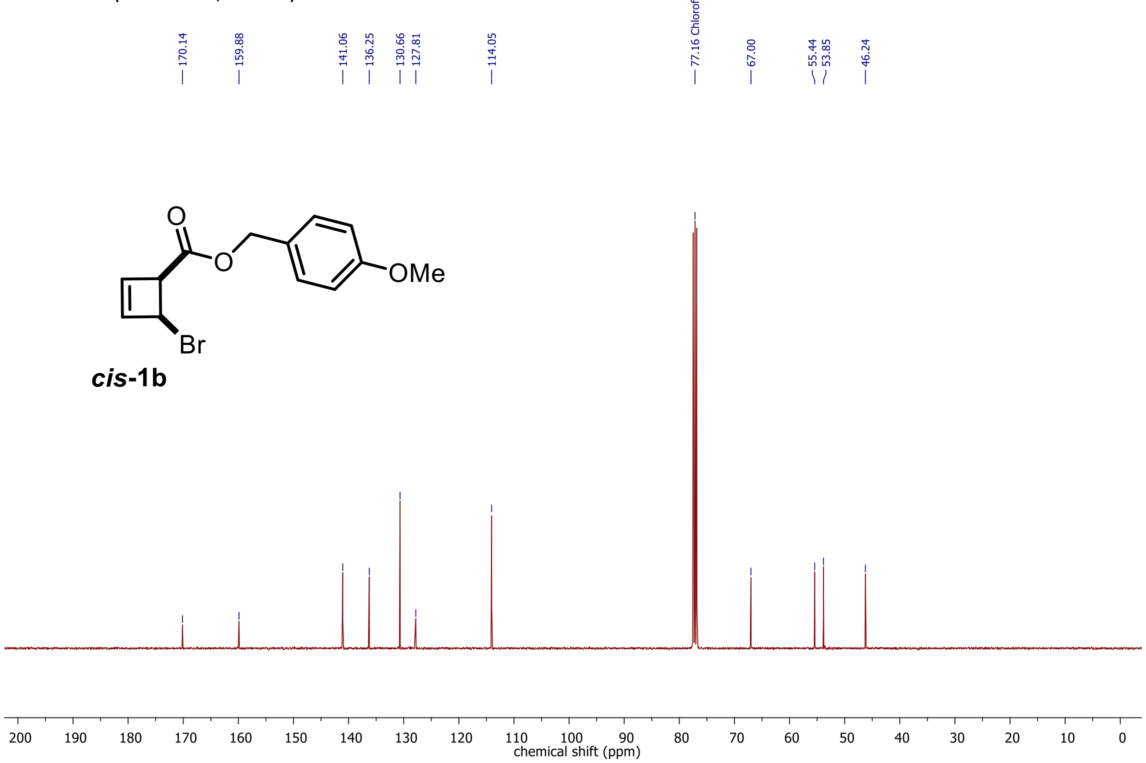

^{1}H NMR (600 MHz, $CDCl_3$)

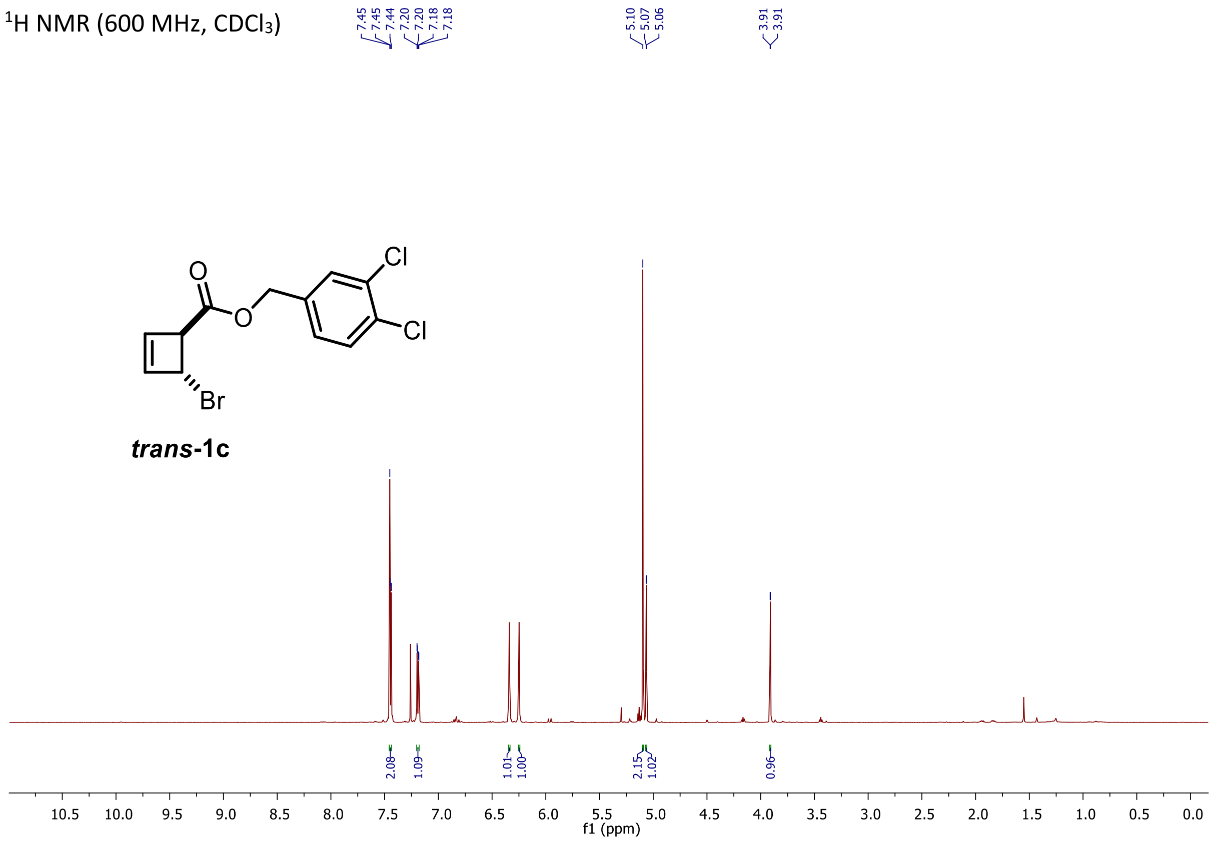

^{13}C NMR (151 MHz, $CDCl_3$)

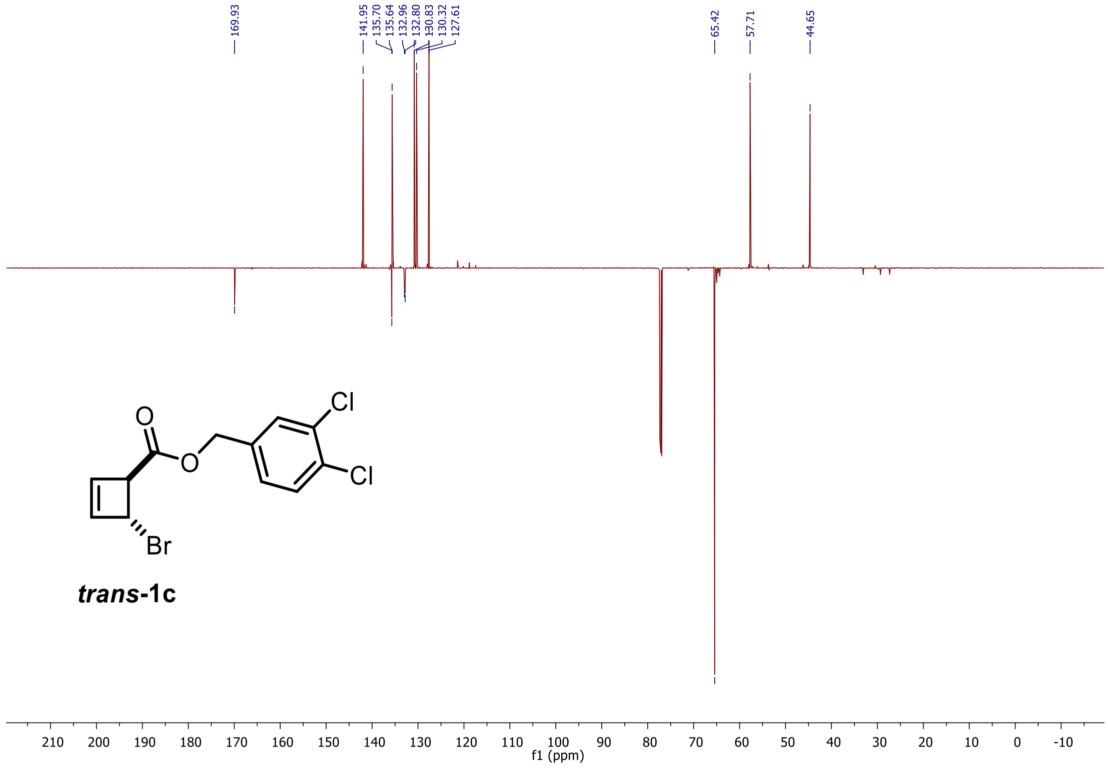

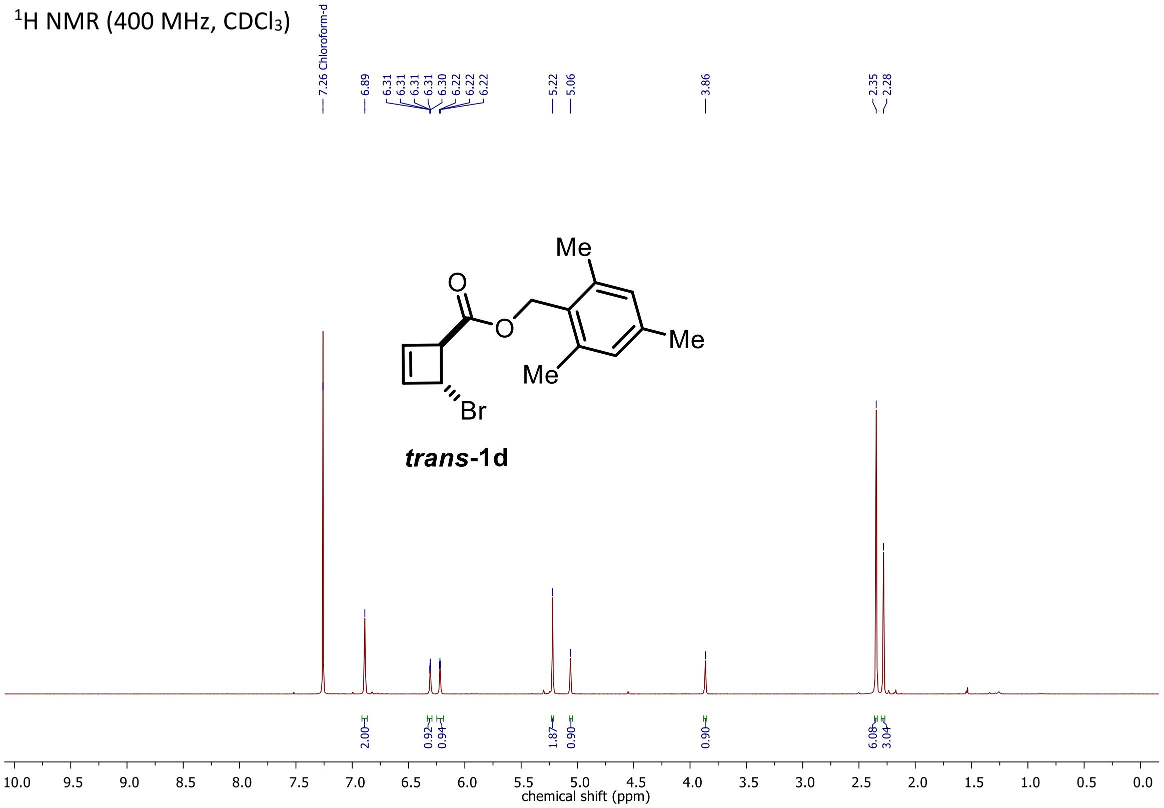

1H NMR (400 MHz, CDCl3)
7.26 Chloroform-d
6.89
6.31
6.31
6.31
6.31
6.30
6.22
6.22
6.22
6.22
5.22
5.06
3.86
2.35
2.28
O
O
Me
Me
Me
Br
trans-1d
2.00
0.92
0.94
1.87
0.90
0.90
6.08
3.04
10.0 9.5 9.0 8.5 8.0 7.5 7.0 6.5 6.0 5.5 5.0 4.5 4.0 3.5 3.0 2.5 2.0 1.5 1.0 0.5 0.0
chemical shift (ppm)

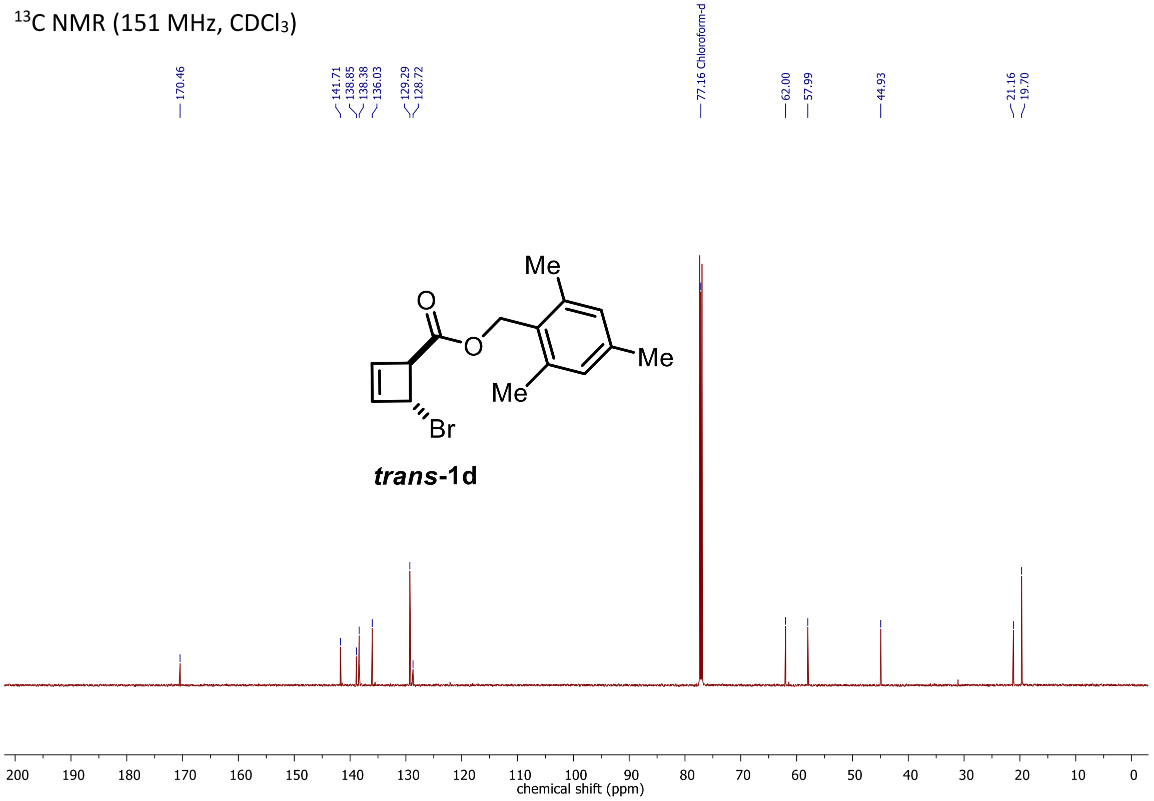

13C NMR (151 MHz, CDCl3)
170.46
141.71
138.85
138.38
136.03
129.29
128.72
77.16 Chloroform-d
62.00
57.99
44.93
21.16
19.70
O
O
Me
Me
Me
Br
trans-1d
200 190 180 170 160 150 140 130 120 110 100 90 80 70 60 50 40 30 20 10 0
chemical shift (ppm)

^1H NMR (400 MHz, $CDCl_3$)

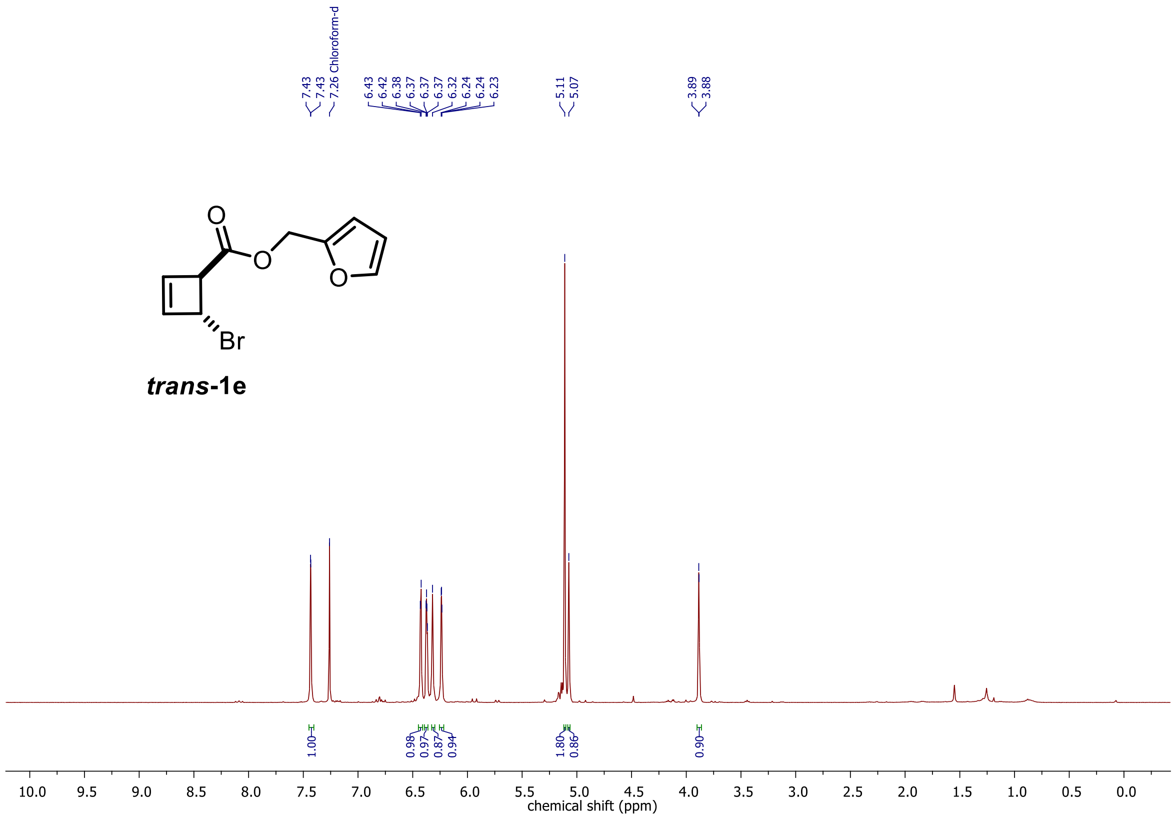

^{13}C NMR (101 MHz, $CDCl_3$)

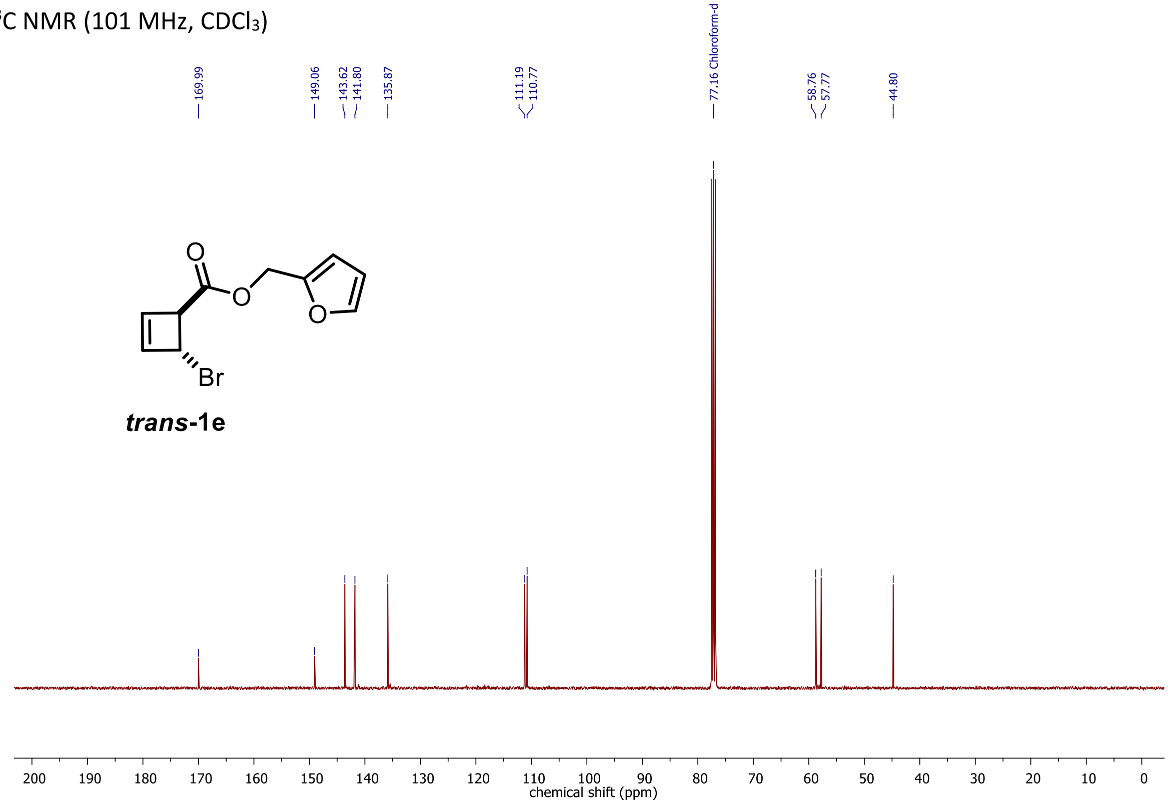

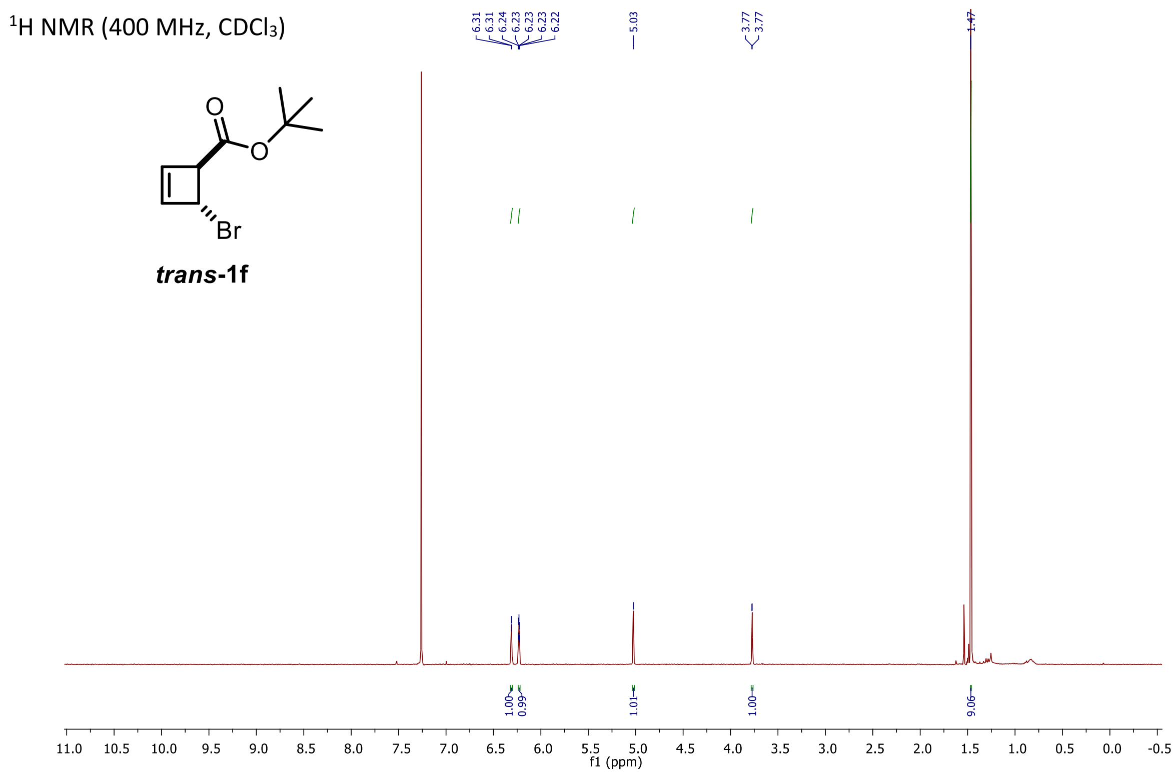
^{1}H NMR (400 MHz, CDCl$_3$)
trans-1f
6.31
6.31
6.24
6.23
6.23
6.23
6.22
5.03
3.77
3.77
1.47
1.00
0.99
1.01
1.00
9.06
f1 (ppm)

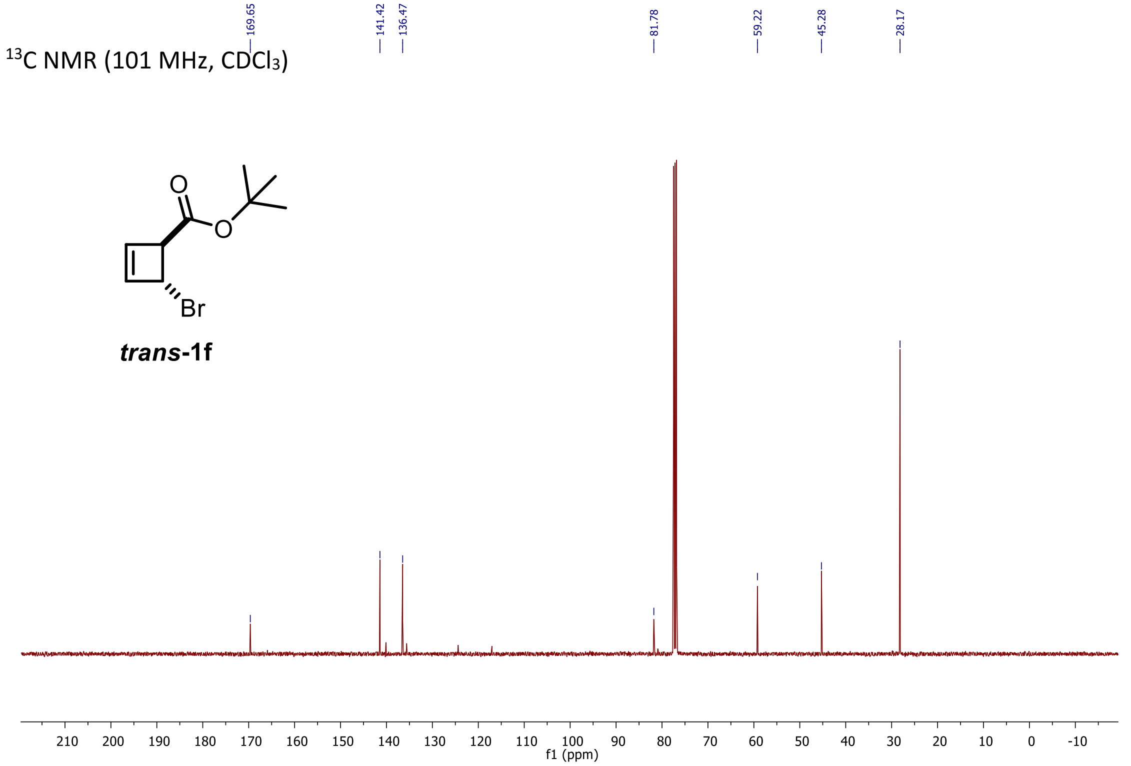
^{13}C NMR (101 MHz, CDCl$_3$)
trans-1f
169.65
141.42
136.47
81.78
59.22
45.28
28.17
f1 (ppm)

^{1}H NMR (600 MHz, $CDCl_3$)

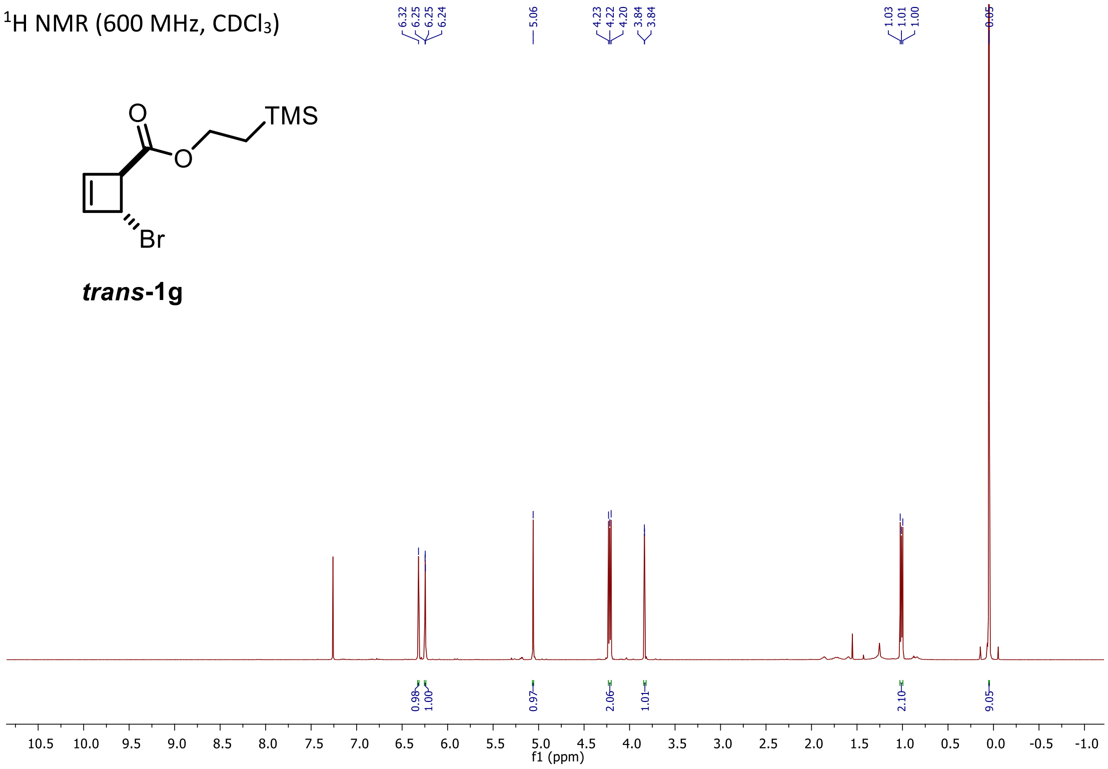

^{13}C NMR (151 MHz, $CDCl_3$)

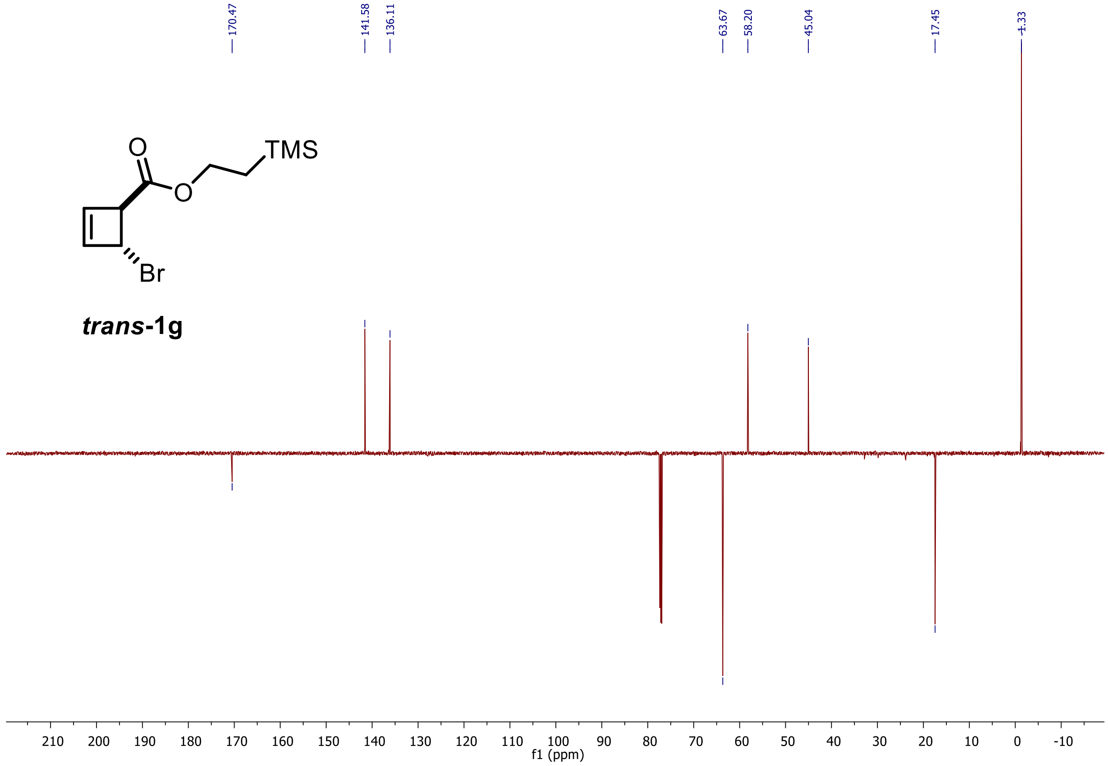

^{1}H NMR (600 MHz, $CDCl_3$)

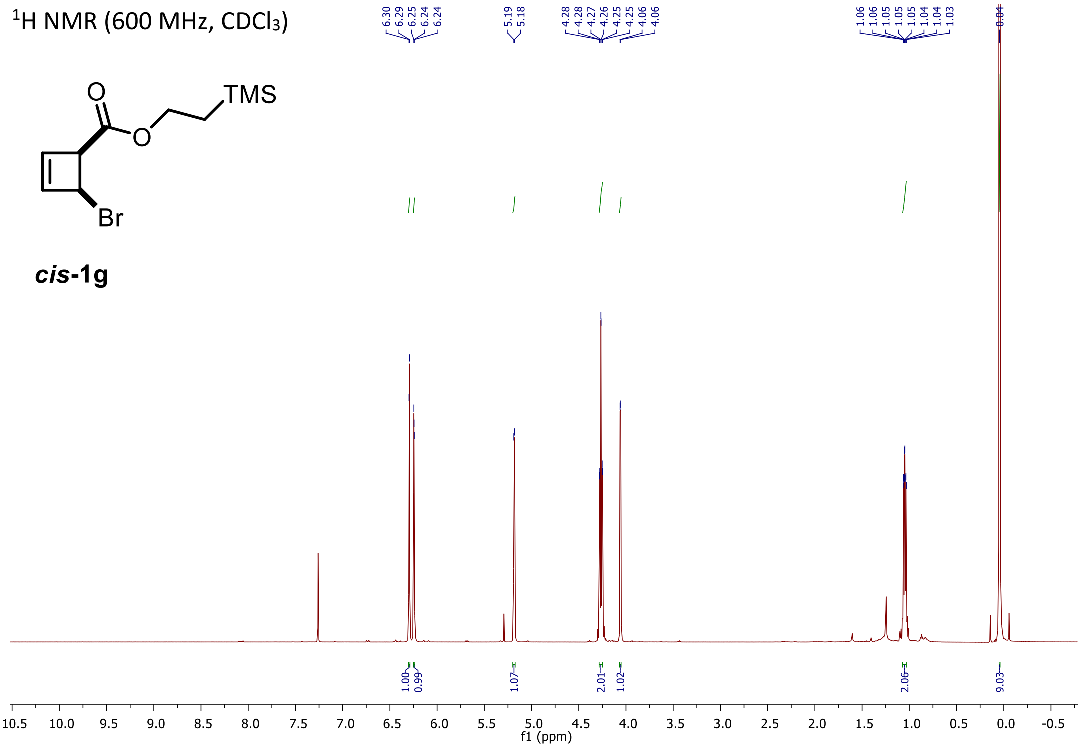

^{13}C NMR (151 MHz, $CDCl_3$)

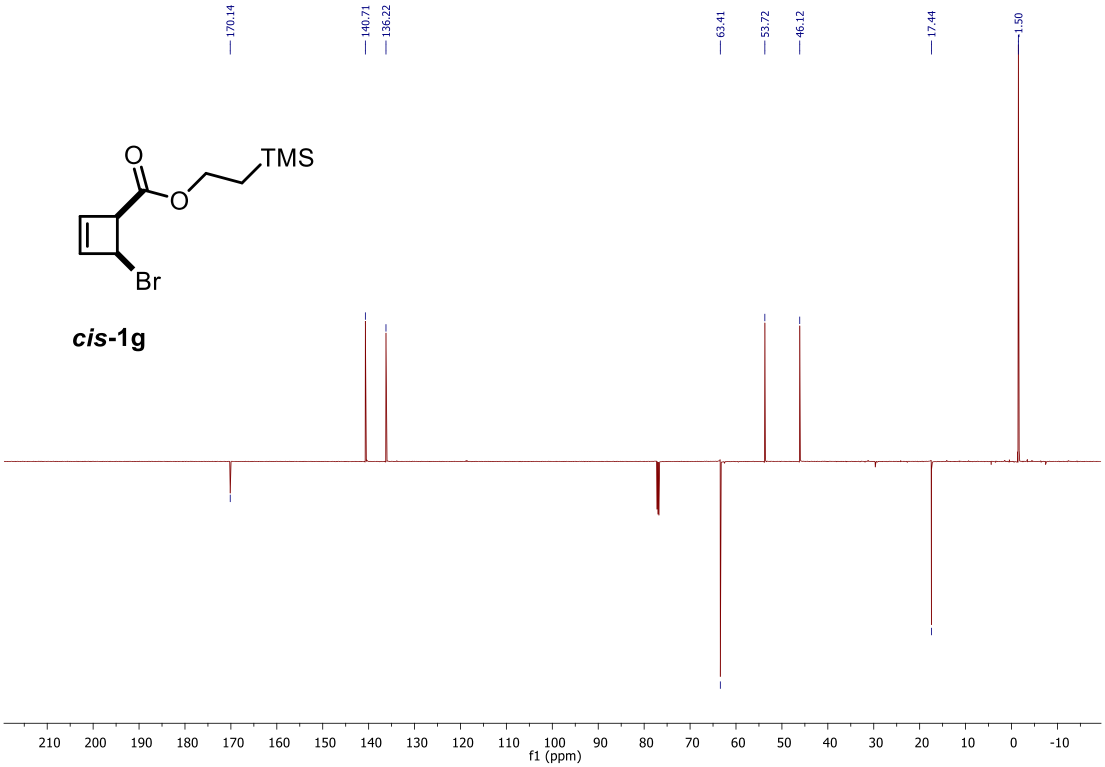

^1H NMR (400 MHz, $CDCl_3$)

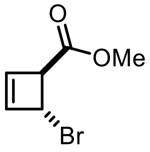

***trans*-1h**

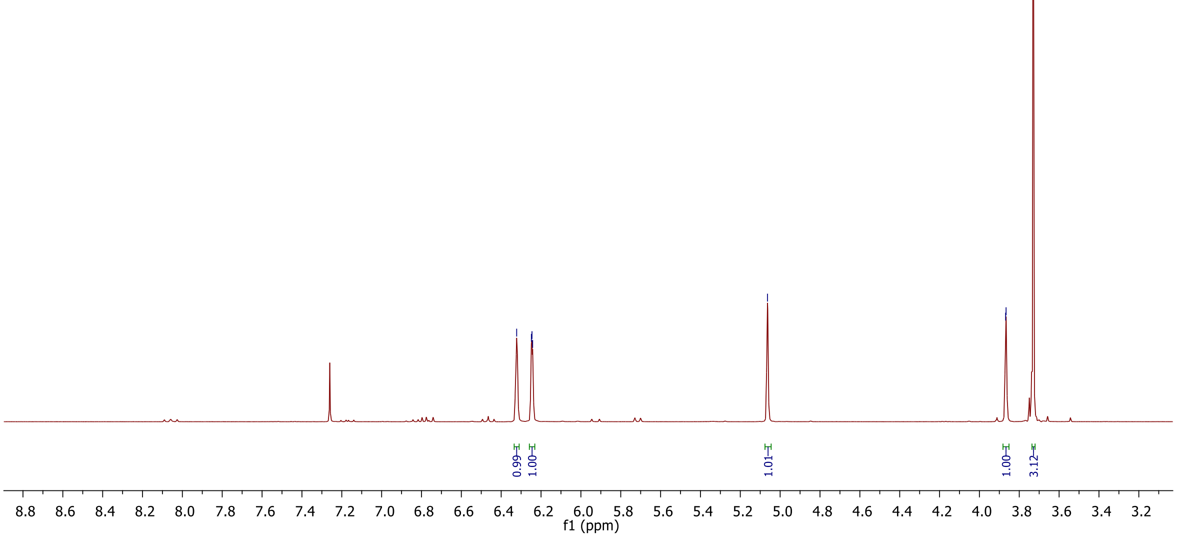

^{1}H NMR (600 MHz, $CDCl_3$)

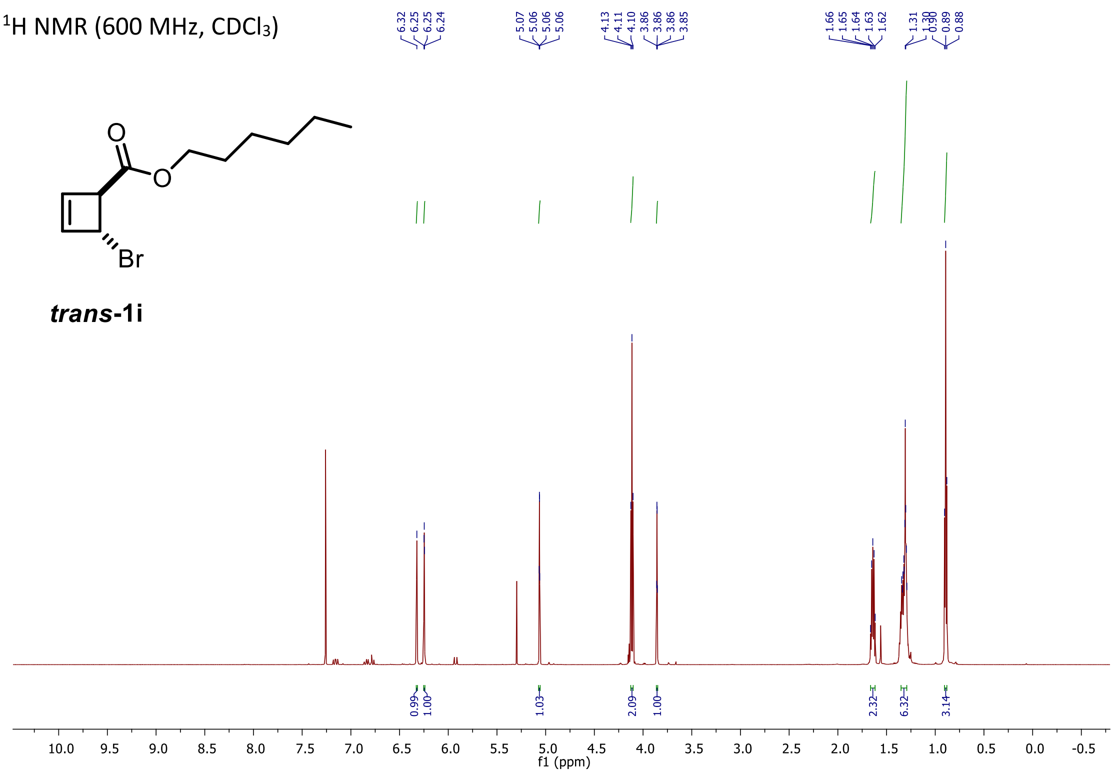

^{13}C NMR (151 MHz, $CDCl_3$)

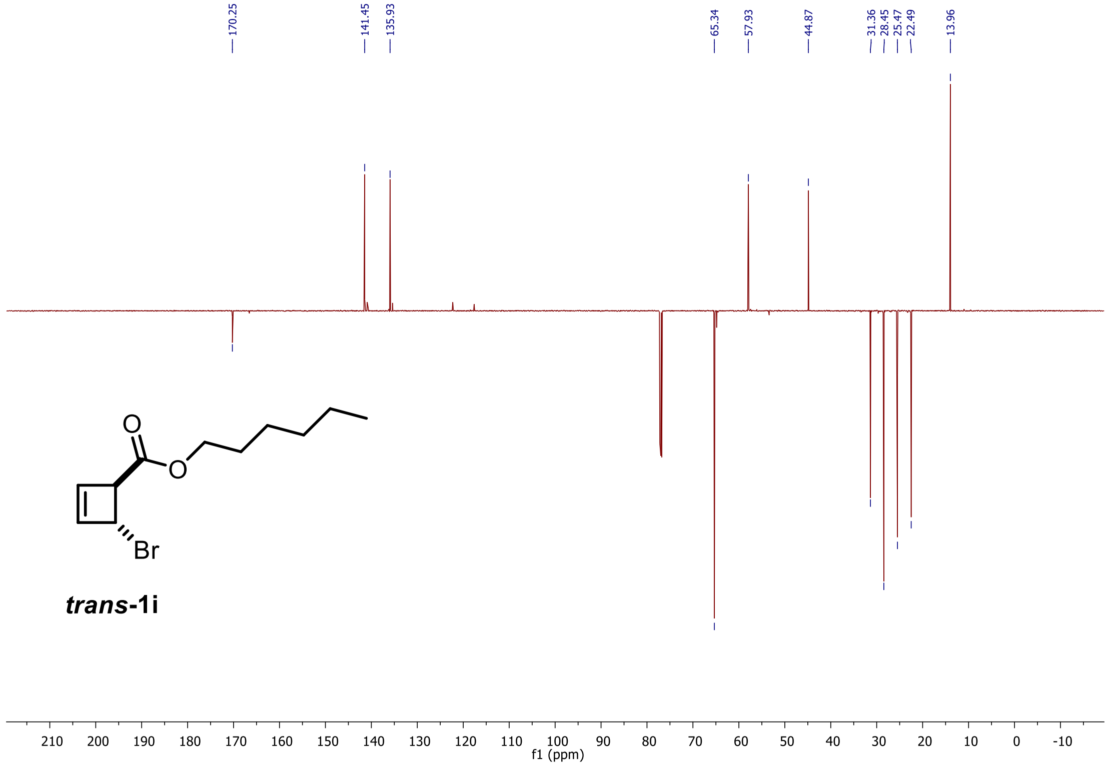

^{1}H NMR (400 MHz, $CDCl_3$)

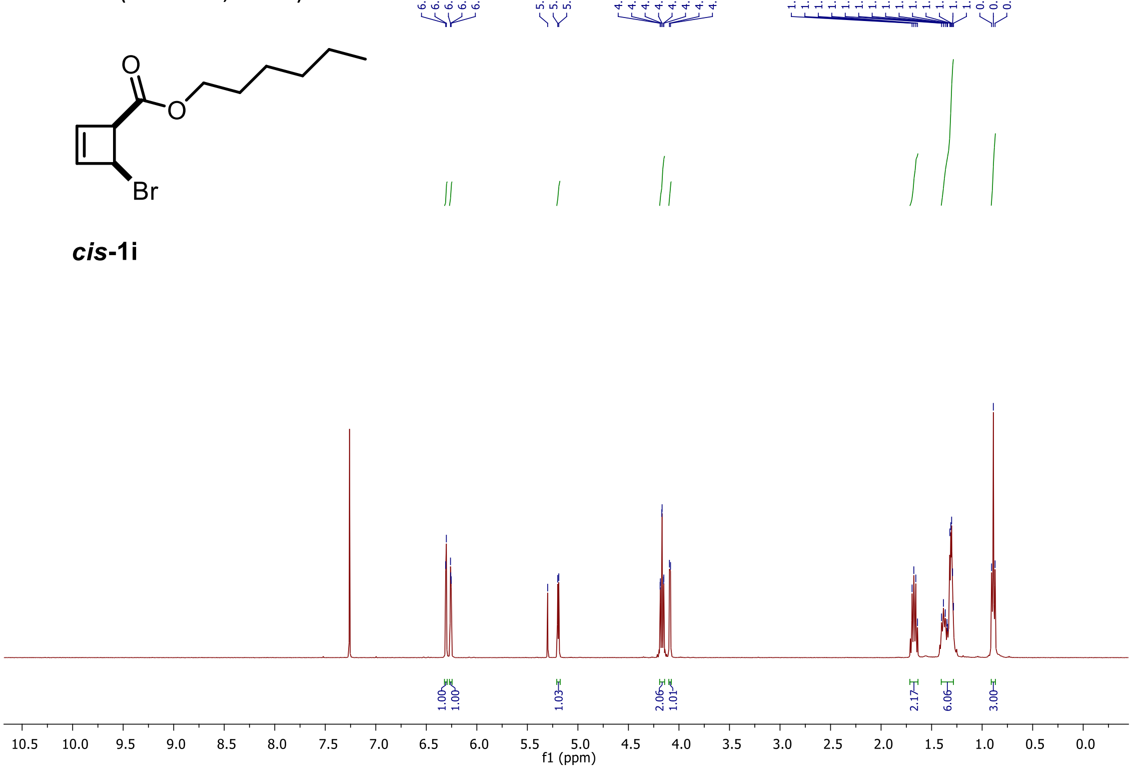

***cis*-1i**

^{13}C NMR (101 MHz, $CDCl_3$)

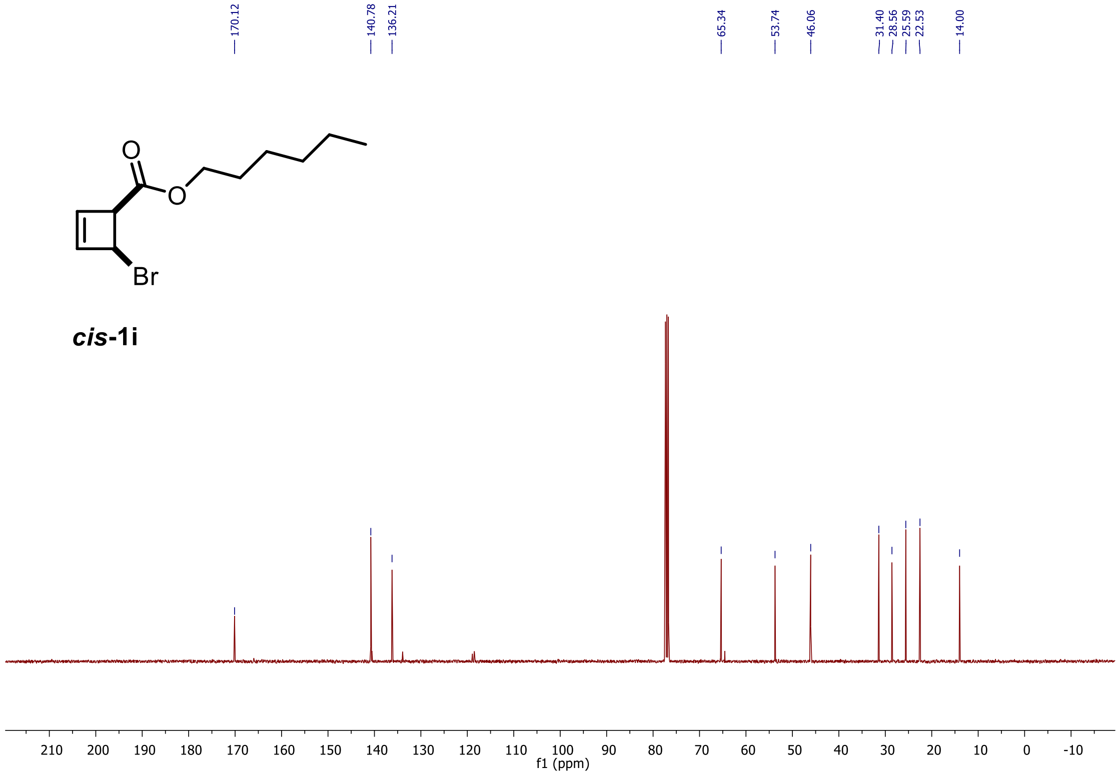

***cis*-1i**

^{1}H NMR (600 MHz, $CDCl_3$)

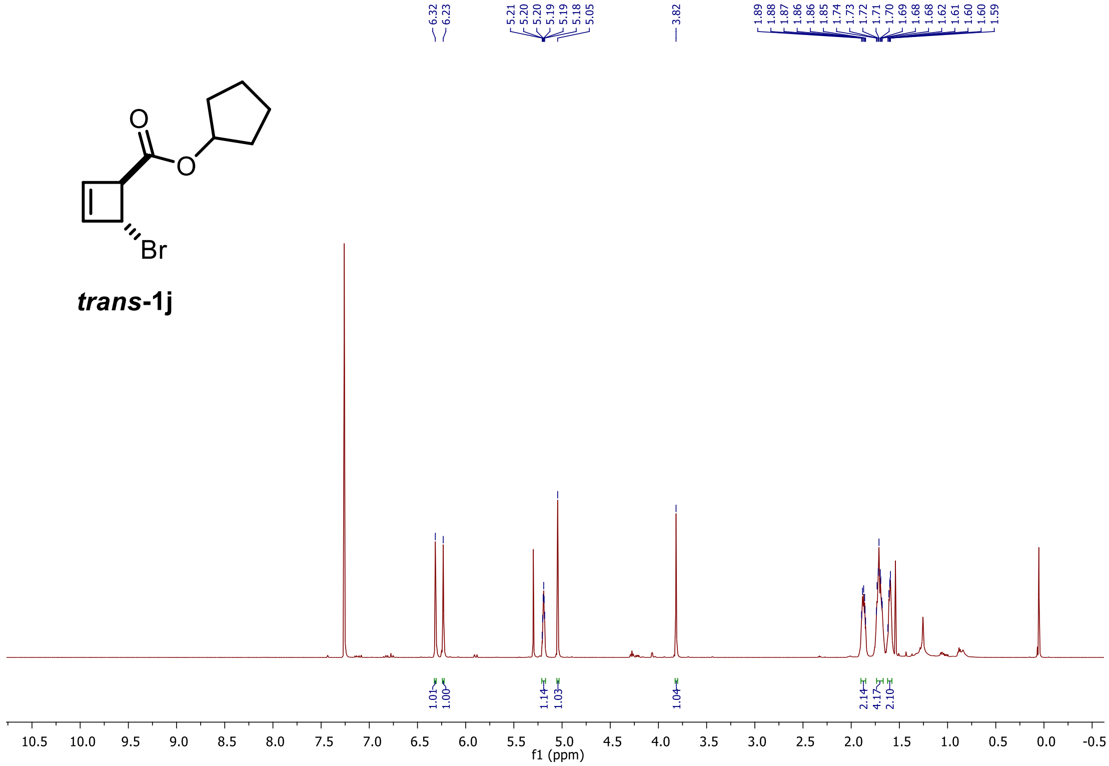

trans-**1j**

^{13}C NMR (151 MHz, $CDCl_3$)

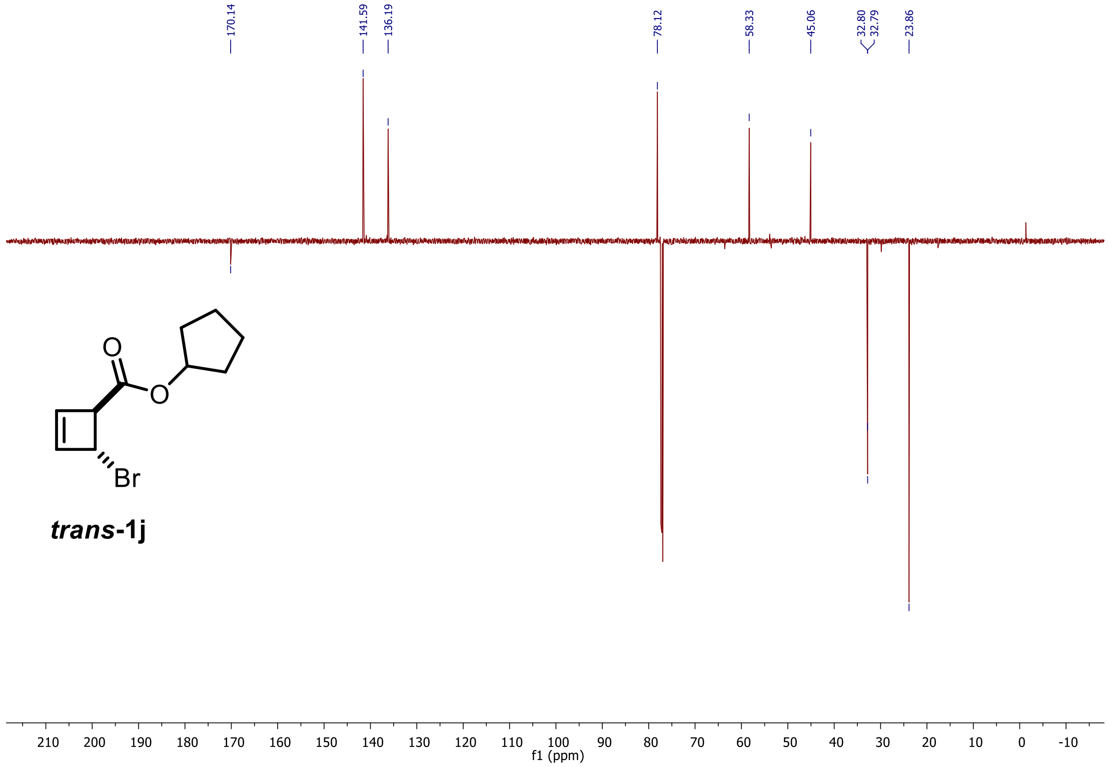

trans-**1j**

^{1}H NMR (600 MHz, $CDCl_3$)

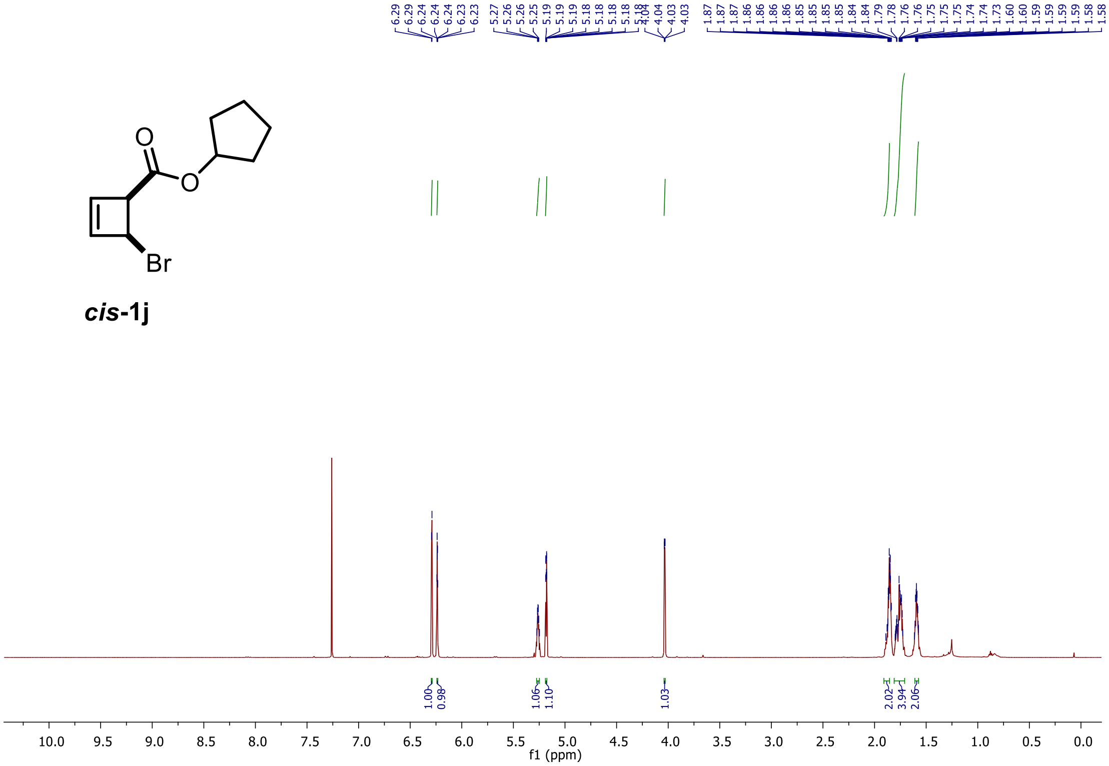

^{13}C NMR (151 MHz, $CDCl_3$)

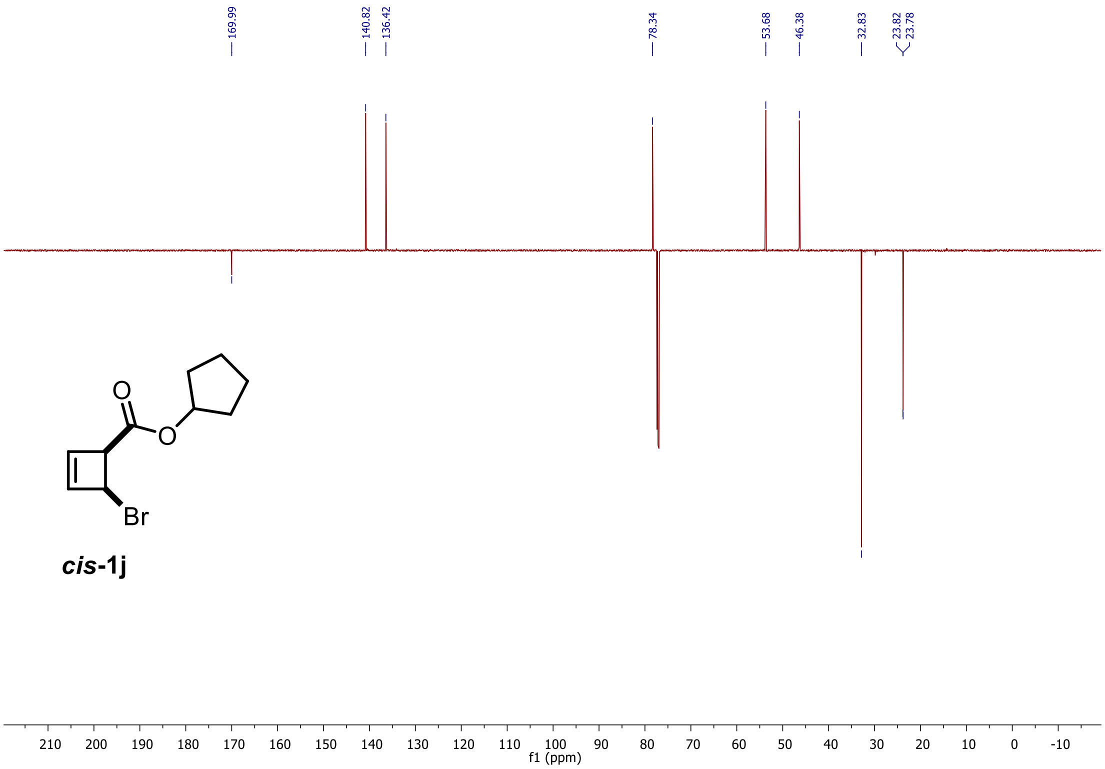

^{1}H NMR (400 MHz, $CDCl_3$)

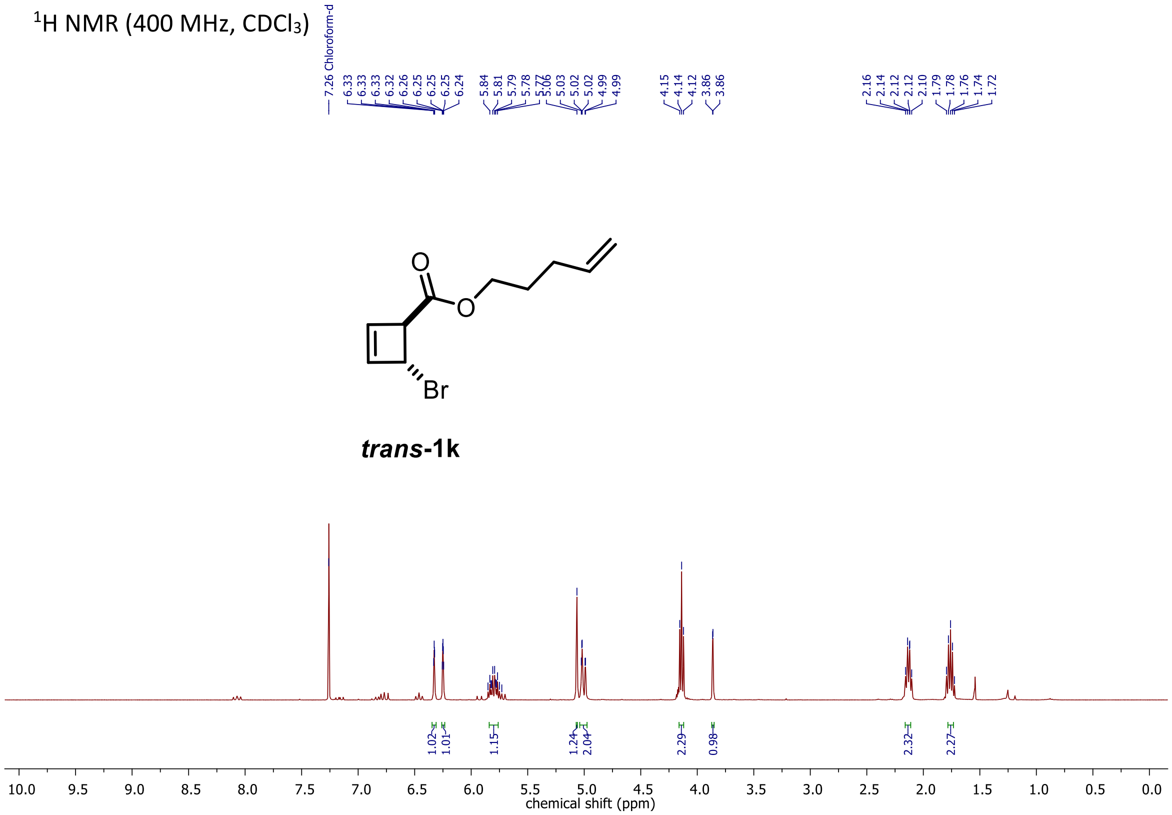

^{13}C NMR (101 MHz, $CDCl_3$)

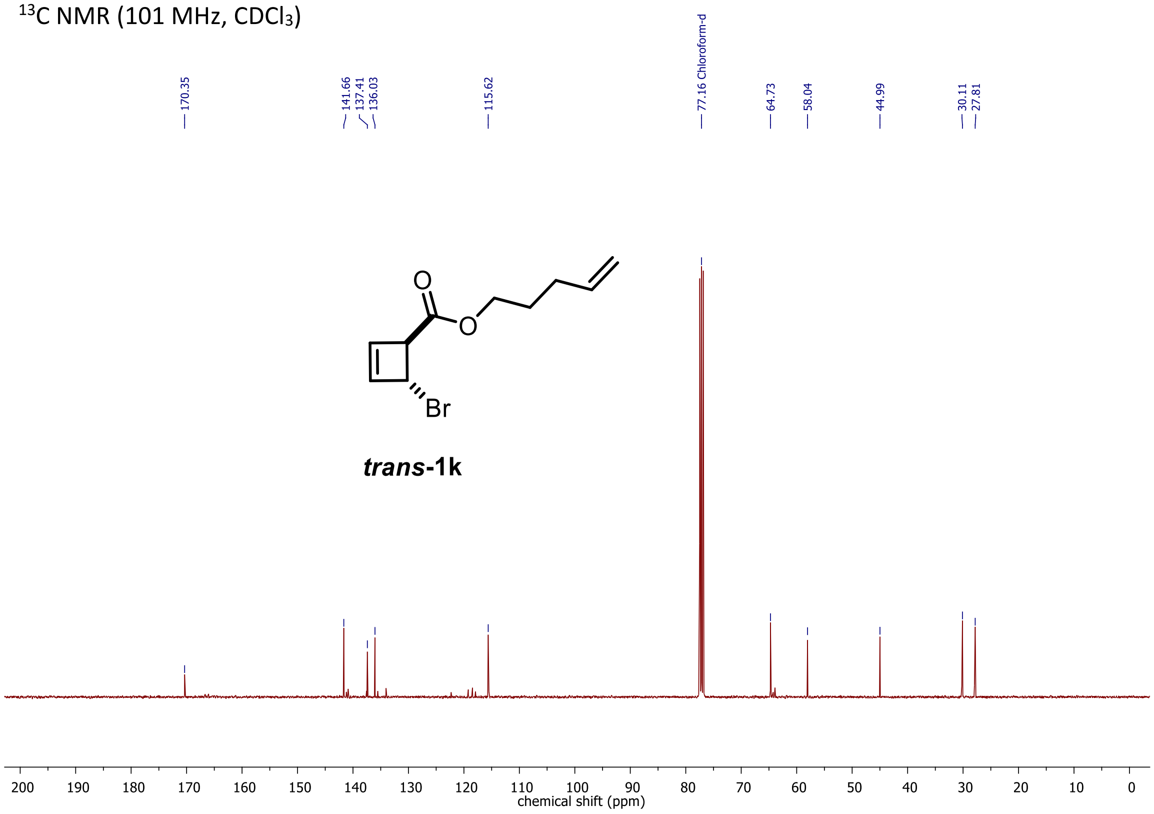

^{1}H NMR (400 MHz, $CDCl_3$)

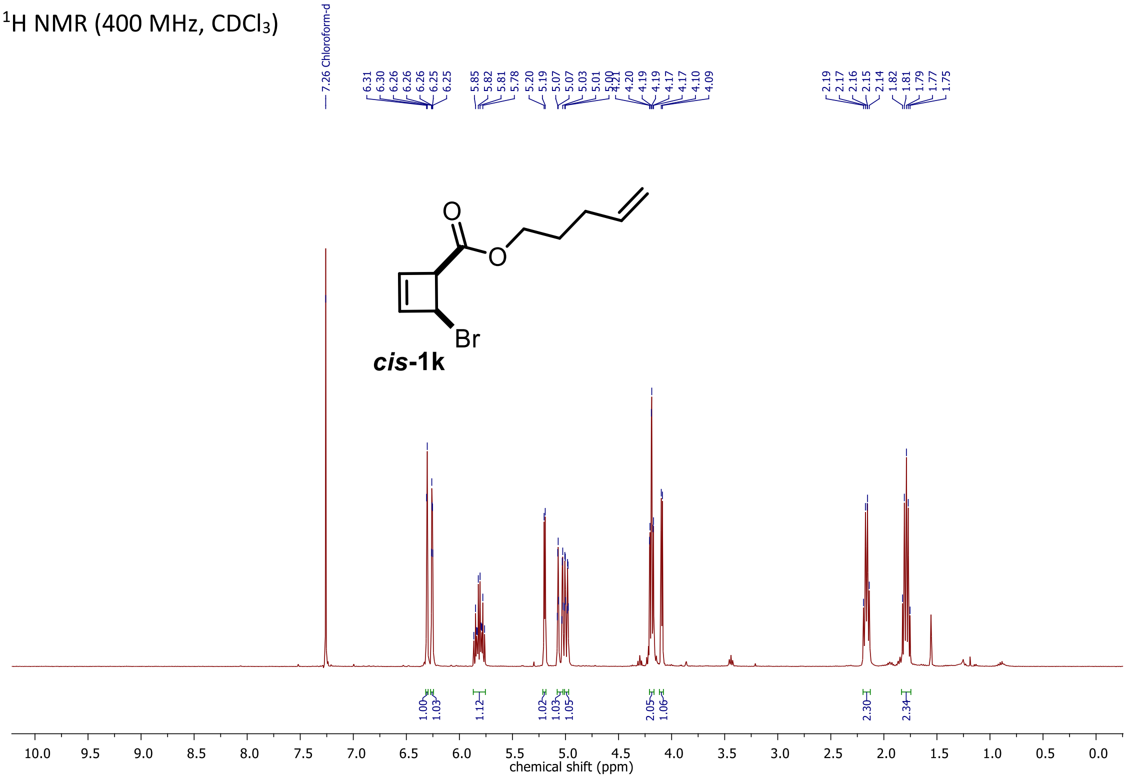

^{13}C NMR (101 MHz, $CDCl_3$)

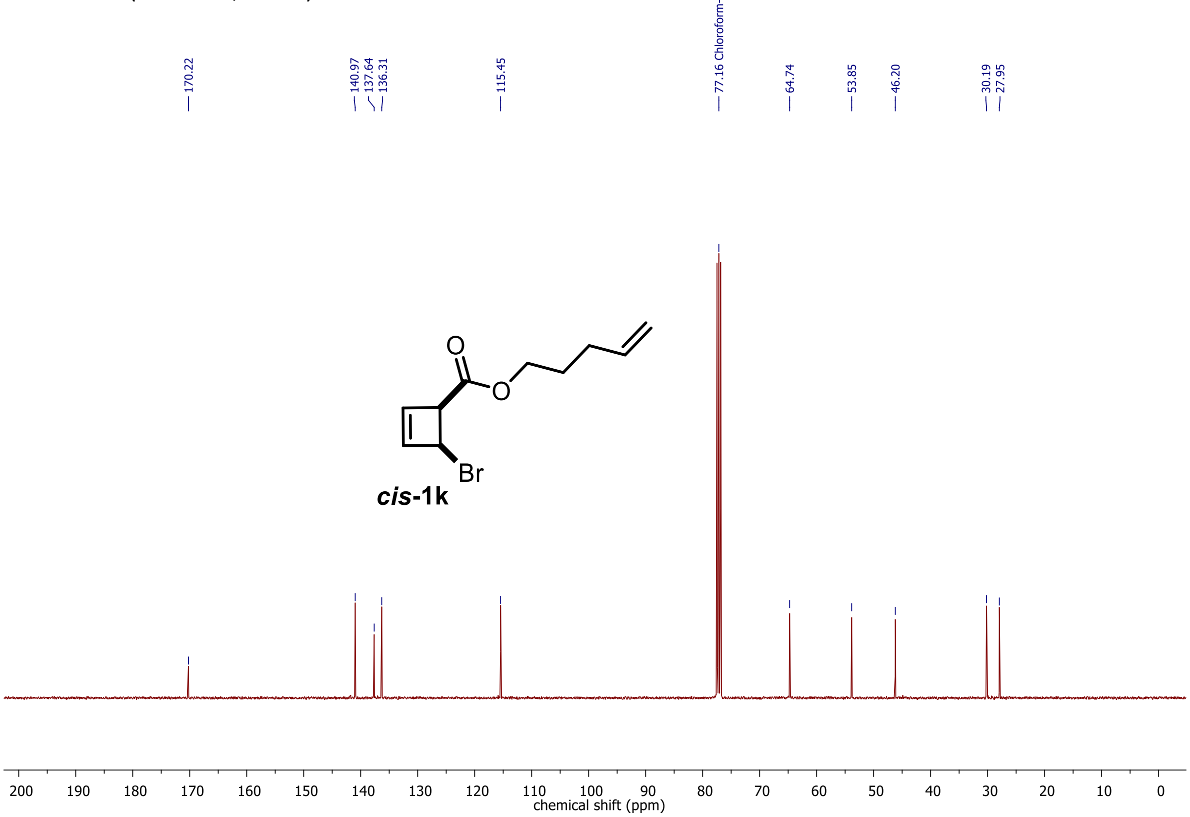

^{1}H NMR (400 MHz, $CDCl_3$)

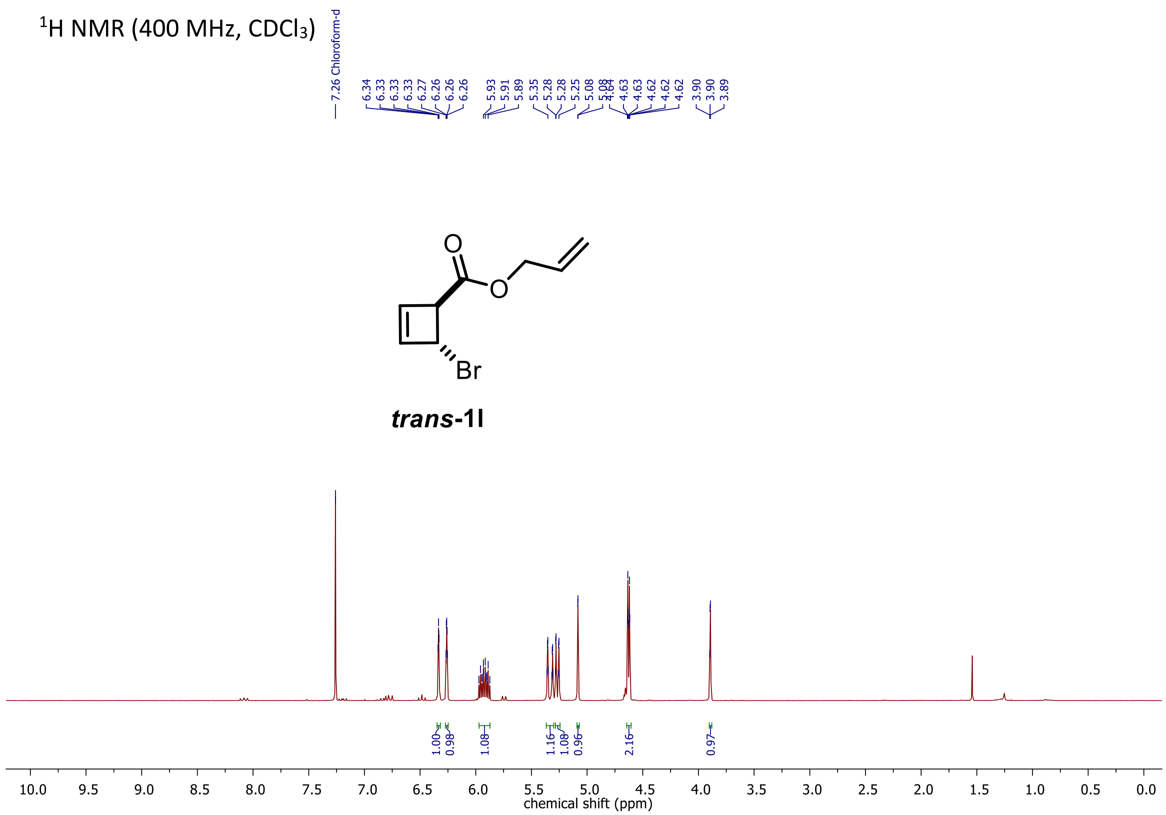

^{13}C NMR (101 MHz, $CDCl_3$)

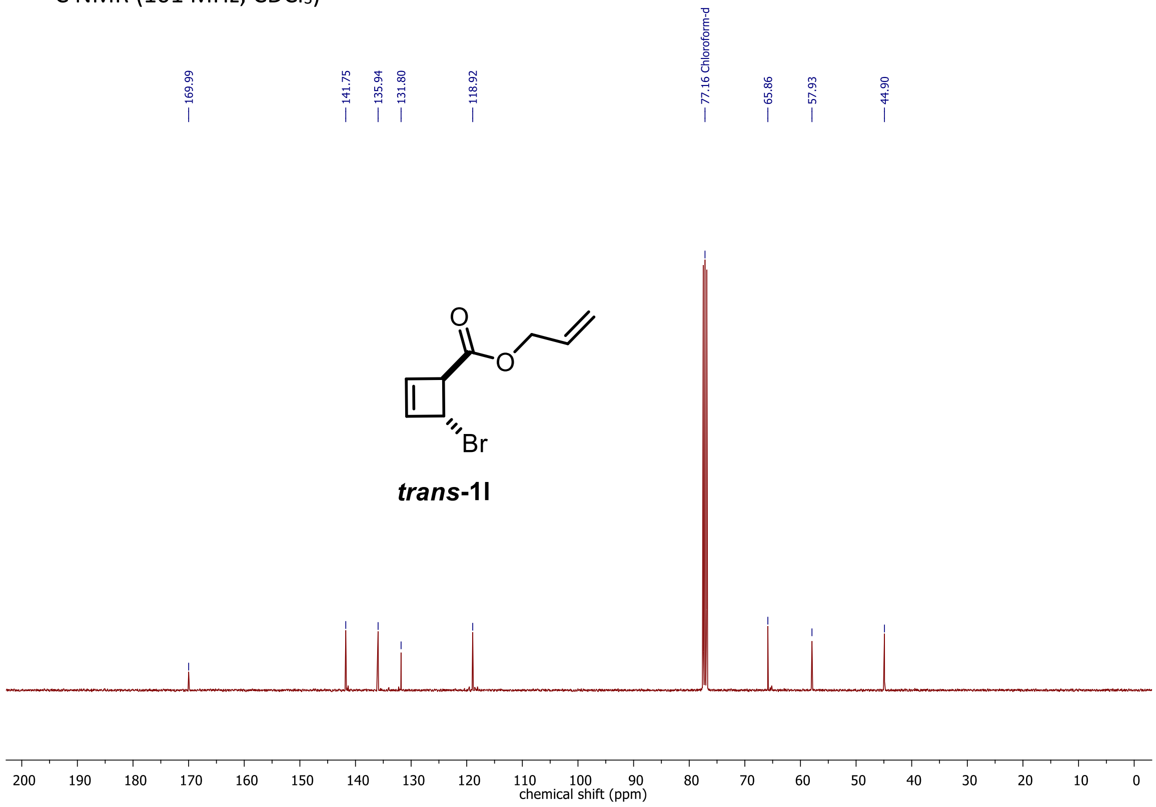

^{1}H NMR (400 MHz, $CDCl_3$)

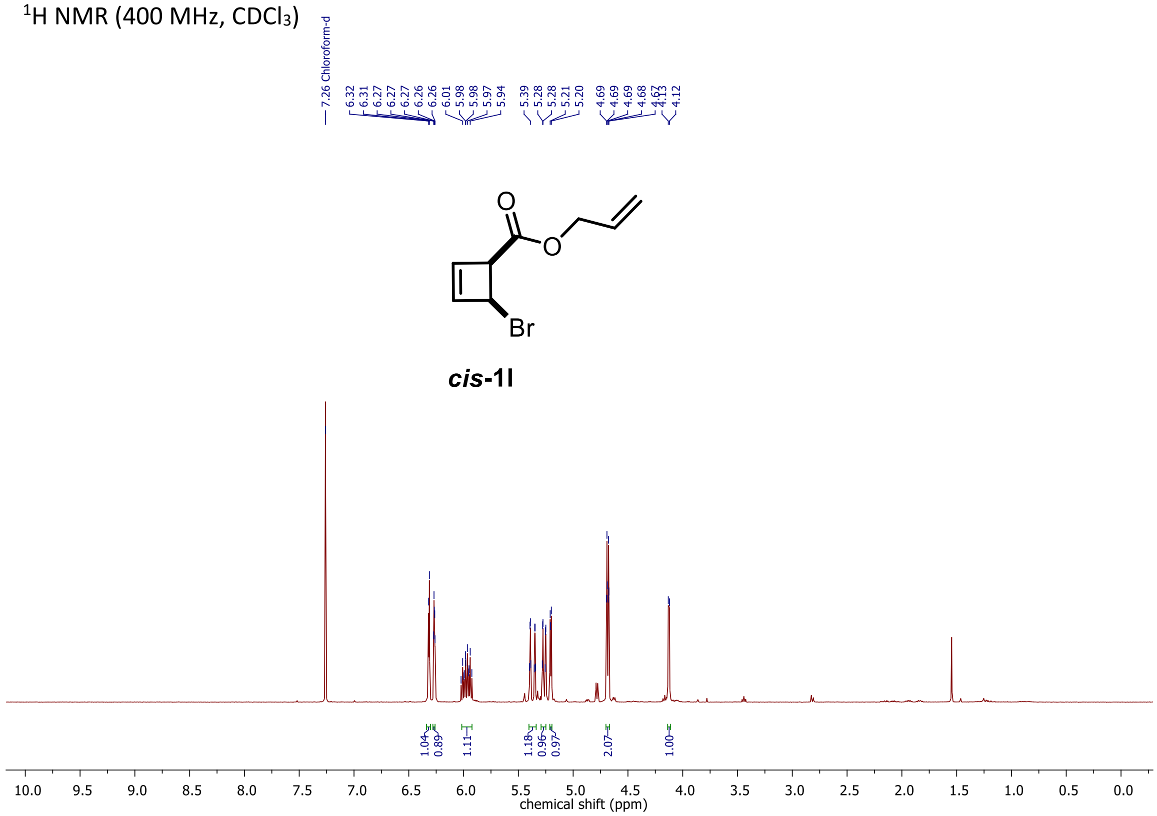

^{13}C NMR (101 MHz, $CDCl_3$)

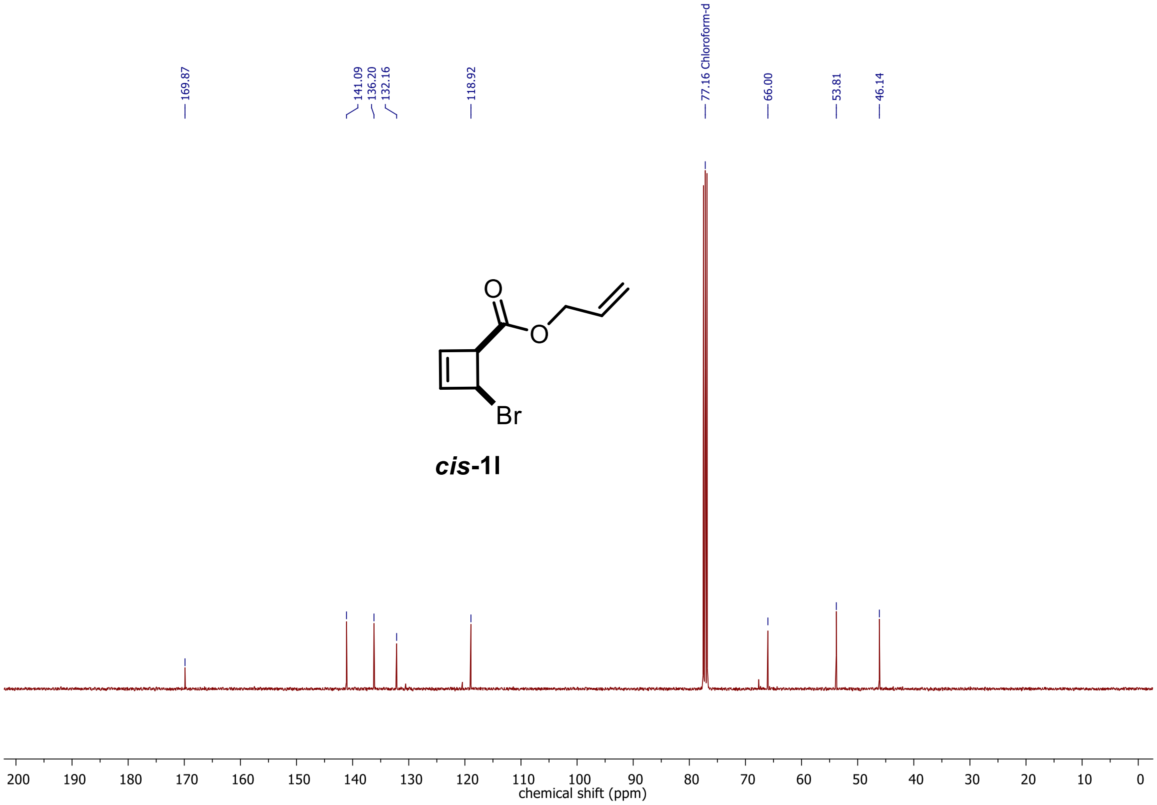

^{1}H NMR (400 MHz, $CDCl_3$)

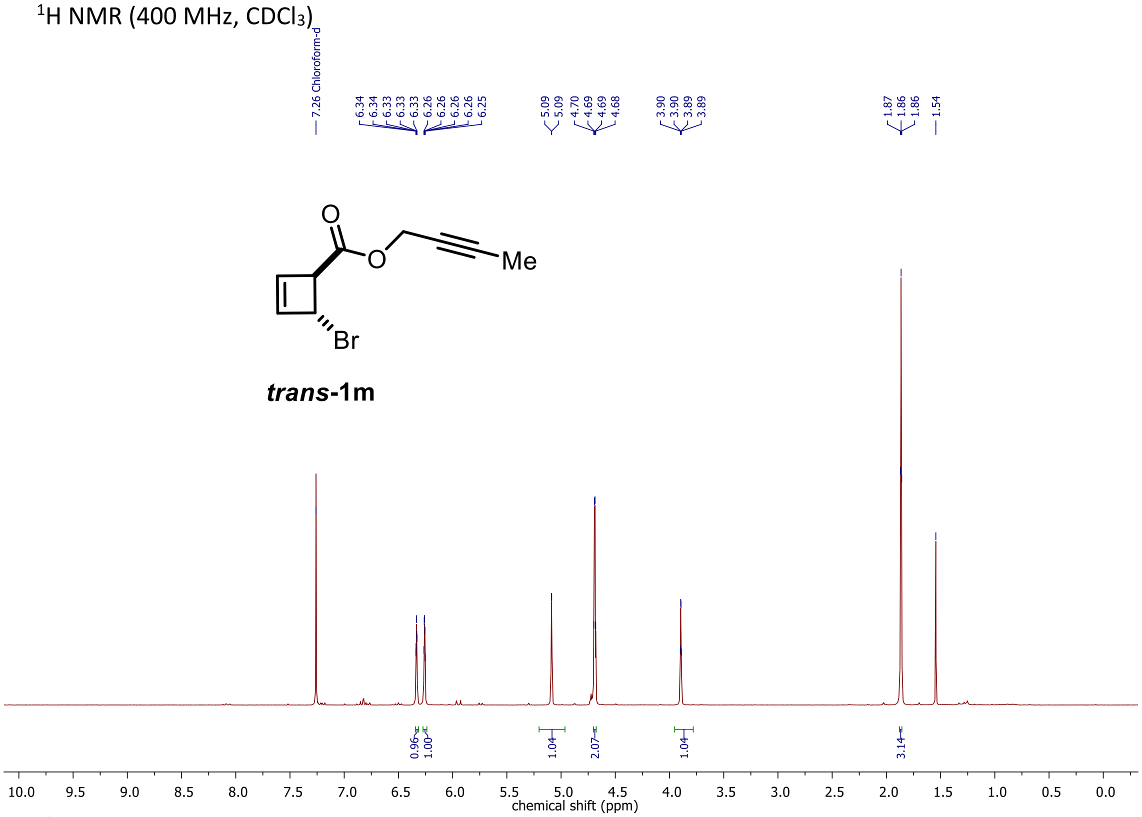

^{13}C NMR (101 MHz, $CDCl_3$)

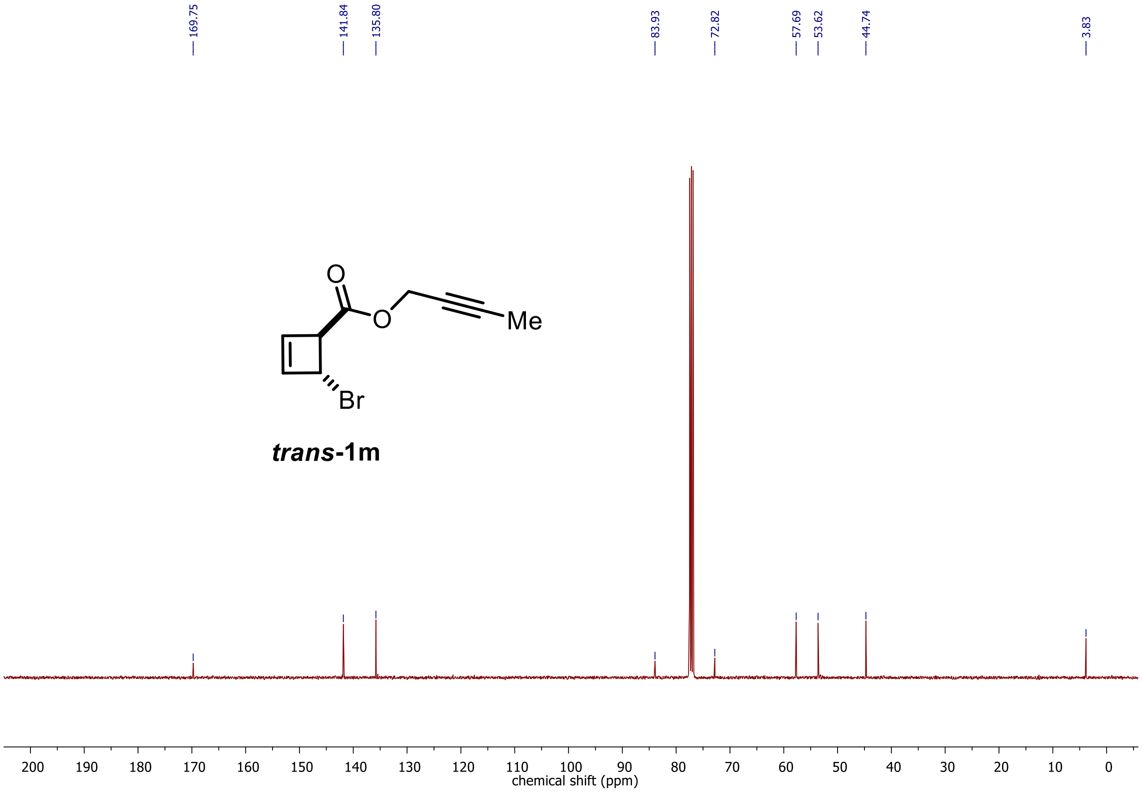

^{1}H NMR (400 MHz, $CDCl_3$)

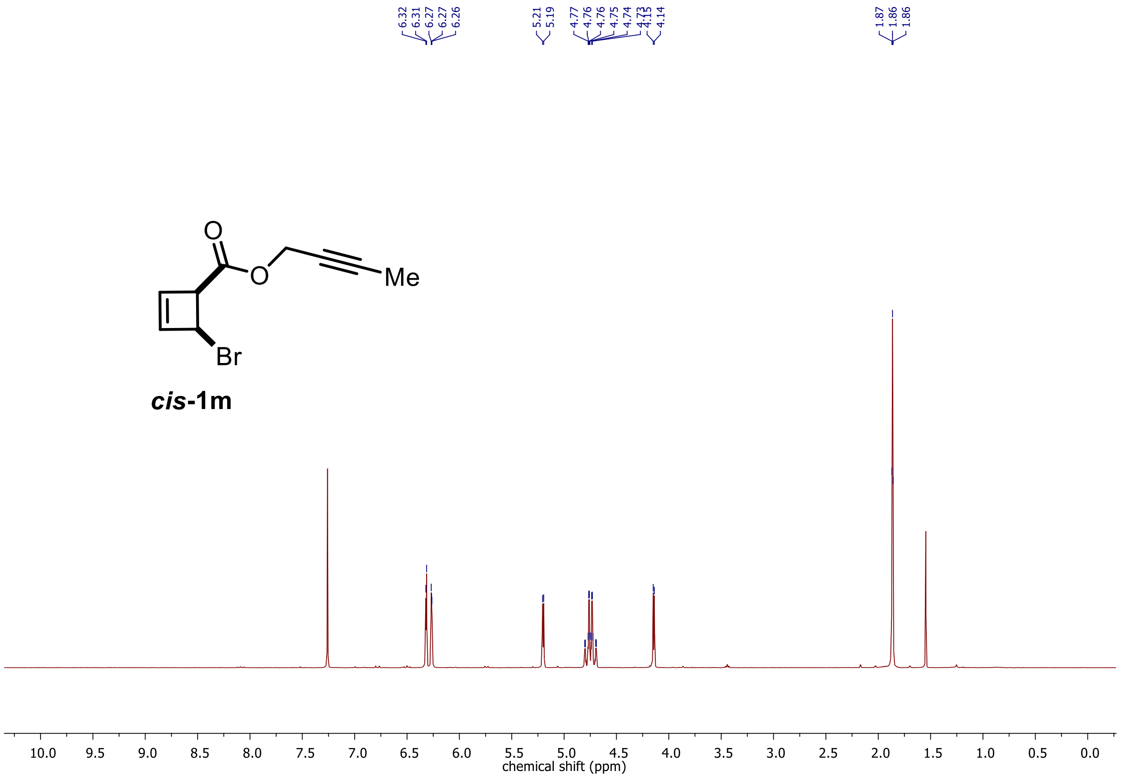

^{13}C NMR (101 MHz, $CDCl_3$)

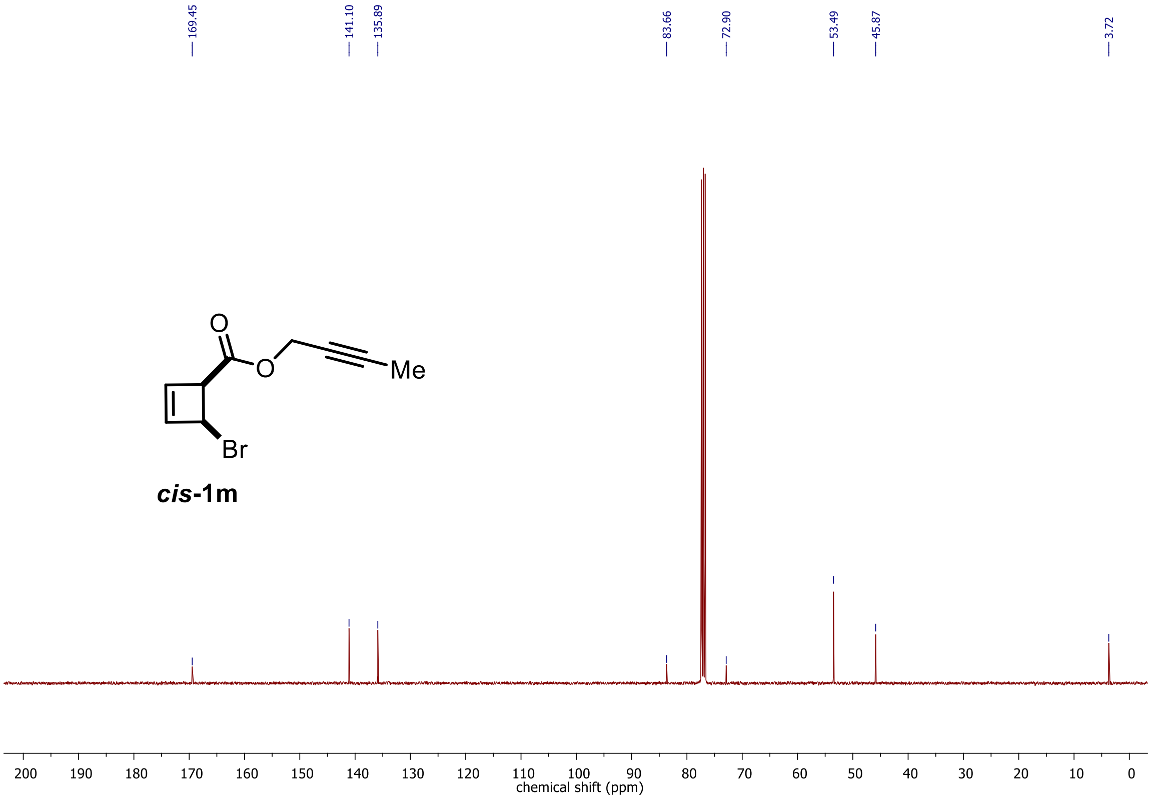

^{1}H NMR (600 MHz, $CDCl_3$)

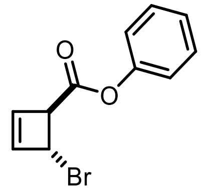

***trans*-1n**

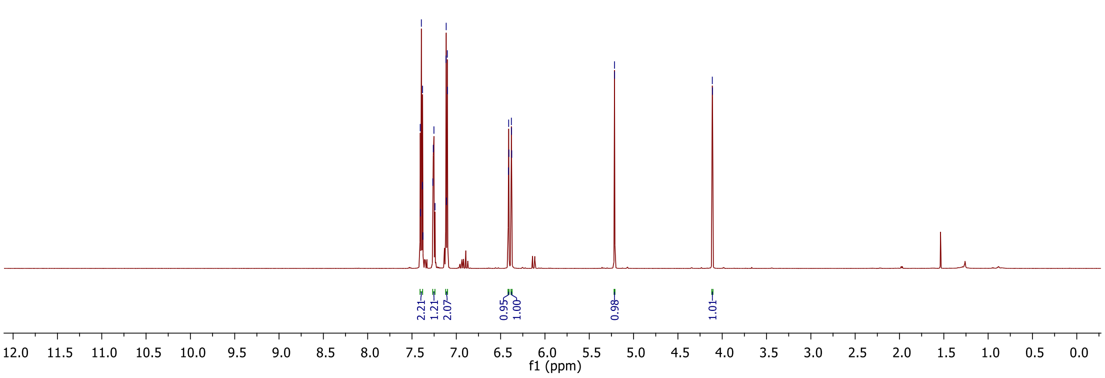

^{13}C NMR (151 MHz, $CDCl_3$)

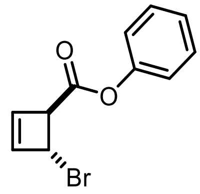

***trans*-1n**

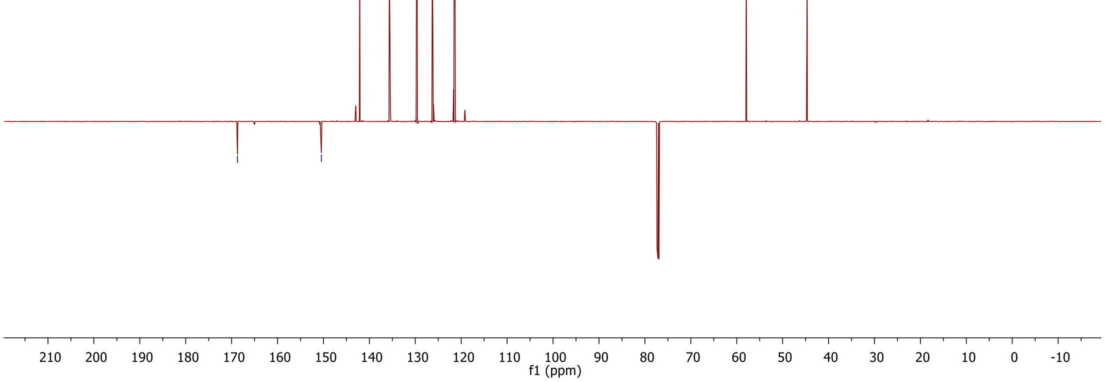

^{1}H NMR (400 MHz, $CDCl_3$)

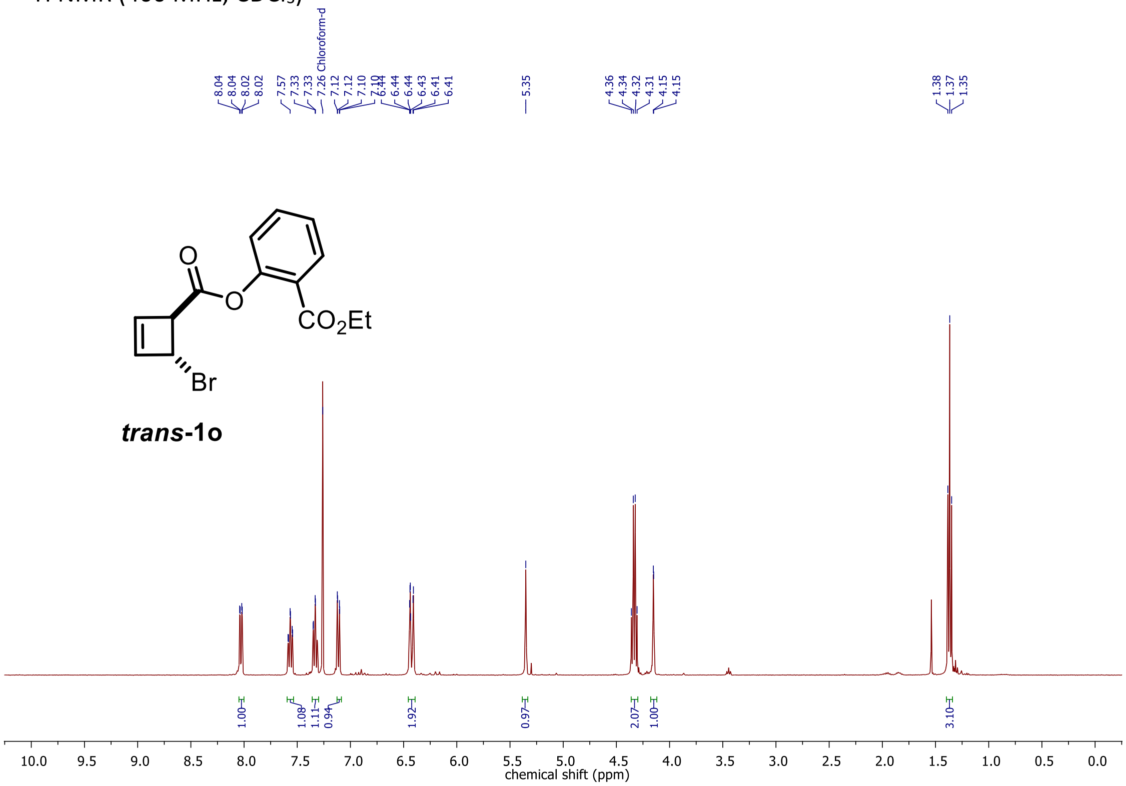

^{13}C NMR (101 MHz, $CDCl_3$)

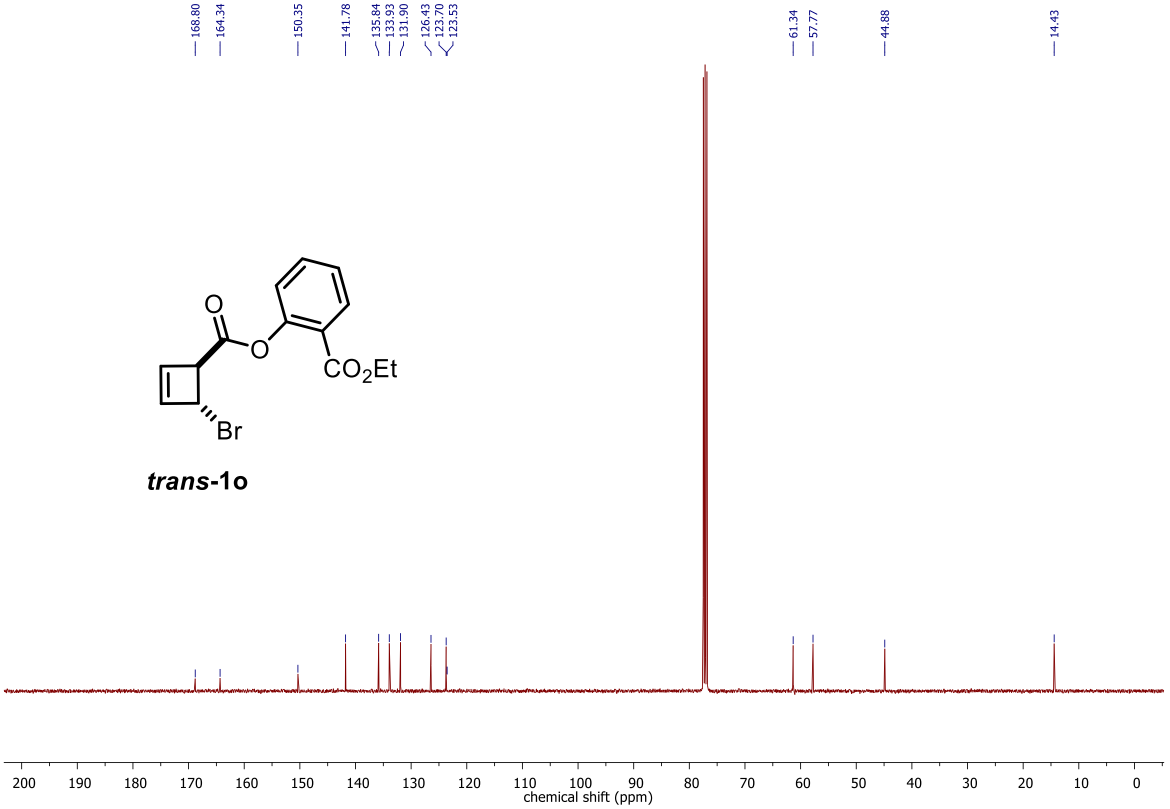

^1H NMR (400 MHz, $CDCl_3$)

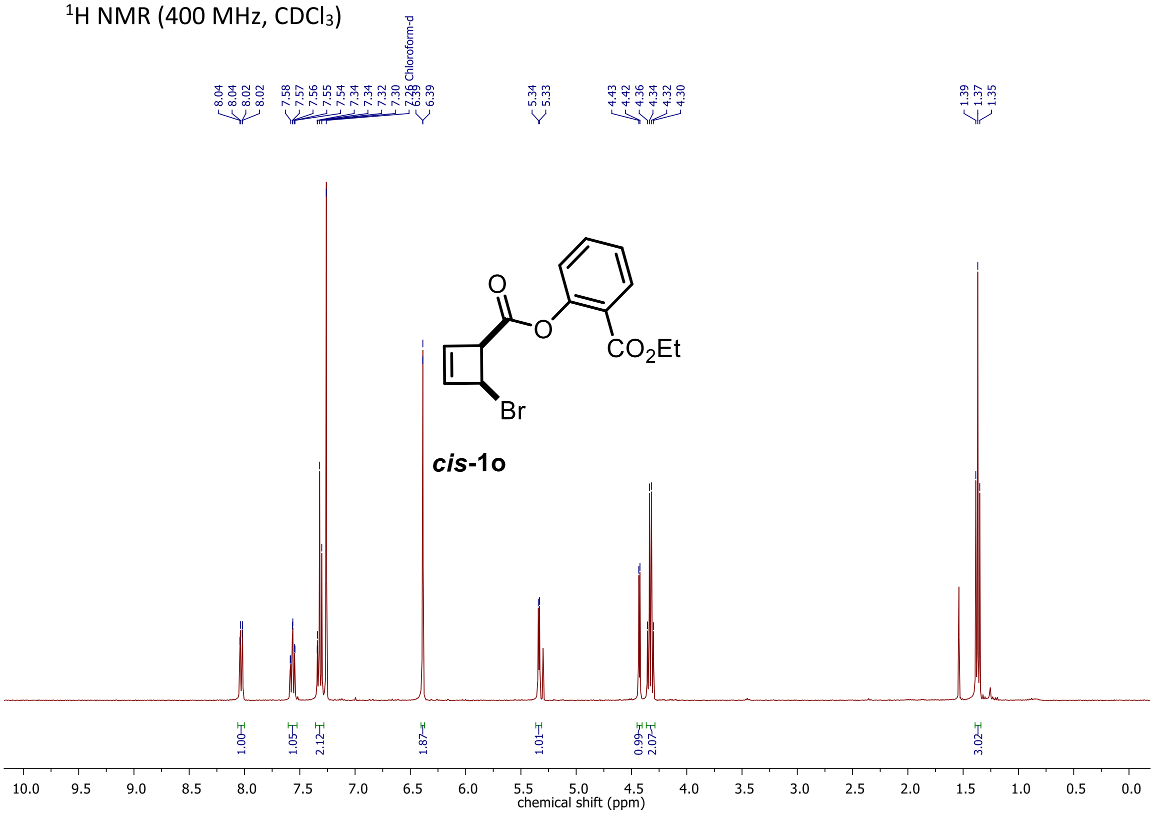

^{13}C NMR (101 MHz, $CDCl_3$)

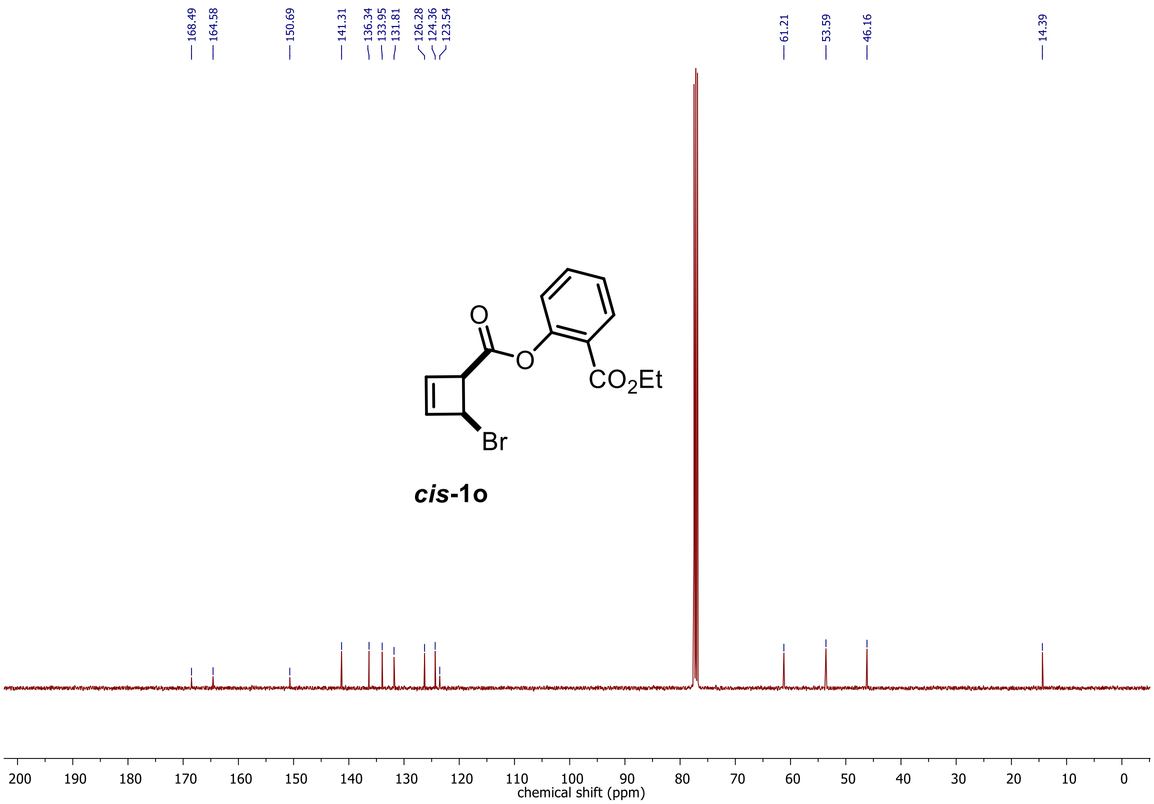

^{1}H NMR (400 MHz, $CDCl_3$)

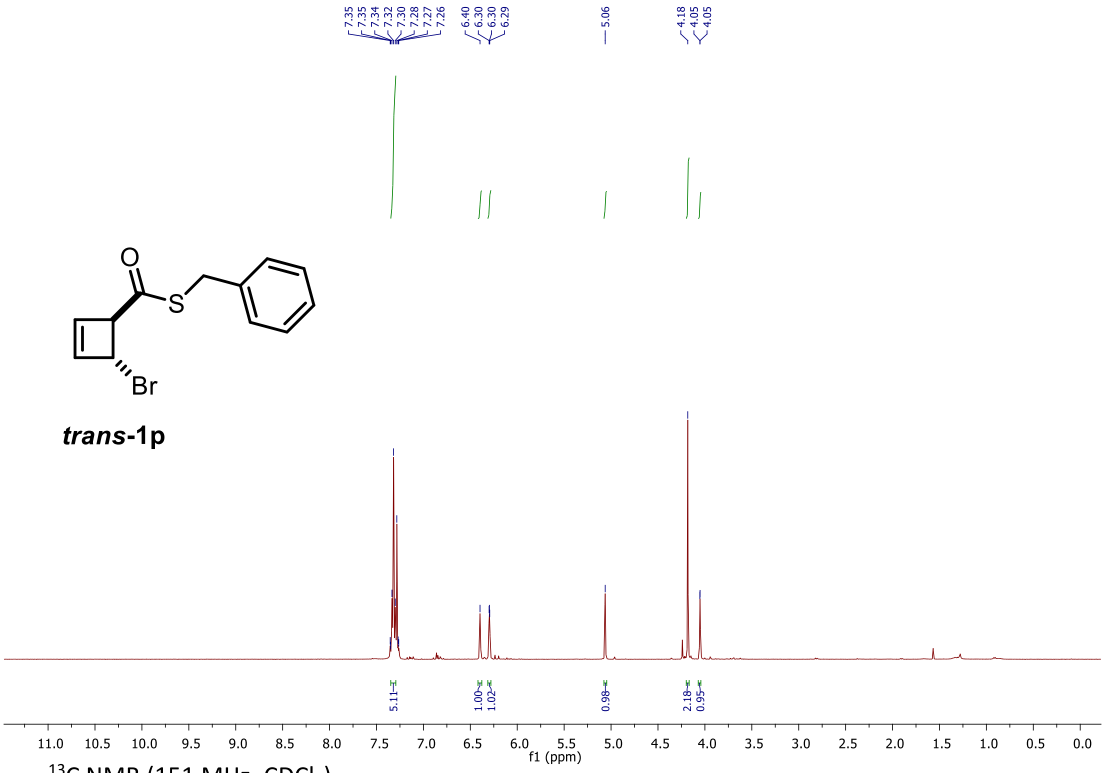

^{13}C NMR (151 MHz, $CDCl_3$)

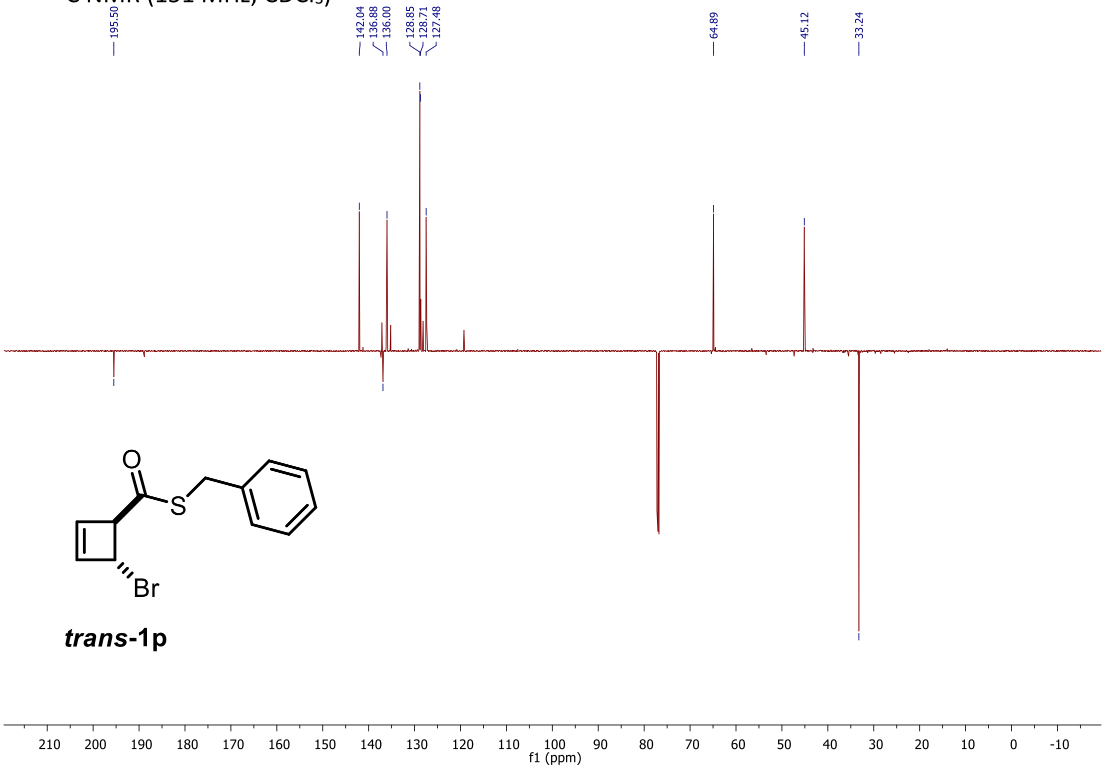

^1H NMR (600 MHz, $CDCl_3$)

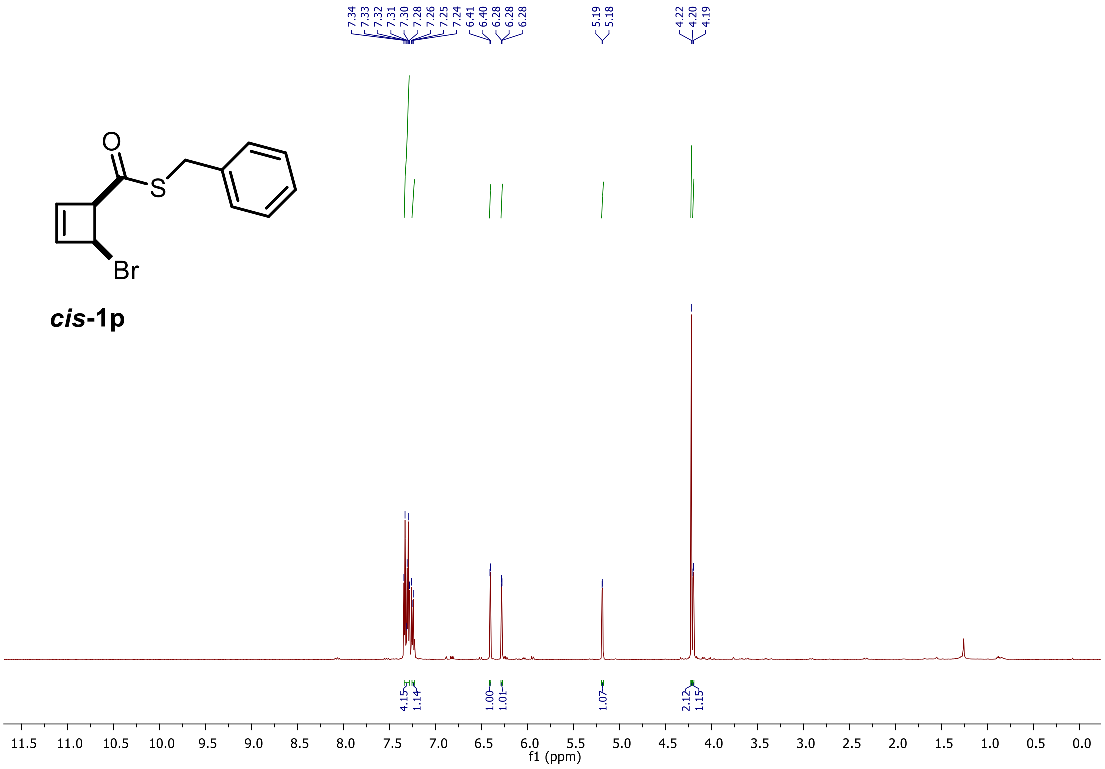

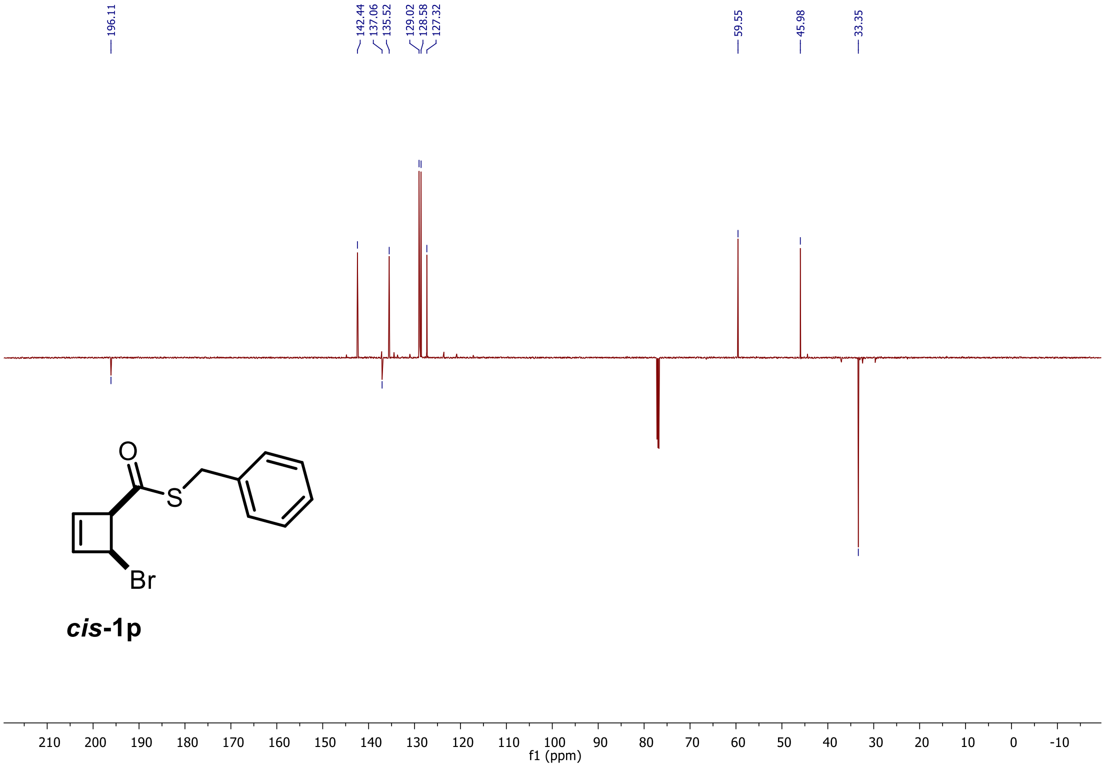

^{1}H NMR (600 MHz, $CDCl_3$)

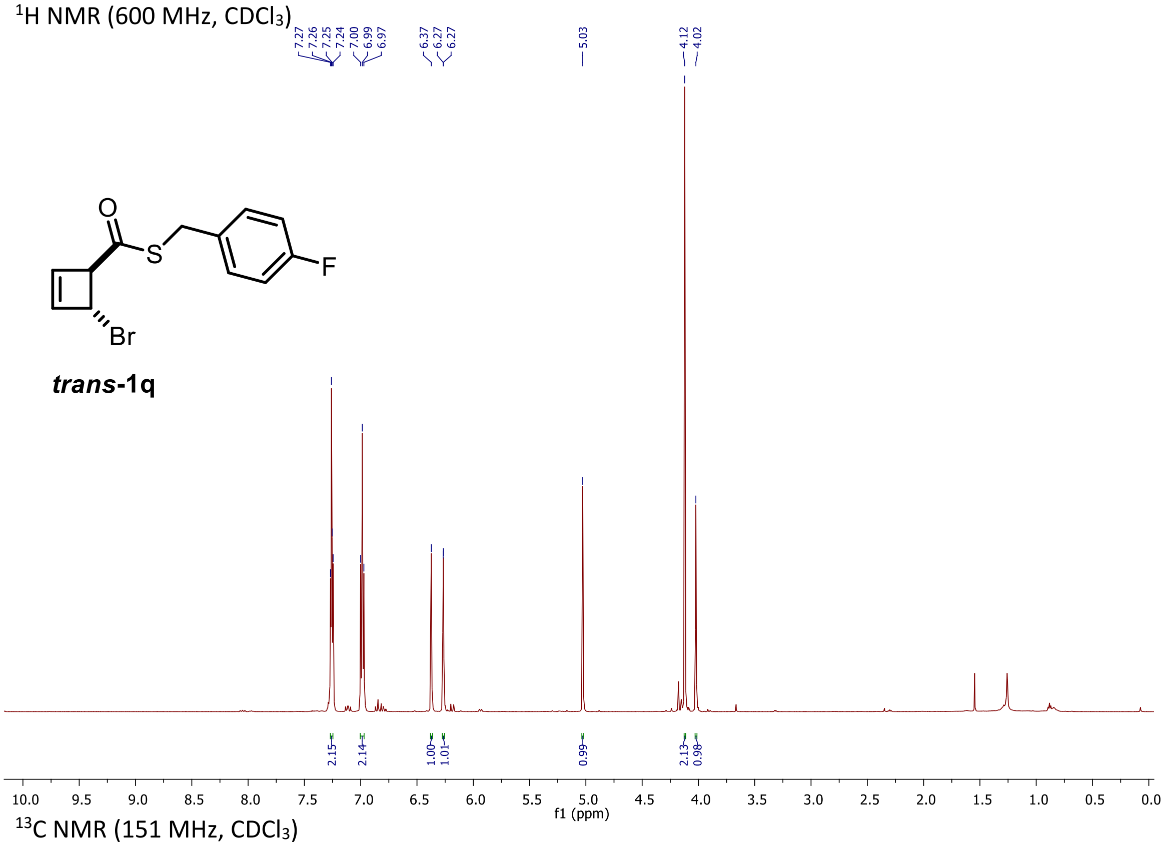

^{13}C NMR (151 MHz, $CDCl_3$)

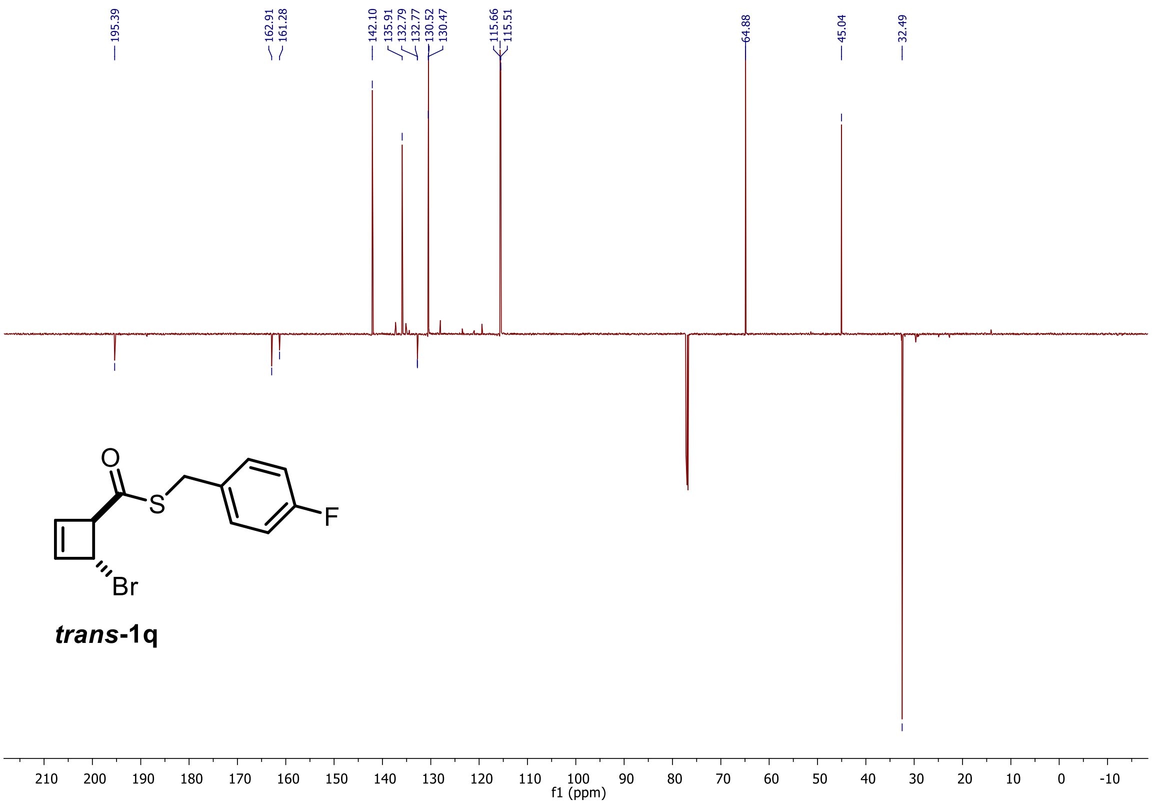

^{19}F NMR (565 MHz, $CDCl_3$)

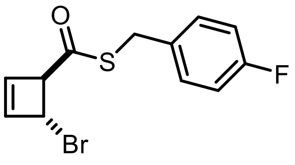

***trans*-1q**

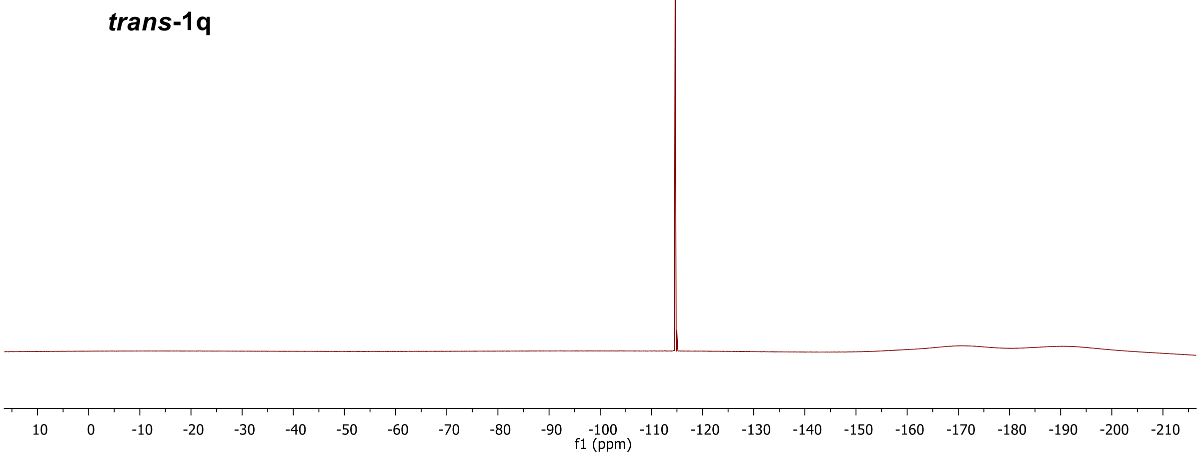

^{1}H NMR (400 MHz, $CDCl_3$)

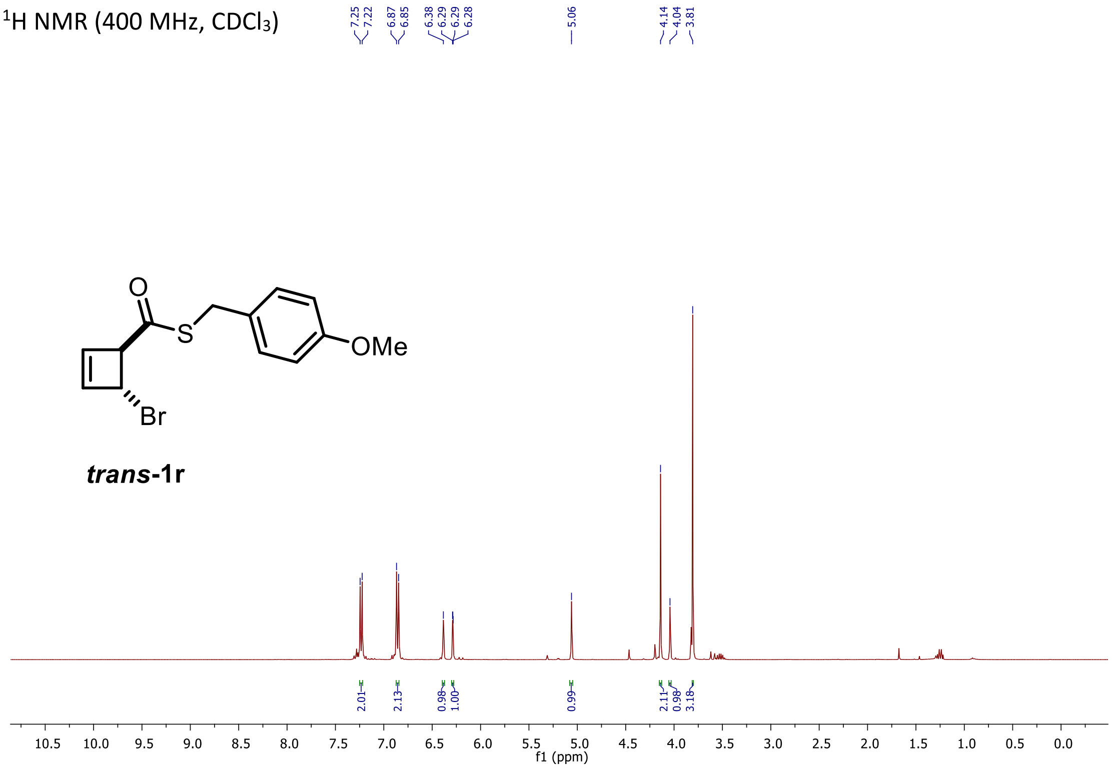

^{13}C NMR (151 MHz, $CDCl_3$)

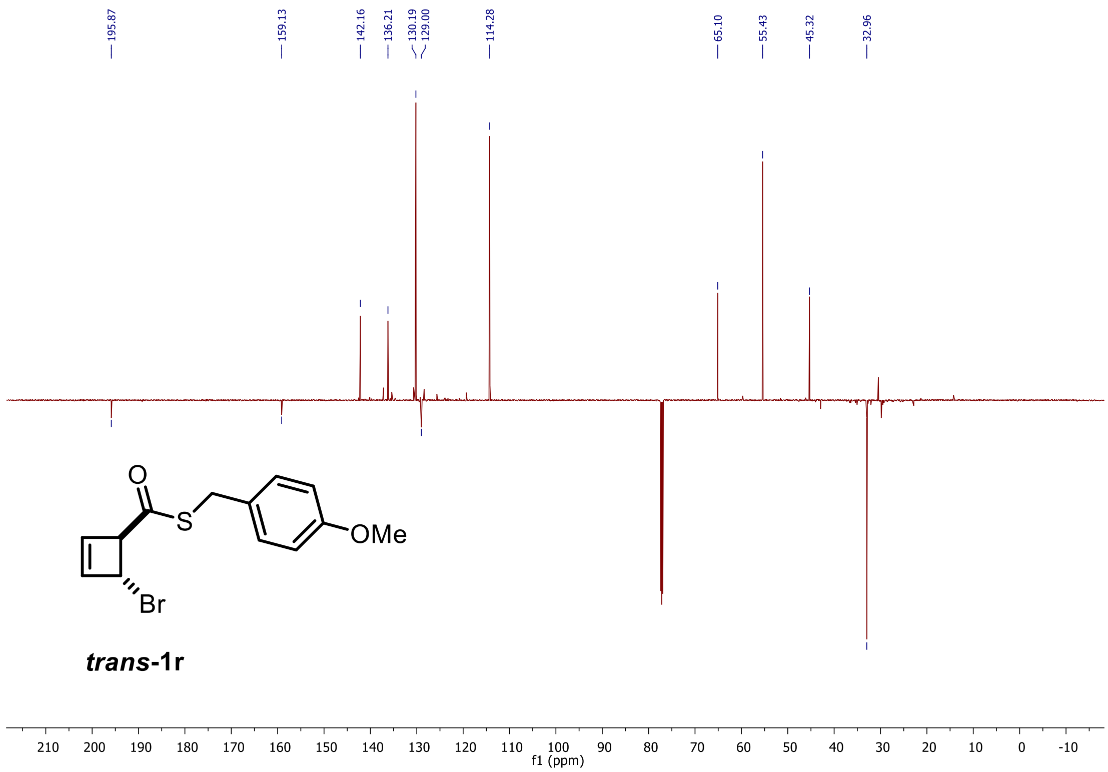

^{1}H NMR (400 MHz, $CDCl_3$)

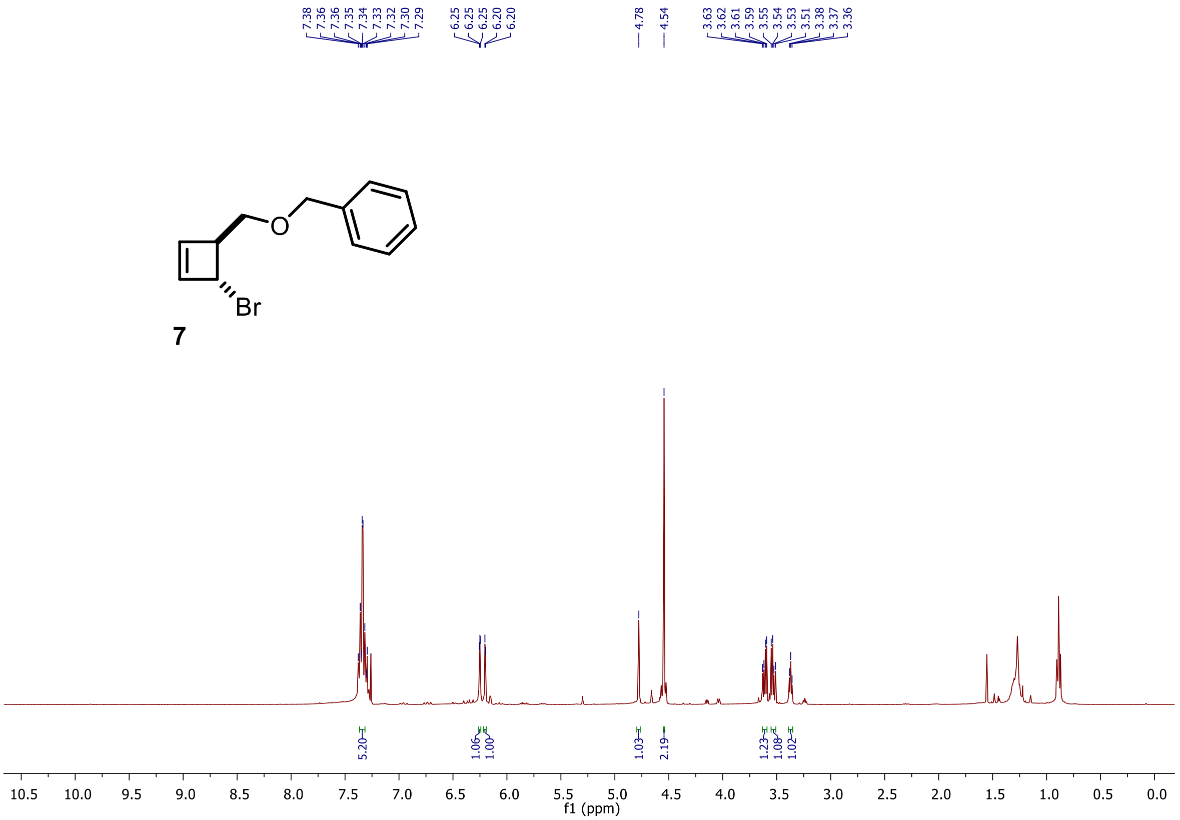

^{13}C NMR (151 MHz, $CDCl_3$)

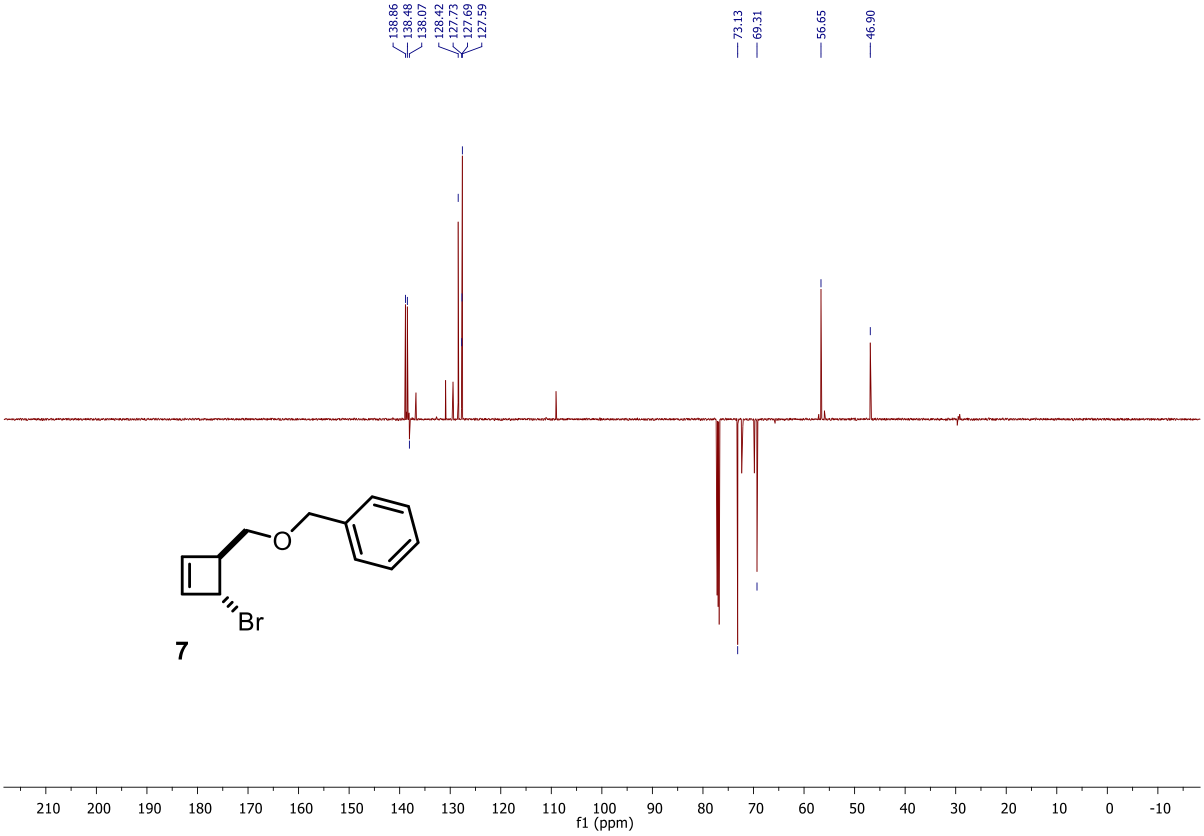

^1H NMR (700 MHz, $CDCl_3$)

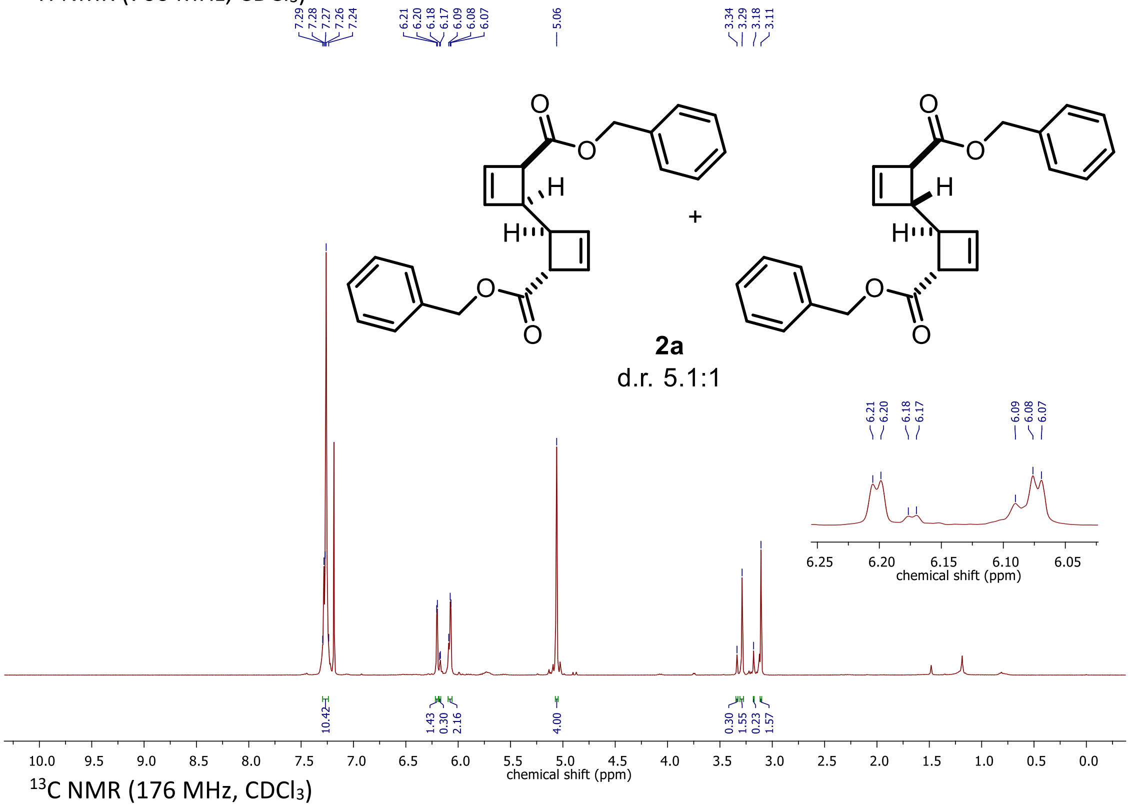

^{13}C NMR (176 MHz, $CDCl_3$)

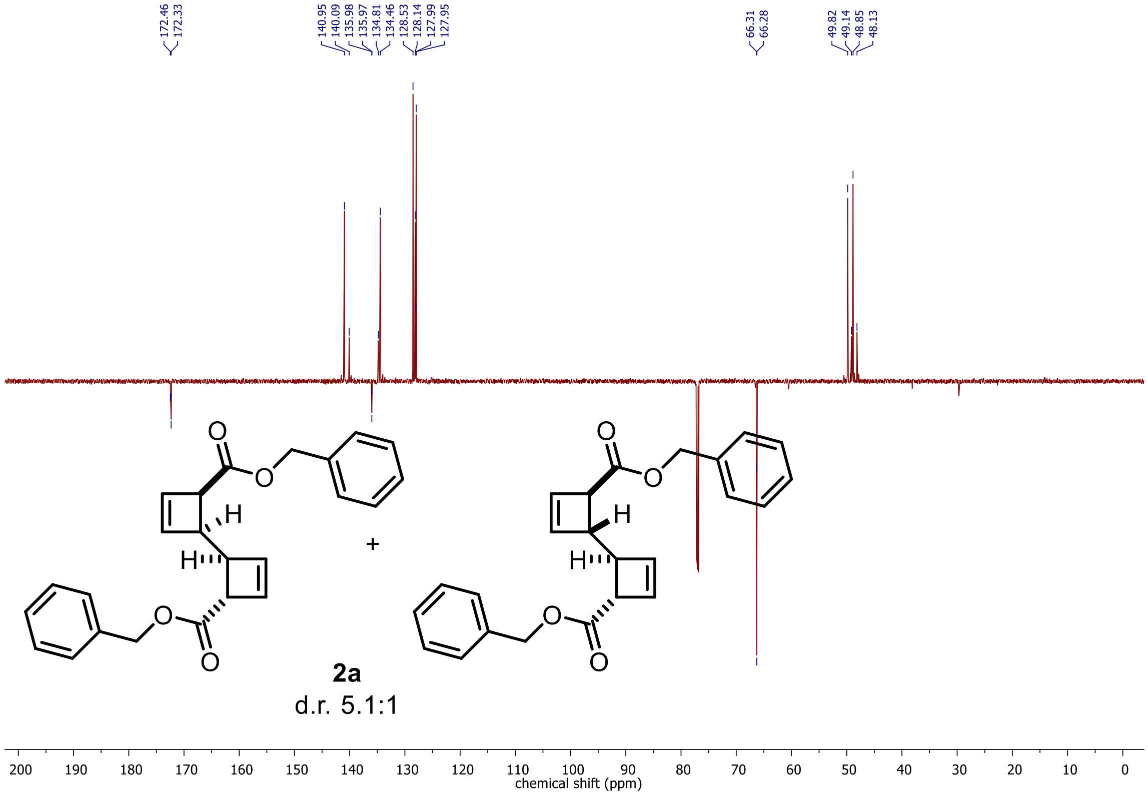

^{1}H NMR (700 MHz, $CDCl_3$)

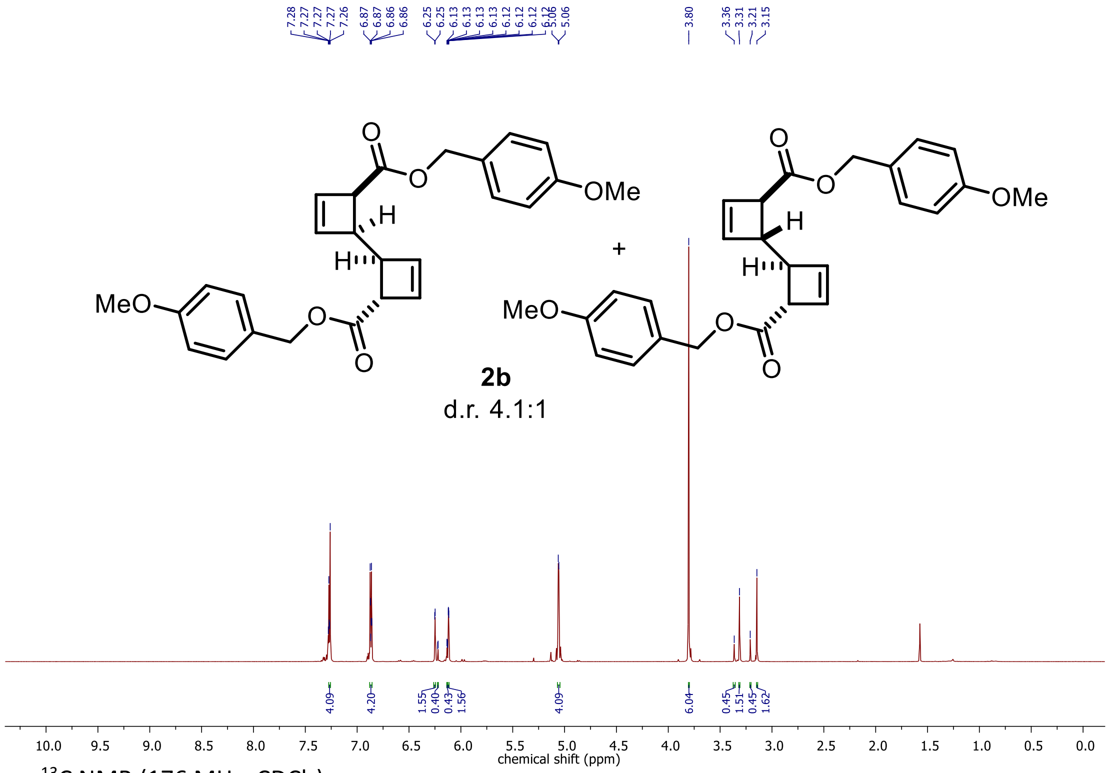

^{13}C NMR (176 MHz, $CDCl_3$)

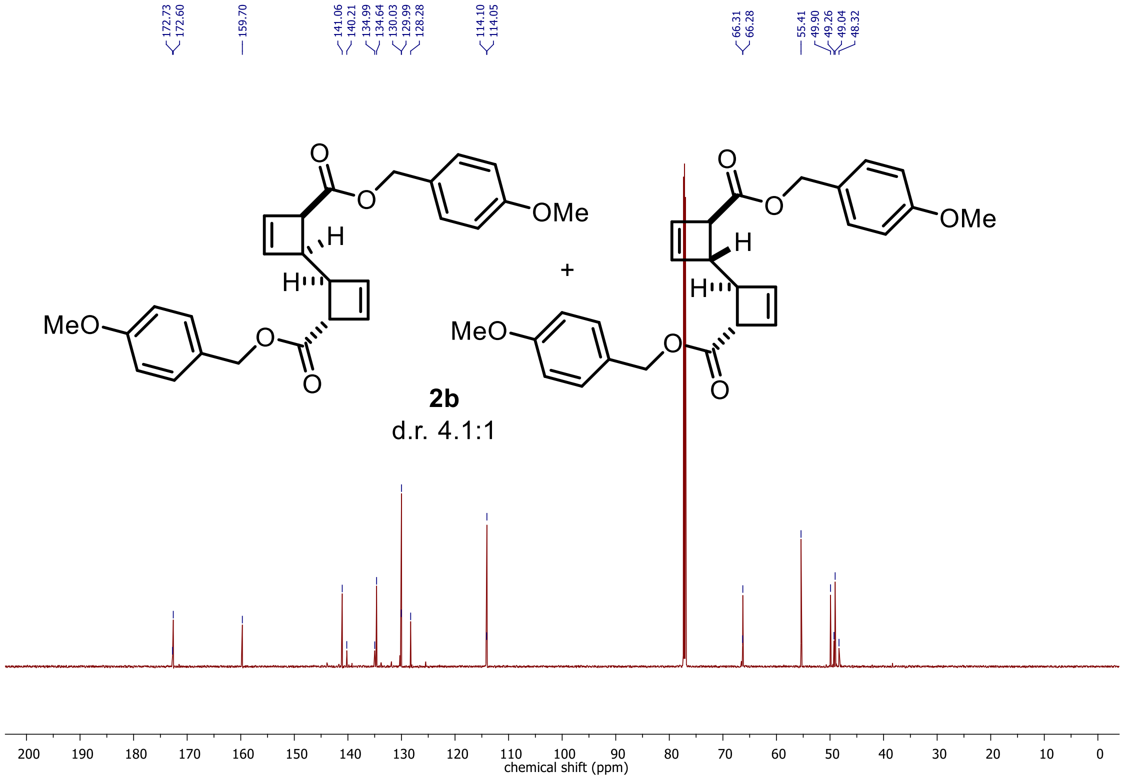

^{1}H NMR (600 MHz, $CDCl_3$)

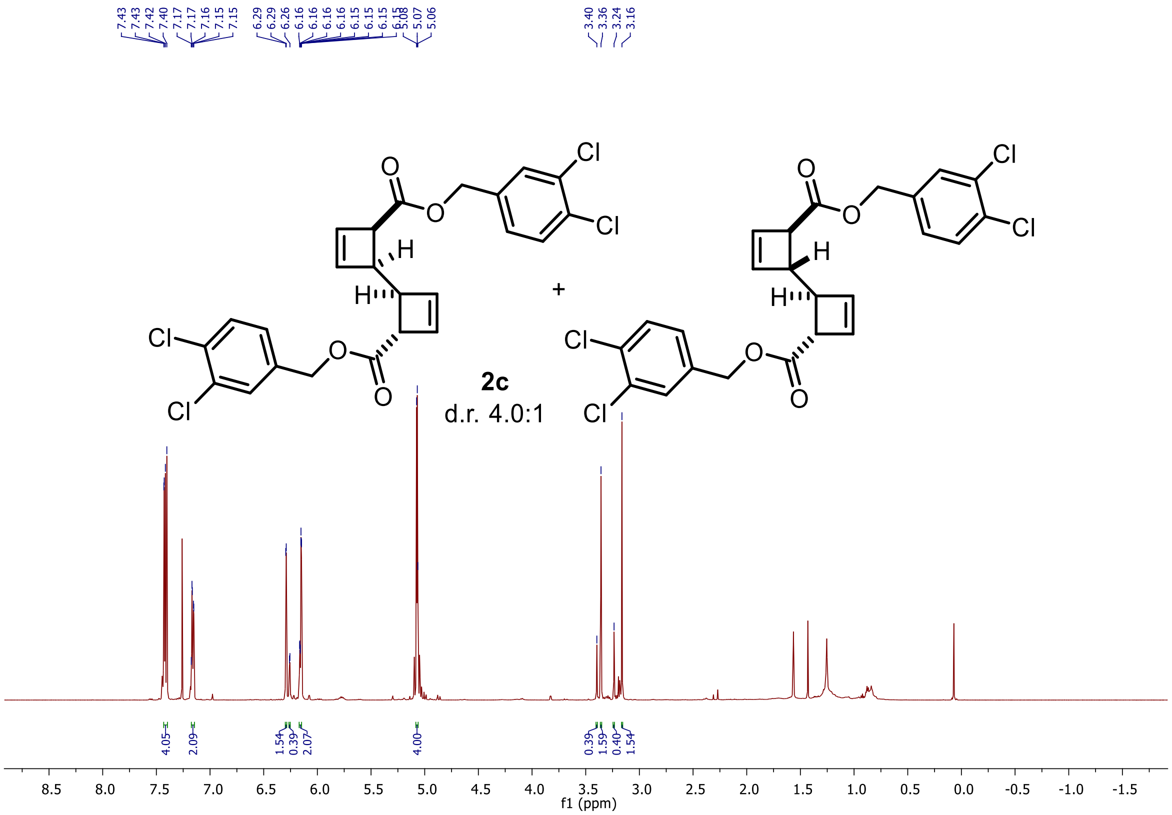

^{13}C NMR (151 MHz, $CDCl_3$)

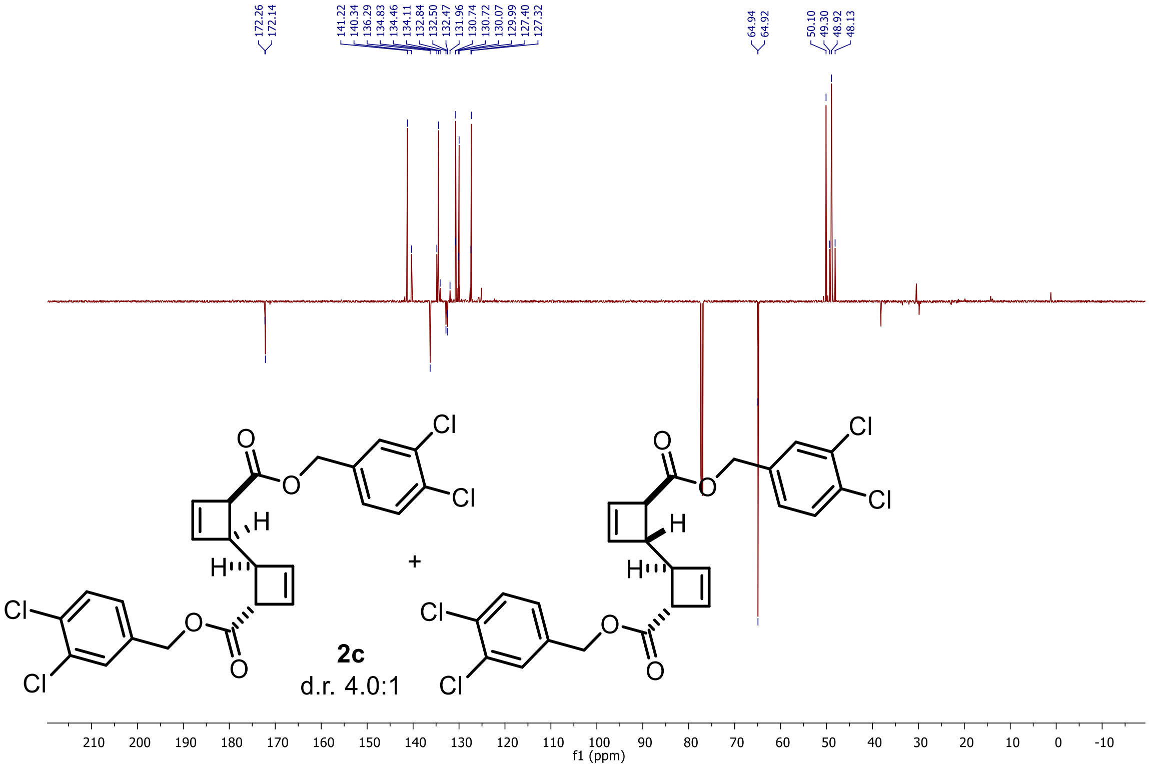

^{1}H NMR (600 MHz, $CDCl_3$)

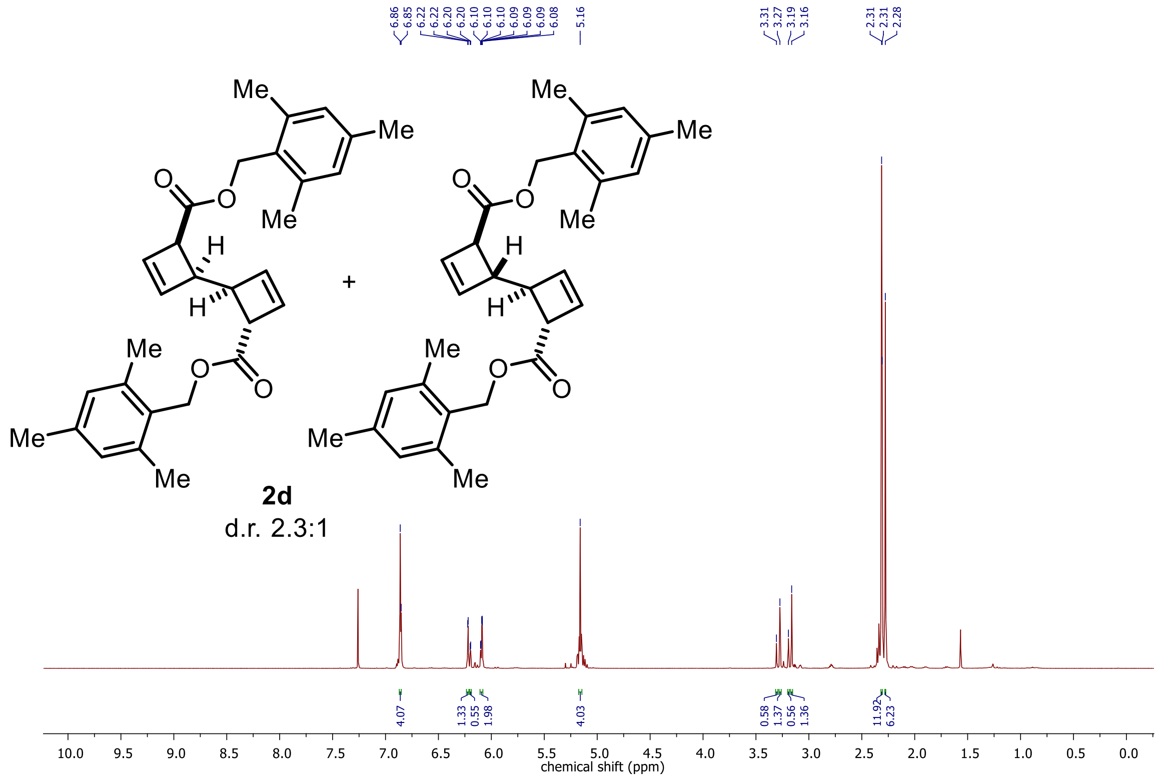

^{13}C NMR (151 MHz, $CDCl_3$)

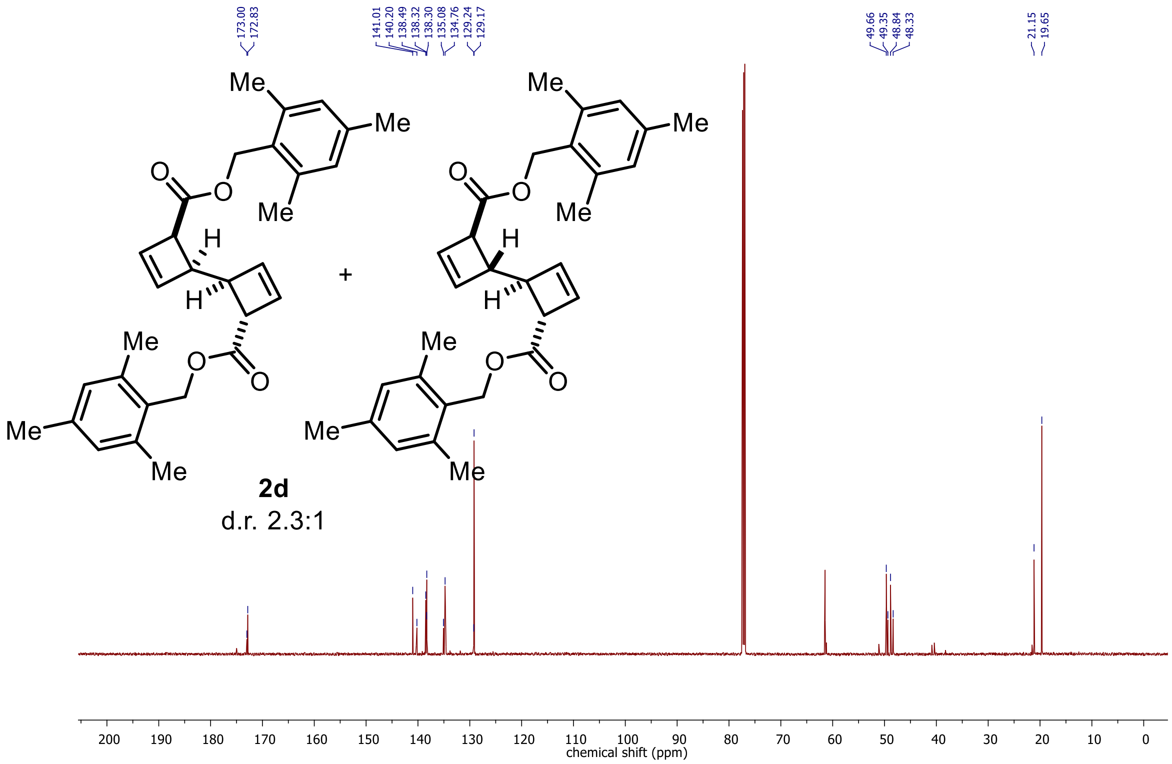

^{1}H NMR (600 MHz, $CDCl_3$)

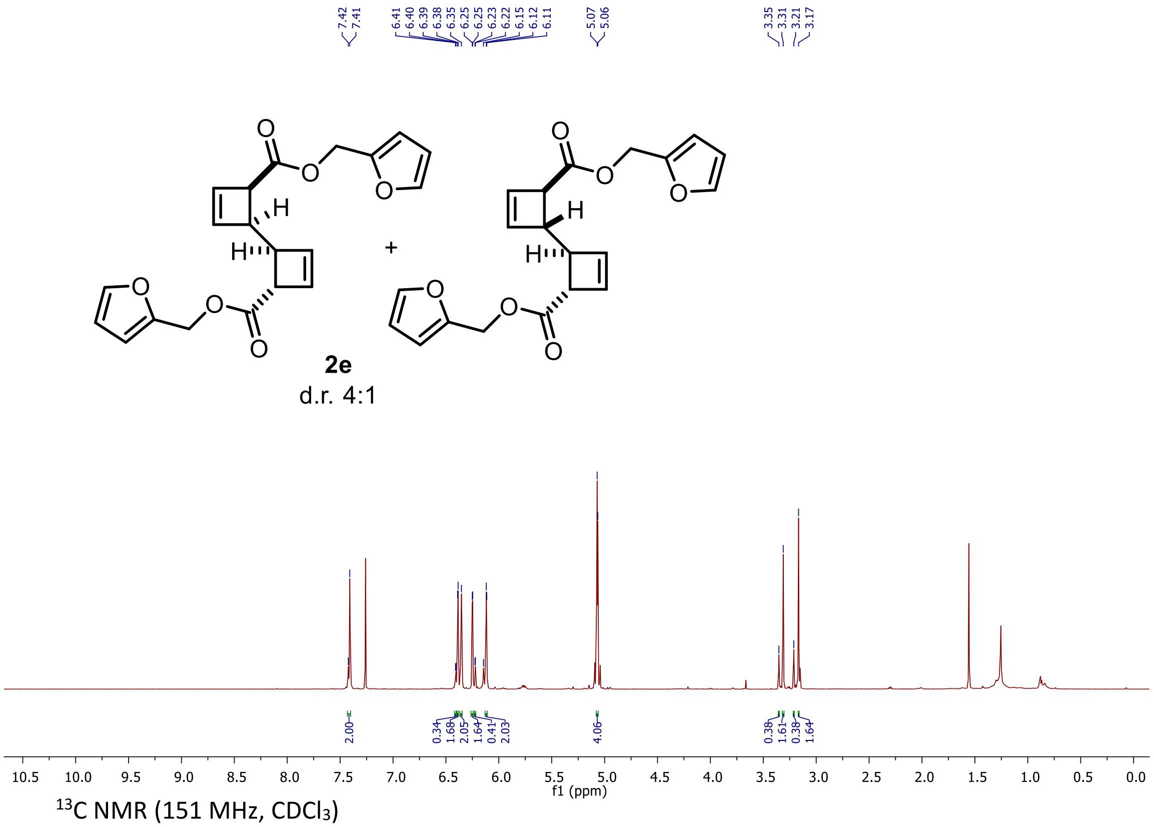

^{13}C NMR (151 MHz, $CDCl_3$)

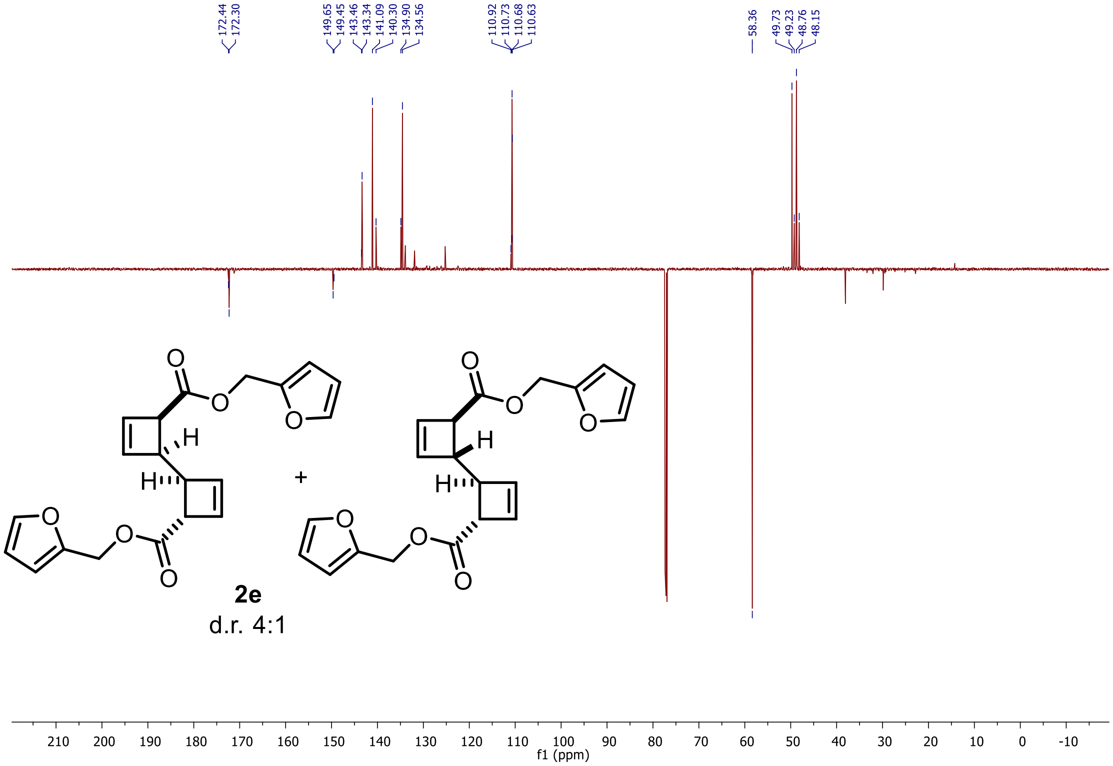

^{1}H NMR (700 MHz, $CDCl_3$)

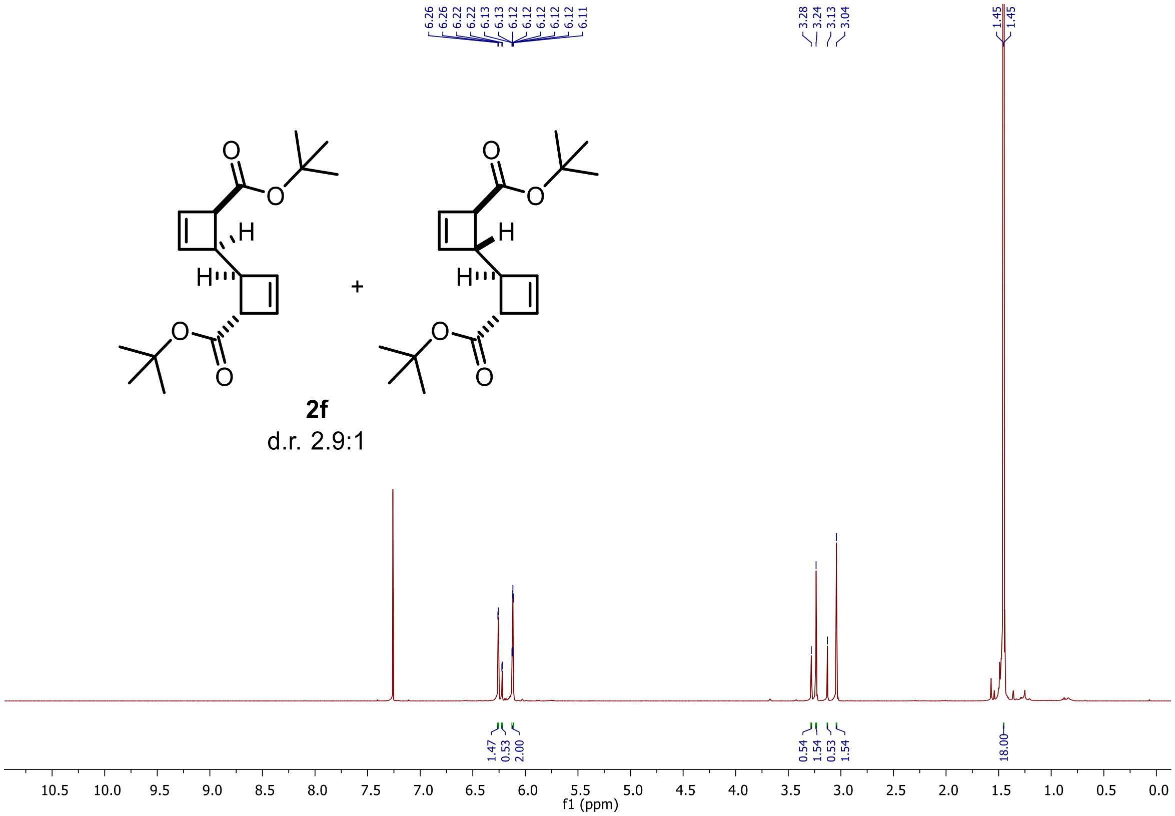

^{13}C NMR (176 MHz, $CDCl_3$)

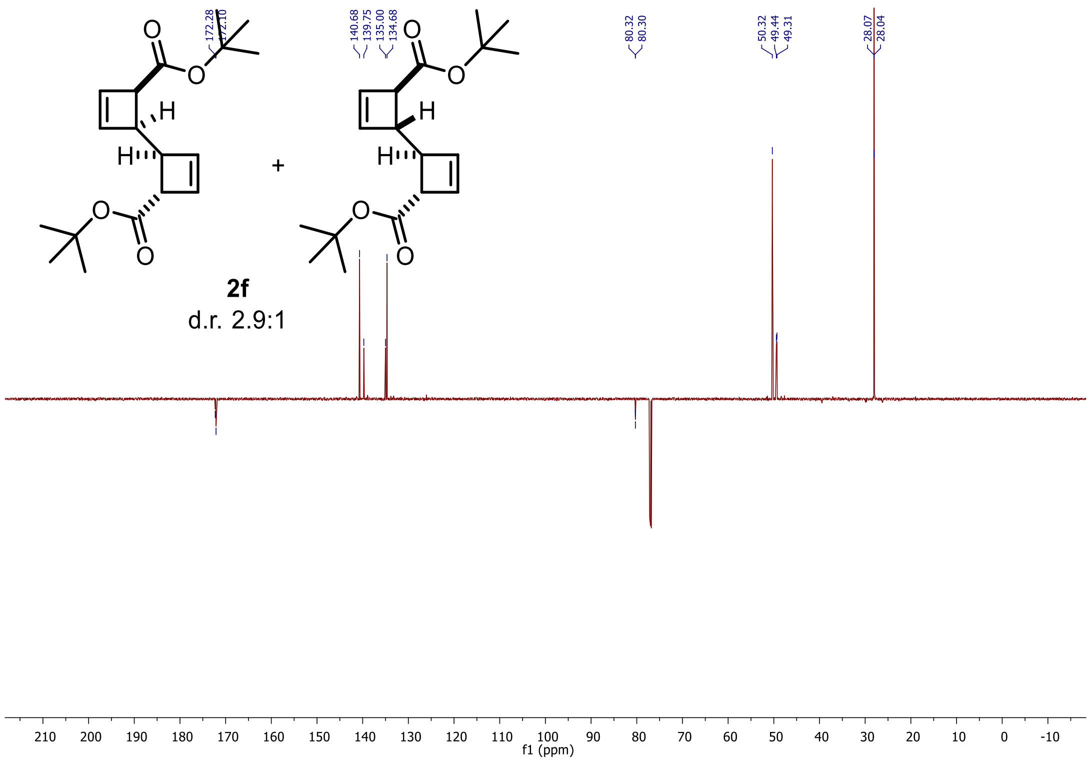

^1H NMR (700 MHz, $CDCl_3$)

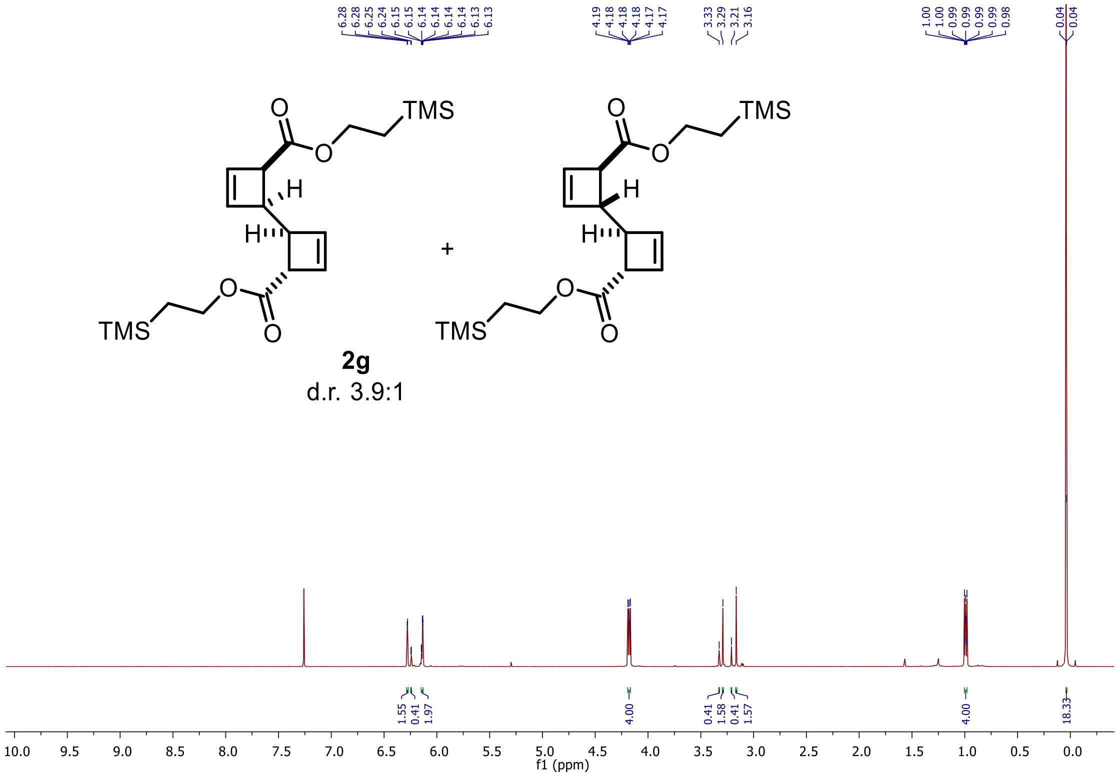

^{13}C NMR (176 MHz, $CDCl_3$)

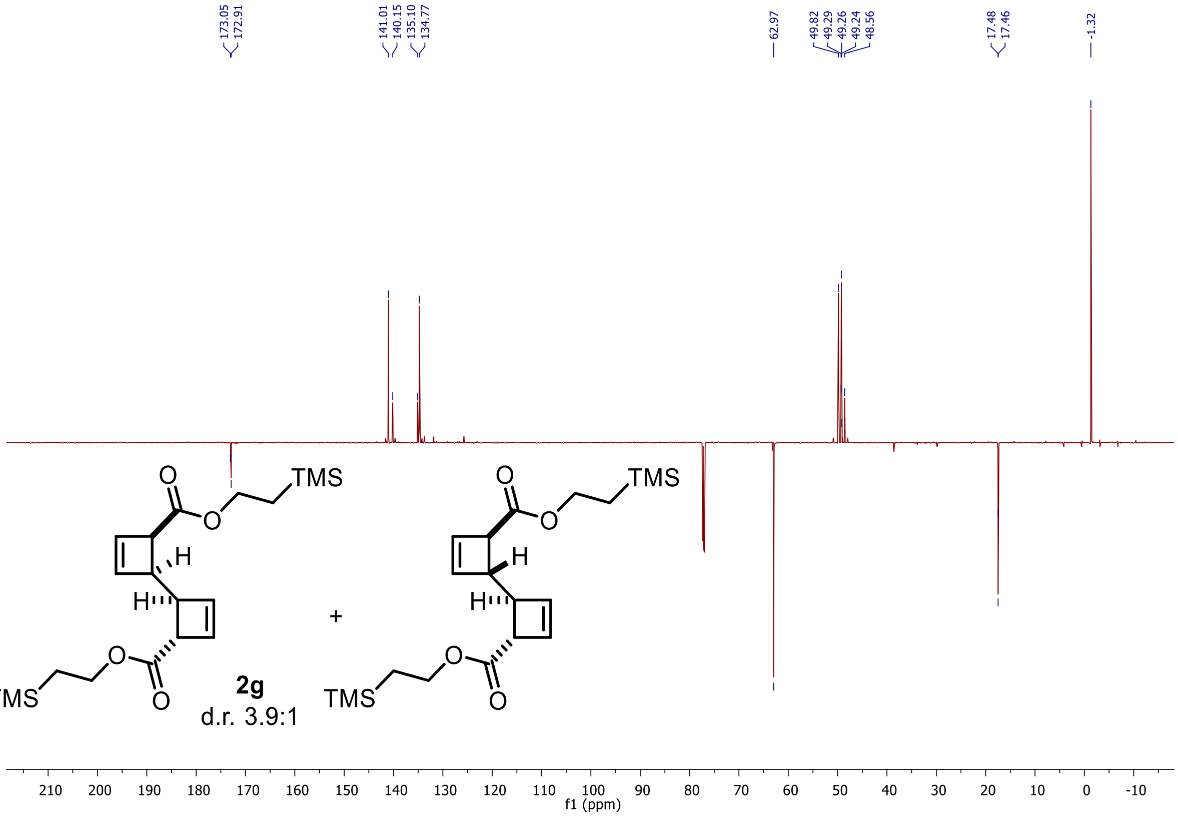

^{1}H NMR (700 MHz, $CDCl_3$)

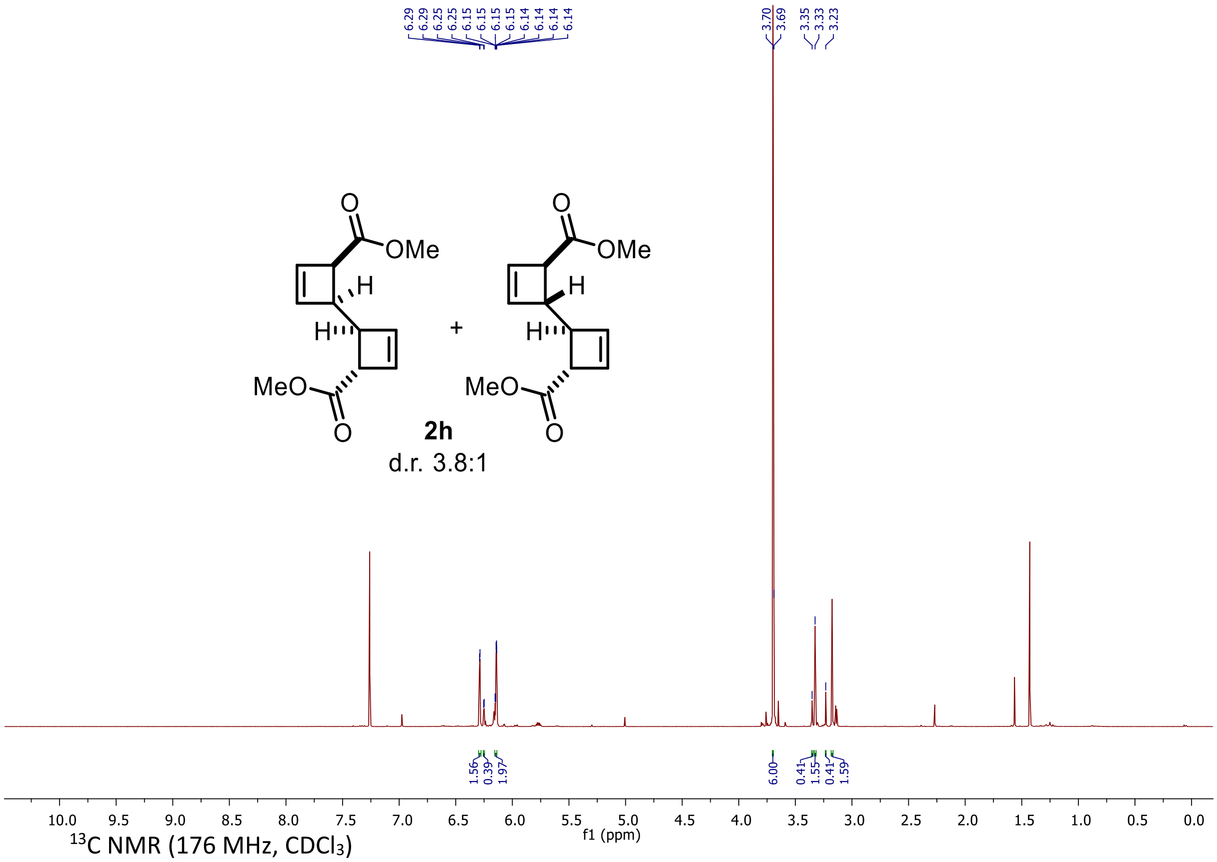

^{13}C NMR (176 MHz, $CDCl_3$)

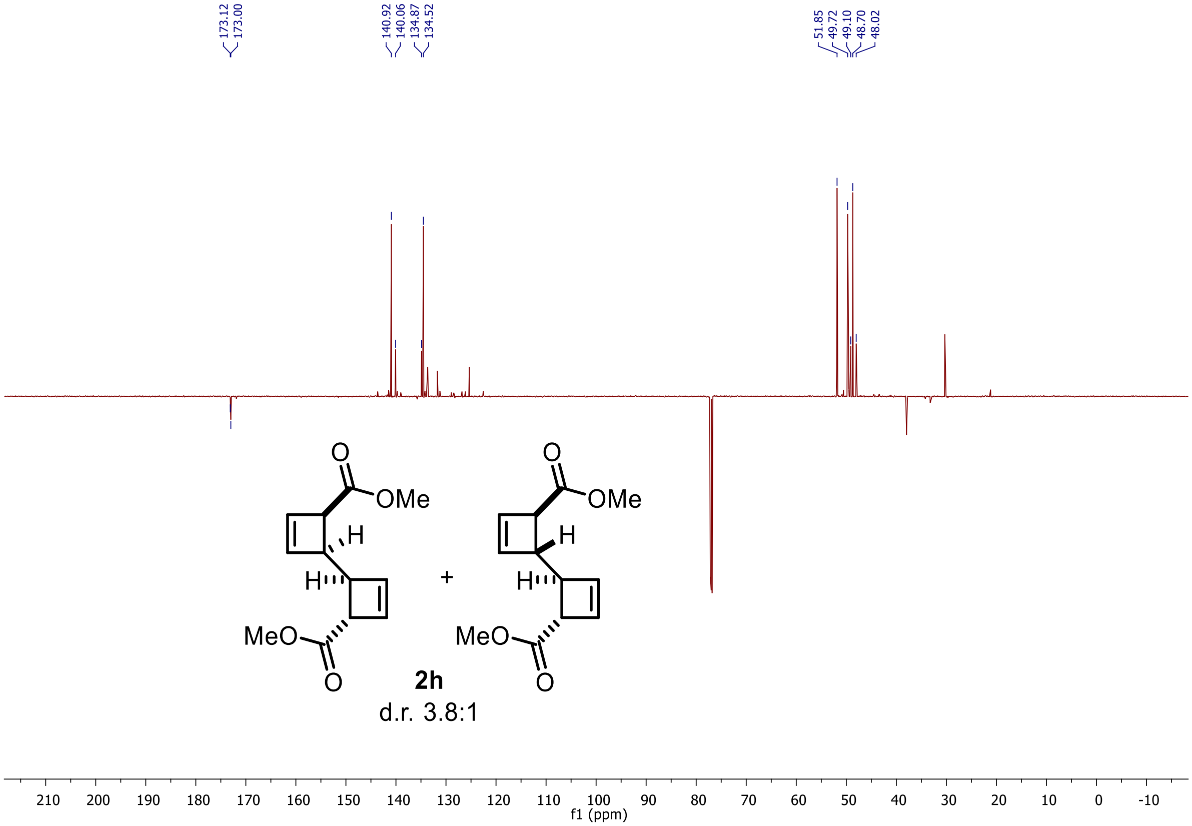

^{1}H NMR (600 MHz, $CDCl_3$)

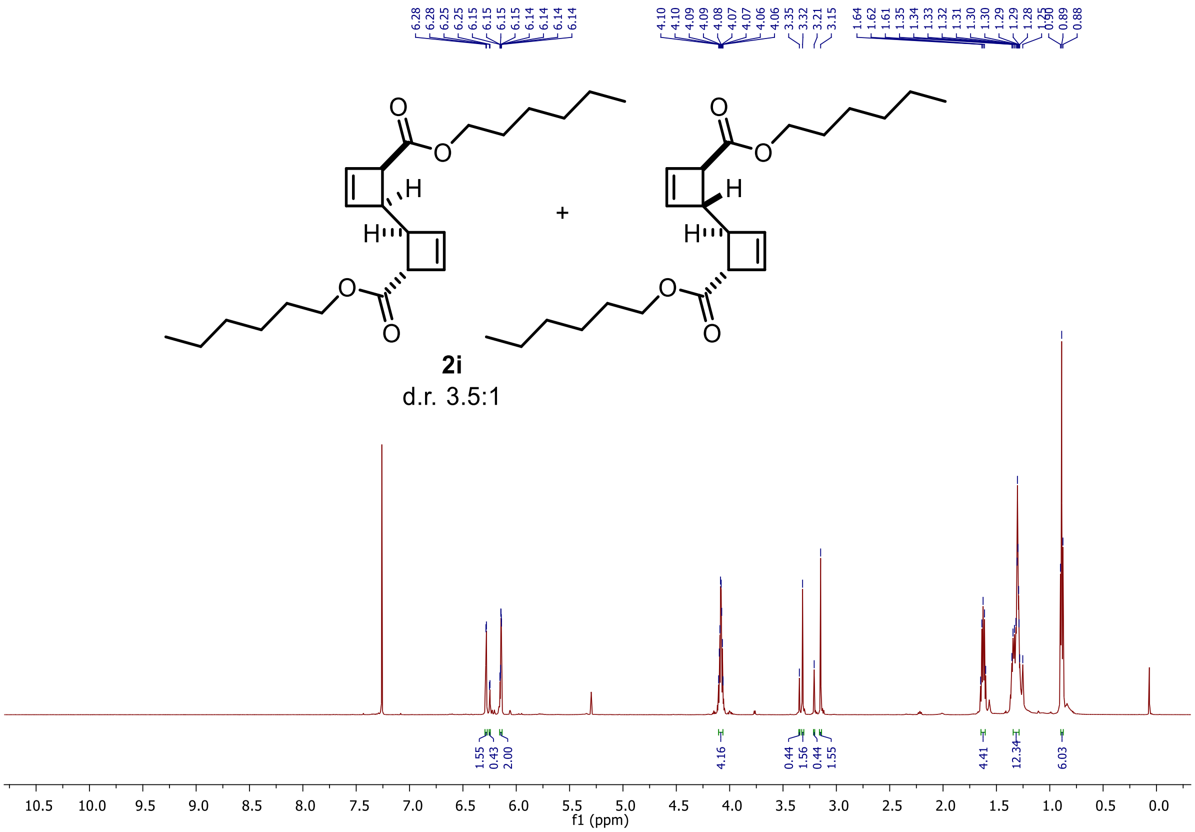

^{13}C NMR (151 MHz, $CDCl_3$)

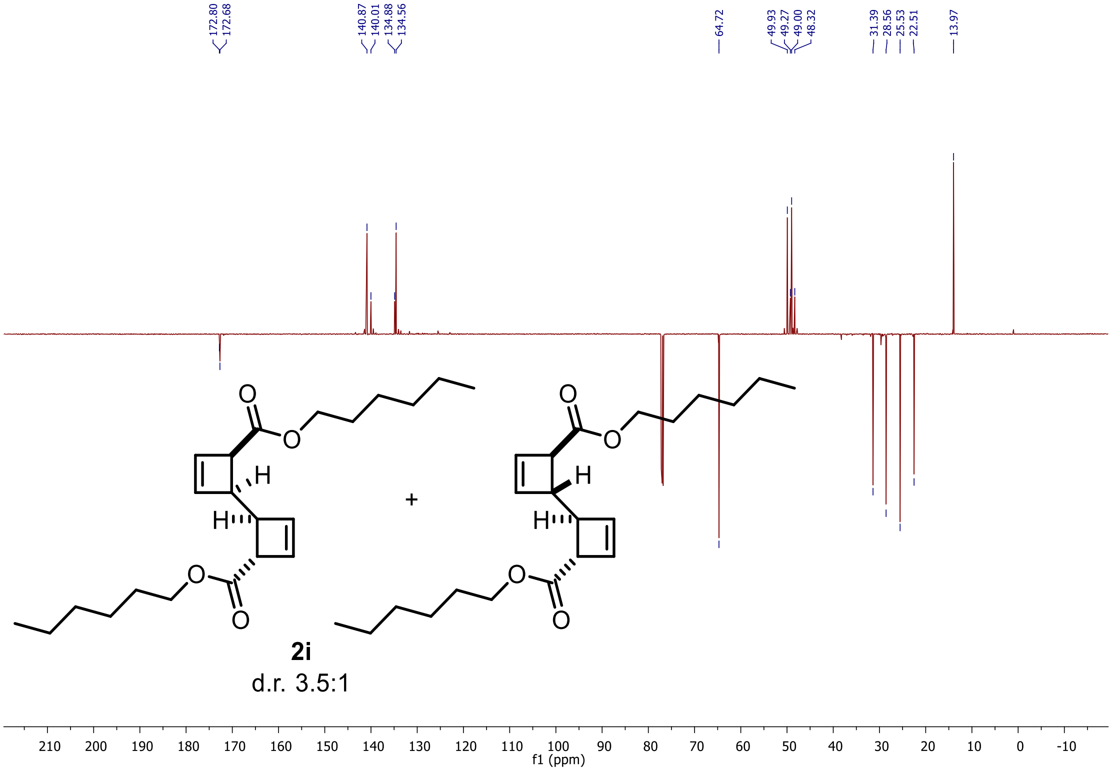

^{1}H NMR (700 MHz, $CDCl_3$)

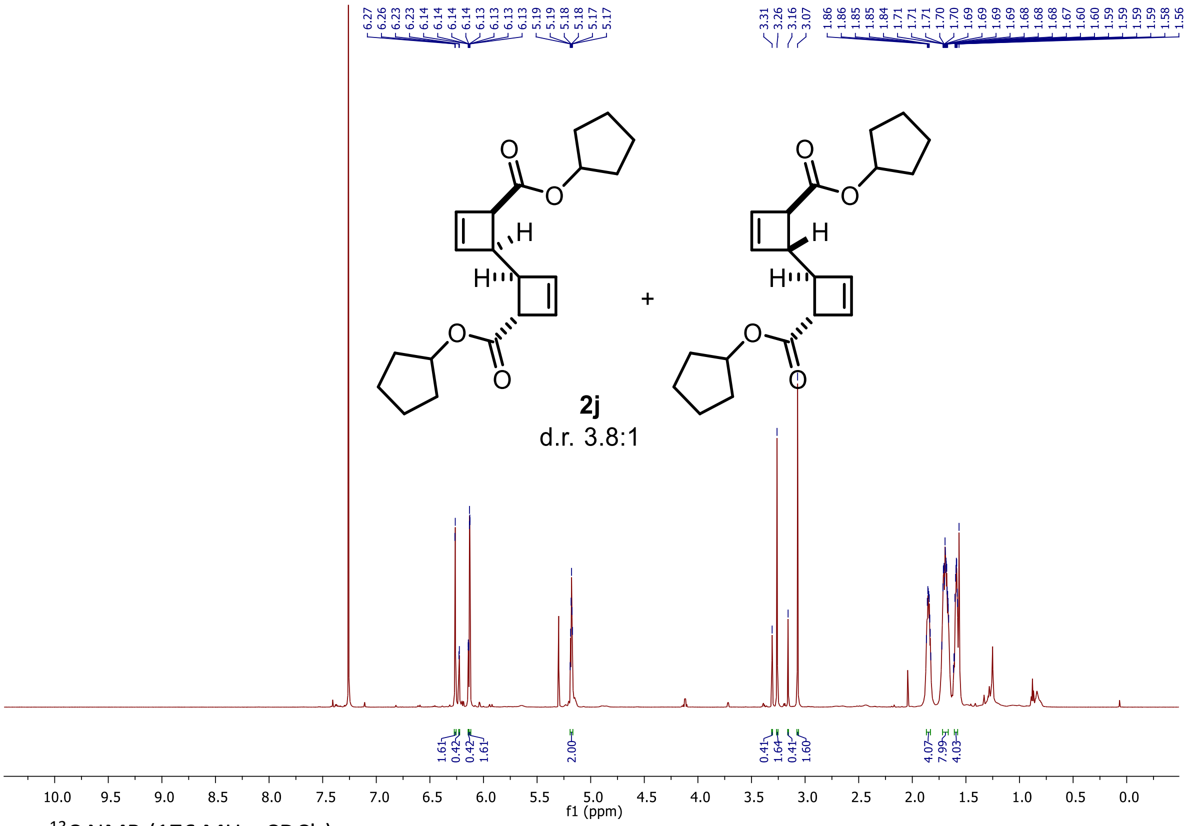

^{13}C NMR (176 MHz, $CDCl_3$)

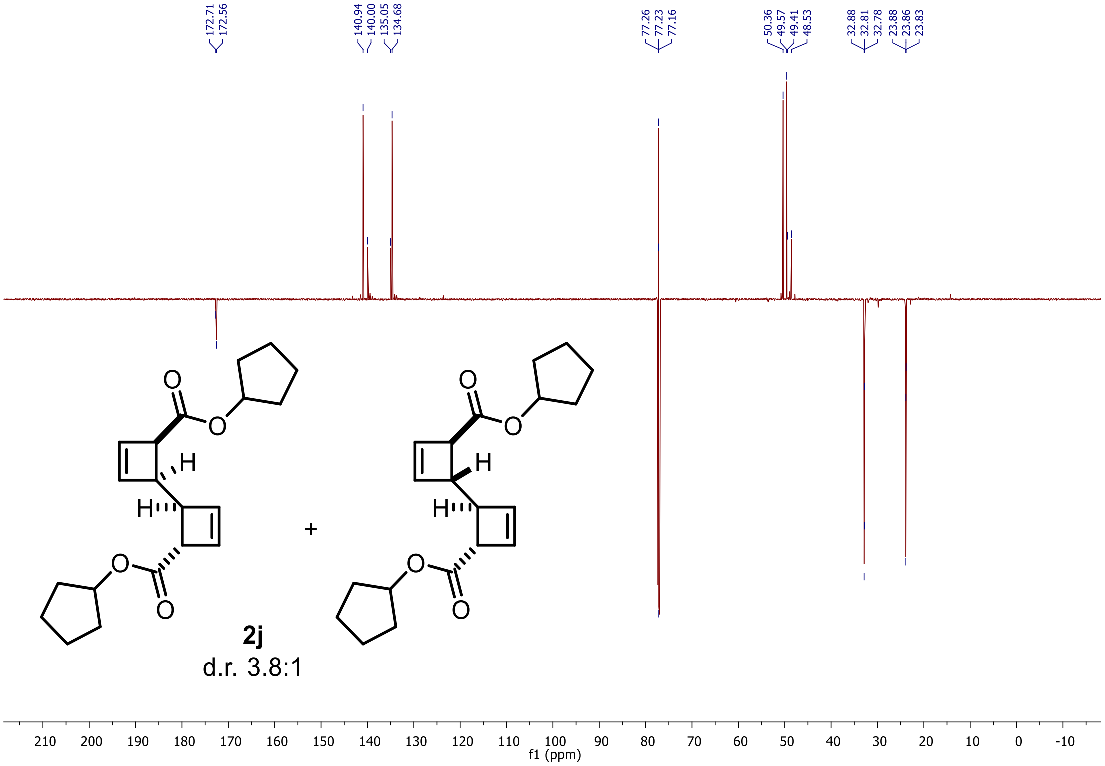

^{1}H NMR (400 MHz, $CDCl_3$)

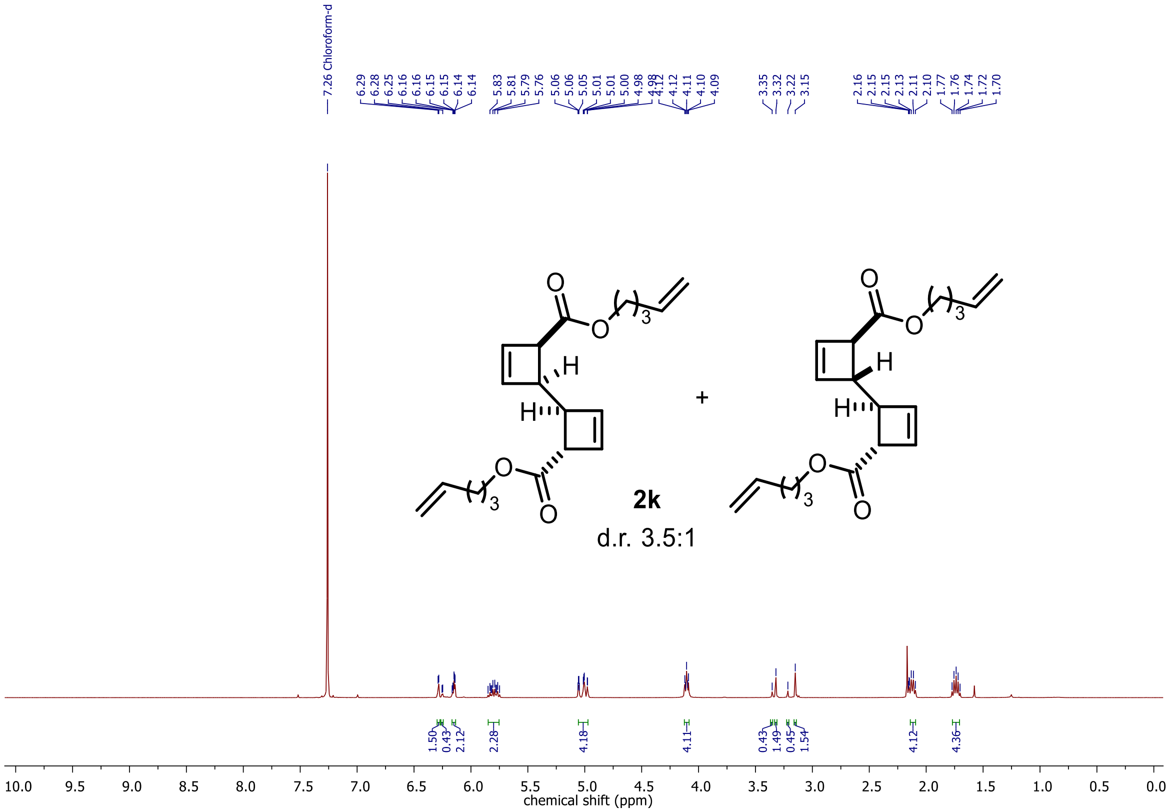

^{13}C NMR (101 MHz, $CDCl_3$)

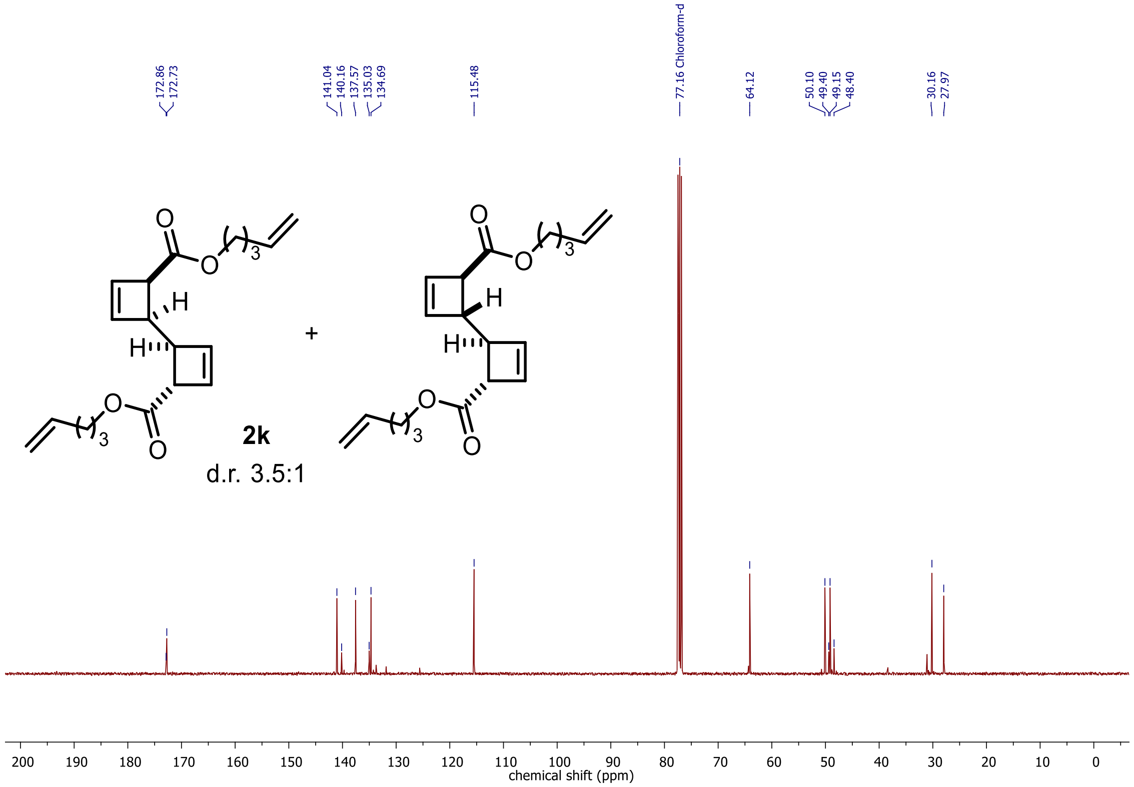

^{1}H NMR (500 MHz, $CDCl_3$)

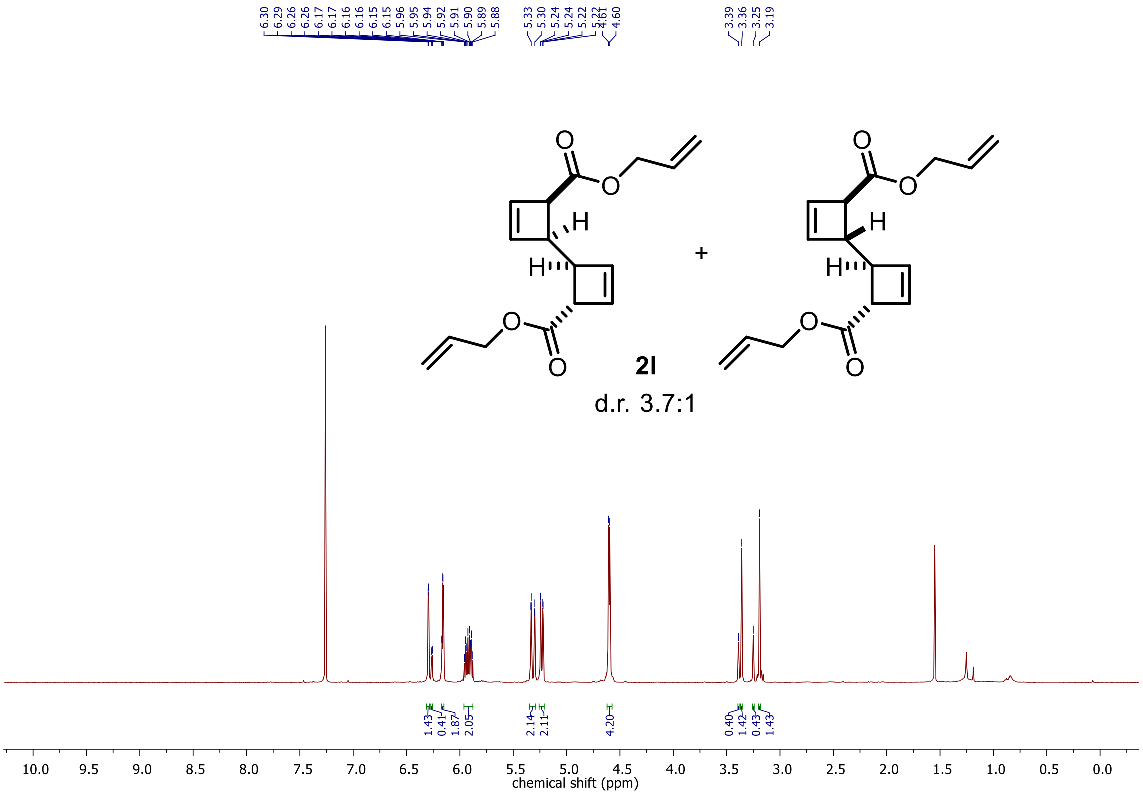

^{13}C NMR (126 MHz, $CDCl_3$)

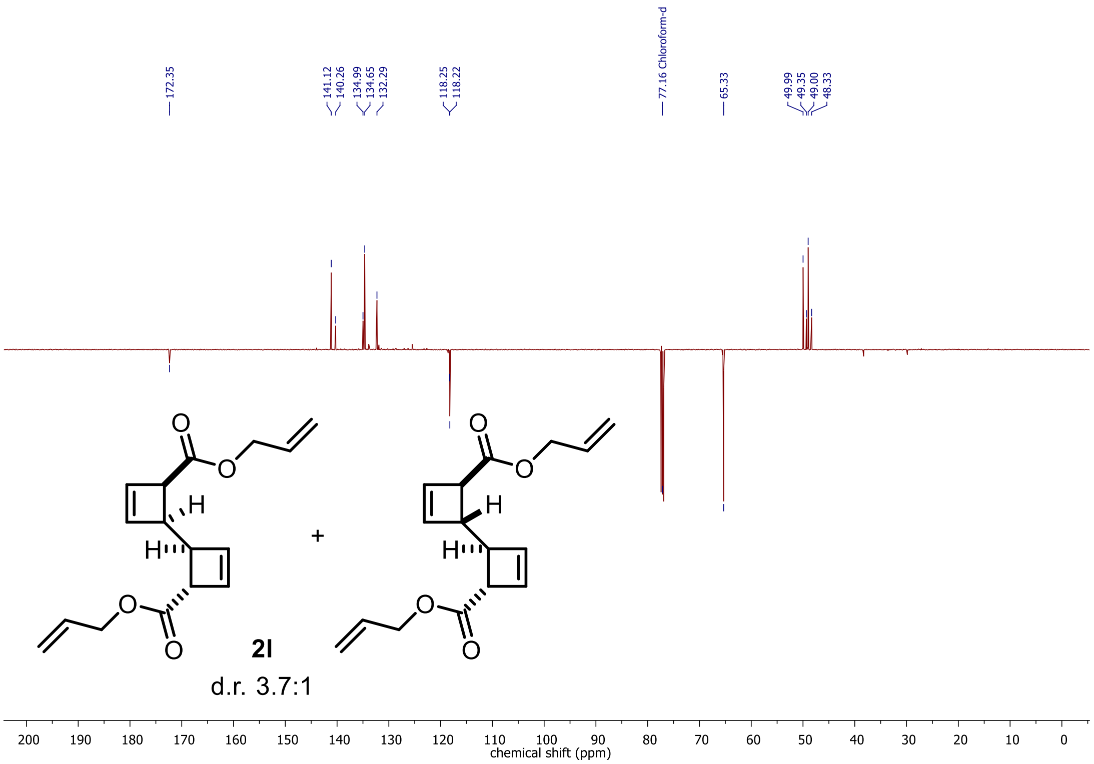

^{1}H NMR (600 MHz, $CDCl_3$)

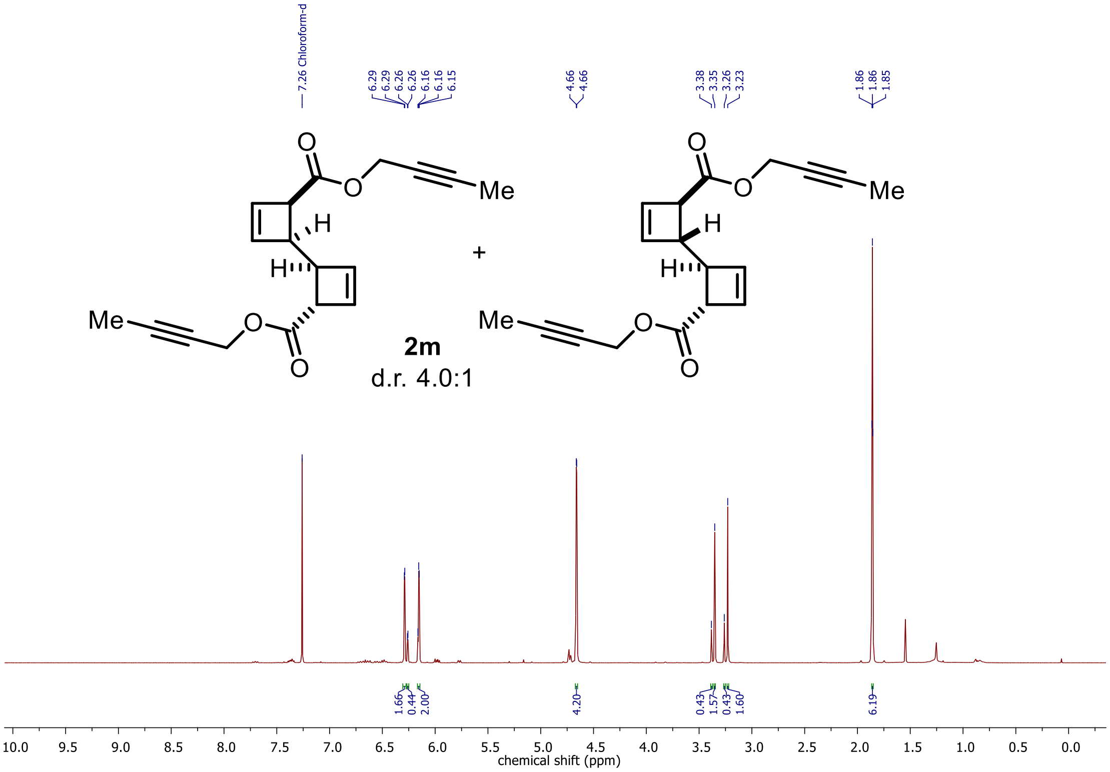

^{13}C NMR (101 MHz, $CDCl_3$)

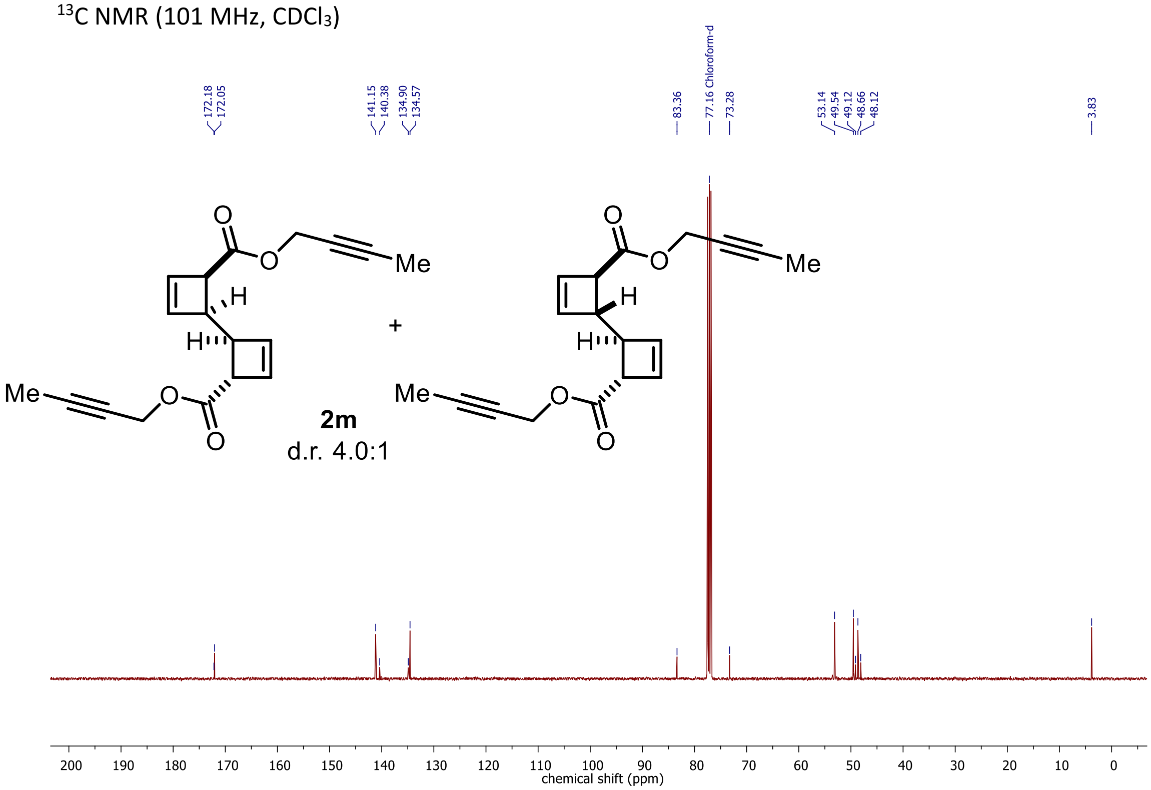

^{1}H NMR (600 MHz, $CDCl_3$)

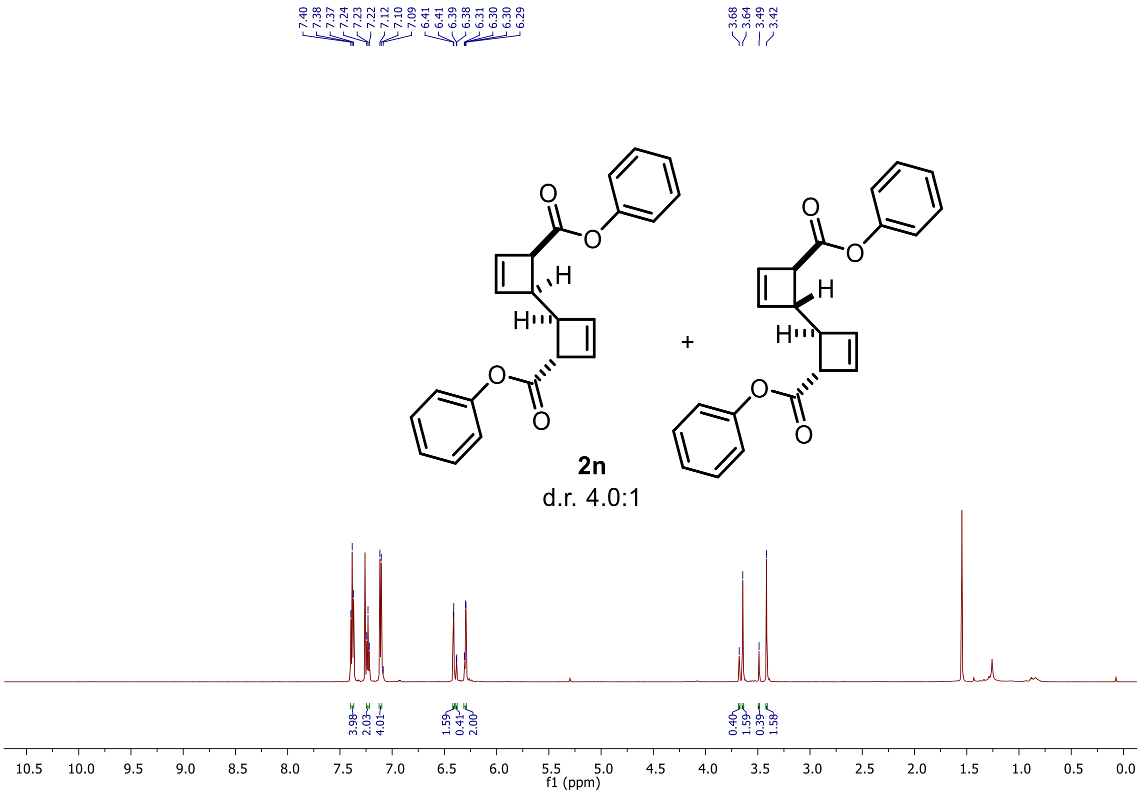

2n

d.r. 4.0:1

^{13}C NMR (151 MHz, $CDCl_3$)

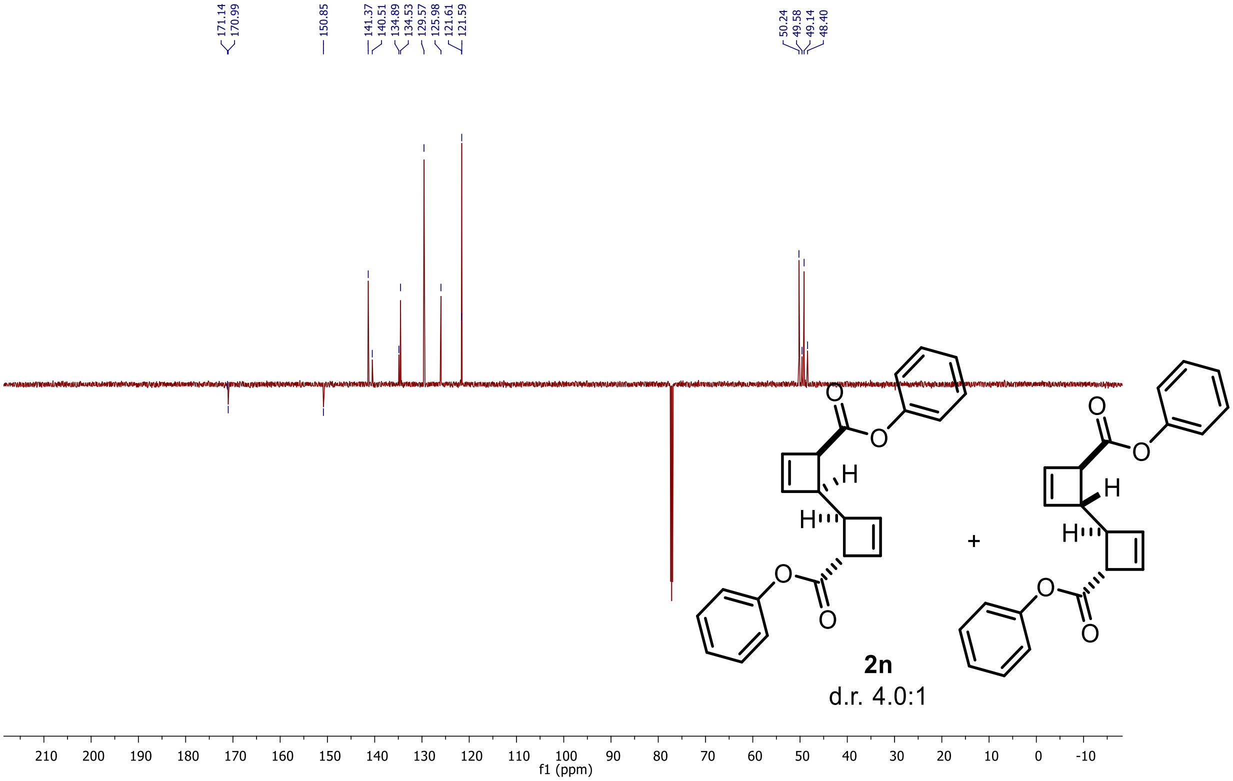

2n

d.r. 4.0:1

^{1}H NMR (700 MHz, $CDCl_3$)

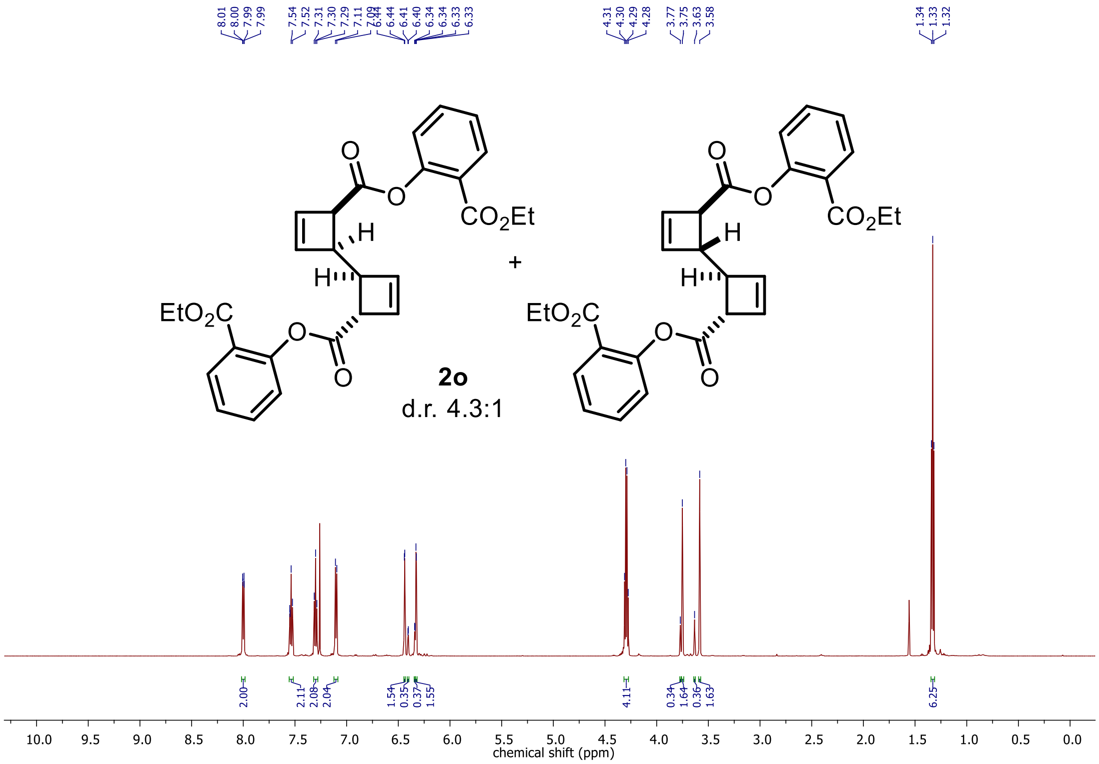

^{13}C NMR (151 MHz, $CDCl_3$)

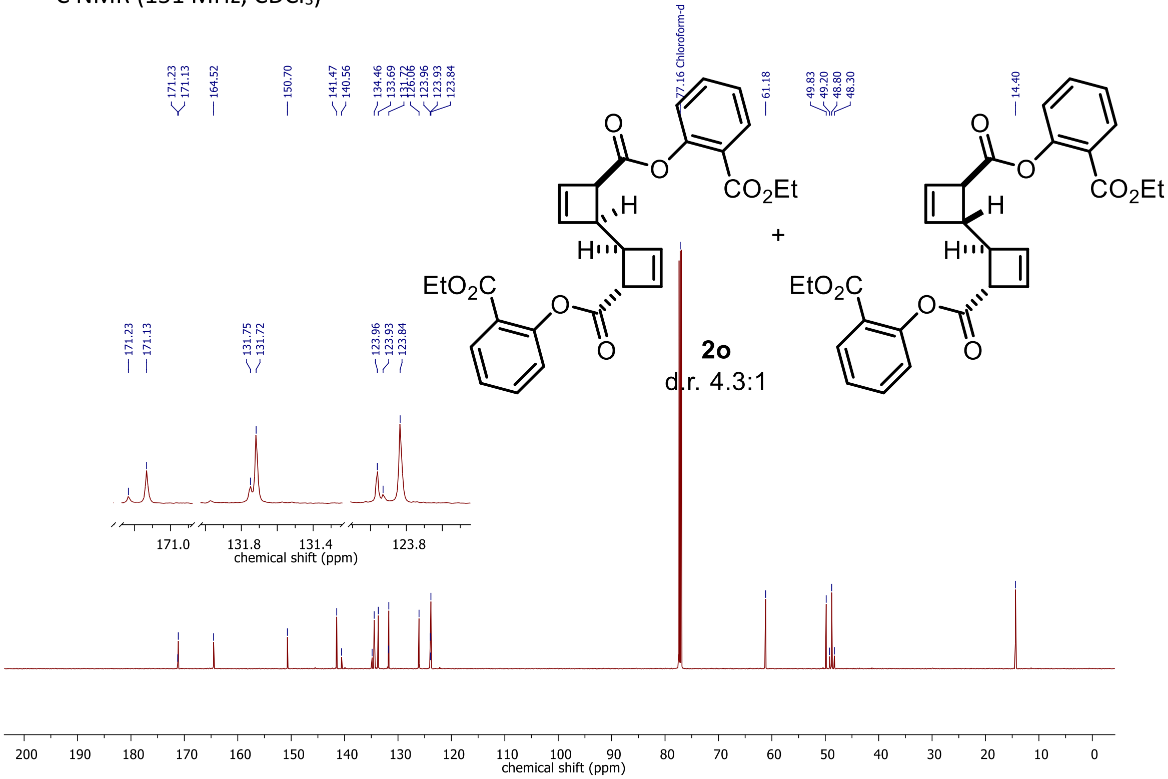

^1H NMR (400 MHz, $CDCl_3$)

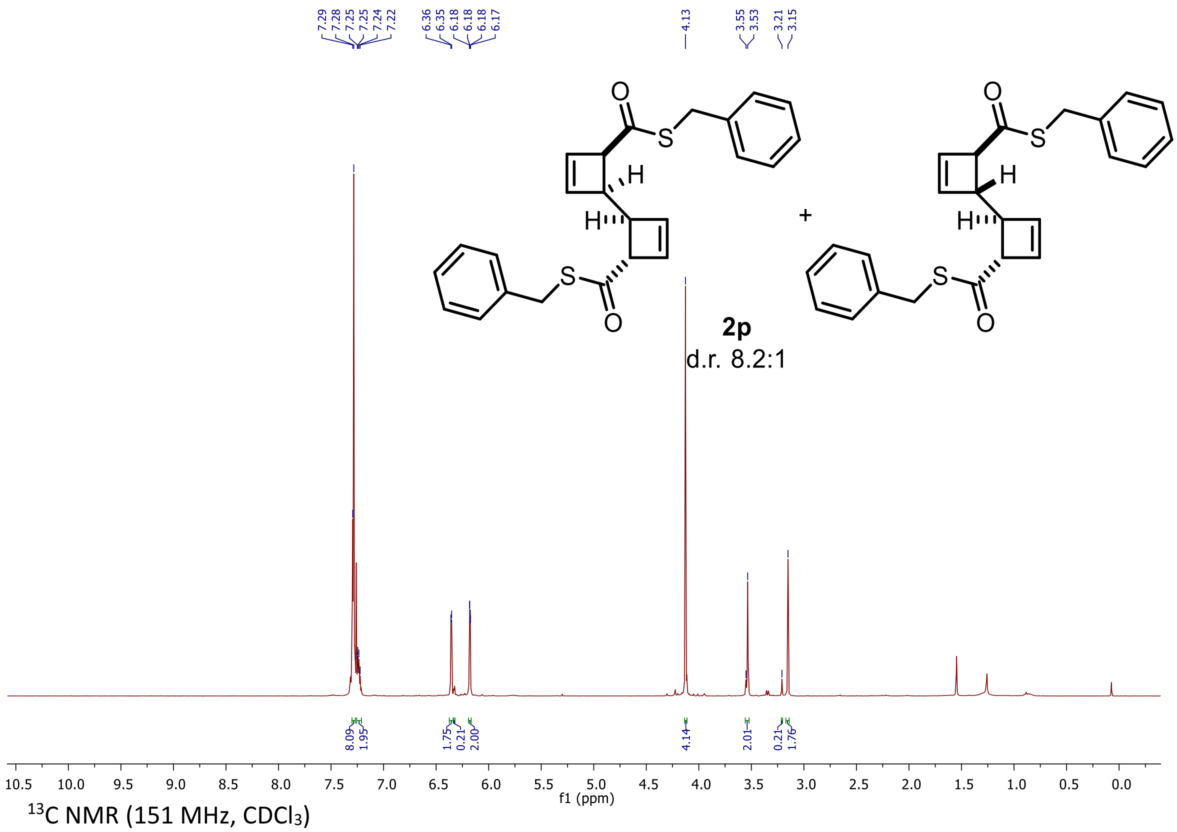

^{13}C NMR (151 MHz, $CDCl_3$)

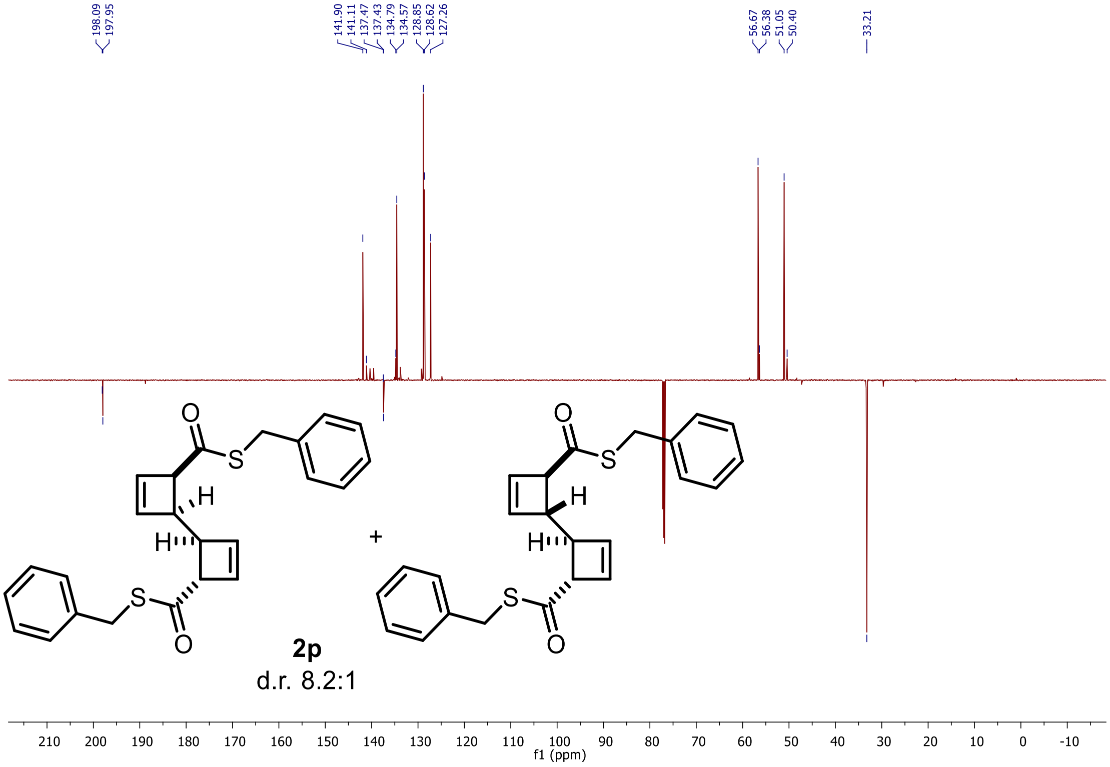

^{1}H NMR (700 MHz, $CDCl_3$)

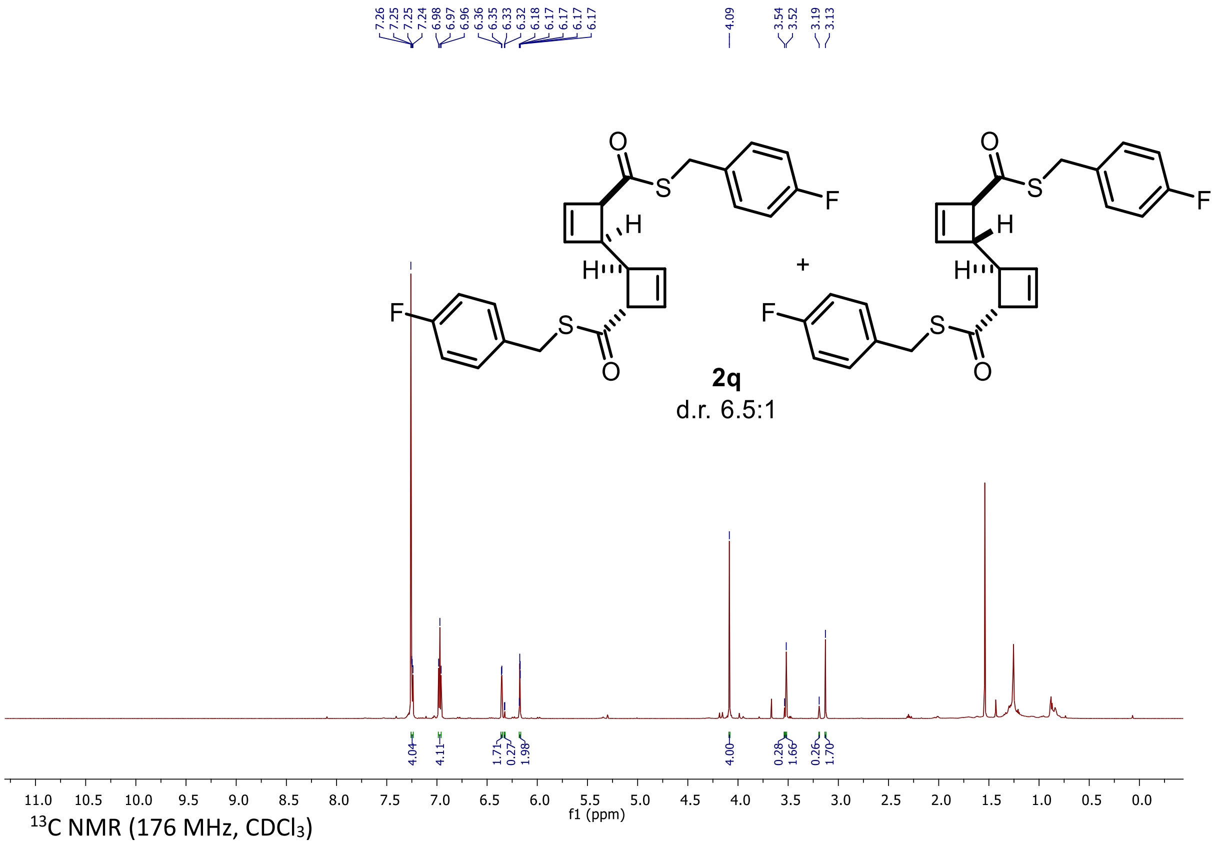

^{13}C NMR (176 MHz, $CDCl_3$)

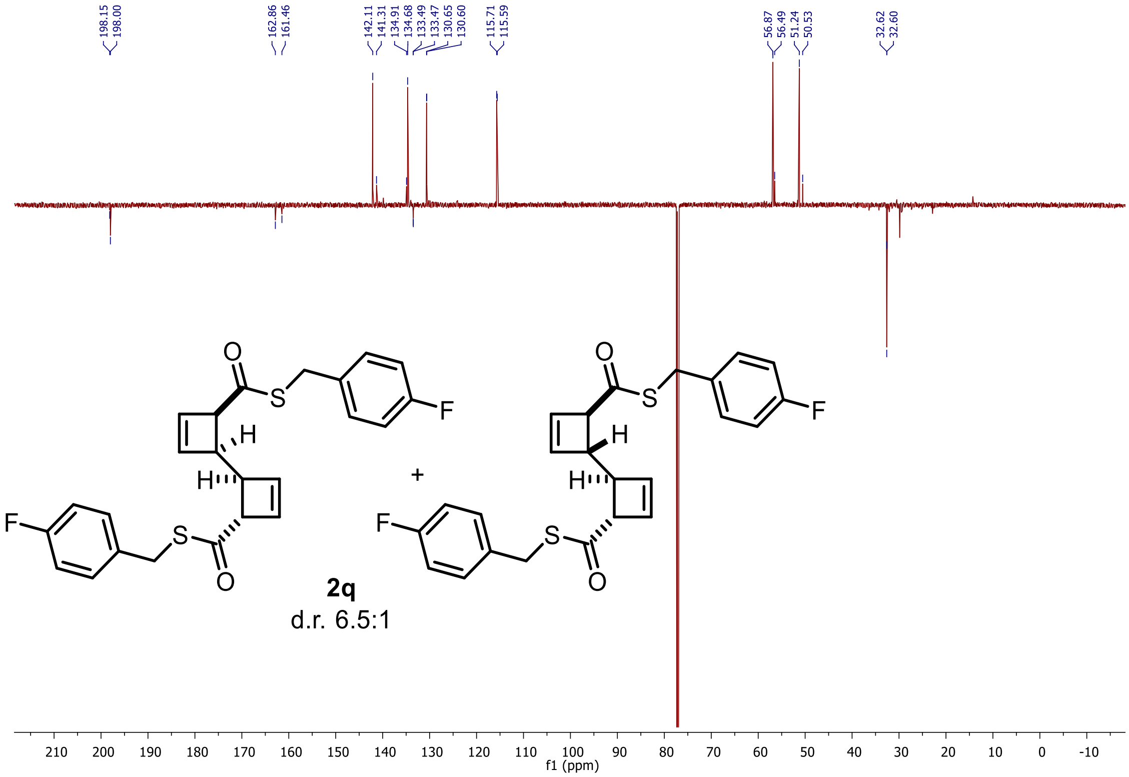

^{19}F NMR (377 MHz, CDCl$_3$)

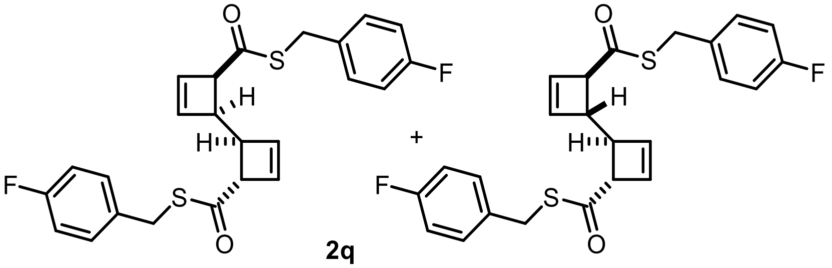

2q
d.r. 6.5:1

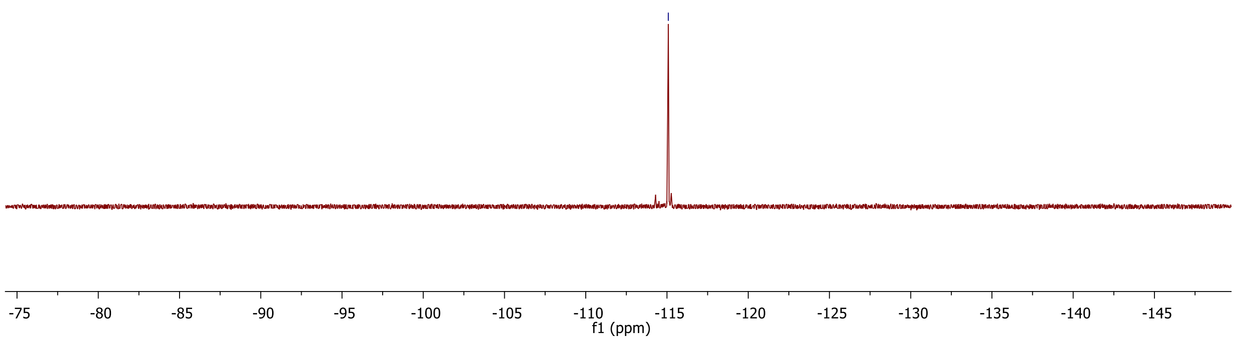

^{1}H NMR (600 MHz, $CDCl_3$)

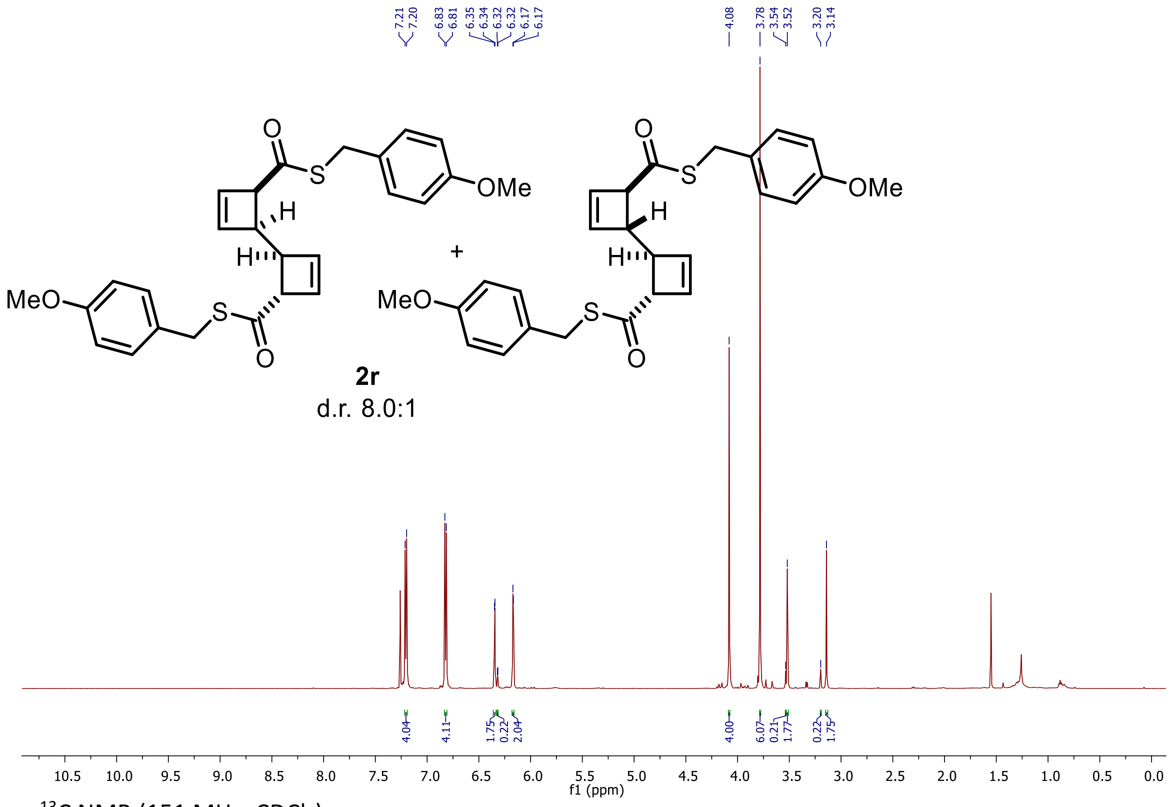

^{13}C NMR (151 MHz, $CDCl_3$)

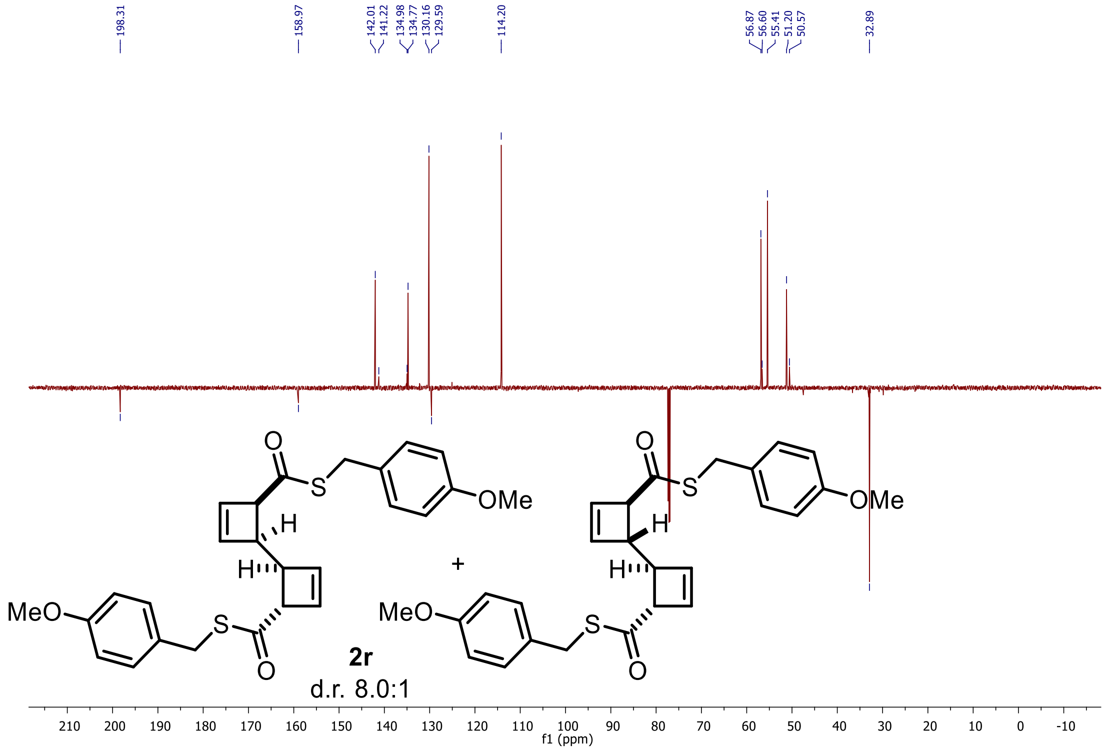

^{1}H NMR (400 MHz, $CDCl_3$)

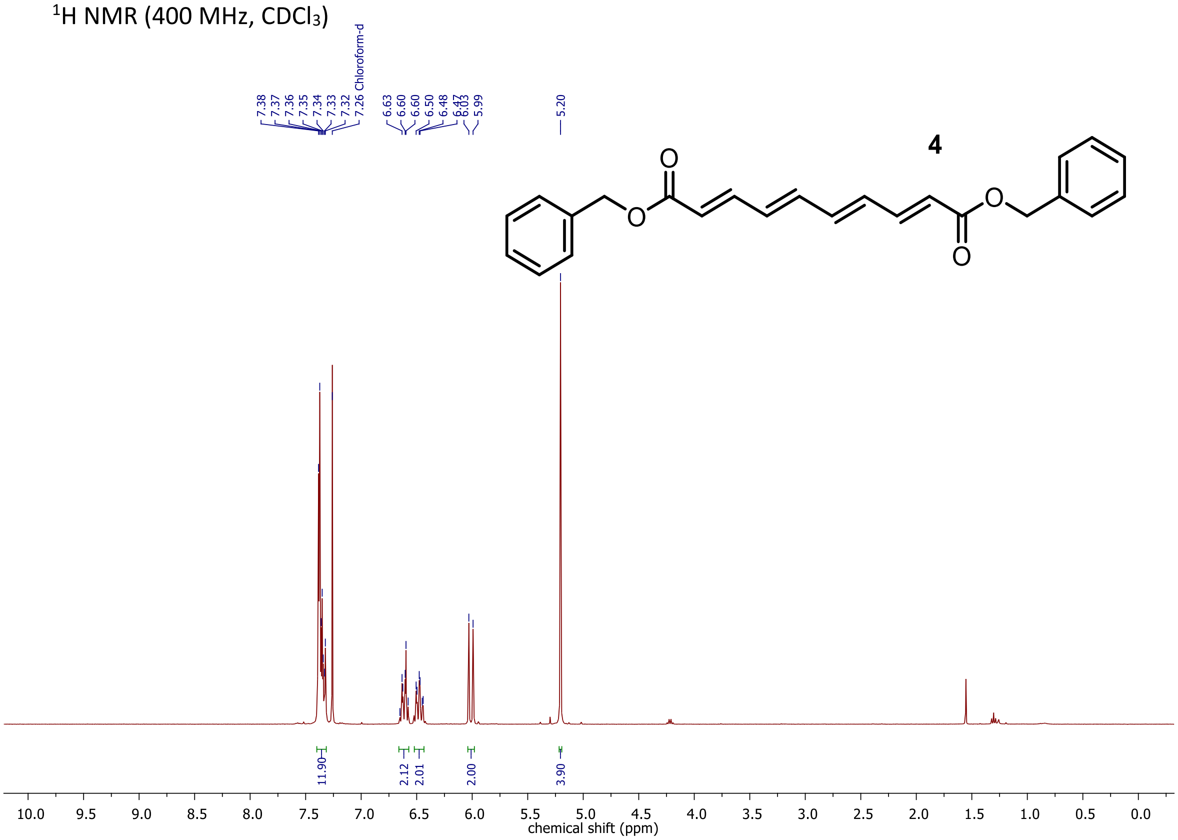

^{13}C NMR (101 MHz, $CDCl_3$)

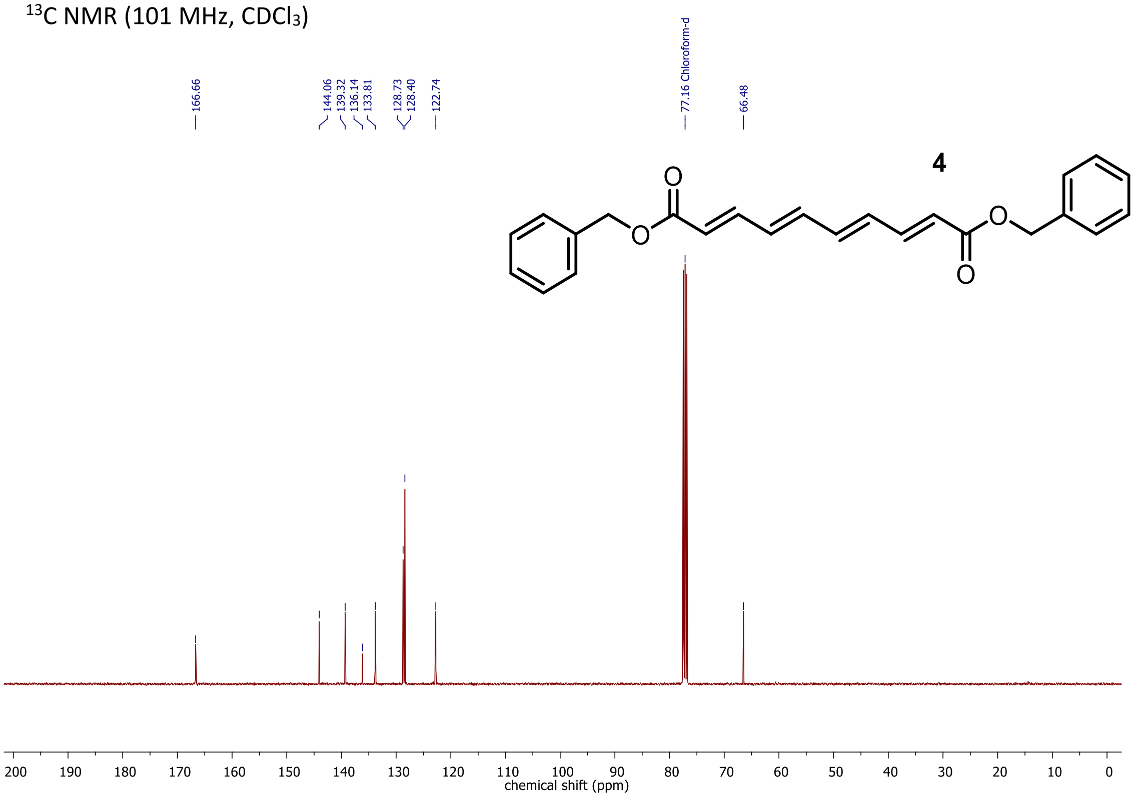

^{1}H NMR (700 MHz, $CDCl_3$)

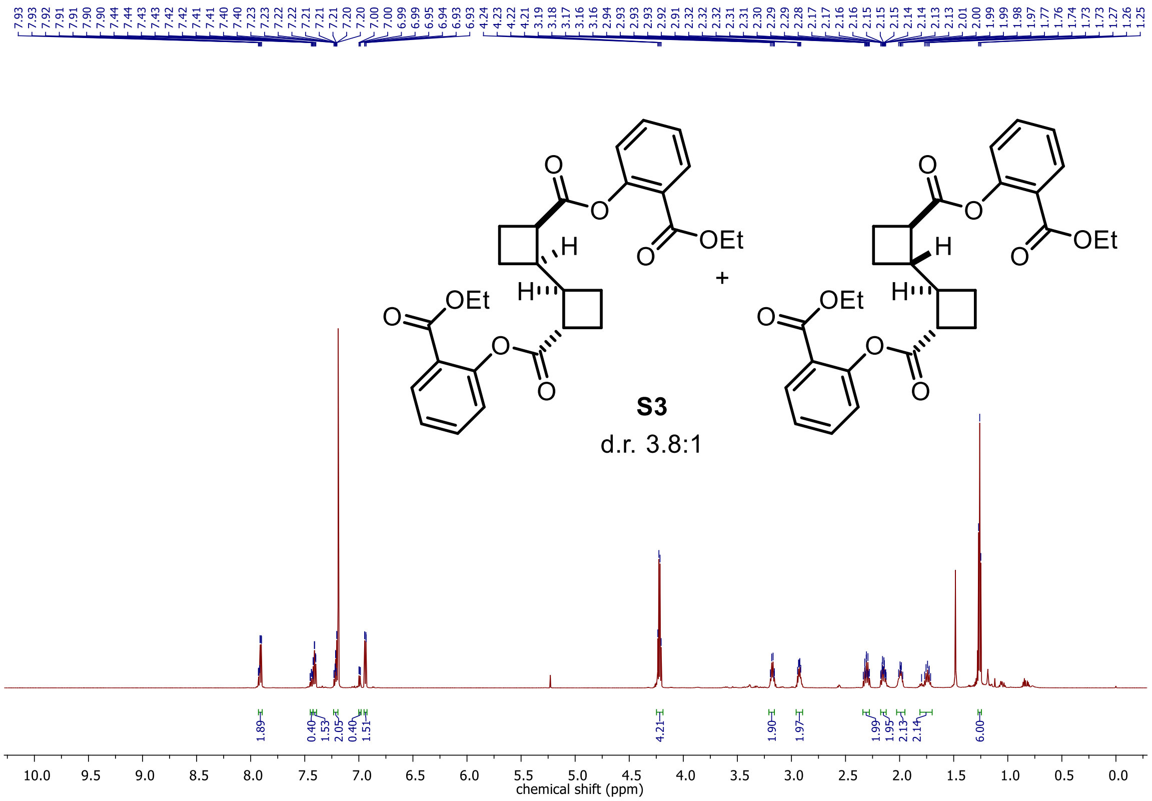

^{13}C NMR (176 MHz, $CDCl_3$)

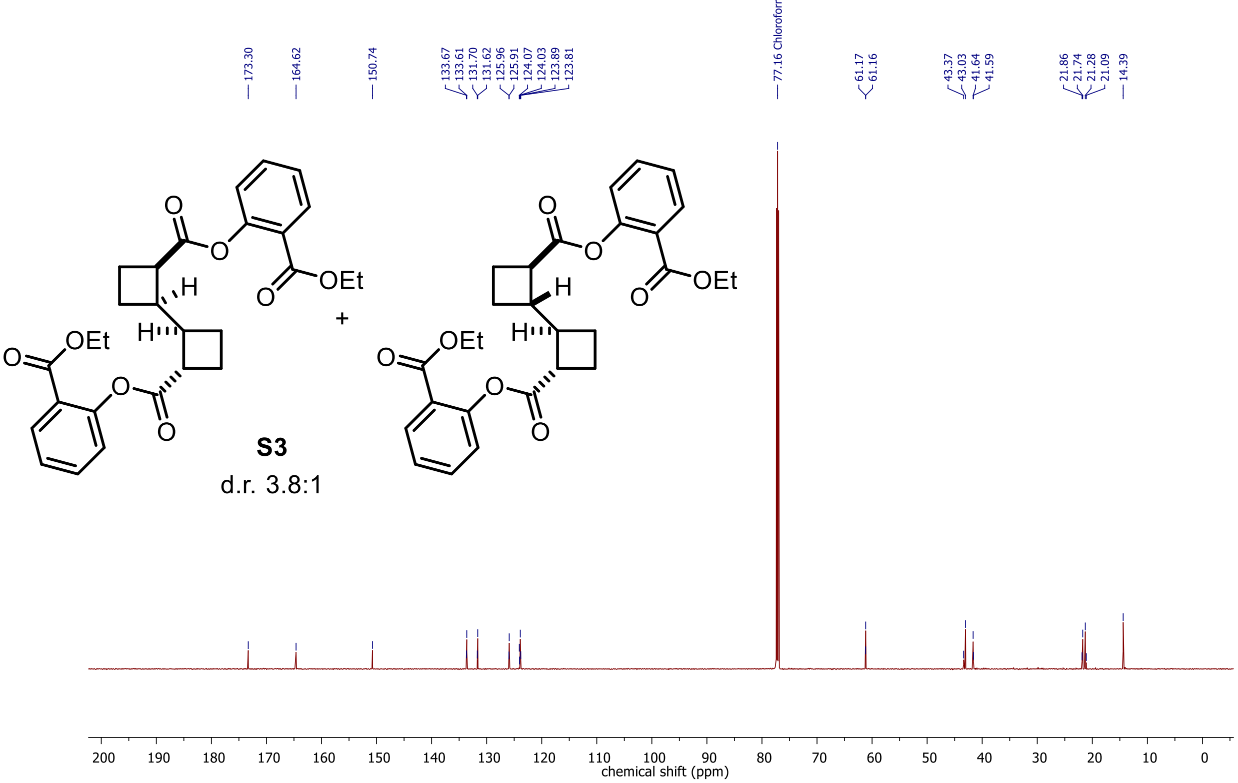

^1H NMR (600 MHz, MeOD)

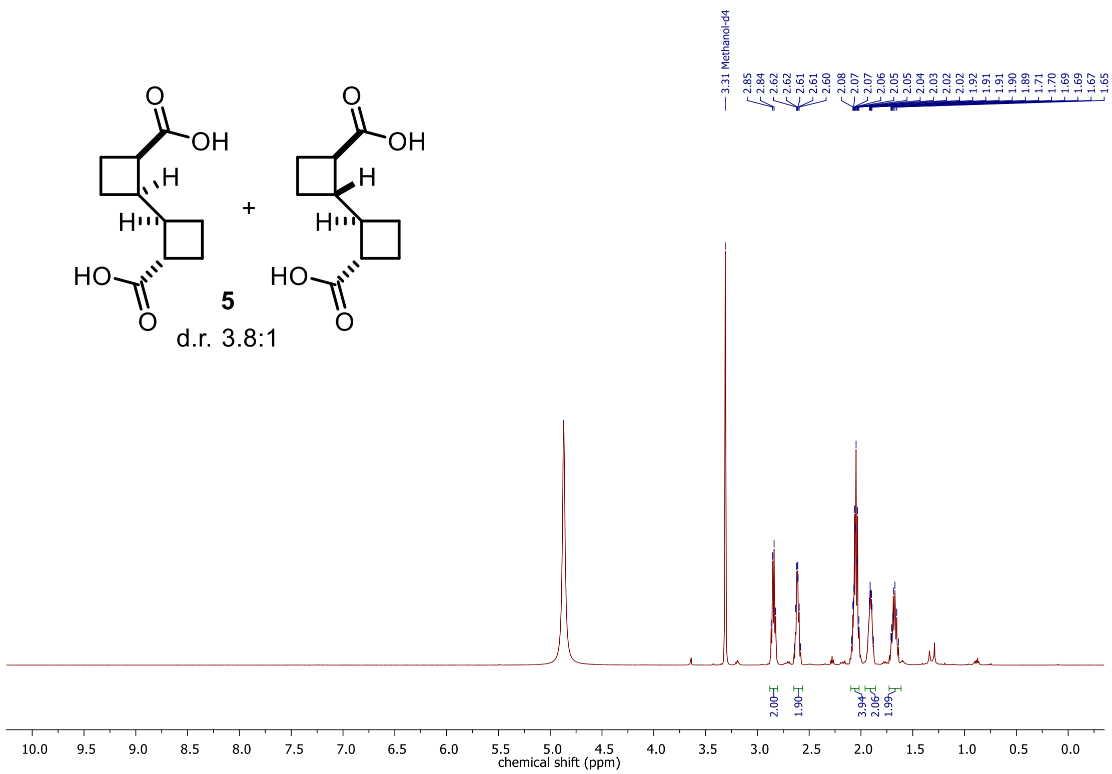

^{13}C NMR (151 MHz, MeOD)

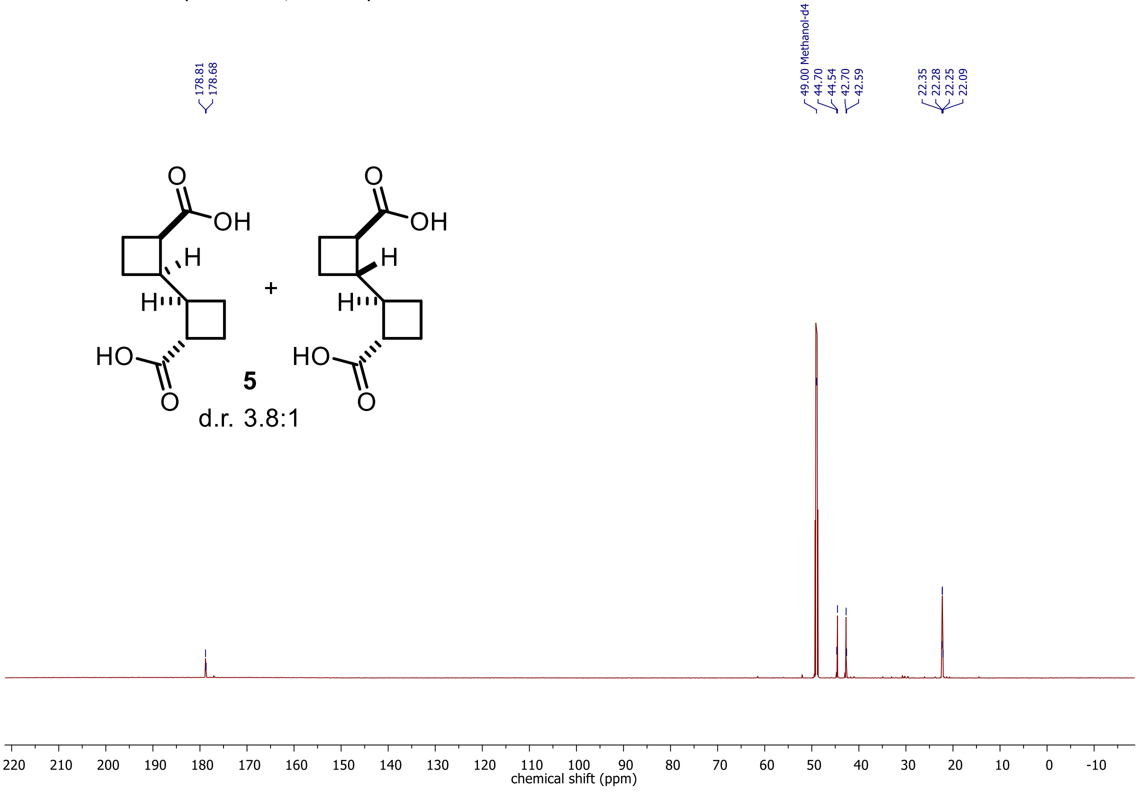

^{1}H NMR (600 MHz, acetone-d_6)

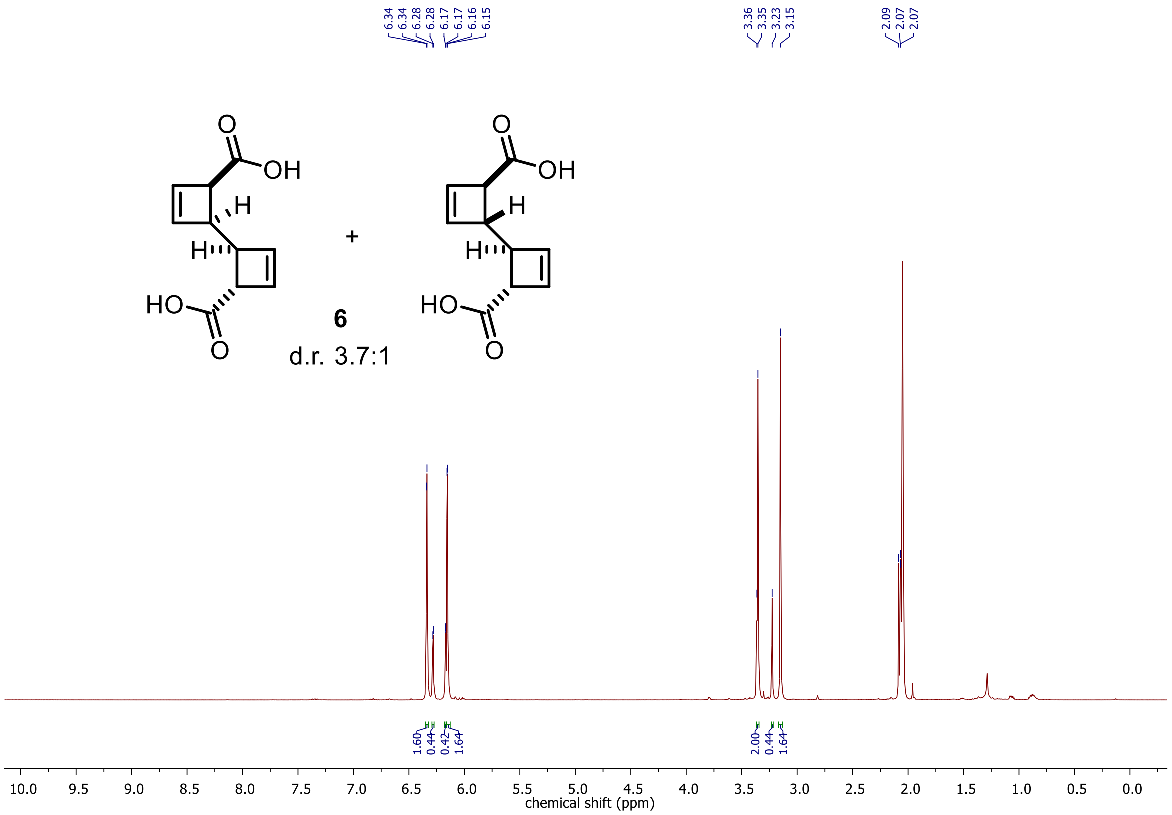

^{13}C NMR (151 MHz, acetone-d_6)

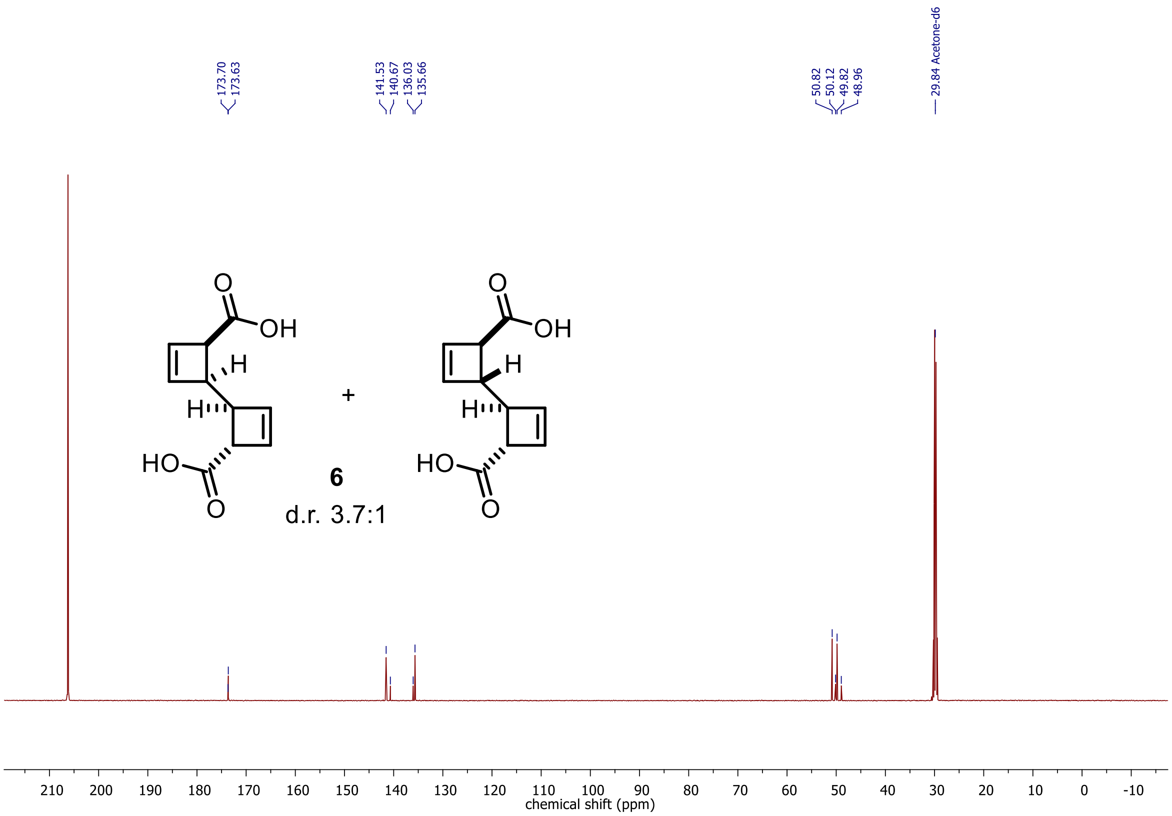

^{1}H NMR (600 MHz, $CDCl_3$)

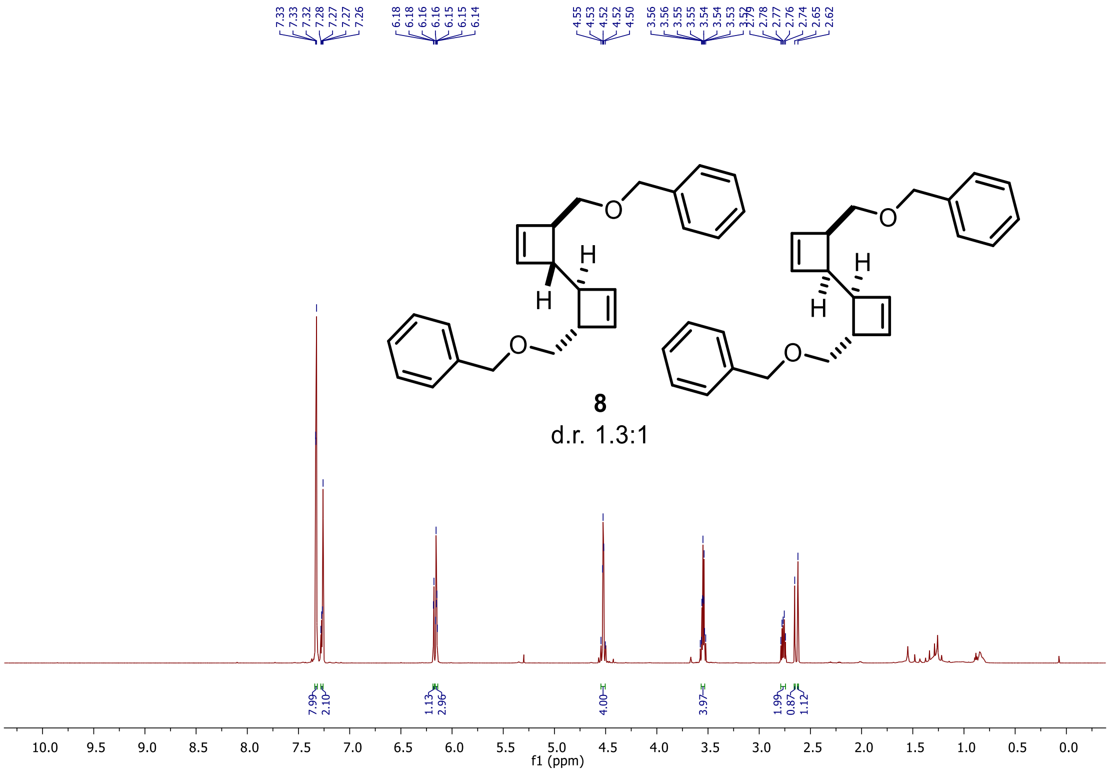

^{13}C NMR (151 MHz, $CDCl_3$)

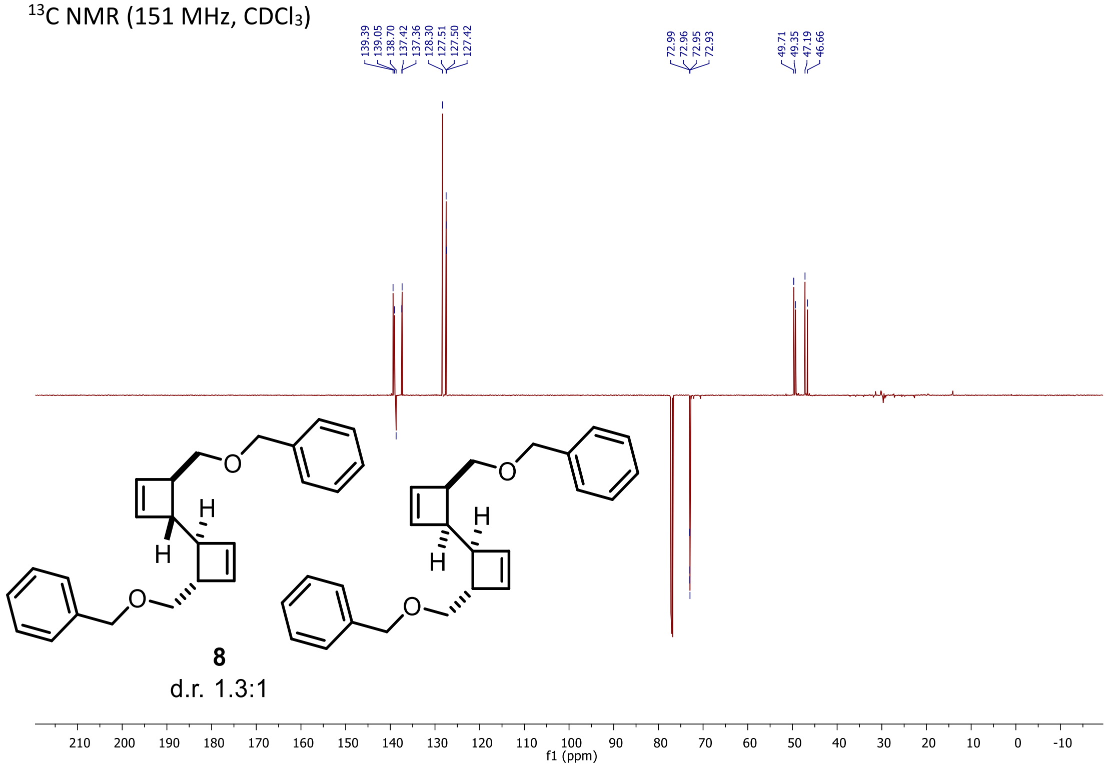

^1H NMR (600 MHz, $CDCl_3$)

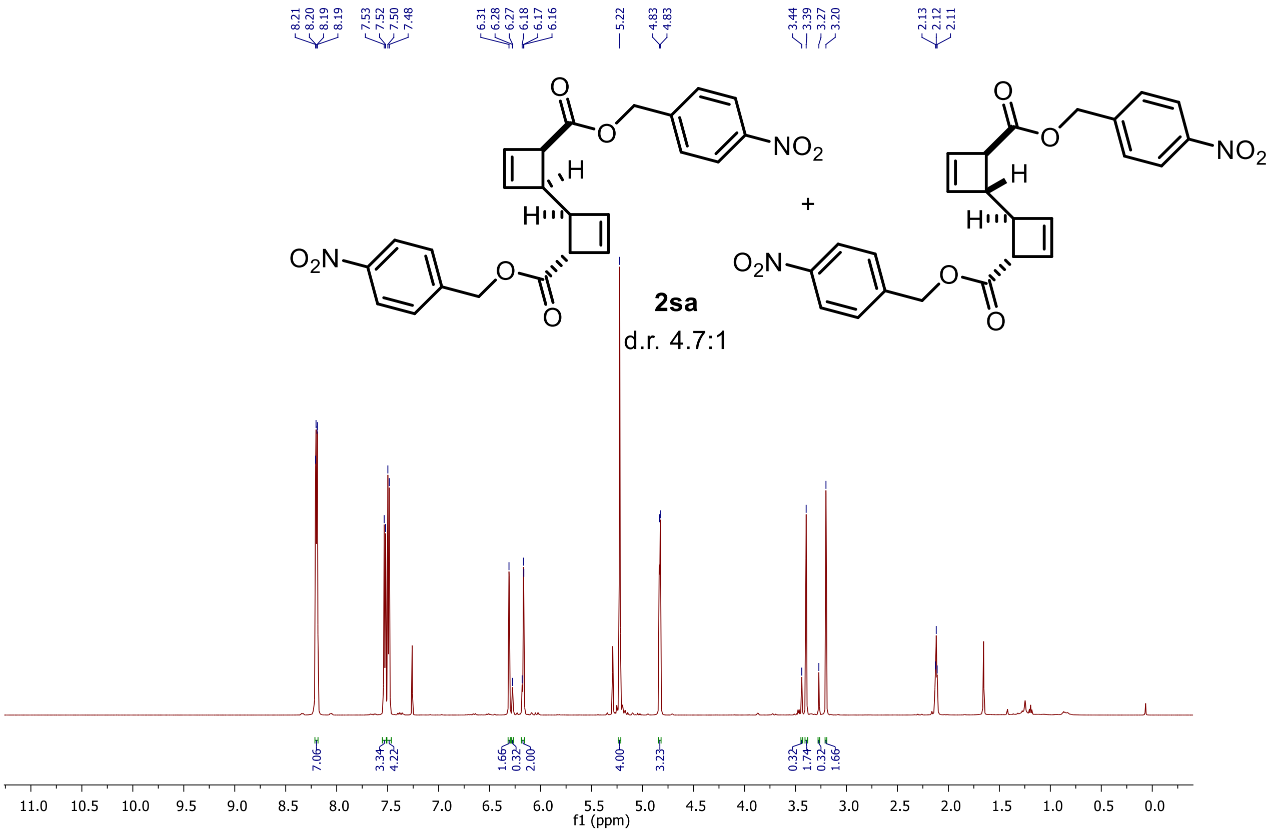

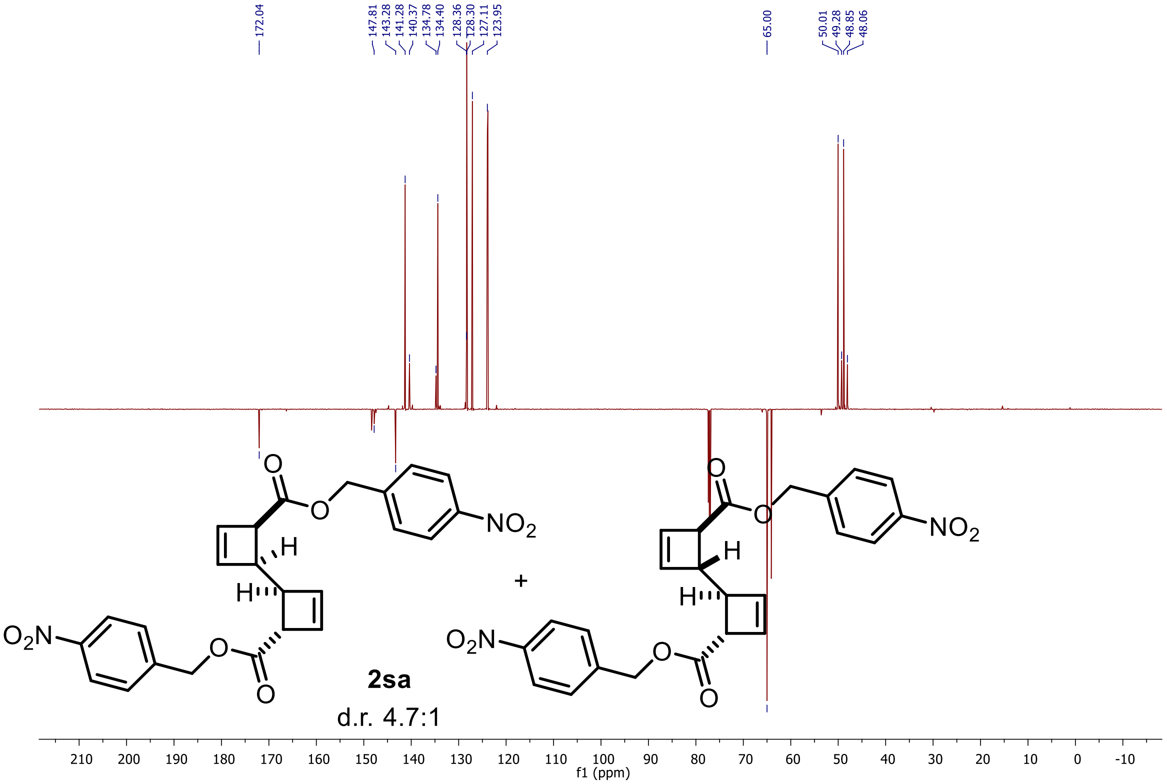

^{1}H NMR (400 MHz, $CDCl_3$)

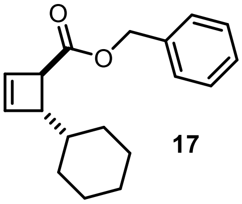

17

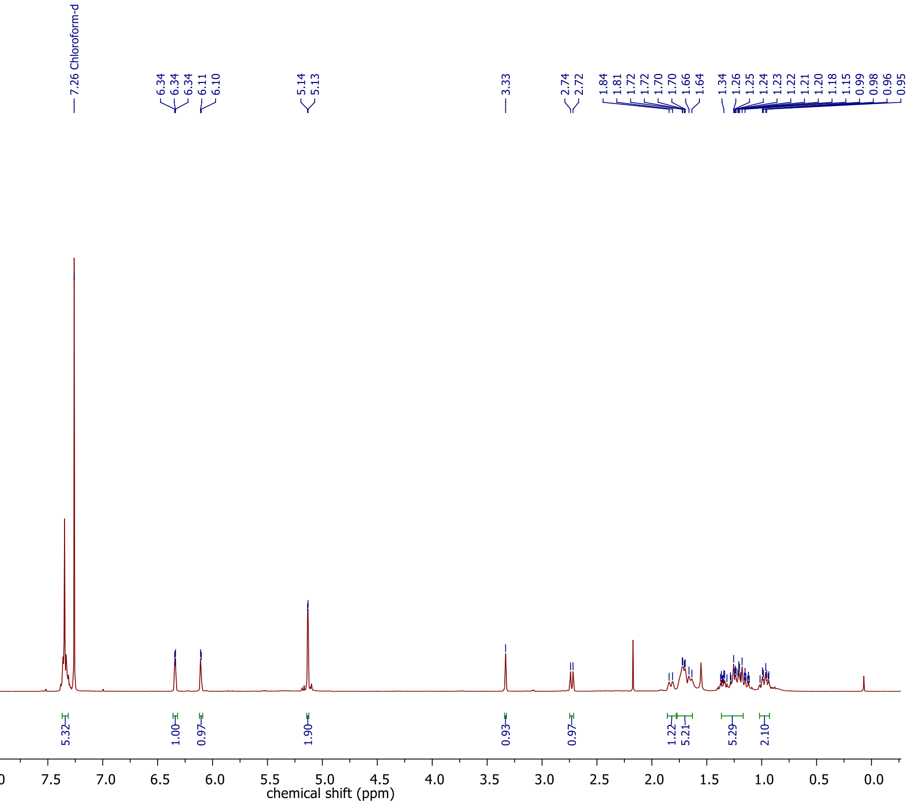

^{13}C NMR (101 MHz, $CDCl_3$)

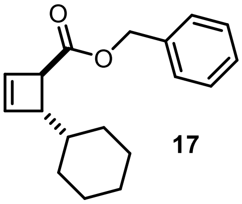

17

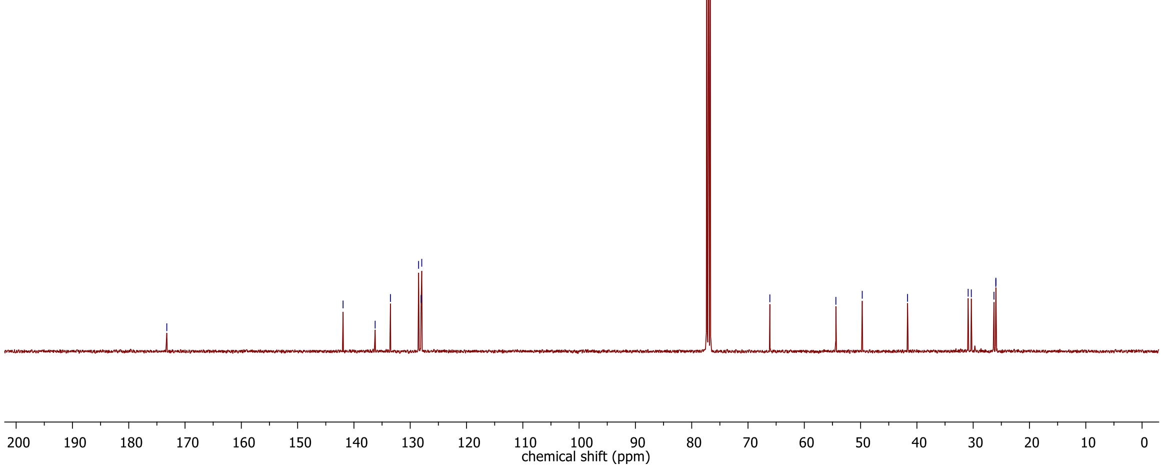

NOESY

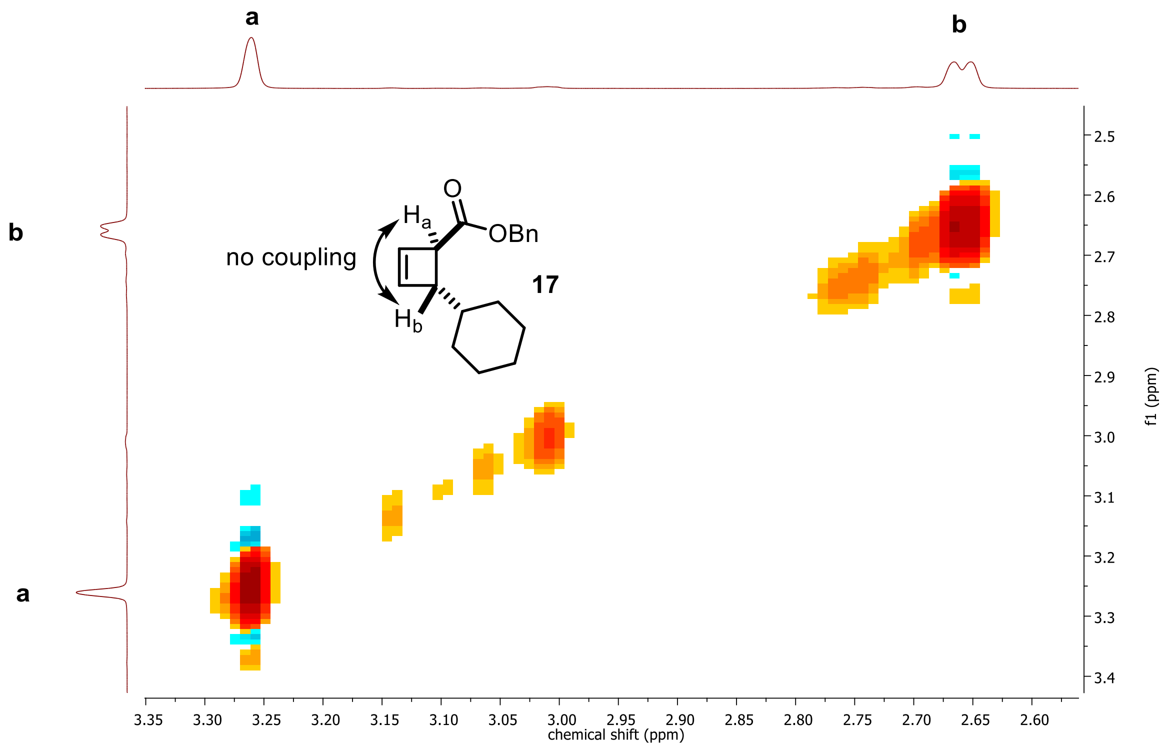

a
b
b
a
no coupling
H_a
O
OBn
17
H_b
2.5
2.6
2.7
2.8
2.9
3.0
3.1
3.2
3.3
3.4
f1 (ppm)
3.35
3.30
3.25
3.20
3.15
3.10
3.05
3.00
2.95
2.90
2.85
2.80
2.75
2.70
2.65
2.60
chemical shift (ppm)

6. X-Ray Crystallographic Data

The X-ray intensity data was measured on Bruker D8 Venture diffractometer equipped with multilayer monochromator, Mo K/α INCOATEC micro focus sealed tube and Oxford cooling system. The structure was solved by Direct Methods. Non-hydrogen atoms were refined with anisotropic displacement parameters. Hydrogen atoms were inserted at calculated positions and refined with riding model. The following software was used: Bruker SAINT software package[i] using a narrow-frame algorithm for frame integration, SADABS[ii] for absorption correction, OLEX2[iii] for structure solution, refinement, molecular diagrams and graphical user-interface, Shelxle[iv] for refinement and graphical user-interface SHELXS 2015[v] for structure solution, SHELXL-2015[vi] for refinement, Platon[vii] for symmetry check.
The crystals in all cases were grown by dissolving the sample in a minimum amount of chloroform, adding heptane and leaving the solvent system partially evaporate slowly under room temperature.

---

[i] Bruker SAINT v8.38B Copyright © 2005-2019 Bruker AXS
[ii] Sheldrick, G. M. (1996). *SADABS*. University of Göttingen, Germany.
[iii] Dolomanov, O.V., Bourhis, L.J., Gildea, R.J, Howard, J.A.K. & Puschmann, H. , OLEX2, (2009), J. Appl. Cryst. 42, 339-341.
[iv]C. B. Huebschle, G. M. Sheldrick and B. Dittrich, ShelXle: a Qt graphical user interface for SHELXL, J. Appl. Cryst., 44, (2011) 1281-1284.
[v] Sheldrick, G. M. (2015). *SHELXS v 2016/4* University of Göttingen, Germany.
[vi] Sheldrick, G. M. (2015). *SHELXL v 2016/4* University of Göttingen, Germany.
[vii] A. L. Spek, Acta Cryst. 2009, D65, 148-155.

Compound **2sa;** CCDC number: **2246979**

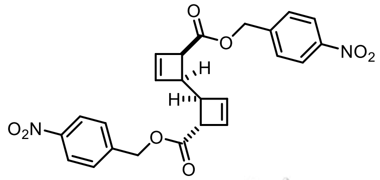

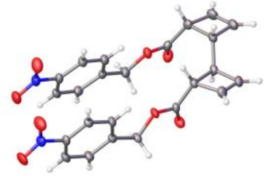

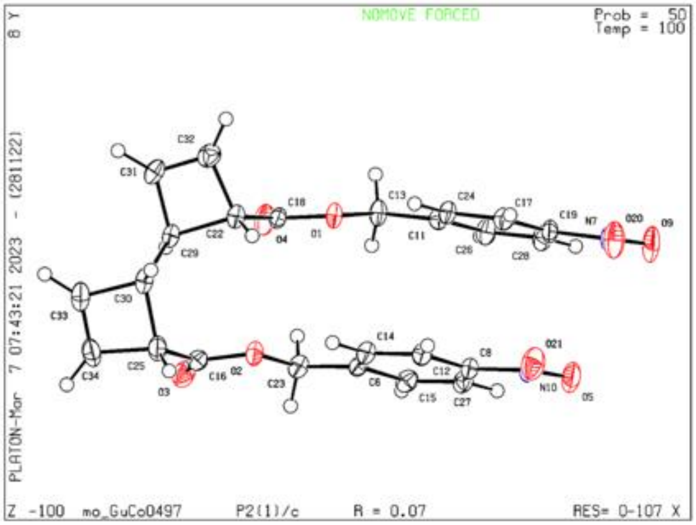

Bond precision: C-C = 0.0038 A Wavelength=0.71073

Cell:	a=14.944(7)	b=12.351(6)	c=12.323(7)
	alpha=90	beta=111.08(2)	gamma=90
Temperature:	100 K		

	Calculated	Reported
Volume	2122.3(19)	2122.2(18)
Space group	P 21/c	P2(1)/c
Hall group	-P 2ybc	-P 2ybc
Moiety formula	C24 H20 N2 O8	?
Sum formula	C24 H20 N2 O8	C24 H20 N2 O8
Mr	464.42	464.42
Dx,g cm-3	1.454	1.454
Z	4	4
Mu (mm-1)	0.111	0.111
F000	968.0	968.0
F000’	968.56	
h,k,lmax	21,17,17	21,17,17
Nref	6529	6212
Tmin,Tmax	0.989,0.995	0.639,0.746
Tmin’	0.983	

Correction method= # Reported T Limits: Tmin=0.639 Tmax=0.746
AbsCorr = NONE

Data completeness= 0.951 Theta(max)= 30.596

R(reflections)= 0.0669(3737) wR2(reflections)= 0.2075(6212)

S = 1.089 Npar= 307

Compound **4**; CCDC number: **2248994**

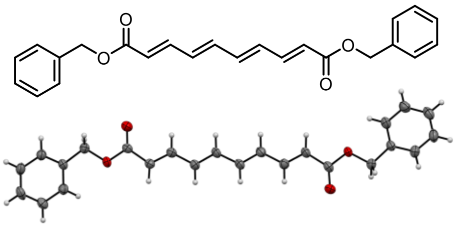

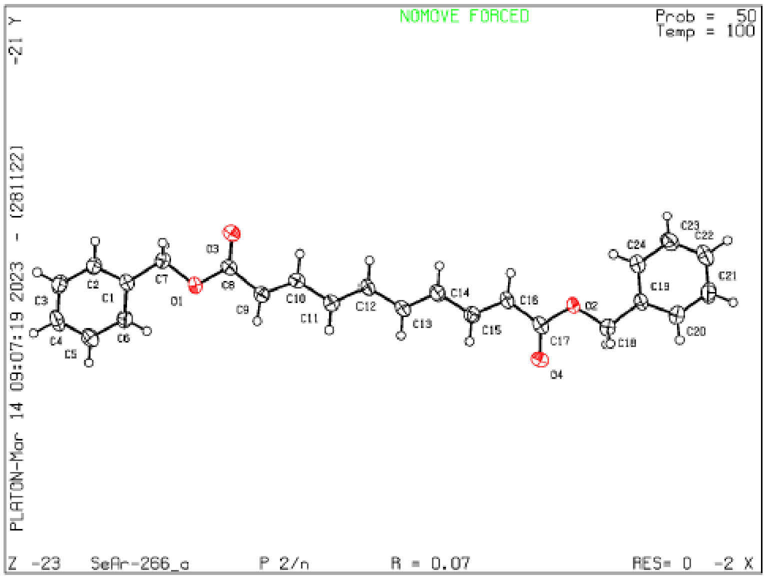

Bond precision: C-C = 0.0065 A Wavelength=0.71073

Cell:	a=18.113(4)	b=5.8485(10)	c=20.030(5)
	alpha=90	beta=112.758(19)	gamma=90
Temperature:	100 K		

	Calculated	Reported
Volume	1956.7(8)	1956.7(8)
Space group	P 2/n	P 2/n
Hall group	-P 2yac	-P 2yac
Moiety formula	C24 H22 O4	?
Sum formula	C24 H22 O4	C24 H22 O4
Mr	374.42	374.43
Dx,g cm-3	1.271	1.271
Z	4	4
Mu (mm-1)	0.086	0.086
F000	792.0	792.0
F000'	792.40	
h,k,lmax	22,7,25	22,7,25
Nref	4124	3954
Tmin,Tmax	0.992,0.994	0.411,0.999
Tmin'	0.973	

Correction method= # Reported T Limits: Tmin=0.411 Tmax=0.999
AbsCorr = MULTI-SCAN

Data completeness= 0.959 Theta(max)= 26.642

R(reflections)= 0.0742(1665) wR2(reflections)= 0.2662(3954)

S = 1.024 Npar= 253
